# Supplementary material for: Connecting intermediate phenotypes to disease using multi-omics in heart failure
Source: Pac Symp Biocomput. Author manuscript; Available in PMC 2025 Feb 13. (PMC11822568; doi:10.1142/9789819807024_0036)
Supplement: Supplementary Figure [file NIHMS2038838-supplement-Supplementary_Figure.pdf]

Supplemental Figures

Supplemental Figure 1: Genomic, Transcriptomic, and Proteomic Associations - Additional Phenotypes

MRI – LV Mass

A

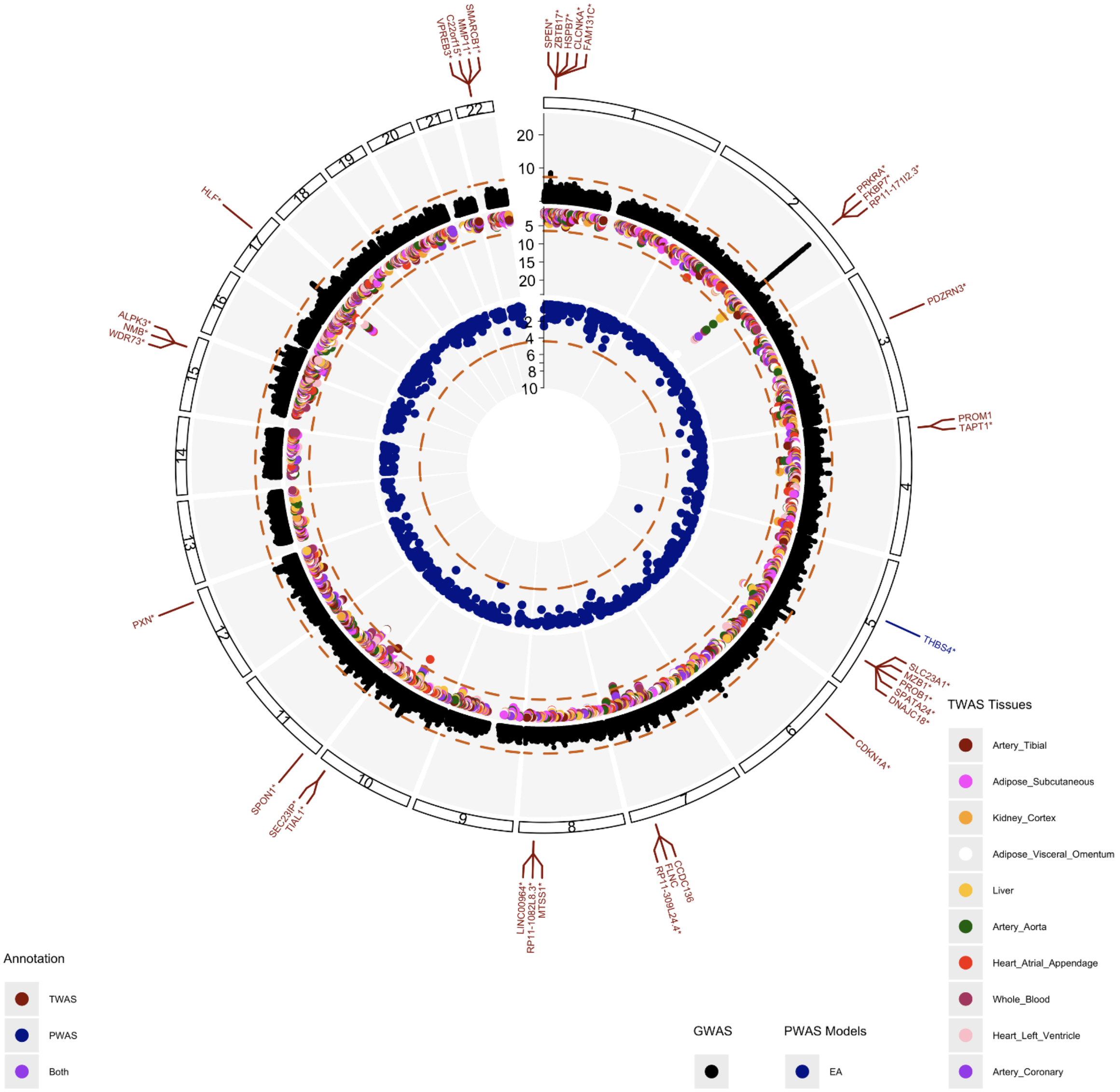

MRI – LV End-Systolic Volume

B

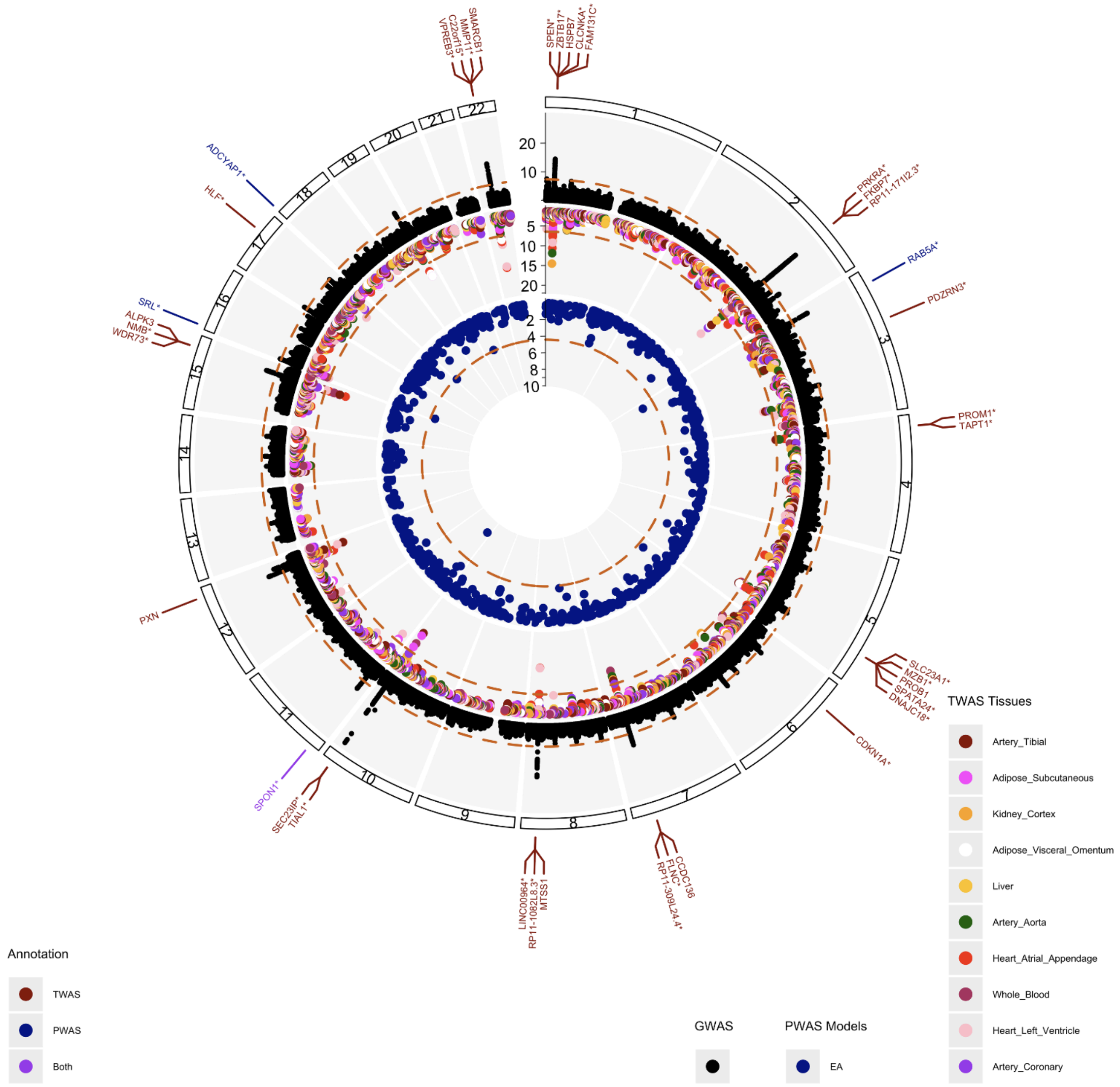

MRI – LV End-Diastolic Volume

C

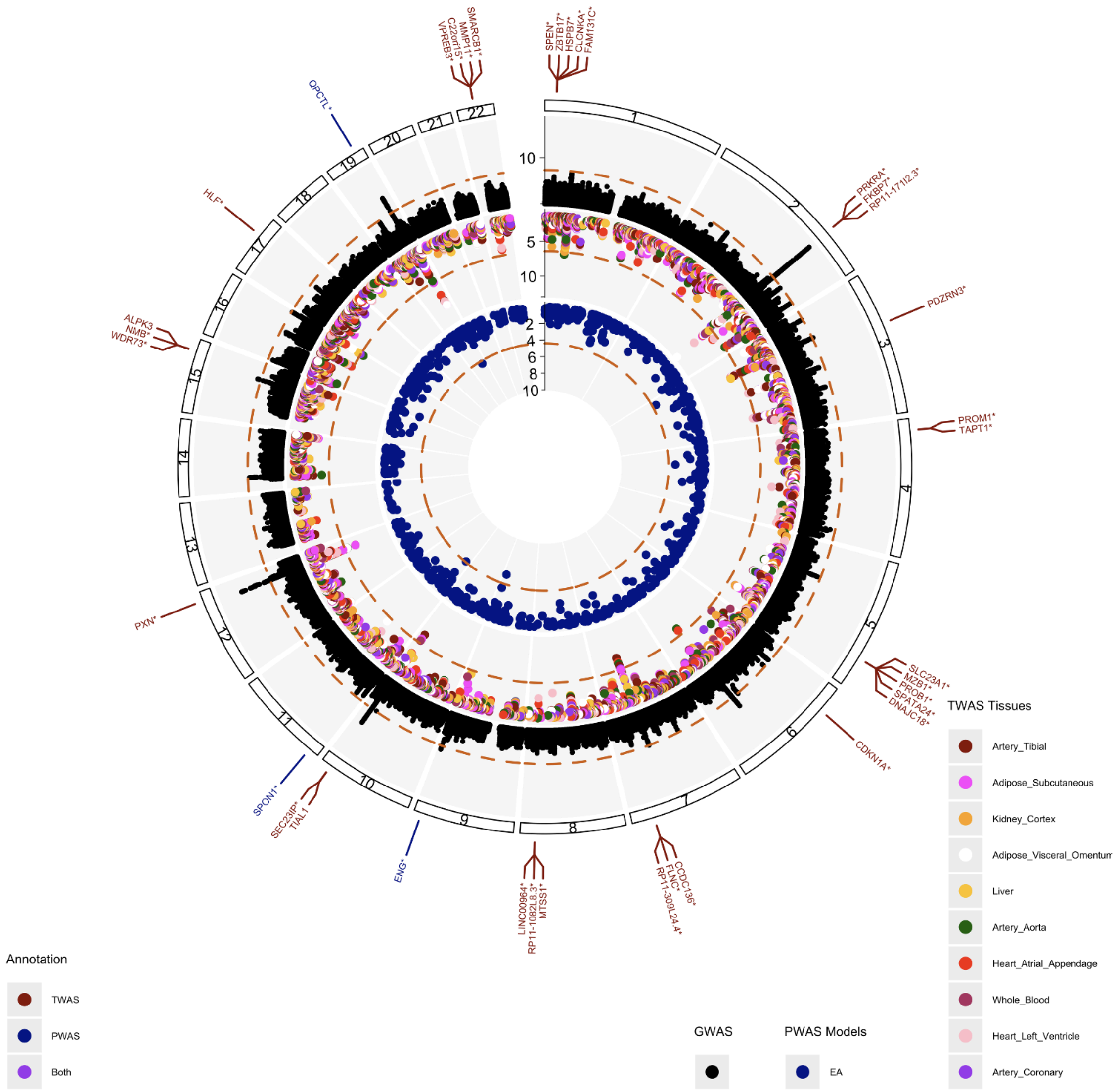

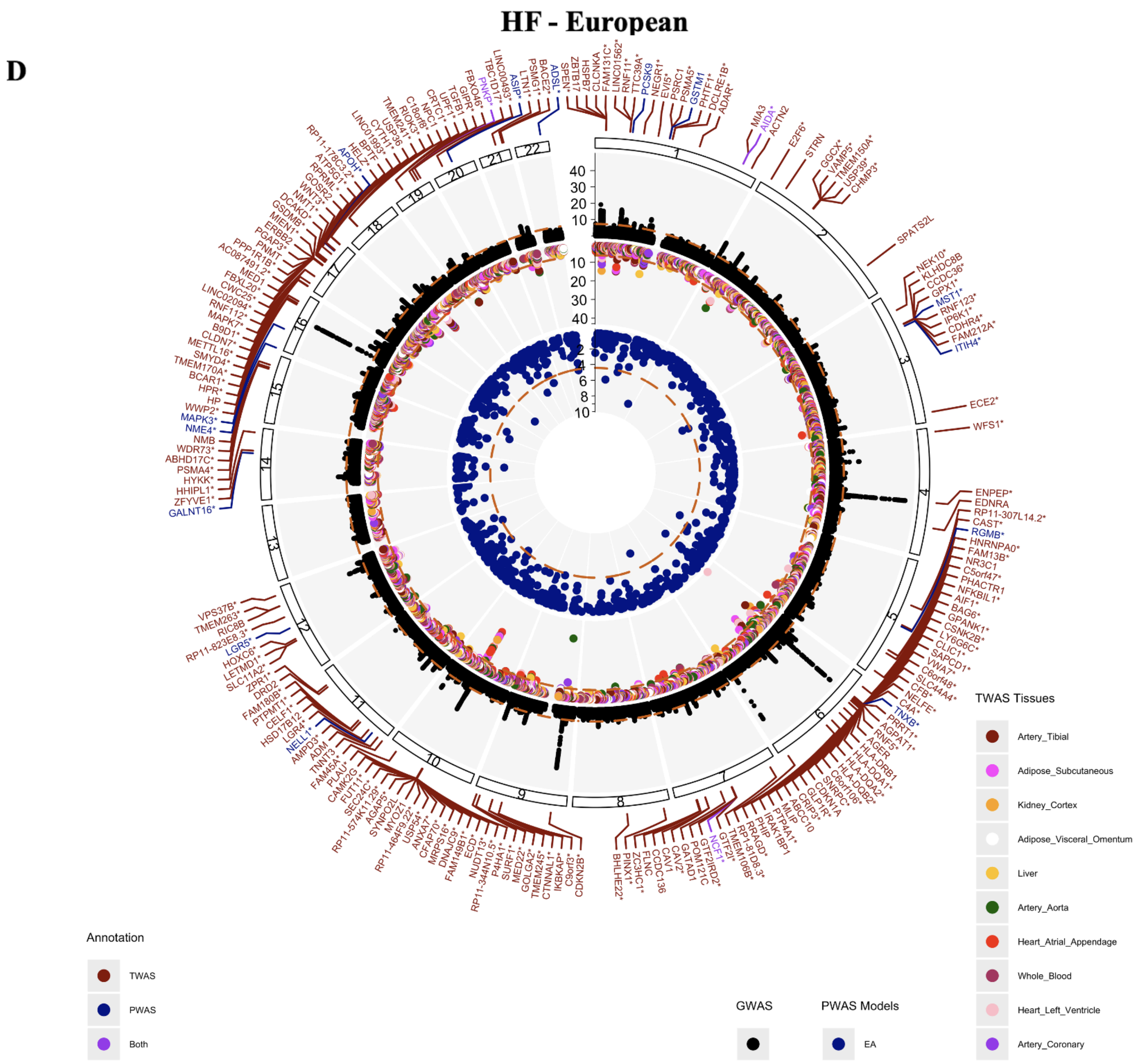

Supplemental Figure 1: Circos plots for A) MRI LV Mass, B) MRI LV End-Systolic Volume, C) MRI LV End-Diastolic Volume, and D) HF - European representing identified associations through GWAS (black), TWAS (red), and PWAS (blue).

Supplemental Figure 2: Gene-Set Enrichment Results - Additional Phenotypes

A

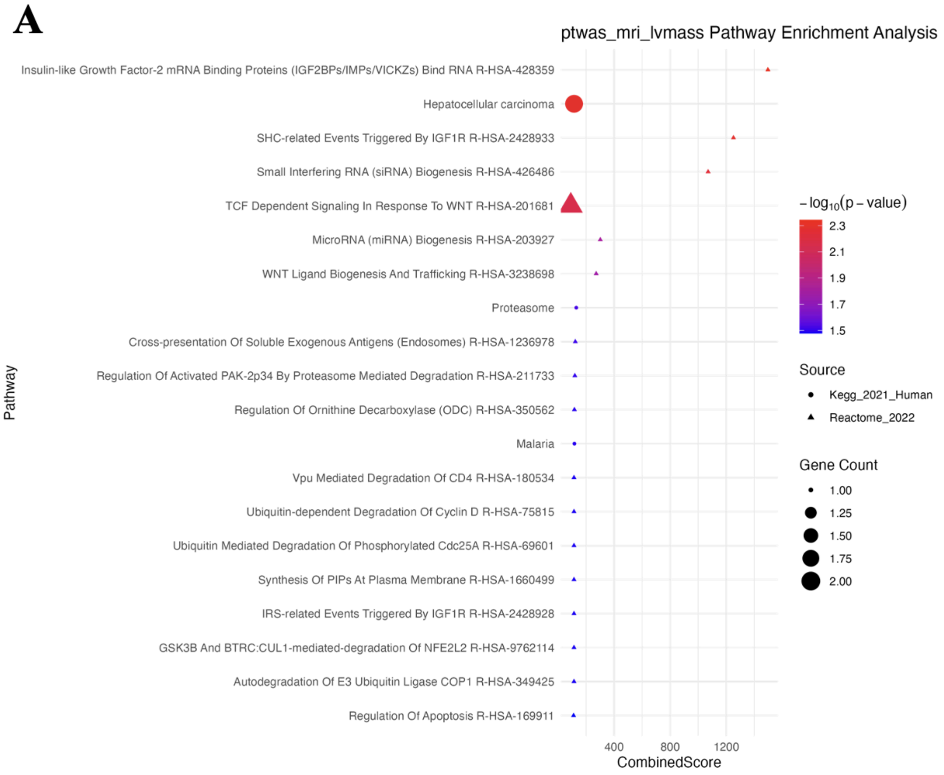

B

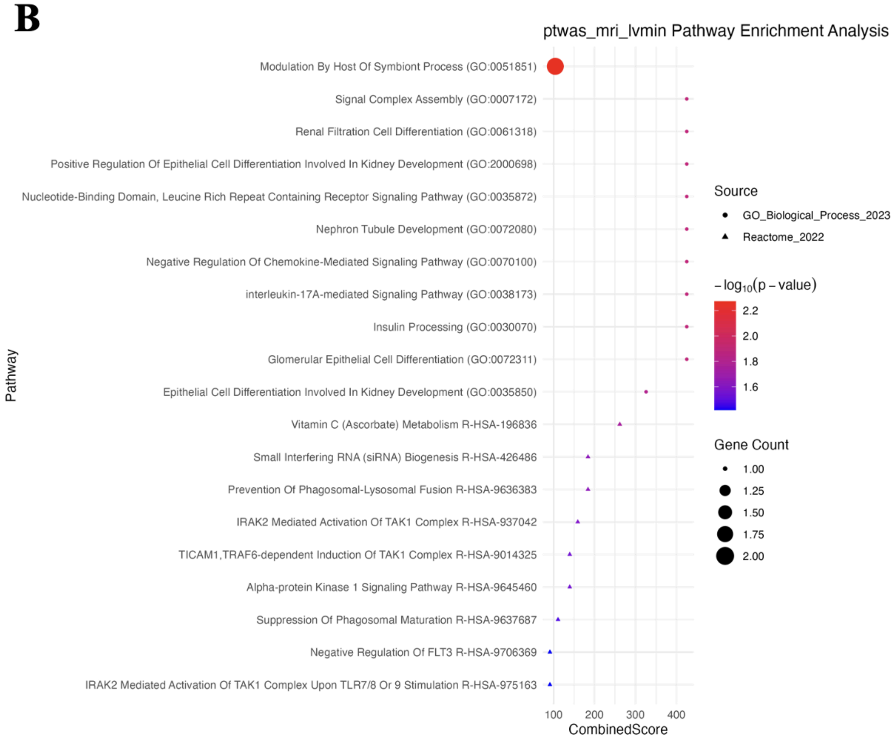

C

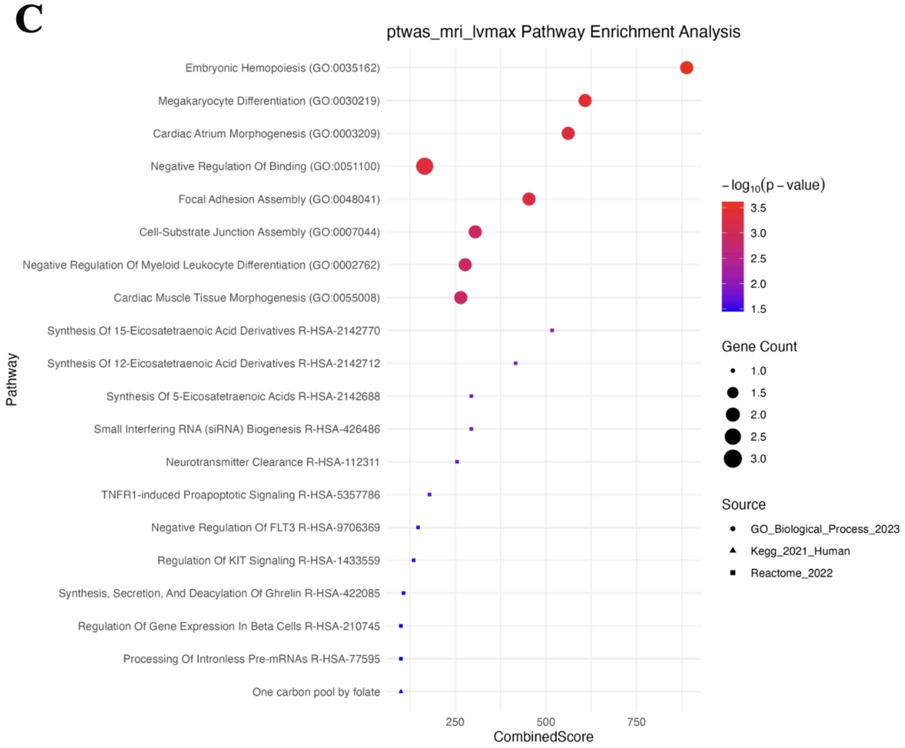

D

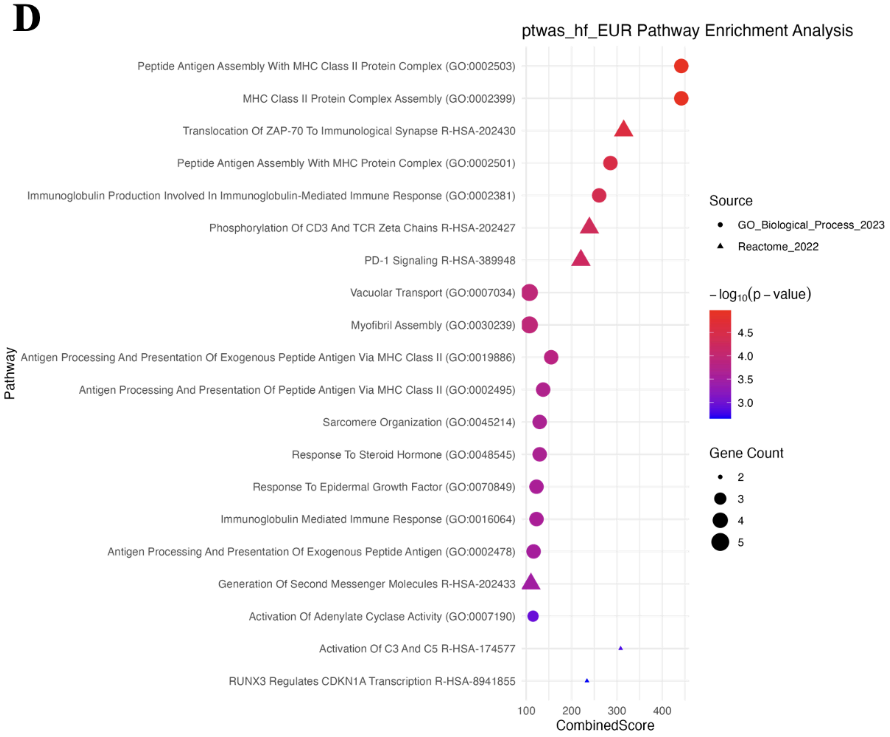

Supplemental Figure 2: Gene-set enrichment results of TWAS and PWAS significant hits for A) MRI LV Mass, B) MRI LV End-Systolic Volume, C) MRI LV End-Diastolic Volume, and D) HF - European phenotypes.

Supplemental Figure 3: PPI Networks - Additional Phenotypes

MRI – LV Mass PPI Network

A

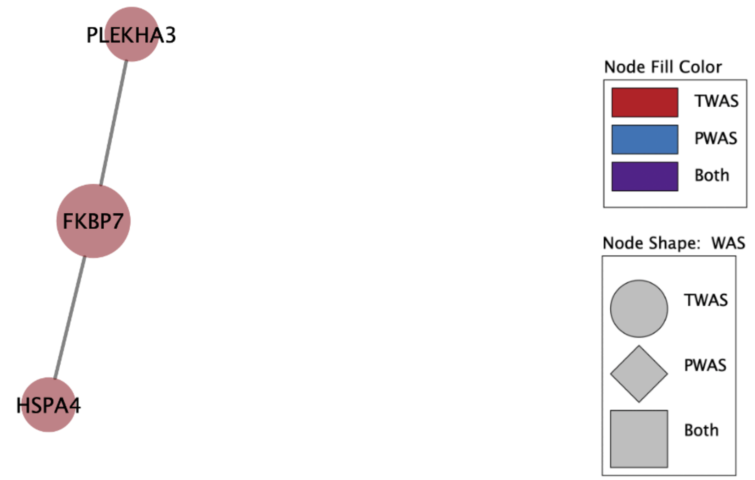

MRI – LVESV PPI Network

B

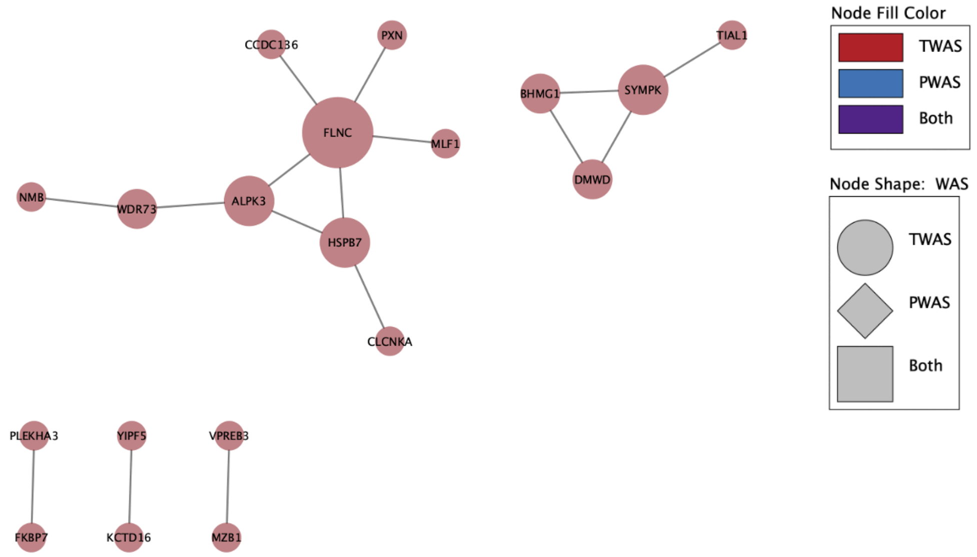

MRI – LVEDV PPI Network

C

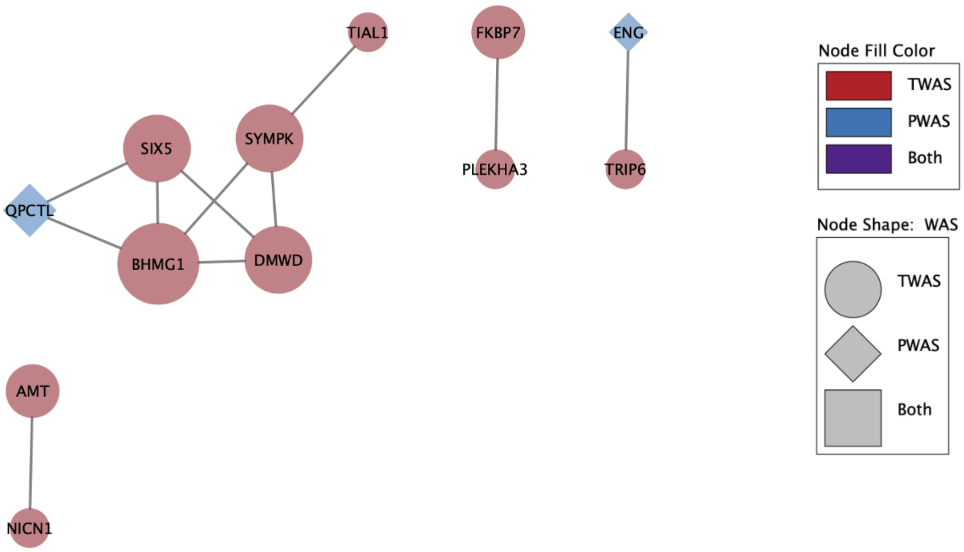

HF – EUR PPI Network

D

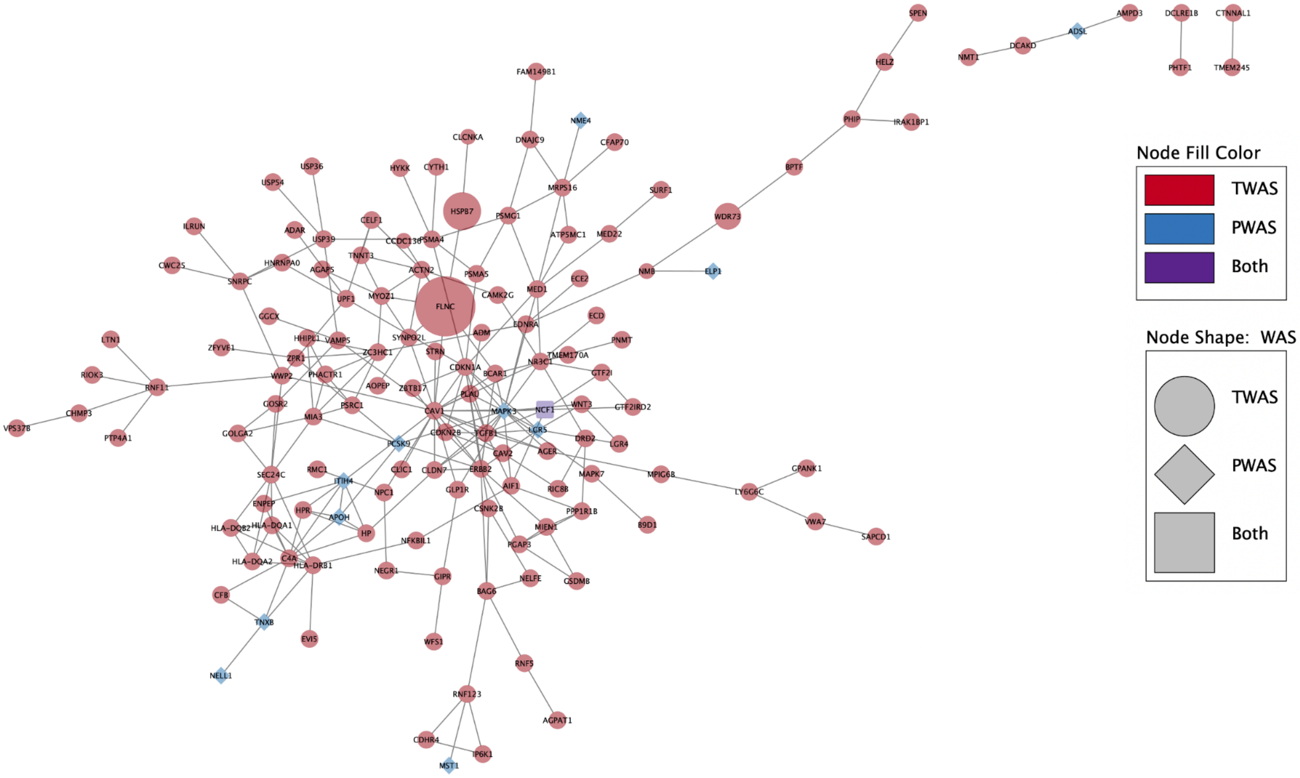

Supplemental Figure 3: PPI networks constructed using TWAS and PWAS significant hits for A) MRI LV Mass, B) MRI LV End-Systolic Volume, C) MRI LV End-Diastolic Volume, and D) HF - European phenotypes
