# Supplementary material for: Connecting intermediate phenotypes to disease using multi-omics in heart failure
Source: Pac Symp Biocomput. Author manuscript; Available in PMC 2025 Feb 13. (PMC11822568; doi:10.1142/9789819807024_0036)
Supplement: Supplemental Table 2 (A-F) [file NIHMS2038838-supplement-Supplemental_Table_2__A-F_.pdf]

Supplementary Table 2A: Left venticular ejection fraction (LVEF)

gene	gene_name	zscore	effect_size	pvalue	var_g	pred_perf_r2	pred_perf_pval	pred	n_snps_used	n_snps_in_cov	n_snps_in_model	best_gwas_p	largest_weight	Bonferroni_Threshold	Phenotype
ENSG00000262655	SPON1	-4.904	-0.086	9.412E-07	0.084	0.184	6.7e-322		131	131	135	2.600E-08	0.078	3.794E-05	invnorm_lvef
ENSG00000206372	C2	-4.823	-0.174	1.415E-06	0.014	0.119	6.677E-201		152	152	161	1.100E-06	0.106	3.794E-05	invnorm_lvef
PACAP	PACAP	4.302	0.806	1.693E-05	0.001	0.004	1.879E-07		21	21	21	3.900E-08	0.033	3.794E-05	invnorm_lvef

Supplementary Table 2B: Left venticular mass (LVM)																
gene	gene_name	zscore	effect_size	pvalue	var_g	pred_perf_r2	pred_perf_pval	pred_	n_snps_used	n_snps_in_cov	n_snps_in_model	best_gwas_p	largest_weight	Bonferroni_Threshold	Phenotype	
ENSG00000113296	THBS4	5.247	1.634	1.542E-07	0.029	0.127	6.479E-215		40	42	42	0.00053	0.110734562	3.79E-05	Khurshid_2023_v19_seg_lvmi_adjusted.bolt.impute	

ed.filtered

Supplementary Table 2C: Left venticular-end systolic volume (LVESV)															
gene	gene_name	zscore	effect_size	pvalue	var_g	pred_perf_r2	pred_perf_pval	pred_per	n_snps_used	n_snps_in_cov	n_snps_in_model	best_gwas_p	largest_weight	Bonferroni_Threshold	Phenotype
ENSG00000262655	SPON1	5.879	0.099	4.122E-09	0.084	0.184	6.7e-322		131	131	135	1.600E-11	0.078	3.794E-05	invnorm_min_lv_indexed
ENSG00000144566	RAB5A	-4.833	-0.717	1.348E-06	0.001	0.005	8.165E-10		8	8	9	2.300E-06	0.014	3.794E-05	invnorm_min_lv_indexed
ENSG00000185739	SRL	4.405	0.315	1.057E-05	0.004	0.013	5.489E-22		11	11	11	5.100E-05	0.045	3.794E-05	invnorm_min_lv_indexed
PACAP	PACAP	-4.216	-0.777	2.483E-05	0.001	0.004	1.879E-07		21	21	21	3.500E-07	0.033	3.794E-05	invnorm_min_lv_indexed

Supplementary Table 2D: Left venticular-end diastolic volume (LVEDV)																
gene	gene_name	zscore	effect_size	pvalue	var_g	pred_perf_r2	pred_perf_pval	pred_p	n_snps_used	n_snps_in_cov	n_snps_in_model	best_gwas_p	largest_weight	Bonferroni_Threshold	Phenotype
ENSG00000106991	ENG	-4.693	-0.210	2.691E-06	0.012	0.049	1.020E-80		41	41	41	3.800E-06	0.080	3.794E-05	invnorm_max_lv_indexed
ENSG00000011478	QPCTL	-4.298	-0.100	1.726E-05	0.039	0.120	8.326E-203		96	97	102	1.400E-07	0.174	3.794E-05	invnorm_max_lv_indexed
ENSG00000262655	SPON1	4.171	0.071	3.035E-05	0.084	0.184	6.7e-322		131	131	135	2.800E-07	0.078	3.794E-05	invnorm_max_lv_indexed

Supplementary Table 2E: All-cause heart failure in a multi-ancestry population																			
gene	gene_name	zscore	effect_size	pvalue	var_g	pred_perf_r2	pred_perf_pval	pred_per	n_snps_used	n_snps_in_cov	n_snps_in_model	best_gwas_p	largest_weight	Bonferroni_Threshold	Phenotype	Model	Ancestry
ENSG00000186063	AIDA	-5.810	-0.181	6.258E-09	0.006	0.028	6.720E-47		17	17	18	3.592E-09	0.018	3.794E-05	HF	EA	multiancestry
ENSG00000103202	NME4	5.541	0.181	3.013E-08	0.007	0.027	7.898E-46		65	65	66	1.786E-07	0.075	3.794E-05	HF	EA	multiancestry
ENSG00000091583	APOH	-5.465	-0.144	4.630E-08	0.016	0.167	3.826E-288		40	40	43	6.080E-09	0.191	3.794E-05	HF	EA	multiancestry
ENSG00000158517	NCF1	5.200	0.112	1.994E-07	0.022	0.139	6.799E-237		113	113	123	3.450E-09	0.161	3.794E-05	HF	EA	multiancestry
ENSG00000169174	PCSK9	4.878	0.196	1.069E-06	0.005	0.043	1.696E-70		42	42	45	1.907E-04	0.153	3.794E-05	HF	EA	multiancestry
ENSG00000229341	TNXB	4.802	0.072	1.568E-06	0.032	0.253	0.000E+00		116	116	123	6.173E-09	0.182	3.794E-05	HF	EA	multiancestry
ENSG00000070614	NDST1	4.600	0.141	4.232E-06	0.008	0.045	1.393E-73		18	18	18	4.650E-06	0.076	3.794E-05	HF	EA	multiancestry
ENSG00000174136	RGMB	-4.571	-0.070	4.847E-06	0.026	0.090	9.357E-150		68	68	72	1.200E-05	0.038	3.794E-05	HF	EA	multiancestry
ENSG00000165661	QSOX2	-4.545	-0.060	5.495E-06	0.045	0.152	2.507E-260		39	39	40	7.535E-06	0.200	3.794E-05	HF	EA	multiancestry
ENSG00000101440	ASIP	-4.543	-0.096	5.557E-06	0.010	0.132	2.456E-224		27	27	28	1.781E-06	0.224	3.794E-05	HF	EA	multiancestry
ENSG00000118849	RARRES1	4.521	0.039	6.146E-06	0.090	0.309	0.000E+00		62	62	63	8.003E-08	0.256	3.794E-05	HF	EA	multiancestry
ENSG00000039650	PNKP	4.505	0.456	6.631E-06	0.001	0.008	9.100E-14		17	17	18	2.193E-09	0.031	3.794E-05	HF	EA	multiancestry
ENSG00000100626	GALNT16	-4.494	-0.064	6.996E-06	0.035	0.147	4.190E-252		88	88	90	3.167E-08	0.169	3.794E-05	HF	EA	multiancestry
ENSG00000239900	ADSL	-4.354	-1.202	1.336E-05	0.000	0.003	9.356E-07		4	4	7	2.398E-05	0.014	3.794E-05	HF	EA	multiancestry
ENSG00000133789	SWAP70	-4.340	-0.049	1.424E-05	0.056	0.209	0.000E+00		62	62	65	1.180E-07	0.111	3.794E-05	HF	EA	multiancestry
ENSG00000165973	NELL1	4.315	0.104	1.598E-05	0.011	0.123	1.160E-207		45	45	46	1.223E-05	0.119	3.794E-05	HF	EA	multiancestry
ENSG00000139292	LGR5	4.305	0.163	1.667E-05	0.004	0.023	4.913E-38		75	75	76	1.069E-02	0.058	3.794E-05	HF	EA	multiancestry
ENSG00000236315	NCR3	4.167	0.101	3.085E-05	0.012	0.109	7.310E-183		97	97	98	6.383E-09	0.148	3.794E-05	HF	EA	multiancestry
ENSG00000186063	AIDA	-5.521	-0.315	3.373E-08	0.002	0.005	9.660E-04		13	17	25	6.896E-09	0.019	3.655E-05	HF	AA	multiancestry
ENSG00000135218	CD36	-5.211	-0.191	1.882E-07	0.062	0.345	5.395E-174		108	111	124	4.270E-08	0.253	3.655E-05	HF	AA	multiancestry
VPS24	VPS24	-4.952	-0.221	7.358E-07	0.004	0.030	1.699E-14		18	21	22	3.199E-07	0.053	3.655E-05	HF	AA	multiancestry
ENSG00000118849	RARRES1	4.907	0.066	9.258E-07	0.077	0.377	9.935E-195		68	70	71	9.064E-08	0.146	3.655E-05	HF	AA	multiancestry
ENSG00000140403	DNAJA4	4.907	0.641	9.260E-07	0.001	0.006	6.921E-04		21	26	28	5.893E-08	0.020	3.655E-05	HF	AA	multiancestry
ENSG00000233192	HLA-DQA2	-4.802	-0.087	1.574E-06	0.042	0.174	6.225E-80		65	72	74	5.351E-07	0.064	3.655E-05	HF	AA	multiancestry
ENSG00000112139	MDGA1	-4.761	-0.230	1.927E-06	0.004	0.435	1.601E-234		110	115	134	1.633E-06	0.045	3.655E-05	HF	AA	multiancestry
ENSG00000133789	SWAP70	-4.740	-0.066	2.142E-06	0.040	0.220	4.782E-103		42	45	54	5.972E-07	0.117	3.655E-05	HF	AA	multiancestry
ENSG00000184232	OAF	4.554	0.106	5.254E-06	0.019	0.221	1.757E-103		95	96	103	9.655E-06	0.073	3.655E-05	HF	AA	multiancestry
ENSG00000116005	PCYOX1	-4.302	-0.301	1.692E-05	0.012	0.226	3.132E-106		66	70	72	4.184E-07	0.123	3.655E-05	HF	AA	multiancestry
ENSG00000236315	NCR3	4.135	0.166	3.543E-05	0.004	0.062	3.159E-28		52	52	53	8.136E-07	0.112	3.655E-05	HF	AA	multiancestry

Supplementary Table 2F: All-cause heart failure in a European population																	
gene	gene_name	zscore	effect_size	pvalue	var_g	pred_perf_r2	pred_perf_pval	pred_perf	n_snps_used	n_snps_in_cov	n_snps_in_model	best_gwas_p	largest_weight	Bonferroni_Thres	Phenotype	Model	Ancestry
ENSG00000186063	AIDA	-5.751	-0.199	8.887E-09	0.006	0.028	6.720E-47		17	17	18	2.182E-08	0.018	3.794E-05	HF	EA	EUR
ENSG00000091583	APOH	-5.621	-0.156	1.895E-08	0.016	0.167	3.826E-288		40	40	43	1.644E-07	0.191	3.794E-05	HF	EA	EUR
ENSG00000158517	NCF1	5.145	0.118	2.677E-07	0.022	0.139	6.799E-237		113	113	123	4.207E-07	0.161	3.794E-05	HF	EA	EUR
ENSG00000100626	GALNT16	-4.977	-0.075	6.468E-07	0.035	0.147	4.190E-252		88	88	90	6.042E-09	0.169	3.794E-05	HF	EA	EUR
ENSG00000103202	NME4	4.925	0.183	8.433E-07	0.007	0.027	7.898E-46		65	65	66	1.691E-06	0.075	3.794E-05	HF	EA	EUR
ENSG00000229341	TNXB	4.863	0.077	1.154E-06	0.032	0.253	0.000E+00		116	116	123	2.962E-09	0.182	3.794E-05	HF	EA	EUR
ENSG00000039650	PNKP	4.856	0.544	1.196E-06	0.001	0.008	9.100E-14		17	17	18	9.102E-11	0.031	3.794E-05	HF	EA	EUR
ENSG00000169174	PCSK9	4.823	0.208	1.412E-06	0.005	0.043	1.696E-70		42	42	45	3.042E-04	0.153	3.794E-05	HF	EA	EUR
ENSG00000174136	RGMB	-4.762	-0.080	1.921E-06	0.026	0.090	9.357E-150		68	68	72	8.665E-06	0.038	3.794E-05	HF	EA	EUR
G6B	G6B	-4.742	-0.517	2.112E-06	0.001	0.003	9.821E-06		21	21	23	5.419E-14	0.014	3.794E-05	HF	EA	EUR
ENSG00000173531	MST1	4.631	0.031	3.642E-06	0.178	0.511	0.000E+00		59	59	62	3.159E-07	0.266	3.794E-05	HF	EA	EUR
ENSG00000102882	MAPK3	-4.606	-0.048	4.111E-06	0.092	0.219	0.000E+00		36	36	37	4.633E-07	0.121	3.794E-05	HF	EA	EUR
ENSG00000101440	ASIP	-4.521	-0.098	6.158E-06	0.010	0.132	2.456E-224		27	27	28	1.543E-06	0.224	3.794E-05	HF	EA	EUR
ENSG00000165973	NELL1	4.269	0.113	1.961E-05	0.011	0.123	1.160E-207		45	45	46	8.503E-06	0.119	3.794E-05	HF	EA	EUR
ENSG00000134184	GSTM1	4.245	0.069	2.186E-05	0.035	0.301	0.000E+00		182	182	189	6.973E-14	0.120	3.794E-05	HF	EA	EUR
ENSG00000055955	ITIH4	4.232	0.207	2.319E-05	0.003	0.019	1.192E-31		42	42	44	1.132E-04	0.054	3.794E-05	HF	EA	EUR
ENSG00000139292	LGR5	4.204	0.174	2.618E-05	0.004	0.023	4.913E-38		75	75	76	1.675E-03	0.058	3.794E-05	HF	EA	EUR
ENSG00000239900	ADSL	-4.195	-1.227	2.734E-05	0.000	0.003	9.356E-07		4	4	7	9.003E-05	0.014	3.794E-05	HF	EA	EUR
