# Supplementary material for: Connecting intermediate phenotypes to disease using multi-omics in heart failure
Source: Pac Symp Biocomput. Author manuscript; Available in PMC 2025 Feb 13. (PMC11822568; doi:10.1142/9789819807024_0036)
Supplement: Supplemental Table 3 (A-F) [file NIHMS2038838-supplement-Supplemental_Table_3__A-F_.pdf]

Supplementary Table 3A: Left venticular ejectrion fraction (LVEF)

Pathway	pvalue	GeneCount	CombinedScore	Source	GeneCountCount	log10_pvalue
Positive Regulation Of Actin Filament Bundle Assembly (GO:0032233)	4.226E-03	PXN;MTSS1	123.416	GO_Biological_Process_2023	2	2.374
TFAP2 (AP-2) Family Regulates Transcription Of Cell Cycle Factors R-HSA-8866911	9.465E-03	CDKN1A	628.431	Reactome_2022	1	2.024
Glomerular Epithelial Cell Differentiation (GO:0072311)	9.465E-03	PROM1	628.431	GO_Biological_Process_2023	1	2.024
Renal Filtration Cell Differentiation (GO:0061318)	9.465E-03	PROM1	628.431	GO_Biological_Process_2023	1	2.024
Nephron Tubule Development (GO:0072080)	9.465E-03	MTSS1	628.431	GO_Biological_Process_2023	1	2.024
Signal Complex Assembly (GO:0007172)	9.465E-03	PXN	628.431	GO_Biological_Process_2023	1	2.024
Positive Regulation Of Epithelial Cell Differentiation Involved In Kidney Development (GO:2000698)	9.465E-03	PROM1	628.431	GO_Biological_Process_2023	1	2.024
Epithelial Cell Differentiation Involved In Kidney Development (GO:0035850)	1.135E-02	MTSS1	483.151	GO_Biological_Process_2023	1	1.945
Regulation Of Respiratory Gaseous Exchange (GO:0043576)	1.135E-02	NMB	483.151	GO_Biological_Process_2023	1	1.945
Transepithelial Chloride Transport (GO:0030321)	1.135E-02	CLCNKA	483.151	GO_Biological_Process_2023	1	1.945
Adherens Junction Maintenance (GO:0034334)	1.135E-02	MTSS1	483.151	GO_Biological_Process_2023	1	1.945
Vitamin C (Ascorbate) Metabolism R-HSA-196836	1.323E-02	SLC23A1	388.832	Reactome_2022	1	1.879
RUNX3 Regulates CDKN1A Transcription R-HSA-8941855	1.323E-02	CDKN1A	388.832	Reactome_2022	1	1.879
Stress-Induced Premature Senescence (GO:0090400)	1.323E-02	CDKN1A	388.832	GO_Biological_Process_2023	1	1.879
Positive Regulation Of Apoptotic Cell Clearance (GO:2000427)	1.323E-02	C2	388.832	GO_Biological_Process_2023	1	1.879
siRNA Processing (GO:0030422)	1.323E-02	PRKRA	388.832	GO_Biological_Process_2023	1	1.879
Integrin Activation (GO:0033622)	1.510E-02	MZB1	323.051	GO_Biological_Process_2023	1	1.821
Response To Misfolded Protein (GO:0051788)	1.510E-02	DNAJC18	323.051	GO_Biological_Process_2023	1	1.821
Regulation Of Nephron Tubule Epithelial Cell Differentiation (GO:0072182)	1.510E-02	PROM1	323.051	GO_Biological_Process_2023	1	1.821
Small Interfering RNA (siRNA) Biogenesis R-HSA-426486	1.697E-02	PRKRA	274.777	Reactome_2022	1	1.770
Positive Regulation Of Hippo Signaling (GO:0035332)	1.697E-02	TIAL1	274.777	GO_Biological_Process_2023	1	1.770
Peptidyl-Amino Acid Modification (GO:0018193)	1.697E-02	FKBP7	274.777	GO_Biological_Process_2023	1	1.770
Positive Regulation Of miRNA-mediated Gene Silencing (GO:2000637)	1.697E-02	TIAL1	274.777	GO_Biological_Process_2023	1	1.770
Positive Regulation Of Transcription Of Nucleolar Large rRNA By RNA Polymerase I (GO:1901838)	1.697E-02	SMARCB1	274.777	GO_Biological_Process_2023	1	1.770
STAT5 Activation Downstream Of FLT3 ITD Mutants R-HSA-9702518	1.884E-02	CDKN1A	237.977	Reactome_2022	1	1.725
Positive Regulation Of Post-Transcriptional Gene Silencing By RNA (GO:1900370)	1.884E-02	TIAL1	237.977	GO_Biological_Process_2023	1	1.725
Positive Regulation Of Lipid Transport (GO:0032370)	2.071E-02	NMB	209.078	GO_Biological_Process_2023	1	1.684
Cellular Response To Fluid Shear Stress (GO:0071498)	2.071E-02	MTSS1	209.078	GO_Biological_Process_2023	1	1.684
Cellular Response To Growth Hormone Stimulus (GO:0071378)	2.071E-02	PXN	209.078	GO_Biological_Process_2023	1	1.684
Podocyte Differentiation (GO:0072112)	2.071E-02	PROM1	209.078	GO_Biological_Process_2023	1	1.684
Eye Photoreceptor Cell Differentiation (GO:0001754)	2.257E-02	PROM1	185.842	GO_Biological_Process_2023	1	1.646

Pathway	pvalue	GeneCount	CombinedScore	Source	GeneCountCount	log10_pvalue
Cell-Cell Junction Maintenance (GO:0045217)	2.257E-02	MTSS1	185.842	GO_Biological_Process_2023	1	1.646
pre-miRNA Processing (GO:0031054)	2.257E-02	PRKRA	185.842	GO_Biological_Process_2023	1	1.646
Regulation Of Transcription Of Nucleolar Large rRNA By RNA Polymerase I (GO:1901836)	2.443E-02	SMARCB1	166.791	GO_Biological_Process_2023	1	1.612
AKT Phosphorylates Targets In Cytosol R-HSA-198323	2.628E-02	CDKN1A	150.918	Reactome_2022	1	1.580
TP53 Regulates Transcription Of Genes Involved In G1 Cell Cycle Arrest R-HSA-6804116	2.628E-02	CDKN1A	150.918	Reactome_2022	1	1.580
Cardiac Cell Development (GO:0055006)	2.628E-02	ALPK3	150.918	GO_Biological_Process_2023	1	1.580
Signaling By FLT3 ITD And TKD Mutants R-HSA-9703648	2.813E-02	CDKN1A	137.510	Reactome_2022	1	1.551
Stem Cell Development (GO:0048864)	2.813E-02	TAPT1	137.510	GO_Biological_Process_2023	1	1.551
Regulation Of Hormone Secretion (GO:0046883)	2.813E-02	NMB	137.510	GO_Biological_Process_2023	1	1.551
Regulation Of miRNA-mediated Gene Silencing (GO:0060964)	2.813E-02	TIAL1	137.510	GO_Biological_Process_2023	1	1.551
Cell-extracellular Matrix Interactions R-HSA-446353	2.998E-02	FLNC	126.050	Reactome_2022	1	1.523
Positive Regulation By Host Of Viral Transcription (GO:0043923)	2.998E-02	SMARCB1	126.050	GO_Biological_Process_2023	1	1.523
FOXO-mediated Transcription Of Cell Cycle Genes R-HSA-9617828	3.183E-02	CDKN1A	116.154	Reactome_2022	1	1.497
Aberrant Regulation Of Mitotic G1/S Transition In Cancer Due To RB1 Defects R-HSA-9659787	3.183E-02	CDKN1A	116.154	Reactome_2022	1	1.497
Formation Of Senescence-Associated Heterochromatin Foci (SAHF) R-HSA-2559584	3.183E-02	CDKN1A	116.154	Reactome_2022	1	1.497
DNA Damage Response, Signal Transduction By P53 Class Mediator Resulting In Cell Cycle Arrest (GO:0006977)	3.183E-02	CDKN1A	116.154	GO_Biological_Process_2023	1	1.497
Positive Regulation Of Leukocyte Proliferation (GO:0070665)	3.183E-02	NMB	116.154	GO_Biological_Process_2023	1	1.497
Transepithelial Transport (GO:0070633)	3.183E-02	CLCNKA	116.154	GO_Biological_Process_2023	1	1.497
Growth Hormone Receptor Signaling Pathway (GO:0060396)	3.183E-02	PXN	116.154	GO_Biological_Process_2023	1	1.497
Positive Regulation Of Cartilage Development (GO:0061036)	3.183E-02	TAPT1	116.154	GO_Biological_Process_2023	1	1.497
Negative Regulation Of Vascular Associated Smooth Muscle Cell Proliferation (GO:1904706)	3.183E-02	CDKN1A	116.154	GO_Biological_Process_2023	1	1.497
Regulation Of Cartilage Development (GO:0061035)	3.183E-02	TAPT1	116.154	GO_Biological_Process_2023	1	1.497
Signaling By FLT3 Fusion Proteins R-HSA-9703465	3.367E-02	CDKN1A	107.533	Reactome_2022	1	1.473
Renal Absorption (GO:0070293)	3.367E-02	CLCNKA	107.533	GO_Biological_Process_2023	1	1.473
Regulation Of Lymphocyte Proliferation (GO:0050670)	3.367E-02	MZB1	107.533	GO_Biological_Process_2023	1	1.473
Neural Crest Cell Differentiation (GO:0014033)	3.367E-02	TAPT1	107.533	GO_Biological_Process_2023	1	1.473
Regulation Of Cell Cycle G2/M Phase Transition (GO:1902749)	3.367E-02	CDKN1A	107.533	GO_Biological_Process_2023	1	1.473
Nucleosome Disassembly (GO:0006337)	3.367E-02	SMARCB1	107.533	GO_Biological_Process_2023	1	1.473
Positive Regulation Of Hormone Secretion (GO:0046887)	3.550E-02	NMB	99.962	GO_Biological_Process_2023	1	1.450
Cellular Response To Misfolded Protein (GO:0071218)	3.550E-02	DNAJC18	99.962	GO_Biological_Process_2023	1	1.450
Negative Regulation Of Cyclin-Dependent Protein Serine/Threonine Kinase Activity (GO:0045736)	3.550E-02	CDKN1A	99.962	GO_Biological_Process_2023	1	1.450
protein-DNA Complex Disassembly (GO:0032986)	3.734E-02	SMARCB1	93.267	GO_Biological_Process_2023	1	1.428
Negative Regulation Of Phosphate Metabolic Process (GO:0045936)	3.734E-02	CDKN1A	93.267	GO_Biological_Process_2023	1	1.428

Pathway	pvalue	GeneCount	CombinedScore	Source	GeneCountCount	log10_pvalue
Negative Regulation Of Cyclin-Dependent Protein Kinase Activity (GO:1904030)	3.734E-02	CDKN1A	93.267	GO_Biological_Process_2023	1	1.428

Supplementary Table 3B: Left venticular mass (LVM)

Pathway	pvalue	GeneCount	CombinedScore	Source	GeneCountCount	log10_pvalue
Positive Regulation Of Endothelial Cell Proliferation (GO:0001938)	1.110E-03	IGF2;THBS4	328.401	GO_Biological_Process_2023	2	2.955
Regulation Of Peptidyl-Tyrosine Phosphorylation (GO:0050730)	1.512E-03	IGF2;THBS4	266.994	GO_Biological_Process_2023	2	2.820
Positive Regulation Of Peptidyl-Tyrosine Phosphorylation (GO:0050731)	3.120E-03	IGF2;THBS4	162.759	GO_Biological_Process_2023	2	2.506
Regulation Of Glycogen (Starch) Synthase Activity (GO:2000465)	3.246E-03	IGF2	2385.602	GO_Biological_Process_2023	1	2.489
Insulin-like Growth Factor-2 mRNA Binding Proteins (IGF2BPs/IMPs/VICKZs) Bind RNA R-HSA-428359	4.542E-03	IGF2	1497.033	Reactome_2022	1	2.343
Myoblast Migration (GO:0051451)	4.542E-03	THBS4	1497.033	GO_Biological_Process_2023	1	2.343
Negative Regulation Of Muscle Cell Differentiation (GO:0051148)	4.542E-03	IGF2	1497.033	GO_Biological_Process_2023	1	2.343
siRNA Processing (GO:0030422)	4.542E-03	PRKRA	1497.033	GO_Biological_Process_2023	1	2.343
Hepatocellular carcinoma	5.148E-03	IGF2;WNT3	114.391	Kegg_2021_Human	2	2.288
SHC-related Events Triggered By IGF1R R-HSA-2428933	5.189E-03	IGF2	1251.416	Reactome_2022	1	2.285
Embryonic Placenta Development (GO:0001892)	5.189E-03	IGF2	1251.416	GO_Biological_Process_2023	1	2.285
Small Interfering RNA (siRNA) Biogenesis R-HSA-426486	5.836E-03	PRKRA	1070.484	Reactome_2022	1	2.234
Muscle Cell Migration (GO:0014812)	5.836E-03	THBS4	1070.484	GO_Biological_Process_2023	1	2.234
Peptidyl-Amino Acid Modification (GO:0018193)	5.836E-03	FKBP7	1070.484	GO_Biological_Process_2023	1	2.234
Wnt Signaling Pathway Involved In Midbrain Dopaminergic Neuron Differentiation (GO:1904953)	5.836E-03	WNT3	1070.484	GO_Biological_Process_2023	1	2.234
Regulation Of Gene Expression By Genomic Imprinting (GO:0006349)	6.482E-03	IGF2	932.060	GO_Biological_Process_2023	1	2.188
TCF Dependent Signaling In Response To WNT R-HSA-201681	7.079E-03	PSMC3;WNT3	90.888	Reactome_2022	2	2.150
Epithelial Cell-Cell Adhesion (GO:0090136)	7.129E-03	THBS4	822.995	GO_Biological_Process_2023	1	2.147
Negative Regulation Of calcineurin-NFAT Signaling Cascade (GO:0070885)	7.129E-03	MYOZ1	822.995	GO_Biological_Process_2023	1	2.147
Negative Regulation Of Calcineurin-Mediated Signaling (GO:0106057)	7.129E-03	MYOZ1	822.995	GO_Biological_Process_2023	1	2.147
Positive Regulation Of Insulin Receptor Signaling Pathway (GO:0046628)	7.774E-03	IGF2	735.017	GO_Biological_Process_2023	1	2.109
Negative Regulation By Host Of Viral Process (GO:0044793)	7.774E-03	PSMC3	735.017	GO_Biological_Process_2023	1	2.109
Negative Regulation By Host Of Viral Transcription (GO:0043922)	7.774E-03	PSMC3	735.017	GO_Biological_Process_2023	1	2.109
Positive Regulation Of Vascular Endothelial Cell Proliferation (GO:1905564)	7.774E-03	IGF2	735.017	GO_Biological_Process_2023	1	2.109
pre-miRNA Processing (GO:0031054)	7.774E-03	PRKRA	735.017	GO_Biological_Process_2023	1	2.109
Positive Regulation Of Glycogen Biosynthetic Process (GO:0045725)	8.420E-03	IGF2	662.671	GO_Biological_Process_2023	1	2.075
Outer Mitochondrial Membrane Organization (GO:0007008)	8.420E-03	HSPA4	662.671	GO_Biological_Process_2023	1	2.075
Protein Insertion Into Mitochondrial Outer Membrane (GO:0045040)	8.420E-03	HSPA4	662.671	GO_Biological_Process_2023	1	2.075
Positive Regulation Of Cellular Response To Insulin Stimulus (GO:1900078)	8.420E-03	IGF2	662.671	GO_Biological_Process_2023	1	2.075
Positive Regulation Of Glycogen Metabolic Process (GO:0070875)	9.064E-03	IGF2	602.215	GO_Biological_Process_2023	1	2.043
Striated Muscle Cell Development (GO:0055002)	9.064E-03	MYOZ1	602.215	GO_Biological_Process_2023	1	2.043

Pathway	pvalue	GeneCount	CombinedScore	Source	GeneCountCount	log10_pvalue
Mammary Gland Epithelium Development (GO:0061180)	9.064E-03	WNT3	602.215	GO_Biological_Process_2023	1	2.043
Receptor Recycling (GO:0001881)	1.100E-02	PLEKHA3	469.124	GO_Biological_Process_2023	1	1.959
Positive Regulation Of Neutrophil Chemotaxis (GO:0090023)	1.164E-02	THBS4	435.941	GO_Biological_Process_2023	1	1.934
Regulation Of Vascular Endothelial Cell Proliferation (GO:1905562)	1.164E-02	IGF2	435.941	GO_Biological_Process_2023	1	1.934
Positive Regulation Of Activated T Cell Proliferation (GO:0042104)	1.228E-02	IGF2	406.730	GO_Biological_Process_2023	1	1.911
Regulation Of Tissue Remodeling (GO:0034103)	1.228E-02	THBS4	406.730	GO_Biological_Process_2023	1	1.911
Receptor Metabolic Process (GO:0043112)	1.293E-02	PLEKHA3	380.838	GO_Biological_Process_2023	1	1.889
Chaperone-Mediated Protein Complex Assembly (GO:0051131)	1.357E-02	HSPA4	357.744	GO_Biological_Process_2023	1	1.867
Positive Regulation Of Granulocyte Chemotaxis (GO:0071624)	1.357E-02	THBS4	357.744	GO_Biological_Process_2023	1	1.867
Mammary Gland Development (GO:0030879)	1.421E-02	WNT3	337.029	GO_Biological_Process_2023	1	1.847
Placenta Development (GO:0001890)	1.421E-02	IGF2	337.029	GO_Biological_Process_2023	1	1.847
Positive Regulation Of Neutrophil Migration (GO:1902624)	1.485E-02	THBS4	318.355	GO_Biological_Process_2023	1	1.828
MicroRNA (miRNA) Biogenesis R-HSA-203927	1.549E-02	PRKRA	301.441	Reactome_2022	1	1.810
Regulation Of Muscle Cell Differentiation (GO:0051147)	1.549E-02	IGF2	301.441	GO_Biological_Process_2023	1	1.810
Regulation Of Glycogen Biosynthetic Process (GO:0005979)	1.613E-02	IGF2	286.058	GO_Biological_Process_2023	1	1.792
Regulation Of Neutrophil Chemotaxis (GO:0090022)	1.613E-02	THBS4	286.058	GO_Biological_Process_2023	1	1.792
WNT Ligand Biogenesis And Trafficking R-HSA-3238698	1.677E-02	WNT3	272.012	Reactome_2022	1	1.775
Positive Regulation Of Nuclear Division (GO:0051785)	1.677E-02	IGF2	272.012	GO_Biological_Process_2023	1	1.775
In Utero Embryonic Development (GO:0001701)	1.741E-02	IGF2	259.141	GO_Biological_Process_2023	1	1.759
Peptidyl-Proline Modification (GO:0018208)	1.741E-02	FKBP7	259.141	GO_Biological_Process_2023	1	1.759
Protein Insertion Into Mitochondrial Membrane (GO:0051204)	1.805E-02	HSPA4	247.309	GO_Biological_Process_2023	1	1.743
Negative Regulation Of Wound Healing (GO:0061045)	1.805E-02	MYOZ1	247.309	GO_Biological_Process_2023	1	1.743
Sarcomere Organization (GO:0045214)	1.869E-02	MYOZ1	236.398	GO_Biological_Process_2023	1	1.728
Negative Regulation Of Developmental Growth (GO:0048640)	1.997E-02	MYOZ1	216.952	GO_Biological_Process_2023	1	1.700
Positive Regulation Of Proteolysis Involved In Protein Catabolic Process (GO:1903052)	1.997E-02	PSMC3	216.952	GO_Biological_Process_2023	1	1.700
miRNA Processing (GO:0035196)	2.061E-02	PRKRA	208.256	GO_Biological_Process_2023	1	1.686
Regulation Of Activated T Cell Proliferation (GO:0046006)	2.125E-02	IGF2	200.154	GO_Biological_Process_2023	1	1.673
Regulation Of calcineurin-NFAT Signaling Cascade (GO:0070884)	2.188E-02	MYOZ1	192.590	GO_Biological_Process_2023	1	1.660
Regulatory ncRNA Processing (GO:0070918)	2.252E-02	PRKRA	185.513	GO_Biological_Process_2023	1	1.647
Positive Regulation Of Mitotic Nuclear Division (GO:0045840)	2.252E-02	IGF2	185.513	GO_Biological_Process_2023	1	1.647
Skeletal Muscle Organ Development (GO:0060538)	2.252E-02	MYOZ1	185.513	GO_Biological_Process_2023	1	1.647
Muscle Tissue Development (GO:0060537)	2.379E-02	MYOZ1	172.651	GO_Biological_Process_2023	1	1.624
Positive Regulation Of Catalytic Activity (GO:0043085)	2.570E-02	IGF2	156.061	GO_Biological_Process_2023	1	1.590

Pathway	pvalue	GeneCount	CombinedScore	Source	GeneCountCount	log10_pvalue
Cellular Response To Retinoic Acid (GO:0071300)	2.633E-02	WNT3	151.138	GO_Biological_Process_2023	1	1.580
Skeletal Muscle Tissue Development (GO:0007519)	2.697E-02	MYOZ1	146.480	GO_Biological_Process_2023	1	1.569
Response To Unfolded Protein (GO:0006986)	2.823E-02	HSPA4	137.878	GO_Biological_Process_2023	1	1.549
Regulation Of Insulin Receptor Signaling Pathway (GO:0046626)	2.823E-02	IGF2	137.878	GO_Biological_Process_2023	1	1.549
Proteasome	2.950E-02	PSMC3	130.117	Kegg_2021_Human	1	1.530
Myofibril Assembly (GO:0030239)	2.950E-02	MYOZ1	130.117	GO_Biological_Process_2023	1	1.530
Regulation Of Nervous System Development (GO:0051960)	2.950E-02	WNT3	130.117	GO_Biological_Process_2023	1	1.530
Insulin Receptor Signaling Pathway (GO:0008286)	3.013E-02	IGF2	126.516	GO_Biological_Process_2023	1	1.521
Cross-presentation Of Soluble Exogenous Antigens (Endosomes) R-HSA-1236978	3.076E-02	PSMC3	123.084	Reactome_2022	1	1.512
Regulation Of Activated PAK-2p34 By Proteasome Mediated Degradation R-HSA-211733	3.140E-02	PSMC3	119.811	Reactome_2022	1	1.503
Malaria	3.203E-02	THBS4	116.685	Kegg_2021_Human	1	1.494
Regulation Of Ornithine Decarboxylase (ODC) R-HSA-350562	3.203E-02	PSMC3	116.685	Reactome_2022	1	1.494
Synthesis Of PIPs At Plasma Membrane R-HSA-1660499	3.266E-02	PLEKHA3	113.697	Reactome_2022	1	1.486
GSK3B And BTRC:CUL1-mediated-degradation Of NFE2L2 R-HSA-9762114	3.266E-02	PSMC3	113.697	Reactome_2022	1	1.486
Autodegradation Of E3 Ubiquitin Ligase COP1 R-HSA-349425	3.266E-02	PSMC3	113.697	Reactome_2022	1	1.486
IRS-related Events Triggered By IGF1R R-HSA-2428928	3.266E-02	IGF2	113.697	Reactome_2022	1	1.486
Ubiquitin Mediated Degradation Of Phosphorylated Cdc25A R-HSA-69601	3.266E-02	PSMC3	113.697	Reactome_2022	1	1.486
Ubiquitin-dependent Degradation Of Cyclin D R-HSA-75815	3.266E-02	PSMC3	113.697	Reactome_2022	1	1.486
Vpu Mediated Degradation Of CD4 R-HSA-180534	3.266E-02	PSMC3	113.697	Reactome_2022	1	1.486
Regulation Of Apoptosis R-HSA-169911	3.329E-02	PSMC3	110.840	Reactome_2022	1	1.478
IGF1R Signaling Cascade R-HSA-2428924	3.329E-02	IGF2	110.840	Reactome_2022	1	1.478
Signaling By PDGF R-HSA-186797	3.329E-02	THBS4	110.840	Reactome_2022	1	1.478
Regulation Of Cell Development (GO:0060284)	3.329E-02	WNT3	110.840	GO_Biological_Process_2023	1	1.478
Negative Regulation Of NOTCH4 Signaling R-HSA-9604323	3.392E-02	PSMC3	108.104	Reactome_2022	1	1.470
Vif-mediated Degradation Of APOBEC3G R-HSA-180585	3.392E-02	PSMC3	108.104	Reactome_2022	1	1.470
Signaling By Type 1 Insulin-like Growth Factor 1 Receptor (IGF1R) R-HSA-2404192	3.392E-02	IGF2	108.104	Reactome_2022	1	1.470
Regulation Of Proteasomal Protein Catabolic Process (GO:0061136)	3.392E-02	PSMC3	108.104	GO_Biological_Process_2023	1	1.470
Endosome Organization (GO:0007032)	3.392E-02	PLEKHA3	108.104	GO_Biological_Process_2023	1	1.470
AUF1 (hnRNP D0) Binds And Destabilizes mRNA R-HSA-450408	3.455E-02	PSMC3	105.482	Reactome_2022	1	1.462
Regulation Of RUNX3 Expression And Activity R-HSA-8941858	3.455E-02	PSMC3	105.482	Reactome_2022	1	1.462
SCF-beta-TrCP Mediated Degradation Of Emi1 R-HSA-174113	3.455E-02	PSMC3	105.482	Reactome_2022	1	1.462
Degradation Of AXIN R-HSA-4641257	3.455E-02	PSMC3	105.482	Reactome_2022	1	1.462
FBXL7 Down-Regulates AURKA During Mitotic Entry And In Early Mitosis R-HSA-8854050	3.455E-02	PSMC3	105.482	Reactome_2022	1	1.462
Degradation Of DVL R-HSA-4641258	3.518E-02	PSMC3	102.968	Reactome_2022	1	1.454
Hh Mutants Are Degraded By ERAD R-HSA-5362768	3.518E-02	PSMC3	102.968	Reactome_2022	1	1.454
Stabilization Of P53 R-HSA-69541	3.581E-02	PSMC3	100.556	Reactome_2022	1	1.446
Regulation Of Mitotic Nuclear Division (GO:0007088)	3.581E-02	IGF2	100.556	GO_Biological_Process_2023	1	1.446

Pathway	pvalue	GeneCount	CombinedScore	Source	GeneCountCount	log10_pvalue
Metabolism Of Polyamines R-HSA-351202	3.706E-02	PSMC3	96.013	Reactome_2022	1	1.431
Hh Mutants Abrogate Ligand Secretion R-HSA-5387390	3.706E-02	PSMC3	96.013	Reactome_2022	1	1.431
NIK To Noncanonical NF-kB Signaling R-HSA-5676590	3.706E-02	PSMC3	96.013	Reactome_2022	1	1.431
GLI3 Is Processed To GLI3R By Proteasome R-HSA-5610785	3.769E-02	PSMC3	93.872	Reactome_2022	1	1.424
SCF(Skp2)-mediated Degradation Of P27/P21 R-HSA-187577	3.769E-02	PSMC3	93.872	Reactome_2022	1	1.424
Role Of GTSE1 In G2/M Progression After G2 Checkpoint R-HSA-8852276	3.769E-02	PSMC3	93.872	Reactome_2022	1	1.424
Degradation Of GLI1 By Proteasome R-HSA-5610780	3.769E-02	PSMC3	93.872	Reactome_2022	1	1.424
Degradation Of GLI2 By Proteasome R-HSA-5610783	3.769E-02	PSMC3	93.872	Reactome_2022	1	1.424
Chordate Embryonic Development (GO:0043009)	3.769E-02	IGF2	93.872	GO_Biological_Process_2023	1	1.424
Protein Targeting To Mitochondrion (GO:0006626)	3.769E-02	HSPA4	93.872	GO_Biological_Process_2023	1	1.424
Defective CFTR Causes Cystic Fibrosis R-HSA-5678895	3.832E-02	PSMC3	91.811	Reactome_2022	1	1.417
Response To Retinoic Acid (GO:0032526)	3.832E-02	WNT3	91.811	GO_Biological_Process_2023	1	1.417

Supplementary Table 3C: Left venticular-end systolic volume (LVESV)						
**Pathway**	**pvalue**	**GeneCount**	**CombinedScore**	**Source**	**GeneCountCount**	**log10_pvalue**
Modulation By Host Of Symbiont Process (GO:0051851)	5.341E-03	SMARCB1;RAB5A	104.164	GO_Biological_Process_2023	2	2.272
Glomerular Epithelial Cell Differentiation (GO:0072311)	1.293E-02	PROM1	425.073	GO_Biological_Process_2023	1	1.888
Renal Filtration Cell Differentiation (GO:0061318)	1.293E-02	PROM1	425.073	GO_Biological_Process_2023	1	1.888
Insulin Processing (GO:0030070)	1.293E-02	YIPF5	425.073	GO_Biological_Process_2023	1	1.888
interleukin-17A-mediated Signaling Pathway (GO:0038173)	1.293E-02	MAP3K7	425.073	GO_Biological_Process_2023	1	1.888
Nephron Tubule Development (GO:0072080)	1.293E-02	MTSS1	425.073	GO_Biological_Process_2023	1	1.888
Signal Complex Assembly (GO:0007172)	1.293E-02	PXN	425.073	GO_Biological_Process_2023	1	1.888
Positive Regulation Of Epithelial Cell Differentiation Involved In Kidney Development (GO:2000698)	1.293E-02	PROM1	425.073	GO_Biological_Process_2023	1	1.888
Negative Regulation Of Chemokine-Mediated Signaling Pathway (GO:0070100)	1.293E-02	SH2B3	425.073	GO_Biological_Process_2023	1	1.888
Nucleotide-Binding Domain, Leucine Rich Repeat Containing Receptor Signaling Pathway (GO:0035872)	1.293E-02	MAP3K7	425.073	GO_Biological_Process_2023	1	1.888
Epithelial Cell Differentiation Involved In Kidney Development (GO:0035850)	1.550E-02	MTSS1	325.882	GO_Biological_Process_2023	1	1.810
Regulation Of Respiratory Gaseous Exchange (GO:0043576)	1.550E-02	NMB	325.882	GO_Biological_Process_2023	1	1.810
Transepithelial Chloride Transport (GO:0030321)	1.550E-02	CLCNKA	325.882	GO_Biological_Process_2023	1	1.810
Vesicle Fusion With Golgi Apparatus (GO:0048280)	1.550E-02	YIPF5	325.882	GO_Biological_Process_2023	1	1.810
Adherens Junction Maintenance (GO:0034334)	1.550E-02	MTSS1	325.882	GO_Biological_Process_2023	1	1.810
Vitamin C (Ascorbate) Metabolism R-HSA-196836	1.806E-02	SLC23A1	261.591	Reactome_2022	1	1.743
Regulation Of Chemokine-Mediated Signaling Pathway (GO:0070099)	1.806E-02	SH2B3	261.591	GO_Biological_Process_2023	1	1.743
Regulation Of Microvillus Organization (GO:0032530)	1.806E-02	TWF2	261.591	GO_Biological_Process_2023	1	1.743
siRNA Processing (GO:0030422)	1.806E-02	PRKRA	261.591	GO_Biological_Process_2023	1	1.743
p38MAPK Cascade (GO:0038066)	2.062E-02	MAP3K7	216.822	GO_Biological_Process_2023	1	1.686
Negative Regulation Of Platelet Aggregation (GO:0090331)	2.062E-02	SH2B3	216.822	GO_Biological_Process_2023	1	1.686
Anoikis (GO:0043276)	2.062E-02	MAP3K7	216.822	GO_Biological_Process_2023	1	1.686
Integrin Activation (GO:0033622)	2.062E-02	MZB1	216.822	GO_Biological_Process_2023	1	1.686
Regulation Of Nephron Tubule Epithelial Cell Differentiation (GO:0072182)	2.062E-02	PROM1	216.822	GO_Biological_Process_2023	1	1.686
Sequestering Of Actin Monomers (GO:0042989)	2.062E-02	TWF2	216.822	GO_Biological_Process_2023	1	1.686
Small Interfering RNA (siRNA) Biogenesis R-HSA-426486	2.316E-02	PRKRA	184.016	Reactome_2022	1	1.635
Prevention Of Phagosomal-Lysosomal Fusion R-HSA-9636383	2.316E-02	RAB5A	184.016	Reactome_2022	1	1.635
Positive Regulation Of Hippo Signaling (GO:0035332)	2.316E-02	TIAL1	184.016	GO_Biological_Process_2023	1	1.635
Peptidyl-Amino Acid Modification (GO:0018193)	2.316E-02	FKBP7	184.016	GO_Biological_Process_2023	1	1.635
I-kappaB Phosphorylation (GO:0007252)	2.316E-02	MAP3K7	184.016	GO_Biological_Process_2023	1	1.635
Positive Regulation Of miRNA-mediated Gene Silencing (GO:2000637)	2.316E-02	TIAL1	184.016	GO_Biological_Process_2023	1	1.635

Pathway	pvalue	GeneCount	CombinedScore	Source	GeneCountCount	log10_pvalue
Regulation Of ER To Golgi Vesicle-Mediated Transport (GO:0060628)	2.316E-02	YIPF5	184.016	GO_Biological_Process_2023	1	1.635
Insulin Metabolic Process (GO:1901142)	2.316E-02	YIPF5	184.016	GO_Biological_Process_2023	1	1.635
Negative Regulation Of Tyrosine Phosphorylation Of STAT Protein (GO:0042532)	2.316E-02	SH2B3	184.016	GO_Biological_Process_2023	1	1.635
Positive Regulation Of Transcription Of Nucleolar Large rRNA By RNA Polymerase I (GO:1901838)	2.316E-02	SMARCB1	184.016	GO_Biological_Process_2023	1	1.635
IRAK2 Mediated Activation Of TAK1 Complex R-HSA-937042	2.570E-02	MAP3K7	159.040	Reactome_2022	1	1.590
Negative Regulation Of Homotypic Cell-Cell Adhesion (GO:0034111)	2.570E-02	SH2B3	159.040	GO_Biological_Process_2023	1	1.590
Megakaryocyte Development (GO:0035855)	2.570E-02	SH2B3	159.040	GO_Biological_Process_2023	1	1.590
Actin Filament Depolymerization (GO:0030042)	2.570E-02	TWF2	159.040	GO_Biological_Process_2023	1	1.590
Positive Regulation Of Post-Transcriptional Gene Silencing By RNA (GO:1900370)	2.570E-02	TIAL1	159.040	GO_Biological_Process_2023	1	1.590
Negative Regulation Of Response To Cytokine Stimulus (GO:0060761)	2.570E-02	SH2B3	159.040	GO_Biological_Process_2023	1	1.590
Regulation Of Long-Term Neuronal Synaptic Plasticity (GO:0048169)	2.570E-02	RAB5A	159.040	GO_Biological_Process_2023	1	1.590
TICAM1,TRAF6-dependent Induction Of TAK1 Complex R-HSA-9014325	2.824E-02	MAP3K7	139.452	Reactome_2022	1	1.549
Alpha-protein Kinase 1 Signaling Pathway R-HSA-9645460	2.824E-02	MAP3K7	139.452	Reactome_2022	1	1.549
Nucleotide-Binding Oligomerization Domain Containing Signaling Pathway (GO:0070423)	2.824E-02	MAP3K7	139.452	GO_Biological_Process_2023	1	1.549
Erythrocyte Development (GO:0048821)	2.824E-02	SH2B3	139.452	GO_Biological_Process_2023	1	1.549
Regulation Of Endosome Size (GO:0051036)	2.824E-02	RAB5A	139.452	GO_Biological_Process_2023	1	1.549
Positive Regulation Of Lipid Transport (GO:0032370)	2.824E-02	NMB	139.452	GO_Biological_Process_2023	1	1.549
Cellular Response To Fluid Shear Stress (GO:0071498)	2.824E-02	MTSS1	139.452	GO_Biological_Process_2023	1	1.549
Cellular Response To Growth Hormone Stimulus (GO:0071378)	2.824E-02	PXN	139.452	GO_Biological_Process_2023	1	1.549
Podocyte Differentiation (GO:0072112)	2.824E-02	PROM1	139.452	GO_Biological_Process_2023	1	1.549
Eye Photoreceptor Cell Differentiation (GO:0001754)	3.077E-02	PROM1	123.721	GO_Biological_Process_2023	1	1.512
Cell-Cell Junction Maintenance (GO:0045217)	3.077E-02	MTSS1	123.721	GO_Biological_Process_2023	1	1.512
MyD88-dependent Toll-Like Receptor Signaling Pathway (GO:0002755)	3.077E-02	MAP3K7	123.721	GO_Biological_Process_2023	1	1.512
pre-miRNA Processing (GO:0031054)	3.077E-02	PRKRA	123.721	GO_Biological_Process_2023	1	1.512
Suppression Of Phagosomal Maturation R-HSA-9637687	3.329E-02	RAB5A	110.840	Reactome_2022	1	1.478
Regulation Of Transcription Of Nucleolar Large rRNA By RNA Polymerase I (GO:1901836)	3.329E-02	SMARCB1	110.840	GO_Biological_Process_2023	1	1.478
Regulation Of Lamellipodium Organization (GO:1902743)	3.329E-02	TWF2	110.840	GO_Biological_Process_2023	1	1.478
Response To Chemokine (GO:1990868)	3.329E-02	SH2B3	110.840	GO_Biological_Process_2023	1	1.478
Embryonic Hemopoiesis (GO:0035162)	3.329E-02	SH2B3	110.840	GO_Biological_Process_2023	1	1.478
Cardiac Cell Development (GO:0055006)	3.580E-02	ALPK3	100.118	GO_Biological_Process_2023	1	1.446
Regulation Of Vesicle Size (GO:0097494)	3.580E-02	RAB5A	100.118	GO_Biological_Process_2023	1	1.446
Negative Regulation Of Receptor Signaling Pathway Via STAT (GO:1904893)	3.580E-02	SH2B3	100.118	GO_Biological_Process_2023	1	1.446
IRAK2 Mediated Activation Of TAK1 Complex Upon TLR7/8 Or 9 Stimulation R-HSA-975163	3.831E-02	MAP3K7	91.071	Reactome_2022	1	1.417

Pathway	pvalue	GeneCount	CombinedScore	Source	GeneCountCount	log10_pvalue
Negative Regulation Of FLT3 R-HSA-9706369	3.831E-02	SH2B3	91.071	Reactome_2022	1	1.417
Stem Cell Development (GO:0048864)	3.831E-02	TAPT1	91.071	GO_Biological_Process_2023	1	1.417
Regulation Of Hormone Secretion (GO:0046883)	3.831E-02	NMB	91.071	GO_Biological_Process_2023	1	1.417
Negative Regulation Of Platelet Activation (GO:0010544)	3.831E-02	SH2B3	91.071	GO_Biological_Process_2023	1	1.417
Activation Of NF-kappaB-inducing Kinase Activity (GO:0007250)	3.831E-02	MAP3K7	91.071	GO_Biological_Process_2023	1	1.417
Regulation Of miRNA-mediated Gene Silencing (GO:0060964)	3.831E-02	TIAL1	91.071	GO_Biological_Process_2023	1	1.417

Supplementary Table 3D: Left venticular-end diastolic volume (LVEDV)						
Pathway	pvalue	GeneCount	CombinedScore	Source	GeneCountCount	log10_pvalue
Embryonic Hemopoiesis (GO:0035162)	2.427E-04	SH2B3;ZFPM1	888.159	GO_Biological_Process_2023	2	3.615
Megakaryocyte Differentiation (GO:0030219)	4.212E-04	SH2B3;ZFPM1	608.048	GO_Biological_Process_2023	2	3.376
Cardiac Atrium Morphogenesis (GO:0003209)	4.733E-04	ZFPM1;ENG	561.461	GO_Biological_Process_2023	2	3.325
Negative Regulation Of Binding (GO:0051100)	4.947E-04	PLN;SYMPK;ZFPM1	165.748	GO_Biological_Process_2023	3	3.306
Focal Adhesion Assembly (GO:0048041)	6.474E-04	TRIP6;TESK2	453.394	GO_Biological_Process_2023	2	3.189
Cell-Substrate Junction Assembly (GO:0007044)	1.156E-03	TRIP6;TESK2	305.053	GO_Biological_Process_2023	2	2.937
Negative Regulation Of Myeloid Leukocyte Differentiation (GO:0002762)	1.328E-03	TCTA;ZFPM1	277.447	GO_Biological_Process_2023	2	2.877
Cardiac Muscle Tissue Morphogenesis (GO:0055008)	1.417E-03	ZFPM1;ENG	265.216	GO_Biological_Process_2023	2	2.848
miRNA Processing (GO:0035196)	1.510E-03	PRKRA;SRRT	253.884	GO_Biological_Process_2023	2	2.821
Negative Regulation Of Protein Kinase B Signaling (GO:0051898)	1.910E-03	DAG1;SH2B3	215.880	GO_Biological_Process_2023	2	2.719
Erythrocyte Differentiation (GO:0030218)	3.513E-03	SH2B3;ZFPM1	140.874	GO_Biological_Process_2023	2	2.454
Blood Circulation (GO:0008015)	3.655E-03	PLN;GPX1	136.963	GO_Biological_Process_2023	2	2.437
Heart Morphogenesis (GO:0003007)	5.053E-03	ZFPM1;ENG	108.630	GO_Biological_Process_2023	2	2.296
Regulation Of Relaxation Of Cardiac Muscle (GO:1901897)	8.968E-03	PLN	672.088	GO_Biological_Process_2023	1	2.047
PERK-mediated Unfolded Protein Response (GO:0036499)	8.968E-03	QRICH1	672.088	GO_Biological_Process_2023	1	2.047
Venous Blood Vessel Morphogenesis (GO:0048845)	8.968E-03	ENG	672.088	GO_Biological_Process_2023	1	2.047
Multinuclear Osteoclast Differentiation (GO:0072674)	8.968E-03	TCTA	672.088	GO_Biological_Process_2023	1	2.047
Negative Regulation Of Calcium Ion Import (GO:0090281)	8.968E-03	PLN	672.088	GO_Biological_Process_2023	1	2.047
Positive Regulation Of Vascular Associated Smooth Muscle Cell Differentiation (GO:1905065)	8.968E-03	ENG	672.088	GO_Biological_Process_2023	1	2.047
Negative Regulation Of Chemokine-Mediated Signaling Pathway (GO:0070100)	8.968E-03	SH2B3	672.088	GO_Biological_Process_2023	1	2.047
Synthesis Of 15-Eicosatetraenoic Acid Derivatives R-HSA-2142770	1.075E-02	GPX1	516.949	Reactome_2022	1	1.968
Negative Regulation Of Heart Rate (GO:0010459)	1.075E-02	PLN	516.949	GO_Biological_Process_2023	1	1.968
Regulation Of Relaxation Of Muscle (GO:1901077)	1.075E-02	PLN	516.949	GO_Biological_Process_2023	1	1.968
Vascular Associated Smooth Muscle Cell Development (GO:0097084)	1.075E-02	ENG	516.949	GO_Biological_Process_2023	1	1.968
Dorsal Aorta Development (GO:0035907)	1.075E-02	ENG	516.949	GO_Biological_Process_2023	1	1.968
Dorsal Aorta Morphogenesis (GO:0035912)	1.075E-02	ENG	516.949	GO_Biological_Process_2023	1	1.968
Atrioventricular Valve Formation (GO:0003190)	1.075E-02	ZFPM1	516.949	GO_Biological_Process_2023	1	1.968
Regulation Of Cardiac Muscle Cell Membrane Potential (GO:0086036)	1.075E-02	PLN	516.949	GO_Biological_Process_2023	1	1.968
Synthesis Of 12-Eicosatetraenoic Acid Derivatives R-HSA-2142712	1.253E-02	GPX1	416.202	Reactome_2022	1	1.902
Regulation Of Chemokine-Mediated Signaling Pathway (GO:0070099)	1.253E-02	SH2B3	416.202	GO_Biological_Process_2023	1	1.902
Cardiac Atrium Development (GO:0003230)	1.253E-02	ENG	416.202	GO_Biological_Process_2023	1	1.902

Pathway	pvalue	GeneCount	CombinedScore	Source	GeneCountCount	log10_pvalue
Negative Regulation Of Intracellular Transport (GO:0032387)	1.253E-02	PLN	416.202	GO_Biological_Process_2023	1	1.902
Regulation Of Epithelial To Mesenchymal Transition Involved In Endocardial Cushion Formation (GO:1905005)	1.253E-02	ENG	416.202	GO_Biological_Process_2023	1	1.902
Venous Blood Vessel Development (GO:0060841)	1.253E-02	ENG	416.202	GO_Biological_Process_2023	1	1.902
Ventricular Trabecula Myocardium Morphogenesis (GO:0003222)	1.253E-02	ENG	416.202	GO_Biological_Process_2023	1	1.902
UV Protection (GO:0009650)	1.253E-02	GPX1	416.202	GO_Biological_Process_2023	1	1.902
Regulation Of ATPase-coupled Calcium Transmembrane Transporter Activity (GO:1901894)	1.253E-02	PLN	416.202	GO_Biological_Process_2023	1	1.902
Regulation Of Mammary Gland Epithelial Cell Proliferation (GO:0033599)	1.253E-02	GPX1	416.202	GO_Biological_Process_2023	1	1.902
Positive Regulation Of Cardiac Epithelial To Mesenchymal Transition (GO:0062043)	1.253E-02	ENG	416.202	GO_Biological_Process_2023	1	1.902
Regulation Of Microvillus Organization (GO:0032530)	1.253E-02	TWF2	416.202	GO_Biological_Process_2023	1	1.902
siRNA Processing (GO:0030422)	1.253E-02	PRKRA	416.202	GO_Biological_Process_2023	1	1.902
Lipoxygenase Pathway (GO:0019372)	1.253E-02	GPX1	416.202	GO_Biological_Process_2023	1	1.902
Epithelial To Mesenchymal Transition Involved In Endocardial Cushion Formation (GO:0003198)	1.431E-02	ENG	345.921	GO_Biological_Process_2023	1	1.844
Vascular Associated Smooth Muscle Cell Differentiation (GO:0035886)	1.431E-02	ENG	345.921	GO_Biological_Process_2023	1	1.844
Negative Regulation Of Platelet Aggregation (GO:0090331)	1.431E-02	SH2B3	345.921	GO_Biological_Process_2023	1	1.844
Sequestering Of Actin Monomers (GO:0042989)	1.431E-02	TWF2	345.921	GO_Biological_Process_2023	1	1.844
Positive Regulation Of Extracellular Matrix Assembly (GO:1901203)	1.431E-02	DAG1	345.921	GO_Biological_Process_2023	1	1.844
Small Interfering RNA (siRNA) Biogenesis R-HSA-426486	1.609E-02	PRKRA	294.333	Reactome_2022	1	1.794
Synthesis Of 5-Eicosatetraenoic Acids R-HSA-2142688	1.609E-02	GPX1	294.333	Reactome_2022	1	1.794
Positive Regulation Of Hippo Signaling (GO:0035332)	1.609E-02	TIAL1	294.333	GO_Biological_Process_2023	1	1.794
Peptidyl-Amino Acid Modification (GO:0018193)	1.609E-02	FKBP7	294.333	GO_Biological_Process_2023	1	1.794
Microtubule Anchoring (GO:0034453)	1.609E-02	DAG1	294.333	GO_Biological_Process_2023	1	1.794
Positive Regulation Of miRNA-mediated Gene Silencing (GO:2000637)	1.609E-02	TIAL1	294.333	GO_Biological_Process_2023	1	1.794
Mitral Valve Morphogenesis (GO:0003183)	1.609E-02	ZFPM1	294.333	GO_Biological_Process_2023	1	1.794
Negative Regulation Of Protein Autophosphorylation (GO:0031953)	1.609E-02	ENG	294.333	GO_Biological_Process_2023	1	1.794
Regulation Of Actin Filament-Based Movement (GO:1903115)	1.609E-02	PLN	294.333	GO_Biological_Process_2023	1	1.794
Negative Regulation Of Tyrosine Phosphorylation Of STAT Protein (GO:0042532)	1.609E-02	SH2B3	294.333	GO_Biological_Process_2023	1	1.794
Smooth Muscle Tissue Development (GO:0048745)	1.609E-02	ENG	294.333	GO_Biological_Process_2023	1	1.794
Neurotransmitter Clearance R-HSA-112311	1.786E-02	ACHE	254.997	Reactome_2022	1	1.748
Negative Regulation Of Homotypic Cell-Cell Adhesion (GO:0034111)	1.786E-02	SH2B3	254.997	GO_Biological_Process_2023	1	1.748
Megakaryocyte Development (GO:0035855)	1.786E-02	SH2B3	254.997	GO_Biological_Process_2023	1	1.748
T-helper Cell Lineage Commitment (GO:0002295)	1.786E-02	ZFPM1	254.997	GO_Biological_Process_2023	1	1.748
Actin Filament Depolymerization (GO:0030042)	1.786E-02	TWF2	254.997	GO_Biological_Process_2023	1	1.748
Positive Regulation Of Post-Transcriptional Gene Silencing By RNA (GO:1900370)	1.786E-02	TIAL1	254.997	GO_Biological_Process_2023	1	1.748

Pathway	pvalue	GeneCount	CombinedScore	Source	GeneCountCount	log10_pvalue
Negative Regulation Of Response To Cytokine Stimulus (GO:0060761)	1.786E-02	SH2B3	254.997	GO_Biological_Process_2023	1	1.748
Insulin-Like Growth Factor Receptor Signaling Pathway (GO:0048009)	1.786E-02	GIGYF1	254.997	GO_Biological_Process_2023	1	1.748
Atrial Septum Morphogenesis (GO:0060413)	1.786E-02	ZFPM1	254.997	GO_Biological_Process_2023	1	1.748
Regulation Of Cardiac Muscle Cell Apoptotic Process (GO:0010665)	1.786E-02	ENG	254.997	GO_Biological_Process_2023	1	1.748
Erythrocyte Development (GO:0048821)	1.963E-02	SH2B3	224.102	GO_Biological_Process_2023	1	1.707
Negative Regulation Of Heart Contraction (GO:0045822)	1.963E-02	PLN	224.102	GO_Biological_Process_2023	1	1.707
Relaxation Of Cardiac Muscle (GO:0055119)	2.139E-02	PLN	199.255	GO_Biological_Process_2023	1	1.670
Atrial Cardiac Muscle Tissue Development (GO:0003228)	2.139E-02	ENG	199.255	GO_Biological_Process_2023	1	1.670
pre-miRNA Processing (GO:0031054)	2.139E-02	PRKRA	199.255	GO_Biological_Process_2023	1	1.670
Primary miRNA Processing (GO:0031053)	2.139E-02	SRRT	199.255	GO_Biological_Process_2023	1	1.670
TNFR1-induced Proapoptotic Signaling R-HSA-5357786	2.316E-02	USP4	178.880	Reactome_2022	1	1.635
Spliceosomal tri-snRNP Complex Assembly (GO:0000244)	2.316E-02	USP4	178.880	GO_Biological_Process_2023	1	1.635
Regulation Of Gastrulation (GO:0010470)	2.316E-02	DAG1	178.880	GO_Biological_Process_2023	1	1.635
Relaxation Of Muscle (GO:0090075)	2.316E-02	PLN	178.880	GO_Biological_Process_2023	1	1.635
Regulation Of Lamellipodium Organization (GO:1902743)	2.316E-02	TWF2	178.880	GO_Biological_Process_2023	1	1.635
Response To Chemokine (GO:1990868)	2.316E-02	SH2B3	178.880	GO_Biological_Process_2023	1	1.635
Atrial Septum Development (GO:0003283)	2.316E-02	ZFPM1	178.880	GO_Biological_Process_2023	1	1.635
Atrioventricular Valve Development (GO:0003171)	2.316E-02	ZFPM1	178.880	GO_Biological_Process_2023	1	1.635
Branching Involved In Blood Vessel Morphogenesis (GO:0001569)	2.316E-02	ENG	178.880	GO_Biological_Process_2023	1	1.635
Negative Regulation Of Receptor Signaling Pathway Via STAT (GO:1904893)	2.492E-02	SH2B3	161.900	GO_Biological_Process_2023	1	1.604
Negative Regulation Of FLT3 R-HSA-9706369	2.667E-02	SH2B3	147.555	Reactome_2022	1	1.574
Negative Regulation Of Platelet Activation (GO:0010544)	2.667E-02	SH2B3	147.555	GO_Biological_Process_2023	1	1.574
Regulation Of miRNA-mediated Gene Silencing (GO:0060964)	2.667E-02	TIAL1	147.555	GO_Biological_Process_2023	1	1.574
Regulation Of KIT Signaling R-HSA-1433559	2.842E-02	SH2B3	135.292	Reactome_2022	1	1.546
Regulation Of Response To Cytokine Stimulus (GO:0060759)	2.842E-02	SH2B3	135.292	GO_Biological_Process_2023	1	1.546
Heart Process (GO:0003015)	2.842E-02	GPX1	135.292	GO_Biological_Process_2023	1	1.546
Negative Regulation Of Receptor Signaling Pathway Via JAK-STAT (GO:0046426)	2.842E-02	SH2B3	135.292	GO_Biological_Process_2023	1	1.546
Negative Regulation Of Release Of Cytochrome C From Mitochondria (GO:0090201)	2.842E-02	GPX1	135.292	GO_Biological_Process_2023	1	1.546
Negative Regulation Of ATP-dependent Activity (GO:0032780)	2.842E-02	PLN	135.292	GO_Biological_Process_2023	1	1.546
Receptor Recycling (GO:0001881)	3.018E-02	PLEKHA3	124.701	GO_Biological_Process_2023	1	1.520
Muscle Tissue Morphogenesis (GO:0060415)	3.018E-02	ZFPM1	124.701	GO_Biological_Process_2023	1	1.520
Amyloid Precursor Protein Metabolic Process (GO:0042982)	3.018E-02	ACHE	124.701	GO_Biological_Process_2023	1	1.520
Aorta Morphogenesis (GO:0035909)	3.018E-02	ENG	124.701	GO_Biological_Process_2023	1	1.520
Negative Regulation Of Calcium Ion Transport (GO:0051926)	3.018E-02	PLN	124.701	GO_Biological_Process_2023	1	1.520
Mesenchyme Morphogenesis (GO:0072132)	3.192E-02	ENG	115.473	GO_Biological_Process_2023	1	1.496
Negative Regulation Of Monoatomic Ion Transport (GO:0043271)	3.192E-02	PLN	115.473	GO_Biological_Process_2023	1	1.496
T-helper Cell Differentiation (GO:0042093)	3.192E-02	ZFPM1	115.473	GO_Biological_Process_2023	1	1.496

Pathway	pvalue	GeneCount	CombinedScore	Source	GeneCountCount	log10_pvalue
Heart Trabecula Morphogenesis (GO:0061384)	3.192E-02	ENG	115.473	GO_Biological_Process_2023	1	1.496
Actin Filament Capping (GO:0051693)	3.192E-02	TWF2	115.473	GO_Biological_Process_2023	1	1.496
Barbed-End Actin Filament Capping (GO:0051016)	3.192E-02	TWF2	115.473	GO_Biological_Process_2023	1	1.496
Synthesis, Secretion, And Deacylation Of Ghrelin R-HSA-422085	3.367E-02	ACHE	107.368	Reactome_2022	1	1.473
Cardiac Epithelial To Mesenchymal Transition (GO:0060317)	3.367E-02	ENG	107.368	GO_Biological_Process_2023	1	1.473
Cardiac Ventricle Development (GO:0003231)	3.367E-02	ENG	107.368	GO_Biological_Process_2023	1	1.473
Positive Regulation Of Axon Extension (GO:0045773)	3.367E-02	TWF2	107.368	GO_Biological_Process_2023	1	1.473
Negative Regulation Of Calcium Ion Transmembrane Transport (GO:1903170)	3.367E-02	PLN	107.368	GO_Biological_Process_2023	1	1.473
Atrioventricular Valve Morphogenesis (GO:0003181)	3.367E-02	ZFPM1	107.368	GO_Biological_Process_2023	1	1.473
One carbon pool by folate	3.541E-02	AMT	100.200	Kegg_2021_Human	1	1.451
Processing Of Intronless Pre-mRNAs R-HSA-77595	3.541E-02	SYMPK	100.200	Reactome_2022	1	1.451
Regulation Of Gene Expression In Beta Cells R-HSA-210745	3.541E-02	FOXA3	100.200	Reactome_2022	1	1.451
Receptor Metabolic Process (GO:0043112)	3.541E-02	PLEKHA3	100.200	GO_Biological_Process_2023	1	1.451
Hydrogen Peroxide Catabolic Process (GO:0042744)	3.541E-02	GPX1	100.200	GO_Biological_Process_2023	1	1.451
Regulation Of Calcium Ion Import (GO:0090279)	3.541E-02	PLN	100.200	GO_Biological_Process_2023	1	1.451
Negative Regulation Of Osteoclast Differentiation (GO:0045671)	3.715E-02	TCTA	93.820	GO_Biological_Process_2023	1	1.430
Endocardial Cushion Morphogenesis (GO:0003203)	3.715E-02	ENG	93.820	GO_Biological_Process_2023	1	1.430

Supplementary Table 3E: All-cause heart failure in a multi-ancestry population						
**Pathway**	**pvalue**	**GeneCount**	**CombinedScore**	**Source**	**GeneCount**	**log10_pvalue**
Sterol Transport (GO:0015918)	5.340E-07	ABCG8;STARD3;ABCG5;NPC1;CAV1;CD36	367.311	GO_Biological_Process_2023	6	6.273
Cholesterol metabolism	2.840E-06	ABCG8;STARD3;ABCG5;NPC1;APOH;PCSK9;CD36	167.898	Kegg_2021_Human	7	5.546
Negative Regulation Of Epithelial Cell Proliferation (GO:0050680)	2.760E-05	MED1;CDKN2B;TGFB1;CAV2;APOH;CAV1;MTSS1	94.115	GO_Biological_Process_2023	7	4.559
Cholesterol Transport (GO:0030301)	4.130E-05	ABCG8;STARD3;ABCG5;NPC1;CAV1;CD36	108.246	GO_Biological_Process_2023	6	4.384
Intestinal Cholesterol Absorption (GO:0030299)	1.551E-04	ABCG8;NPC1;CD36	349.131	GO_Biological_Process_2023	3	3.809
Fat digestion and absorption	1.951E-04	ABCG8;ABCG5;CD36;AGPAT1;PLPP3	90.059	Kegg_2021_Human	5	3.710
Intestinal Lipid Absorption (GO:0098856)	2.991E-04	ABCG8;NPC1;CD36	242.224	GO_Biological_Process_2023	3	3.524
Response To Steroid Hormone (GO:0048545)	4.494E-04	MED1;TGFB1;CAV1;NR3C1	98.478	GO_Biological_Process_2023	4	3.347
Sarcomere Organization (GO:0045214)	4.494E-04	SYNPO2L;TNNT3;FLNC;MYOZ1	98.478	GO_Biological_Process_2023	4	3.347
Nephron Tubule Development (GO:0072080)	1.530E-03	TFAP2B;MTSS1	342.710	GO_Biological_Process_2023	2	2.815
Cellular Response To Hepatocyte Growth Factor Stimulus (GO:0035729)	2.276E-03	NELL1;BCAR1	241.272	GO_Biological_Process_2023	2	2.643
Response To Hepatocyte Growth Factor (GO:0035728)	2.276E-03	NELL1;BCAR1	241.272	GO_Biological_Process_2023	2	2.643
Dendrite Arborization (GO:0140059)	2.276E-03	NELL1;PHACTR1	241.272	GO_Biological_Process_2023	2	2.643
Positive Regulation Of Necrotic Cell Death (GO:0010940)	2.276E-03	SLC6A6;ATG9B	241.272	GO_Biological_Process_2023	2	2.643
RUNX3 Regulates CDKN1A Transcription R-HSA-8941855	3.160E-03	CDKN1A;TGFB1	182.599	Reactome_2022	2	2.500
Cross-presentation Of Particulate Exogenous Antigens (Phagosomes) R-HSA-1236973	4.179E-03	NCF1;CD36	144.774	Reactome_2022	2	2.379
Regulation Of Necrotic Cell Death (GO:0010939)	4.179E-03	SLC6A6;ATG9B	144.774	GO_Biological_Process_2023	2	2.379
Regulation Of Pattern Recognition Receptor Signaling Pathway (GO:0062207)	4.179E-03	GFI1;CD36	144.774	GO_Biological_Process_2023	2	2.379
Positive Regulation Of Extracellular Matrix Assembly (GO:1901203)	4.179E-03	TGFB1;DAG1	144.774	GO_Biological_Process_2023	2	2.379
Cellular Response To Oxidised Low-Density Lipoprotein Particle Stimulus (GO:0140052)	5.329E-03	MIA3;CD36	118.581	GO_Biological_Process_2023	2	2.273
Positive Regulation Of Cardiocyte Differentiation (GO:1905209)	5.329E-03	TGFB1;NKX2-5	118.581	GO_Biological_Process_2023	2	2.273
Negative Regulation Of Cholesterol Transport (GO:0032375)	6.606E-03	ABCG8;ABCG5	99.494	GO_Biological_Process_2023	2	2.180
Positive Regulation Of Macrophage Differentiation (GO:0045651)	6.606E-03	TGFB1;PRKCA	99.494	GO_Biological_Process_2023	2	2.180
Receptor-Mediated Endocytosis Of Virus By Host Cell (GO:0019065)	6.606E-03	CAV2;CAV1	99.494	GO_Biological_Process_2023	2	2.180

Supplementary Table 3F: All-cause heart failure in a European population						
Pathway	pvalue	GeneCount	CombinedScore	Source	GeneCountCount	log10_pvalue
MHC Class II Protein Complex Assembly (GO:0002399)	1.070E-05	HLA-DQA2;HLA-DQA1;HLA-DQB2;HLA-DRB1	441.810	GO_Biological_Process_2023	4	4.971
Peptide Antigen Assembly With MHC Class II Protein Complex (GO:0002503)	1.070E-05	HLA-DQA2;HLA-DQA1;HLA-DQB2;HLA-DRB1	441.810	GO_Biological_Process_2023	4	4.971
Translocation Of ZAP-70 To Immunological Synapse R-HSA-202430	2.480E-05	HLA-DQA2;HLA-DQA1;HLA-DQB2;HLA-DRB1	314.823	Reactome_2022	4	4.606
Peptide Antigen Assembly With MHC Protein Complex (GO:0002501)	3.160E-05	HLA-DQA2;HLA-DQA1;HLA-DQB2;HLA-DRB1	285.619	GO_Biological_Process_2023	4	4.500
Immunoglobulin Production Involved In Immunoglobulin-Mediated Immune Response (GO:0002381)	3.970E-05	HLA-DQA2;HLA-DQA1;HLA-DQB2;HLA-DRB1	260.694	GO_Biological_Process_2023	4	4.401
Phosphorylation Of CD3 And TCR Zeta Chains R-HSA-202427	4.920E-05	HLA-DQA2;HLA-DQA1;HLA-DQB2;HLA-DRB1	239.205	Reactome_2022	4	4.308
PD-1 Signaling R-HSA-389948	6.030E-05	HLA-DQA2;HLA-DQA1;HLA-DQB2;HLA-DRB1	220.513	Reactome_2022	4	4.220
Vacuolar Transport (GO:0007034)	1.149E-04	NPC1;PCSK9;CHMP3;TMEM106B;VPS37B	107.100	GO_Biological_Process_2023	5	3.940
Myofibril Assembly (GO:0030239)	1.149E-04	SYNPO2L;ACTN2;TNNT3;FLNC;MYOZ1	107.100	GO_Biological_Process_2023	5	3.940
Antigen Processing And Presentation Of Exogenous Peptide Antigen Via MHC Class II (GO:0019886)	1.446E-04	HLA-DQA2;HLA-DQA1;HLA-DQB2;HLA-DRB1	155.020	GO_Biological_Process_2023	4	3.840
Antigen Processing And Presentation Of Peptide Antigen Via MHC Class II (GO:0002495)	1.949E-04	HLA-DQA2;HLA-DQA1;HLA-DQB2;HLA-DRB1	137.296	GO_Biological_Process_2023	4	3.710
Response To Steroid Hormone (GO:0048545)	2.242E-04	MED1;TGFB1;CAV1;NR3C1	129.633	GO_Biological_Process_2023	4	3.649
Sarcomere Organization (GO:0045214)	2.242E-04	SYNPO2L;TNNT3;FLNC;MYOZ1	129.633	GO_Biological_Process_2023	4	3.649
Response To Epidermal Growth Factor (GO:0070849)	2.566E-04	MED1;ERBB2;MAPK3;ZPR1	122.640	GO_Biological_Process_2023	4	3.591
Immunoglobulin Mediated Immune Response (GO:0016064)	2.566E-04	HLA-DQA2;HLA-DQA1;HLA-DQB2;HLA-DRB1	122.640	GO_Biological_Process_2023	4	3.591
Antigen Processing And Presentation Of Exogenous Peptide Antigen (GO:0002478)	2.922E-04	HLA-DQA2;HLA-DQA1;HLA-DQB2;HLA-DRB1	116.235	GO_Biological_Process_2023	4	3.534
Generation Of Second Messenger Molecules R-HSA-202433	3.312E-04	HLA-DQA2;HLA-DQA1;HLA-DQB2;HLA-DRB1	110.352	Reactome_2022	4	3.480
Activation Of Adenylate Cyclase Activity (GO:0007190)	1.125E-03	GLP1R;EDNRA;GIPR	115.024	GO_Biological_Process_2023	3	2.949
Activation Of C3 And C5 R-HSA-174577	1.586E-03	C4A;CFB	308.117	Reactome_2022	2	2.800
Cellular Response To Hepatocyte Growth Factor Stimulus (GO:0035729)	1.586E-03	NELL1;BCAR1	308.117	GO_Biological_Process_2023	2	2.800
Response To Hepatocyte Growth Factor (GO:0035728)	1.586E-03	NELL1;BCAR1	308.117	GO_Biological_Process_2023	2	2.800
Dendrite Arborization (GO:0140059)	1.586E-03	NELL1;PHACTR1	308.117	GO_Biological_Process_2023	2	2.800
RUNX3 Regulates CDKN1A Transcription R-HSA-8941855	2.205E-03	CDKN1A;TGFB1	233.881	Reactome_2022	2	2.657
ER-associated Misfolded Protein Catabolic Process (GO:0071712)	3.728E-03	BAG6;RNF5	152.699	GO_Biological_Process_2023	2	2.429
Receptor-Mediated Endocytosis Of Virus By Host Cell (GO:0019065)	4.628E-03	CAV2;CAV1	128.438	GO_Biological_Process_2023	2	2.335
Regulation Of Cytoplasmic Transport (GO:1903649)	5.618E-03	CHMP3;MAPK3	110.045	GO_Biological_Process_2023	2	2.250
Post-Transcriptional Gene Silencing (GO:0016441)	5.618E-03	CELF1;HELZ	110.045	GO_Biological_Process_2023	2	2.250
Positive Regulation Of ERAD Pathway (GO:1904294)	6.695E-03	BAG6;CAV1	95.683	GO_Biological_Process_2023	2	2.174
