# Supplementary material for: Connecting intermediate phenotypes to disease using multi-omics in heart failure
Source: Pac Symp Biocomput. Author manuscript; Available in PMC 2025 Feb 13. (PMC11822568; doi:10.1142/9789819807024_0036)
Supplement: Supplemental Table 1 (A-F) [file NIHMS2038838-supplement-Supplemental_Table_1__A-F_.pdf]

Supplementary Table 1A: Left venticular ejectrion fraction (LVEF)														
gene	gene_name	zscore	effect_size	pvalue	var_g	pred_	pred	pred_	n_snps_used	n_snps_in_cov	n_snps_in_model	best_gwas_p	largest_weight	Tissue
ENSG00000170873.18	MTSS1	8.395	0.170	4.655E-17	0.045				2	2	2	1.600E-21	3.219E-01	Heart_Atrial_Appendag
ENSG00000099953.9	MMP11	7.988	0.176	1.377E-15	0.049				2	2	2	1.400E-15	3.704E-01	Heart_Left_Ventricle
ENSG00000099953.9	MMP11	7.985	0.202	1.400E-15	0.039				1	1	1	1.400E-15	3.407E-01	Heart_Atrial_Appendag
ENSG00000271401.1	RP11-171I2.3	7.930	1.036	2.200E-15	0.001				1	1	1	2.200E-15	6.549E-02	Adipose_Visceral_Om
ENSG00000249816.6	LINC00964	7.789	0.165	6.743E-15	0.042				3	3	3	4.700E-21	2.349E-01	Heart_Left_Ventricle
ENSG00000186510.11	CLCNKA	-7.696	-0.257	1.400E-14	0.019				1	1	1	1.400E-14	2.162E-01	Kidney_Cortex
ENSG00000185519.8	FAM131C	7.105	2.197	1.200E-12	0.000				1	1	1	1.200E-12	2.368E-02	Heart_Left_Ventricle
ENSG00000186510.11	CLCNKA	-6.964	-0.118	3.300E-12	0.088				1	1	1	3.300E-12	3.994E-01	Artery_Coronary
ENSG00000186510.11	CLCNKA	-6.964	-0.101	3.300E-12	0.110				1	1	1	3.300E-12	4.671E-01	Artery_Aorta
ENSG00000116809.11	ZBTB17	-6.897	-375.343	5.300E-12	0.000				1	1	1	5.300E-12	1.234E-04	Liver
ENSG00000116809.11	ZBTB17	6.897	9.886	5.300E-12	0.000				1	1	1	5.300E-12	4.683E-03	Artery_Aorta
ENSG00000116809.11	ZBTB17	6.897	13.074	5.300E-12	0.000				1	1	1	5.300E-12	3.542E-03	Whole_Blood
ENSG00000116809.11	ZBTB17	6.897	47.087	5.300E-12	0.000				1	1	1	5.300E-12	9.833E-04	Heart_Atrial_Appendag
ENSG00000107651.12	SEC23IP	6.439	0.626	1.200E-10	0.002				1	1	1	1.200E-10	7.087E-02	Adipose_Subcutaneou
ENSG00000136383.6	ALPK3	6.397	0.265	1.589E-10	0.014				2	2	2	6.700E-10	1.479E-01	Heart_Atrial_Appendag
ENSG00000124762.13	CDKN1A	6.369	1.822	1.900E-10	0.000				1	1	1	1.900E-10	2.761E-02	Heart_Left_Ventricle
ENSG00000065526.10	SPEN	-6.340	-1.087	2.300E-10	0.001				1	1	1	2.300E-10	3.895E-02	Artery_Aorta
ENSG00000065526.10	SPEN	-6.340	-1.323	2.300E-10	0.000				1	1	1	2.300E-10	3.200E-02	Adipose_Visceral_Om
ENSG00000065526.10	SPEN	-6.129	-1.009	8.835E-10	0.001				2	2	2	2.300E-10	6.847E-02	Whole_Blood
ENSG00000099956.18	SMARCB1	6.108	0.097	1.010E-09	0.088				2	2	2	1.300E-07	4.972E-01	Kidney_Cortex
ENSG00000173641.17	HSPB7	-5.974	-0.078	2.322E-09	0.137				3	3	3	3.300E-12	3.307E-01	Kidney_Cortex
ENSG00000186510.11	CLCNKA	-5.916	-0.064	3.305E-09	0.214				3	3	3	1.000E-08	4.000E-01	Heart_Atrial_Appendag
ENSG00000128591.15	FLNC	-5.911	-2.215	3.400E-09	0.000				1	1	1	3.400E-09	2.753E-02	Heart_Left_Ventricle
ENSG00000128591.15	FLNC	5.911	14.301	3.400E-09	0.000				1	1	1	3.400E-09	4.264E-03	Artery_Coronary
ENSG00000128591.15	FLNC	5.911	1.074	3.400E-09	0.001				1	1	1	3.400E-09	5.676E-02	Kidney_Cortex
ENSG00000128591.15	FLNC	-5.911	-3.237	3.400E-09	0.000				1	1	1	3.400E-09	1.884E-02	Whole_Blood
ENSG00000065526.10	SPEN	-5.861	-1.154	4.600E-09	0.001				1	1	1	4.600E-09	3.688E-02	Artery_Tibial
ENSG00000136383.6	ALPK3	5.852	0.499	4.871E-09	0.003				2	2	2	6.400E-09	9.668E-02	Artery_Tibial
ENSG00000177082.12	WDR73	-5.843	-0.135	5.116E-09	0.047				2	2	2	3.300E-09	3.141E-01	Adipose_Subcutaneou
ENSG00000089159.16	PXN	-5.807	-0.479	6.349E-09	0.003				2	2	2	8.900E-09	2.826E-01	Artery_Tibial
ENSG00000128596.16	CCDC136	-5.788	-0.260	7.126E-09	0.013				2	2	2	7.200E-09	2.280E-01	Artery_Tibial

gene	gene_name	zscore	effect_size	pvalue	var_g	pred_	pred	pred_	n_snps_used	n_snps_in_cov	n_snps_in_model	best_gwas_p	largest_weight	Tissue
ENSG00000089159.16	PXN	-5.750	-0.272	8.900E-09	0.008				1	1	1	8.900E-09	4.896E-01	Heart_Atrial_Appendag
ENSG00000228672.3	PROB1	5.745	0.153	9.200E-09	0.034				1	2	2	9.200E-09	2.921E-01	Adipose_Subcutaneou
ENSG00000228672.3	PROB1	5.745	0.103	9.200E-09	0.075				1	1	1	9.200E-09	4.350E-01	Artery_Tibial
ENSG00000228672.3	PROB1	5.745	0.132	9.200E-09	0.044				1	1	1	9.200E-09	3.376E-01	Artery_Aorta
ENSG00000228672.3	PROB1	5.745	0.330	9.200E-09	0.007				1	1	1	9.200E-09	1.355E-01	Heart_Atrial_Appendag
ENSG00000228672.3	PROB1	5.745	0.149	9.200E-09	0.036				1	1	1	9.200E-09	3.000E-01	Artery_Coronary
ENSG00000079150.17	FKBP7	-5.737	-0.129	9.610E-09	0.041				2	2	2	2.700E-07	3.413E-01	Heart_Left_Ventricle
ENSG00000272899.3	RP11-309L24.	-5.734	-0.137	9.833E-09	0.034				3	3	3	1.400E-08	2.000E-01	Whole_Blood
ENSG00000228672.3	PROB1	5.662	0.310	1.498E-08	0.008				2	2	2	9.200E-09	1.390E-01	Heart_Left_Ventricle
ENSG00000128596.16	CCDC136	5.545	0.237	2.943E-08	0.011				2	2	2	2.400E-08	1.830E-01	Whole_Blood
ENSG00000151923.17	TIAL1	5.541	0.524	3.012E-08	0.003				2	2	2	5.700E-08	7.547E-02	Artery_Tibial
ENSG00000128218.7	VPREB3	-5.530	-0.094	3.195E-08	0.095				2	2	2	2.300E-08	5.422E-01	Artery_Aorta
ENSG00000170464.9	DNAJC18	-5.525	-0.147	3.300E-08	0.034				1	1	1	3.300E-08	2.922E-01	Artery_Tibial
ENSG00000170464.9	DNAJC18	-5.525	-0.064	3.300E-08	0.179				1	1	1	3.300E-08	6.771E-01	Heart_Atrial_Appendag
ENSG00000170464.9	DNAJC18	-5.525	-0.100	3.300E-08	0.093				1	1	1	3.300E-08	4.313E-01	Kidney_Cortex
ENSG00000121440.14	PDZRN3	5.514	0.167	3.505E-08	0.022				3	3	3	4.800E-08	2.310E-01	Heart_Left_Ventricle
ENSG00000169314.14	C22orf15	5.505	0.272	3.700E-08	0.010				1	1	1	3.700E-08	1.886E-01	Whole_Blood
ENSG00000128596.16	CCDC136	5.497	0.190	3.865E-08	0.018				2	2	2	3.400E-08	2.156E-01	Heart_Atrial_Appendag
ENSG00000170482.16	SLC23A1	5.495	1.317	3.900E-08	0.000				1	1	1	3.900E-08	3.153E-02	Artery_Coronary
ENSG00000170482.16	SLC23A1	5.495	0.453	3.900E-08	0.003				1	1	1	3.900E-08	9.162E-02	Artery_Aorta
ENSG00000170482.16	SLC23A1	5.495	1.080	3.900E-08	0.001				1	1	1	3.900E-08	3.844E-02	Heart_Atrial_Appendag
ENSG00000170482.16	SLC23A1	5.495	0.157	3.900E-08	0.028				1	1	1	3.900E-08	2.649E-01	Heart_Left_Ventricle
ENSG00000128218.7	VPREB3	-5.494	-0.053	3.927E-08	0.249				3	3	3	3.100E-08	5.539E-01	Adipose_Subcutaneou
ENSG00000170464.9	DNAJC18	-5.444	-0.074	5.200E-08	0.125				1	1	1	5.200E-08	5.737E-01	Heart_Left_Ventricle
ENSG00000170482.16	SLC23A1	5.444	0.445	5.200E-08	0.003				1	1	1	5.200E-08	8.850E-02	Whole_Blood
ENSG00000249816.6	LINC00964	5.430	0.101	5.627E-08	0.056				3	3	3	4.700E-21	2.156E-01	Heart_Atrial_Appendag
ENSG00000151923.17	TIAL1	5.417	0.417	6.065E-08	0.004				2	2	2	7.600E-08	9.169E-02	Adipose_Subcutaneou
ENSG00000169314.14	C22orf15	5.396	0.263	6.800E-08	0.010				1	2	2	6.800E-08	1.764E-01	Artery_Tibial
ENSG00000079150.17	FKBP7	-5.380	-0.094	7.464E-08	0.060				2	2	2	7.900E-07	4.172E-01	Artery_Coronary
ENSG00000170464.9	DNAJC18	-5.372	-0.077	7.787E-08	0.110				2	2	2	3.300E-08	3.480E-01	Adipose_Visceral_Om
ENSG00000177082.12	WDR73	-5.363	-0.088	8.186E-08	0.093				2	2	2	8.400E-08	4.836E-01	Artery_Tibial
ENSG00000177082.12	WDR73	-5.356	-0.172	8.498E-08	0.026				2	2	2	8.700E-08	2.523E-01	Heart_Left_Ventricle
ENSG00000177082.12	WDR73	-5.336	-0.119	9.497E-08	0.050				2	2	2	9.900E-08	3.567E-01	Adipose_Visceral_Om

gene	gene_name	zscore	effect_size	pvalue	var_g	pred_	pred_	pred_	n_snps_used	n_snps_in_cov	n_snps_in_model	best_gwas_p	largest_weight	Tissue
ENSG00000128218.7	VPREB3	-5.335	-0.058	9.556E-08	0.184				3	3	3	6.800E-08	4.537E-01	Adipose_Visceral_Om
ENSG00000177082.12	WDR73	-5.332	-0.090	9.734E-08	0.094				2	2	2	9.900E-08	4.691E-01	Artery_Aorta
ENSG00000177082.12	WDR73	-5.330	-0.266	9.818E-08	0.010				2	2	2	9.600E-08	1.594E-01	Whole_Blood
ENSG00000170469.10	SPATA24	-5.327	-0.153	1.000E-07	0.028				1	1	1	1.000E-07	2.530E-01	Adipose_Subcutaneou
ENSG00000170469.10	SPATA24	-5.327	-0.246	1.000E-07	0.013				1	1	1	1.000E-07	1.570E-01	Kidney_Cortex
ENSG00000272899.3	RP11-309L24.	-5.317	-0.173	1.055E-07	0.021				2	2	2	1.100E-07	2.068E-01	Liver
ENSG00000170469.10	SPATA24	-5.309	-0.169	1.100E-07	0.022				1	1	1	1.100E-07	2.280E-01	Heart_Atrial_Appendag
ENSG00000170469.10	SPATA24	-5.309	-0.152	1.100E-07	0.028				1	1	1	1.100E-07	2.527E-01	Whole_Blood
ENSG00000272899.3	RP11-309L24.	-5.309	-0.283	1.100E-07	0.007				1	1	1	1.100E-07	1.549E-01	Artery_Aorta
ENSG00000170469.10	SPATA24	-5.309	-0.154	1.100E-07	0.026				1	1	1	1.100E-07	2.502E-01	Heart_Left_Ventricle
ENSG00000170469.10	SPATA24	-5.309	-0.142	1.100E-07	0.031				1	1	1	1.100E-07	2.722E-01	Adipose_Visceral_Om
ENSG00000170464.9	DNAJC18	-5.298	-0.148	1.168E-07	0.031				2	2	2	3.300E-08	1.685E-01	Adipose_Subcutaneou
ENSG00000272899.3	RP11-309L24.	-5.297	-0.244	1.177E-07	0.011				2	2	2	1.100E-07	1.528E-01	Heart_Atrial_Appendag
ENSG00000170464.9	DNAJC18	-5.279	-0.051	1.297E-07	0.223				2	2	2	3.300E-08	4.808E-01	Liver
ENSG00000228672.3	PROB1	5.279	0.157	1.300E-07	0.027				1	1	1	1.300E-07	2.436E-01	Whole_Blood
ENSG00000099956.18	SMARCB1	5.279	0.135	1.300E-07	0.040				1	1	1	1.300E-07	4.204E-01	Heart_Left_Ventricle
ENSG00000170476.15	MZB1	5.279	1.425	1.300E-07	0.000				1	1	1	1.300E-07	2.678E-02	Heart_Left_Ventricle
ENSG00000170464.9	DNAJC18	-5.256	-0.067	1.469E-07	0.151				2	2	2	3.300E-08	3.584E-01	Whole_Blood
ENSG00000272899.3	RP11-309L24.	-5.249	-0.138	1.527E-07	0.031				2	2	2	1.100E-07	2.504E-01	Artery_Tibial
ENSG00000197696.9	NMB	-5.241	-0.181	1.600E-07	0.021				1	1	1	1.600E-07	2.314E-01	Artery_Coronary
ENSG00000197696.9	NMB	-5.241	-0.112	1.600E-07	0.059				1	1	1	1.600E-07	3.751E-01	Artery_Aorta
ENSG00000197696.9	NMB	-5.241	-0.170	1.600E-07	0.025				1	1	1	1.600E-07	2.463E-01	Heart_Atrial_Appendag
ENSG00000197696.9	NMB	-5.241	-0.169	1.600E-07	0.025				1	1	1	1.600E-07	2.482E-01	Heart_Left_Ventricle
ENSG00000197696.9	NMB	-5.241	-0.096	1.600E-07	0.076				1	1	1	1.600E-07	4.381E-01	Liver
ENSG00000197696.9	NMB	-5.241	-0.157	1.600E-07	0.028				1	1	1	1.600E-07	2.663E-01	Artery_Tibial
ENSG00000197696.9	NMB	-5.241	-0.266	1.600E-07	0.010				1	1	1	1.600E-07	1.577E-01	Adipose_Visceral_Om
ENSG00000173641.17	HSPB7	5.236	0.456	1.641E-07	0.003				2	2	2	3.300E-12	6.982E-02	Heart_Left_Ventricle
ENSG00000197696.9	NMB	-5.229	-0.213	1.700E-07	0.015				1	1	1	1.700E-07	1.963E-01	Whole_Blood
ENSG00000089159.16	PXN	-5.229	-0.353	1.700E-07	0.005				1	1	1	1.700E-07	3.017E-01	Heart_Left_Ventricle
ENSG00000197696.9	NMB	-5.229	-0.106	1.700E-07	0.079				1	1	1	1.700E-07	3.932E-01	Kidney_Cortex
ENSG00000255491.2	RP11-1082L8.	5.227	1.618	1.722E-07	0.000				2	2	2	1.900E-07	2.261E-02	Adipose_Visceral_Om
ENSG00000170464.9	DNAJC18	-5.207	-0.088	1.918E-07	0.088				2	2	2	3.300E-08	2.580E-01	Artery_Coronary
ENSG00000169762.16	TAPT1	-5.194	-0.260	2.056E-07	0.009				3	3	3	3.600E-07	1.498E-01	Artery_Tibial
ENSG00000262655.3	SPON1	-5.180	-0.047	2.216E-07	0.286				2	2	2	2.500E-08	4.914E-01	Heart_Left_Ventricle
ENSG00000108924.13	HLF	5.157	0.345	2.508E-07	0.005				2	2	2	2.600E-07	1.143E-01	Heart_Left_Ventricle
ENSG00000128218.7	VPREB3	-5.130	-0.083	2.895E-07	0.101				2	2	2	3.100E-08	4.760E-01	Liver
ENSG00000173641.17	HSPB7	5.128	0.189	2.921E-07	0.018				3	3	3	3.300E-12	1.225E-01	Heart_Atrial_Appendag

gene	gene_name	zscore	effect_size	pvalue	var_g	pred_	pred	pred_	n_snps_used	n_snps_in_cov	n_snps_in_model	best_gwas_p	largest_weight	Tissue
ENSG00000180228.12	PRKRA	-5.117	-0.590	3.100E-07	0.001				1	1	1	3.100E-07	1.765E-01	Artery_Aorta
ENSG00000099956.18	SMARCB1	5.104	0.104	3.333E-07	0.062				2	2	2	1.300E-07	5.333E-01	Adipose_Visceral_Om
ENSG00000107651.12	SEC23IP	5.102	0.646	3.354E-07	0.001				2	2	2	5.700E-07	5.760E-02	Artery_Coronary
ENSG00000007062.11	PROM1	-5.084	-0.052	3.700E-07	0.224				1	1	1	3.700E-07	8.113E-01	Artery_Coronary

Supplementary Table 1B: Left venticular mass (LVM)

gene	gene_name	zscore	effect_size	pvalue	var_g	pred_	pred_p	pred_	n_snps_used	n_snps_in_cov	n_snps_in_model	best_gwas_p	largest_weig	Tissue
ENSG00000271401.1	RP11-171I2.3	-10.084	-14.314	6.500E-24	0.001				1	1	1	6.500E-24	0.065	Adipose_Visc
ENSG00000079150.17	FKBP7	8.551	2.164	1.225E-17	0.041				2	2	2	4.600E-19	0.341	Heart_Left_Ve
ENSG00000079150.17	FKBP7	8.283	1.662	1.198E-16	0.063				2	2	2	1.700E-16	0.459	Artery_Tibial
ENSG00000079150.17	FKBP7	8.242	2.802	1.700E-16	0.021				1	1	1	1.700E-16	0.276	Heart_Atrial_A
ENSG00000079150.17	FKBP7	8.240	2.702	1.716E-16	0.024				2	2	2	1.700E-16	0.278	Adipose_Visc
ENSG00000079150.17	FKBP7	8.180	1.638	2.847E-16	0.060				2	2	2	1.700E-16	0.417	Artery_Corona
ENSG00000079150.17	FKBP7	7.572	2.233	3.664E-14	0.029				2	2	2	5.300E-14	0.326	Adipose_Sub
ENSG00000079150.17	FKBP7	7.524	2.873	5.300E-14	0.017				1	1	1	5.300E-14	0.261	Whole_Blood
ENSG00000180228.12	PRKRA	7.455	16.687	9.000E-14	0.000				1	1	1	9.000E-14	0.044	Heart_Atrial_A
ENSG00000180228.12	PRKRA	-7.434	-21.860	1.057E-13	0.000				2	2	2	9.000E-14	0.034	Artery_Tibial
ENSG00000079150.17	FKBP7	7.357	1.307	1.874E-13	0.087				2	2	2	1.700E-16	0.505	Artery_Aorta
ENSG00000108379.9	WNT3	-6.937	-1.295	4.014E-12	0.081				2	2	2	1.300E-09	0.419	Heart_Atrial_A
ENSG00000108379.9	WNT3	-6.821	-0.801	9.047E-12	0.205				2	2	2	1.700E-09	0.538	Adipose_Sub
ENSG00000204311.12	DFNB59	6.557	9.733	5.500E-11	0.001				1	1	1	5.500E-11	0.056	Artery_Aorta
ENSG00000180228.12	PRKRA	6.535	15.110	6.365E-11	0.000				2	2	2	5.500E-11	0.036	Heart_Left_Ve
ENSG00000108379.9	WNT3	-6.470	-1.174	9.811E-11	0.090				2	2	2	7.500E-08	0.392	Artery_Corona
ENSG00000108379.9	WNT3	-6.360	-1.157	2.012E-10	0.090				2	2	2	1.300E-09	0.403	Artery_Tibial
ENSG00000177791.11	MYOZ1	-6.124	-0.431	9.115E-10	0.509				2	2	2	5.600E-08	1.044	Heart_Atrial_A
ENSG00000108379.9	WNT3	-6.091	-2.668	1.119E-09	0.015				2	2	2	4.400E-09	0.205	Whole_Blood
ENSG00000108379.9	WNT3	-6.067	-0.914	1.300E-09	0.127				1	1	1	1.300E-09	0.565	Artery_Aorta
ENSG00000165916.8	PSMC3	5.901	2.676	3.612E-09	0.013				2	2	2	3.000E-06	0.138	Artery_Aorta
ENSG00000165916.8	PSMC3	5.891	3.417	3.840E-09	0.008				2	2	2	3.000E-06	0.106	Artery_Tibial
ENSG00000108379.9	WNT3	-5.868	-1.137	4.400E-09	0.082				1	2	2	4.400E-09	0.481	Heart_Left_Ve
ENSG00000165916.8	PSMC3	5.830	3.000	5.554E-09	0.011				2	2	2	3.000E-06	0.130	Adipose_Visc
ENSG00000116095.10	PLEKHA3	5.632	4.203	1.784E-08	0.006				2	2	2	1.300E-10	0.074	Liver
ENSG00000272899.3	RP11-309L24.	5.390	1.501	7.053E-08	0.034				3	3	3	1.600E-07	0.200	Whole_Blood
ENSG00000170606.14	HSPA4	5.372	7.381	7.800E-08	0.001				1	2	2	7.800E-08	0.064	Artery_Tibial
ENSG00000170606.14	HSPA4	5.372	4.289	7.800E-08	0.003				1	1	1	7.800E-08	0.110	Kidney_Corte
ENSG00000079150.17	FKBP7	5.289	2.313	1.229E-07	0.014				2	2	2	5.300E-14	0.259	Liver
ENSG00000170606.14	HSPA4	5.258	7.034	1.453E-07	0.001				2	2	2	7.800E-08	0.065	Heart_Left_Ve
ENSG00000170606.14	HSPA4	5.156	8.837	2.528E-07	0.001				2	3	3	4.200E-07	0.053	Adipose_Sub

gene	gene_name	zscore	effect_size	pvalue	var_g	pred_	pred_p	pred_	n_snps_used	n_snps_in_cov	n_snps_in_model	best_gwas_p	largest_weig	Tissue
ENSG00000272899.3	RP11-309L24.	5.136	3.169	2.800E-07	0.007				1	1	1	2.800E-07	0.155	Artery_Aorta
ENSG00000108379.9	WNT3	-5.130	-1.464	2.900E-07	0.040				1	1	1	2.900E-07	0.305	Kidney_Corte
ENSG00000108379.9	WNT3	-5.124	-0.517	2.990E-07	0.260				3	3	3	1.000E-06	0.538	Adipose_Visc
ENSG00000167244.18	IGF2	5.117	4.052	3.100E-07	0.004				1	1	1	3.100E-07	0.095	Artery_Tibial

Supplementary Table 1C: Left venticular-end systolic volume (LVESV)														
gene	gene_name	zscore	effect_size	pvalue	var_g	pred_pe	pred_p	pred_p	n_snps_used	n_snps_in_cov	n_snps_in_model	best_gwas_p	largest_weight	Tissue
ENSG00000271401.1	RP11-171I2.3	-9.599	-1.200	8.100E-22	0.001				1	1	1	8.100E-22	0.065	Adipose_Visceral_Om
ENSG00000099953.9	MMP11	-7.919	-0.186	2.400E-15	0.039				1	1	1	2.400E-15	0.341	Heart_Atrial_Appendag
ENSG00000186510.11	CLCNKA	7.883	0.249	3.200E-15	0.019				1	1	1	3.200E-15	0.216	Kidney_Cortex
ENSG00000099953.9	MMP11	-7.833	-0.161	4.756E-15	0.049				2	2	2	2.400E-15	0.370	Heart_Left_Ventricle
ENSG00000079150.17	FKBP7	7.695	0.168	1.410E-14	0.041				2	2	2	3.000E-14	0.341	Heart_Left_Ventricle
ENSG00000170873.18	MTSS1	-7.481	-0.144	7.399E-14	0.045				2	2	2	1.100E-18	0.322	Heart_Atrial_Appendag
ENSG00000249816.6	LINC00964	-7.454	-0.150	9.049E-14	0.042				3	3	3	6.000E-18	0.235	Heart_Left_Ventricle
ENSG00000151923.17	TIAL1	-7.143	-0.634	9.155E-13	0.003				2	2	2	2.300E-12	0.075	Artery_Tibial
ENSG00000186510.11	CLCNKA	7.084	0.112	1.400E-12	0.088				1	1	1	1.400E-12	0.399	Artery_Coronary
ENSG00000186510.11	CLCNKA	7.084	0.096	1.400E-12	0.110				1	1	1	1.400E-12	0.467	Artery_Aorta
ENSG00000136383.6	ALPK3	-7.048	-0.276	1.813E-12	0.014				2	2	2	1.500E-11	0.148	Heart_Atrial_Appendag
ENSG00000079150.17	FKBP7	6.982	0.120	2.918E-12	0.060				2	2	2	4.300E-11	0.417	Artery_Coronary
ENSG00000151923.17	TIAL1	-6.888	-0.496	5.645E-12	0.004				2	2	2	6.400E-12	0.092	Adipose_Subcutaneou
ENSG00000079150.17	FKBP7	6.756	0.193	1.423E-11	0.024				2	2	2	4.300E-11	0.278	Adipose_Visceral_Om
ENSG00000116809.11	ZBTB17	-6.668	-11.676	2.600E-11	0.000				1	1	1	2.600E-11	0.004	Whole_Blood
ENSG00000116809.11	ZBTB17	6.668	335.212	2.600E-11	0.000				1	1	1	2.600E-11	0.000	Liver
ENSG00000116809.11	ZBTB17	-6.668	-8.829	2.600E-11	0.000				1	1	1	2.600E-11	0.005	Artery_Aorta
ENSG00000116809.11	ZBTB17	-6.668	-42.053	2.600E-11	0.000				1	1	1	2.600E-11	0.001	Heart_Atrial_Appendag
ENSG00000079150.17	FKBP7	6.593	0.197	4.300E-11	0.021				1	1	1	4.300E-11	0.276	Heart_Atrial_Appendag
ENSG00000079150.17	FKBP7	6.592	0.116	4.329E-11	0.063				2	2	2	4.300E-11	0.459	Artery_Tibial
ENSG00000136383.6	ALPK3	-6.541	-0.528	6.092E-11	0.003				2	2	2	2.900E-11	0.097	Artery_Tibial
ENSG00000089159.16	PXN	6.485	0.284	8.900E-11	0.008				1	1	1	8.900E-11	0.490	Heart_Atrial_Appendag
ENSG00000089159.16	PXN	6.460	0.492	1.047E-10	0.003				2	2	2	8.900E-11	0.283	Artery_Tibial
ENSG00000272899.3	RP11-309L24.4	6.428	0.144	1.297E-10	0.034				3	3	3	1.500E-10	0.200	Whole_Blood
ENSG00000176182.5	MYPOP	6.378	2.526	1.800E-10	0.000				1	1	1	1.800E-10	0.017	Adipose_Visceral_Om
ENSG00000170608.2	FOXA3	6.378	271.179	1.800E-10	0.000				1	1	1	1.800E-10	0.000	Adipose_Subcutaneou
ENSG00000125755.18	SYMPK	-6.378	-0.660	1.800E-10	0.002				1	2	2	1.800E-10	0.066	Heart_Left_Ventricle
ENSG00000185800.11	DMWD	-6.370	-0.379	1.889E-10	0.006				2	2	2	1.700E-10	0.120	Heart_Left_Ventricle
ENSG00000128591.15	FLNC	-6.361	-14.576	2.000E-10	0.000				1	1	1	2.000E-10	0.004	Artery_Coronary
ENSG00000128591.15	FLNC	-6.361	-1.095	2.000E-10	0.001				1	1	1	2.000E-10	0.057	Kidney_Cortex
ENSG00000128591.15	FLNC	6.361	3.300	2.000E-10	0.000				1	1	1	2.000E-10	0.019	Whole_Blood

gene	gene_name	zscore	effect_size	pvalue	var_g	pred_pe	pred_p	pred_pe	n_snps_used	n_snps_in_cov	n_snps_in_model	best_gwas_p	largest_weight	Tissue
ENSG00000128591.15	FLNC	6.361	2.258	2.000E-10	0.000				1	1	1	2.000E-10	0.028	Heart_Left_Ventricle
ENSG00000177082.12	WDR73	6.345	0.136	2.231E-10	0.047				2	2	2	1.400E-10	0.314	Adipose_Subcutaneou
ENSG00000128596.16	CCDC136	6.339	0.269	2.318E-10	0.013				2	2	2	2.000E-10	0.228	Artery_Tibial
ENSG00000185519.8	FAM131C	-6.171	-1.787	6.800E-10	0.000				1	1	1	6.800E-10	0.024	Heart_Left_Ventricle
ENSG00000177082.12	WDR73	6.153	0.185	7.616E-10	0.026				2	2	2	8.100E-10	0.252	Heart_Left_Ventricle
ENSG00000185800.11	DMWD	-6.118	-0.190	9.470E-10	0.022				2	2	2	3.400E-10	0.196	Adipose_Visceral_Om
ENSG00000065526.10	SPEN	6.113	1.133	9.800E-10	0.001				1	1	1	9.800E-10	0.037	Artery_Tibial
ENSG00000151923.17	TIAL1	6.055	2.178	1.400E-09	0.000				1	1	1	1.400E-09	0.022	Whole_Blood
ENSG00000197696.9	NMB	6.034	0.185	1.600E-09	0.025				1	1	1	1.600E-09	0.246	Heart_Atrial_Appendag
ENSG00000197696.9	NMB	6.034	0.171	1.600E-09	0.028				1	1	1	1.600E-09	0.266	Artery_Tibial
ENSG00000197696.9	NMB	6.034	0.197	1.600E-09	0.021				1	1	1	1.600E-09	0.231	Artery_Coronary
ENSG00000197696.9	NMB	6.034	0.104	1.600E-09	0.076				1	1	1	1.600E-09	0.438	Liver
ENSG00000197696.9	NMB	6.034	0.184	1.600E-09	0.025				1	1	1	1.600E-09	0.248	Heart_Left_Ventricle
ENSG00000197696.9	NMB	6.034	0.122	1.600E-09	0.059				1	1	1	1.600E-09	0.375	Artery_Aorta
ENSG00000197696.9	NMB	6.034	0.289	1.600E-09	0.010				1	1	1	1.600E-09	0.158	Adipose_Visceral_Om
ENSG00000197696.9	NMB	6.015	0.116	1.800E-09	0.079				1	1	1	1.800E-09	0.393	Kidney_Cortex
ENSG00000197696.9	NMB	6.015	0.232	1.800E-09	0.015				1	1	1	1.800E-09	0.196	Whole_Blood
ENSG00000173641.17	HSPB7	5.992	0.074	2.079E-09	0.137				3	3	3	1.400E-12	0.331	Kidney_Cortex
ENSG00000177082.12	WDR73	5.984	0.093	2.176E-09	0.093				2	2	2	2.300E-09	0.484	Artery_Tibial
ENSG00000262655.3	SPON1	5.980	0.052	2.232E-09	0.286				2	2	2	4.500E-11	0.491	Heart_Left_Ventricle
ENSG00000116095.10	PLEKHA3	5.980	0.394	2.234E-09	0.006				2	2	2	3.200E-08	0.074	Liver
ENSG00000099956.18	SMARCB1	-5.977	-0.087	2.277E-09	0.088				2	2	2	2.300E-09	0.497	Kidney_Cortex
ENSG00000099956.18	SMARCB1	-5.975	-0.140	2.300E-09	0.040				1	1	1	2.300E-09	0.420	Heart_Left_Ventricle
ENSG00000089159.16	PXN	5.926	0.377	3.100E-09	0.005				1	1	1	3.100E-09	0.302	Heart_Left_Ventricle
ENSG00000177082.12	WDR73	5.919	0.095	3.231E-09	0.094				2	2	2	3.400E-09	0.469	Artery_Aorta
ENSG00000177082.12	WDR73	5.919	0.279	3.235E-09	0.010				2	2	2	3.300E-09	0.159	Whole_Blood
ENSG00000177082.12	WDR73	5.918	0.125	3.252E-09	0.050				2	2	2	3.400E-09	0.357	Adipose_Visceral_Om
ENSG00000128591.15	FLNC	5.916	6.165	3.300E-09	0.000				1	1	1	3.300E-09	0.011	Artery_Aorta
ENSG00000128591.15	FLNC	-5.916	-0.571	3.300E-09	0.002				1	1	1	3.300E-09	0.114	Liver
ENSG00000272899.3	RP11-309L24.4	5.906	0.293	3.500E-09	0.007				1	1	1	3.500E-09	0.155	Artery_Aorta
ENSG00000177082.12	WDR73	5.891	0.157	3.844E-09	0.026				2	2	2	4.000E-09	0.246	Artery_Coronary
ENSG00000178053.17	MLF1	-5.888	-0.069	3.911E-09	0.142				3	3	3	1.500E-07	0.330	Heart_Left_Ventricle
ENSG00000128596.16	CCDC136	5.881	0.272	4.082E-09	0.009				2	2	2	3.700E-09	0.237	Artery_Aorta

gene	gene_name	zscore	effect_size	pvalue	var_g	pred_pe	pred_p	pred_pe	n_snps_used	n_snps_in_cov	n_snps_in_model	best_gwas_p	largest_weight	Tissue
ENSG00000177051.5	FBXO46	5.861	0.566	4.609E-09	0.002				2	2	2	4.800E-09	0.117	Artery_Tibial
ENSG00000177051.5	FBXO46	5.854	0.715	4.800E-09	0.001				1	1	1	4.800E-09	0.058	Artery_Coronary
ENSG00000177051.5	FBXO46	5.854	0.635	4.800E-09	0.002				1	1	1	4.800E-09	0.065	Artery_Aorta
ENSG00000237452.2	BHMG1	-5.854	-23.253	4.800E-09	0.000				1	1	1	4.800E-09	0.002	Heart_Atrial_Appendag
ENSG00000177051.5	FBXO46	5.854	0.724	4.800E-09	0.001				1	1	1	4.800E-09	0.057	Adipose_Subcutaneou
ENSG00000177051.5	FBXO46	5.854	0.669	4.800E-09	0.002				1	1	1	4.800E-09	0.062	Adipose_Visceral_Om
ENSG00000173641.17	HSPB7	-5.839	-0.458	5.244E-09	0.003				2	2	2	1.400E-12	0.070	Heart_Left_Ventricle
ENSG00000169314.14	C22orf15	-5.784	-0.263	7.300E-09	0.010				1	2	2	7.300E-09	0.176	Artery_Tibial
ENSG00000128596.16	CCDC136	-5.730	-0.233	1.005E-08	0.011				2	2	2	1.000E-08	0.183	Whole_Blood
ENSG00000099956.18	SMARCB1	-5.711	-0.110	1.120E-08	0.059				3	3	3	2.300E-09	0.496	Heart_Atrial_Appendag
ENSG00000065526.10	SPEN	5.700	0.924	1.200E-08	0.001				1	1	1	1.200E-08	0.039	Artery_Aorta
ENSG00000065526.10	SPEN	5.700	1.124	1.200E-08	0.000				1	1	1	1.200E-08	0.032	Adipose_Visceral_Om
ENSG00000178053.17	MLF1	-5.699	-0.104	1.207E-08	0.056				2	2	2	1.500E-07	0.273	Heart_Atrial_Appendag
ENSG00000178053.17	MLF1	-5.678	-0.114	1.360E-08	0.049				3	3	3	1.500E-07	0.239	Artery_Tibial
ENSG00000128596.16	CCDC136	5.655	0.184	1.556E-08	0.020				2	2	2	3.700E-09	0.172	Artery_Coronary
ENSG00000128218.7	VPREB3	5.635	0.282	1.756E-08	0.009				2	2	2	5.600E-08	0.148	Whole_Blood
ENSG00000107651.12	SEC23IP	-5.630	-0.509	1.800E-08	0.002				1	1	1	1.800E-08	0.071	Adipose_Subcutaneou
ENSG00000272899.3	RP11-309L24.4	5.584	0.239	2.355E-08	0.011				2	2	2	3.500E-09	0.153	Heart_Atrial_Appendag
ENSG00000186510.11	CLCNKA	5.571	0.056	2.537E-08	0.214				3	3	3	5.500E-07	0.400	Heart_Atrial_Appendag
ENSG00000272899.3	RP11-309L24.4	5.559	0.169	2.710E-08	0.021				2	2	2	3.500E-09	0.207	Liver
ENSG00000145817.16	YIPF5	-5.536	-4.144	3.100E-08	0.000				1	2	2	3.100E-08	0.012	Liver
ENSG00000145817.16	YIPF5	5.536	0.489	3.100E-08	0.003				1	1	1	3.100E-08	0.098	Artery_Coronary
ENSG00000145817.16	YIPF5	5.536	0.098	3.100E-08	0.071				1	1	1	3.100E-08	0.487	Whole_Blood
ENSG00000145817.16	YIPF5	5.536	0.323	3.100E-08	0.006				1	1	1	3.100E-08	0.148	Adipose_Visceral_Om
ENSG00000183775.10	KCTD16	-5.536	-0.562	3.100E-08	0.002				1	1	1	3.100E-08	0.085	Adipose_Subcutaneou
ENSG00000183775.10	KCTD16	-5.536	-0.778	3.100E-08	0.001				1	1	1	3.100E-08	0.062	Kidney_Cortex
ENSG00000183775.10	KCTD16	-5.536	-0.640	3.100E-08	0.002				1	1	1	3.100E-08	0.075	Artery_Coronary
ENSG00000183775.10	KCTD16	-5.536	-0.741	3.100E-08	0.001				1	1	1	3.100E-08	0.065	Artery_Tibial
ENSG00000099956.18	SMARCB1	-5.514	-0.101	3.507E-08	0.062				2	2	2	2.300E-09	0.533	Adipose_Visceral_Om
ENSG00000177082.12	WDR73	5.512	0.113	3.554E-08	0.057				2	2	2	3.800E-08	0.362	Heart_Atrial_Appendag
ENSG00000272899.3	RP11-309L24.4	5.458	0.134	4.828E-08	0.031				2	2	2	3.500E-09	0.250	Artery_Tibial
ENSG00000173641.17	HSPB7	-5.445	-0.182	5.180E-08	0.018				3	3	3	1.400E-12	0.122	Heart_Atrial_Appendag
ENSG00000128218.7	VPREB3	5.438	0.055	5.383E-08	0.184				3	3	3	7.300E-09	0.454	Adipose_Visceral_Om
ENSG00000183775.10	KCTD16	-5.437	-0.567	5.417E-08	0.002				2	2	2	3.100E-08	0.077	Adipose_Visceral_Om
ENSG00000196141.13	SPATS2L	5.425	0.182	5.806E-08	0.019				3	3	3	7.800E-06	0.277	Heart_Left_Ventricle
ENSG00000128218.7	VPREB3	5.416	0.084	6.105E-08	0.095				2	2	2	3.000E-08	0.542	Artery_Aorta
ENSG00000128218.7	VPREB3	5.415	0.096	6.132E-08	0.056				2	2	2	7.300E-09	0.370	Artery_Coronary

gene	gene_name	zscore	effect_size	pvalue	var_g	pred_pe	pred_p	pred_pe	n_snps_used	n_snps_in_cov	n_snps_in_model	best_gwas_p	largest_weight	Tissue
ENSG00000178053.17	MLF1	-5.411	-0.152	6.275E-08	0.025				2	2	2	1.500E-07	0.193	Whole_Blood
ENSG00000169314.14	C22orf15	-5.391	-0.245	7.000E-08	0.010				1	1	1	7.000E-08	0.189	Whole_Blood
ENSG00000257595.2	RP3-473L9.4	-5.388	-0.224	7.121E-08	0.011				2	2	2	7.600E-08	0.184	Adipose_Subcutaneou
ENSG00000180228.12	PRKRA	5.384	1.079	7.300E-08	0.000				1	1	1	7.300E-08	0.044	Heart_Atrial_Appendag
ENSG00000228672.3	PROB1	-5.376	-0.141	7.600E-08	0.034				1	2	2	7.600E-08	0.292	Adipose_Subcutaneou
ENSG00000228672.3	PROB1	-5.376	-0.122	7.600E-08	0.044				1	1	1	7.600E-08	0.338	Artery_Aorta
ENSG00000228672.3	PROB1	-5.376	-0.095	7.600E-08	0.075				1	1	1	7.600E-08	0.435	Artery_Tibial
ENSG00000228672.3	PROB1	-5.376	-0.138	7.600E-08	0.036				1	1	1	7.600E-08	0.300	Artery_Coronary
ENSG00000228672.3	PROB1	-5.376	-0.304	7.600E-08	0.007				1	1	1	7.600E-08	0.136	Heart_Atrial_Appendag
ENSG00000111252.10	SH2B3	-5.367	-4.528	8.000E-08	0.000				1	1	1	8.000E-08	0.009	Artery_Aorta
ENSG00000111252.10	SH2B3	5.367	3.029	8.000E-08	0.000				1	1	1	8.000E-08	0.013	Whole_Blood
ENSG00000111252.10	SH2B3	-5.367	-3.835	8.000E-08	0.000				1	1	1	8.000E-08	0.010	Artery_Tibial
ENSG00000111252.10	SH2B3	5.367	6.082	8.000E-08	0.000				1	1	1	8.000E-08	0.006	Adipose_Subcutaneou
ENSG00000111252.10	SH2B3	5.367	9.181	8.000E-08	0.000				1	1	1	8.000E-08	0.004	Liver
ENSG00000111252.10	SH2B3	-5.367	-4.846	8.000E-08	0.000				1	1	1	8.000E-08	0.008	Artery_Coronary
ENSG00000111252.10	SH2B3	-5.367	-72.800	8.000E-08	0.000				1	1	1	8.000E-08	0.001	Heart_Atrial_Appendag
ENSG00000196141.13	SPATS2L	5.344	0.086	9.087E-08	0.081				4	4	4	3.600E-07	0.331	Heart_Atrial_Appendag
ENSG00000180228.12	PRKRA	-5.339	-1.406	9.340E-08	0.000				2	2	2	7.300E-08	0.034	Artery_Tibial
ENSG00000128596.16	CCDC136	-5.332	-0.176	9.700E-08	0.018				2	2	2	1.000E-07	0.216	Heart_Atrial_Appendag
ENSG00000262655.3	SPON1	5.323	0.372	1.020E-07	0.005				2	2	2	6.600E-08	0.094	Adipose_Subcutaneou
ENSG00000128218.7	VPREB3	5.309	0.074	1.101E-07	0.110				2	2	2	3.000E-08	0.501	Artery_Tibial
ENSG00000079150.17	FKBP7	5.300	0.141	1.159E-07	0.029				2	2	2	1.600E-07	0.326	Adipose_Subcutaneou
ENSG00000128218.7	VPREB3	5.279	0.047	1.302E-07	0.249				3	3	3	5.600E-08	0.554	Adipose_Subcutaneou
ENSG00000228672.3	PROB1	-5.258	-0.284	1.457E-07	0.008				2	2	2	7.600E-08	0.139	Heart_Left_Ventricle
ENSG00000257595.2	RP3-473L9.4	-5.255	-0.175	1.479E-07	0.014				2	2	2	3.200E-08	0.223	Adipose_Visceral_Om
ENSG00000176371.13	ZSCAN2	-5.253	-0.469	1.500E-07	0.003				1	1	1	1.500E-07	0.081	Whole_Blood
ENSG00000176371.13	ZSCAN2	-5.253	-0.450	1.500E-07	0.003				1	1	1	1.500E-07	0.085	Liver
ENSG00000178053.17	MLF1	-5.253	-0.502	1.500E-07	0.002				1	1	1	1.500E-07	0.067	Kidney_Cortex
ENSG00000178053.17	MLF1	-5.253	-0.165	1.500E-07	0.020				1	1	1	1.500E-07	0.202	Artery_Coronary
ENSG00000257595.2	RP3-473L9.4	-5.252	-0.379	1.501E-07	0.004				2	2	2	3.100E-07	0.107	Artery_Aorta
ENSG00000079150.17	FKBP7	5.241	0.179	1.600E-07	0.017				1	1	1	1.600E-07	0.261	Whole_Blood
ENSG00000257595.2	RP3-473L9.4	-5.233	-0.344	1.669E-07	0.004				2	2	2	3.100E-07	0.117	Artery_Coronary
ENSG00000170476.15	MZB1	-5.229	-1.357	1.700E-07	0.000				1	1	1	1.700E-07	0.027	Heart_Left_Ventricle
ENSG00000228672.3	PROB1	-5.229	-0.149	1.700E-07	0.027				1	1	1	1.700E-07	0.244	Whole_Blood
ENSG00000136383.6	ALPK3	-5.196	-0.289	2.041E-07	0.006				2	2	2	6.200E-08	0.109	Heart_Left_Ventricle
ENSG00000135341.17	MAP3K7	-5.190	-0.217	2.100E-07	0.010				1	1	1	2.100E-07	0.147	Adipose_Subcutaneou
ENSG00000135341.17	MAP3K7	-5.190	-0.143	2.100E-07	0.023				1	1	1	2.100E-07	0.222	Liver

gene	gene_name	zscore	effect_size	pvalue	var_g	pred_pe	pred_p	pred_pe	n_snps_used	n_snps_in_cov	n_snps_in_model	best_gwas_p	largest_weight	Tissue
ENSG00000135341.17	MAP3K7	-5.190	-0.196	2.100E-07	0.014				1	1	1	2.100E-07	0.162	Kidney_Cortex
ENSG00000257595.2	RP3-473L9.4	-5.189	-0.351	2.120E-07	0.005				2	2	2	3.100E-07	0.113	Heart_Atrial_Appendag
ENSG00000247596.8	TWF2	-5.188	-3.233	2.130E-07	0.000				2	2	2	6.900E-04	0.027	Liver
ENSG00000108924.13	HLF	-5.182	-0.339	2.196E-07	0.005				2	2	2	2.500E-07	0.114	Heart_Left_Ventricle
ENSG00000128218.7	VPREB3	5.179	0.076	2.228E-07	0.101				2	2	2	5.600E-08	0.476	Liver
ENSG00000079150.17	FKBP7	5.175	0.079	2.276E-07	0.087				2	2	2	4.300E-11	0.505	Artery_Aorta
ENSG00000197696.9	NMB	5.170	0.282	2.343E-07	0.008				2	2	2	1.800E-09	0.119	Adipose_Subcutaneou
ENSG00000065526.10	SPEN	5.168	0.740	2.369E-07	0.001				2	2	2	1.200E-08	0.068	Whole_Blood
ENSG00000007062.11	PROM1	5.159	0.061	2.477E-07	0.168				3	3	3	8.400E-06	0.461	Heart_Left_Ventricle
ENSG00000093167.17	LRRFIP2	5.147	0.380	2.651E-07	0.004				3	3	3	2.900E-06	0.089	Artery_Coronary
ENSG00000178053.17	MLF1	-5.125	-0.136	2.968E-07	0.027				2	2	2	1.500E-07	0.246	Artery_Aorta
ENSG00000272087.1	RP11-379F4.7	-5.123	-0.168	3.000E-07	0.017				1	1	1	3.000E-07	0.193	Adipose_Visceral_Om
ENSG00000257595.2	RP3-473L9.4	-5.117	-0.342	3.100E-07	0.005				1	1	1	3.100E-07	0.114	Whole_Blood
ENSG00000257595.2	RP3-473L9.4	-5.117	-0.454	3.100E-07	0.003				1	1	1	3.100E-07	0.086	Artery_Tibial
ENSG00000257595.2	RP3-473L9.4	-5.117	-0.305	3.100E-07	0.005				1	1	1	3.100E-07	0.127	Kidney_Cortex
ENSG00000249816.6	LINC00964	-5.113	-0.090	3.169E-07	0.056				3	3	3	6.000E-18	0.216	Heart_Atrial_Appendag
ENSG00000170482.16	SLC23A1	-5.094	-0.141	3.500E-07	0.028				1	1	1	3.500E-07	0.265	Heart_Left_Ventricle
ENSG00000170482.16	SLC23A1	-5.094	-0.409	3.500E-07	0.003				1	1	1	3.500E-07	0.092	Artery_Aorta
ENSG00000170482.16	SLC23A1	-5.094	-1.188	3.500E-07	0.000				1	1	1	3.500E-07	0.032	Artery_Coronary
ENSG00000170482.16	SLC23A1	-5.094	-0.975	3.500E-07	0.001				1	1	1	3.500E-07	0.038	Heart_Atrial_Appendag
ENSG00000169762.16	TAPT1	5.092	0.245	3.541E-07	0.009				3	3	3	3.200E-07	0.150	Artery_Tibial
ENSG00000007062.11	PROM1	5.089	0.050	3.600E-07	0.224				1	1	1	3.600E-07	0.811	Artery_Coronary
ENSG00000272899.3	RP11-309L24.4	5.086	0.210	3.652E-07	0.011				2	2	2	3.500E-09	0.212	Artery_Coronary

Supplementary Table 1D: Left venticular-end diastolic volume (LVEDV)

gene	gene_name	zscore	effect_size	pvalue	var_g	pred_p	pred_p	pred_p	n_snps_used	n_snps_in_cov	n_snps_in_model	best_gwas_p	largest_weight	Tissue
ENSG00000271401.1	RP11-171I2.3	-7.356	-0.929	1.900E-13	0.001				1	1	1	1.900E-13	0.065	Adipose_Visceral_Om
ENSG00000185800.11	DMWD	-6.573	-0.206	4.925E-11	0.022				2	2	2	2.800E-11	0.196	Adipose_Visceral_Om
ENSG00000125755.18	SYMPK	-6.551	-0.680	5.700E-11	0.002				1	2	2	5.700E-11	0.066	Heart_Left_Ventricle
ENSG00000176182.5	MYPOP	6.551	2.603	5.700E-11	0.000				1	1	1	5.700E-11	0.017	Adipose_Visceral_Om
ENSG00000170608.2	FOXA3	6.551	279.358	5.700E-11	0.000				1	1	1	5.700E-11	0.000	Adipose_Subcutaneou
ENSG00000185800.11	DMWD	-6.341	-0.379	2.286E-10	0.006				2	2	2	7.800E-11	0.120	Heart_Left_Ventricle
ENSG00000079150.17	FKBP7	6.300	0.141	2.982E-10	0.041				2	2	2	1.800E-11	0.341	Heart_Left_Ventricle
ENSG00000177051.5	FBXO46	6.288	0.617	3.216E-10	0.002				2	2	2	2.600E-09	0.117	Artery_Tibial
ENSG00000237452.2	BHMG1	-5.955	-23.668	2.600E-09	0.000				1	1	1	2.600E-09	0.002	Heart_Atrial_Appendag
ENSG00000177051.5	FBXO46	5.955	0.728	2.600E-09	0.001				1	1	1	2.600E-09	0.058	Artery_Coronary
ENSG00000177051.5	FBXO46	5.955	0.737	2.600E-09	0.001				1	1	1	2.600E-09	0.057	Adipose_Subcutaneou
ENSG00000177051.5	FBXO46	5.955	0.646	2.600E-09	0.002				1	1	1	2.600E-09	0.065	Artery_Aorta
ENSG00000177051.5	FBXO46	5.955	0.680	2.600E-09	0.002				1	1	1	2.600E-09	0.062	Adipose_Visceral_Om
ENSG00000087077.13	TRIP6	5.784	0.155	7.290E-09	0.029				3	3	3	1.000E-07	0.155	Artery_Tibial
ENSG00000111252.10	SH2B3	-5.739	-4.306	9.500E-09	0.000				1	1	1	9.500E-09	0.010	Artery_Tibial
ENSG00000111252.10	SH2B3	5.739	3.401	9.500E-09	0.000				1	1	1	9.500E-09	0.013	Whole_Blood
ENSG00000111252.10	SH2B3	-5.739	-5.085	9.500E-09	0.000				1	1	1	9.500E-09	0.009	Artery_Aorta
ENSG00000111252.10	SH2B3	-5.739	-5.441	9.500E-09	0.000				1	1	1	9.500E-09	0.008	Artery_Coronary
ENSG00000111252.10	SH2B3	5.739	10.309	9.500E-09	0.000				1	1	1	9.500E-09	0.004	Liver
ENSG00000111252.10	SH2B3	-5.739	-81.743	9.500E-09	0.000				1	1	1	9.500E-09	0.001	Heart_Atrial_Appendag
ENSG00000111252.10	SH2B3	5.739	6.829	9.500E-09	0.000				1	1	1	9.500E-09	0.006	Adipose_Subcutaneou
ENSG00000079150.17	FKBP7	5.733	0.156	9.884E-09	0.029				2	2	2	3.900E-08	0.326	Adipose_Subcutaneou
ENSG00000079150.17	FKBP7	5.679	0.102	1.357E-08	0.063				2	2	2	1.400E-08	0.459	Artery_Tibial
ENSG00000079150.17	FKBP7	5.673	0.173	1.400E-08	0.021				1	1	1	1.400E-08	0.276	Heart_Atrial_Appendag
ENSG00000247596.8	TWF2	-5.670	-3.621	1.427E-08	0.000				2	2	2	5.800E-05	0.027	Liver
ENSG00000151923.17	TIAL1	-5.656	-0.495	1.552E-08	0.003				2	2	2	2.700E-08	0.075	Artery_Tibial
ENSG00000145020.15	AMT	5.650	0.117	1.600E-08	0.049				2	2	2	5.600E-08	0.263	Kidney_Cortex
ENSG00000079150.17	FKBP7	5.638	0.166	1.722E-08	0.024				2	2	2	1.400E-08	0.278	Adipose_Visceral_Om
ENSG00000145029.13	NICN1	5.622	0.210	1.892E-08	0.015				2	2	2	5.400E-08	0.157	Heart_Left_Ventricle
ENSG00000145029.13	NICN1	5.602	0.116	2.117E-08	0.051				2	2	2	5.400E-08	0.307	Adipose_Subcutaneou
ENSG00000145020.15	AMT	5.584	0.080	2.352E-08	0.105				2	2	2	5.100E-08	0.407	Adipose_Subcutaneou

gene	gene_name	zscore	effect_size	pvalue	var_g	pred_p	pred_p	pred_p	n_snps_used	n_snps_in_cov	n_snps_in_model	best_gwas_p	largest_weight	Tissue
ENSG00000145020.15	AMT	5.563	0.072	2.656E-08	0.130				2	2	2	5.600E-08	0.458	Artery_Aorta
ENSG00000145029.13	NICN1	5.559	0.119	2.713E-08	0.047				2	2	2	5.400E-08	0.295	Adipose_Visceral_Om
ENSG00000079150.17	FKBP7	5.537	0.099	3.069E-08	0.060				2	2	2	1.400E-08	0.417	Artery_Coronary
ENSG00000087085.13	ACHE	5.527	0.053	3.257E-08	0.232				3	3	3	3.400E-07	0.597	Heart_Atrial_Appendag
ENSG00000111860.13	CEP85L	-5.520	-0.168	3.399E-08	0.026				3	3	3	4.300E-08	0.151	Artery_Aorta
ENSG00000079150.17	FKBP7	5.495	0.191	3.900E-08	0.017				1	1	1	3.900E-08	0.261	Whole_Blood
ENSG00000145022.4	TCTA	5.486	0.369	4.100E-08	0.005				1	1	1	4.100E-08	0.111	Artery_Tibial
ENSG00000180228.12	PRKRA	5.466	1.117	4.600E-08	0.000				1	1	1	4.600E-08	0.044	Heart_Atrial_Appendag
ENSG00000087077.13	TRIP6	5.461	0.077	4.741E-08	0.109				3	3	3	1.000E-07	0.502	Heart_Atrial_Appendag
ENSG00000233276.3	GPX1	-5.451	-0.371	5.000E-08	0.005				1	1	1	5.000E-08	0.110	Artery_Aorta
ENSG00000233276.3	GPX1	-5.451	-0.486	5.000E-08	0.003				1	1	1	5.000E-08	0.084	Heart_Atrial_Appendag
ENSG00000233276.3	GPX1	-5.451	-0.904	5.000E-08	0.001				1	1	1	5.000E-08	0.045	Adipose_Visceral_Om
ENSG00000233276.3	GPX1	-5.451	-0.579	5.000E-08	0.002				1	1	1	5.000E-08	0.071	Artery_Tibial
ENSG00000233276.3	GPX1	-5.451	-1.450	5.000E-08	0.000				1	1	1	5.000E-08	0.028	Adipose_Subcutaneou
ENSG00000173402.11	DAG1	5.448	1.055	5.100E-08	0.001				1	1	1	5.100E-08	0.039	Heart_Left_Ventricle
ENSG00000145020.15	AMT	5.448	0.889	5.100E-08	0.001				1	1	1	5.100E-08	0.046	Liver
ENSG00000173402.11	DAG1	5.448	1.090	5.100E-08	0.001				1	1	1	5.100E-08	0.037	Artery_Tibial
ENSG00000145029.13	NICN1	5.438	0.187	5.400E-08	0.019				1	1	1	5.400E-08	0.217	Kidney_Cortex
ENSG00000145029.13	NICN1	5.438	1.380	5.400E-08	0.000				1	1	1	5.400E-08	0.030	Whole_Blood
ENSG00000145029.13	NICN1	5.438	0.168	5.400E-08	0.025				1	1	1	5.400E-08	0.242	Artery_Coronary
ENSG00000087085.13	ACHE	5.433	0.029	5.551E-08	0.799				2	2	2	3.400E-07	1.007	Artery_Aorta
ENSG00000145020.15	AMT	5.431	0.109	5.600E-08	0.059				1	1	1	5.600E-08	0.371	Artery_Coronary
ENSG00000145020.15	AMT	5.431	0.110	5.600E-08	0.054				1	1	1	5.600E-08	0.369	Adipose_Visceral_Om
ENSG00000145020.15	AMT	5.431	0.107	5.600E-08	0.057				1	1	1	5.600E-08	0.380	Whole_Blood
ENSG00000145022.4	TCTA	5.414	0.376	6.175E-08	0.005				2	2	2	4.100E-08	0.100	Adipose_Visceral_Om
ENSG00000180228.12	PRKRA	-5.402	-1.451	6.572E-08	0.000				2	2	2	4.600E-08	0.034	Artery_Tibial
ENSG00000196141.13	SPATS2L	5.395	0.090	6.863E-08	0.081				4	4	4	9.700E-08	0.331	Heart_Atrial_Appendag
ENSG00000114316.12	USP4	-5.391	-0.479	7.000E-08	0.003				1	1	1	7.000E-08	0.084	Artery_Coronary
ENSG00000114316.12	USP4	-5.391	-0.334	7.000E-08	0.006				1	1	1	7.000E-08	0.121	Heart_Left_Ventricle
ENSG00000114316.12	USP4	-5.391	-0.344	7.000E-08	0.006				1	1	1	7.000E-08	0.118	Heart_Atrial_Appendag
ENSG00000114316.12	USP4	-5.391	-0.464	7.000E-08	0.003				1	1	1	7.000E-08	0.087	Artery_Tibial
ENSG00000114316.12	USP4	-5.391	-0.381	7.000E-08	0.005				1	1	1	7.000E-08	0.106	Liver
ENSG00000114316.12	USP4	-5.391	-0.290	7.000E-08	0.008				1	1	1	7.000E-08	0.139	Artery_Aorta

gene	gene_name	zscore	effect_size	pvalue	var_g	pred_p	pred_p	pred_p	n_snps_used	n_snps_in_cov	n_snps_in_model	best_gwas_p	largest_weight	Tissue
ENSG00000114316.12	USP4	-5.391	-1.127	7.000E-08	0.001				1	1	1	7.000E-08	0.036	Whole_Blood
ENSG00000145022.4	TCTA	5.385	0.284	7.231E-08	0.008				2	2	2	4.100E-08	0.133	Adipose_Subcutaneou
ENSG00000198523.5	PLN	-5.381	-2.630	7.403E-08	0.000				2	2	2	7.600E-08	0.028	Heart_Atrial_Appendag
ENSG00000151923.17	TIAL1	-5.359	-0.377	8.352E-08	0.004				2	2	2	8.200E-08	0.092	Adipose_Subcutaneou
ENSG00000087087.18	SRRT	5.327	0.205	1.000E-07	0.013				1	1	1	1.000E-07	0.214	Heart_Atrial_Appendag
ENSG00000087087.18	SRRT	5.327	0.225	1.000E-07	0.011				1	1	1	1.000E-07	0.195	Artery_Coronary
ENSG00000087087.18	SRRT	5.327	0.309	1.000E-07	0.006				1	1	1	1.000E-07	0.142	Heart_Left_Ventricle
ENSG00000087087.18	SRRT	5.327	0.305	1.000E-07	0.006				1	1	1	1.000E-07	0.144	Kidney_Cortex
ENSG00000145022.4	TCTA	5.319	0.365	1.046E-07	0.005				2	2	2	4.100E-08	0.099	Artery_Coronary
ENSG00000145022.4	TCTA	5.309	0.794	1.102E-07	0.001				2	2	2	4.100E-08	0.055	Whole_Blood
ENSG00000196141.13	SPATS2L	5.307	0.186	1.113E-07	0.019				3	3	3	1.300E-05	0.277	Heart_Left_Ventricle
ENSG00000087085.13	ACHE	5.284	0.063	1.266E-07	0.154				2	2	2	3.300E-07	0.680	Artery_Tibial
ENSG00000145022.4	TCTA	5.276	0.509	1.317E-07	0.003				2	2	2	4.100E-08	0.097	Liver
ENSG00000179588.8	ZFPM1	5.271	0.193	1.355E-07	0.016				2	2	2	1.400E-07	0.189	Artery_Aorta
ENSG00000145022.4	TCTA	5.271	0.474	1.360E-07	0.003				2	2	2	4.100E-08	0.098	Kidney_Cortex
ENSG00000145020.15	AMT	5.265	0.180	1.403E-07	0.017				2	2	2	5.100E-08	0.117	Heart_Left_Ventricle
ENSG00000116095.10	PLEKHA3	5.237	0.355	1.630E-07	0.006				2	2	2	3.700E-06	0.074	Liver
ENSG00000151923.17	TIAL1	5.229	1.921	1.700E-07	0.000				1	1	1	1.700E-07	0.022	Whole_Blood
ENSG00000070759.16	TESK2	5.229	0.443	1.705E-07	0.003				2	2	2	2.300E-07	0.080	Artery_Aorta
ENSG00000198218.10	QRICH1	-5.223	-0.233	1.758E-07	0.013				2	2	2	3.800E-06	0.201	Whole_Blood
ENSG00000173064.12	HECTD4	5.222	0.458	1.770E-07	0.002				2	2	2	8.200E-07	0.095	Artery_Aorta
ENSG00000178053.17	MLF1	-5.212	-0.063	1.869E-07	0.142				3	3	3	1.200E-05	0.330	Heart_Left_Ventricle
ENSG00000173064.12	HECTD4	5.207	0.456	1.922E-07	0.002				2	2	2	8.200E-07	0.095	Adipose_Visceral_Om
ENSG00000173421.16	CCDC36	5.204	0.032	1.954E-07	0.588				2	2	2	7.300E-07	0.697	Artery_Aorta
ENSG00000198218.10	QRICH1	-5.192	-0.126	2.086E-07	0.041				2	2	2	2.700E-06	0.317	Adipose_Subcutaneou
ENSG00000177045.7	SIX5	5.143	0.435	2.700E-07	0.003				1	1	1	2.700E-07	0.076	Adipose_Visceral_Om
ENSG00000198218.10	QRICH1	-5.138	-0.225	2.778E-07	0.013				2	2	2	2.700E-06	0.174	Artery_Tibial
ENSG00000185800.11	DMWD	-5.111	-0.221	3.200E-07	0.012				1	1	1	3.200E-07	0.149	Liver
ENSG00000185800.11	DMWD	-5.111	-0.174	3.200E-07	0.018				1	1	1	3.200E-07	0.190	Adipose_Subcutaneou
ENSG00000087085.13	ACHE	5.100	0.104	3.400E-07	0.051				1	1	1	3.400E-07	0.414	Adipose_Visceral_Om
ENSG00000087085.13	ACHE	5.100	0.804	3.400E-07	0.001				1	1	1	3.400E-07	0.054	Whole_Blood
ENSG00000087085.13	ACHE	5.100	0.162	3.400E-07	0.022				1	1	1	3.400E-07	0.267	Kidney_Cortex
ENSG00000087085.13	ACHE	5.100	0.064	3.400E-07	0.126				1	1	1	3.400E-07	0.673	Artery_Coronary
ENSG00000177045.7	SIX5	5.084	0.383	3.700E-07	0.004				1	1	1	3.700E-07	0.085	Artery_Tibial
ENSG00000146830.9	GIGYF1	5.082	0.111	3.734E-07	0.043				2	2	2	1.600E-05	0.331	Adipose_Visceral_Om

Supplementary Table 1E: All-cause heart failure in a multi-ancestry population														
gene	gene_name	zscore	effect_size	pvalue	var_g	pred_	pred_	pred_	n_snps_used	n_snps_in_cov	n_snps_in_model	best_gwas_p	largest_weight	Tissue
ENSG00000124762.13	CDKN1A	15.071	2.358	2.521E-51	0.000				1	1	1	2.521E-51	0.028	Heart_Left_Ventricle
ENSG00000147883.10	CDKN2B	12.097	0.683	1.094E-33	0.002				2	2	2	3.577E-34	0.061	Artery_Aorta
ENSG00000177791.11	MYOZ1	-10.717	-0.035	8.461E-27	0.509				2	2	2	1.447E-26	1.044	Heart_Atrial_Appendag
ENSG00000166317.11	SYNPO2L	10.235	0.979	1.388E-24	0.000				2	2	2	1.780E-24	0.048	Heart_Left_Ventricle
ENSG00000166317.11	SYNPO2L	10.234	0.558	1.400E-24	0.002				1	1	1	1.400E-24	0.083	Heart_Atrial_Appendag
ENSG00000166317.11	SYNPO2L	-10.185	-0.277	2.311E-24	0.007				2	2	2	1.400E-24	0.166	Artery_Tibial
ENSG00000196968.10	FUT11	9.925	0.186	3.226E-23	0.021				2	2	2	1.913E-22	0.160	Whole_Blood
ENSG00000196968.10	FUT11	9.880	0.083	5.108E-23	0.085				2	2	2	5.219E-21	0.326	Adipose_Visceral_Om
ENSG00000196968.10	FUT11	9.879	0.063	5.143E-23	0.144				2	2	2	7.496E-21	0.410	Adipose_Subcutaneou
ENSG00000176986.15	SEC24C	-9.542	-0.748	1.399E-21	0.001				2	2	2	4.210E-21	0.034	Artery_Aorta
ENSG00000172650.13	AGAP5	-9.436	-0.440	3.857E-21	0.003				1	1	1	3.857E-21	0.114	Artery_Aorta
ENSG00000176986.15	SEC24C	9.427	2.374	4.210E-21	0.000				1	1	1	4.210E-21	0.021	Kidney_Cortex
ENSG00000196968.10	FUT11	9.367	0.127	7.496E-21	0.028				1	1	1	7.496E-21	0.380	Artery_Coronary
ENSG00000196968.10	FUT11	9.367	0.191	7.496E-21	0.009				1	1	1	7.496E-21	0.254	Kidney_Cortex
ENSG00000186510.11	CLCNKA	-9.268	-0.169	1.889E-20	0.019				1	1	1	1.889E-20	0.216	Kidney_Cortex
ENSG00000176986.15	SEC24C	-9.240	-0.972	2.463E-20	0.001				1	1	1	2.463E-20	0.051	Artery_Tibial
ENSG00000176986.15	SEC24C	-9.240	-4.760	2.463E-20	0.000				1	1	1	2.463E-20	0.010	Whole_Blood
ENSG00000176986.15	SEC24C	-9.174	-1.024	4.565E-20	0.000				2	2	2	2.463E-20	0.027	Heart_Left_Ventricle
ENSG00000176986.15	SEC24C	-9.077	-0.908	1.112E-19	0.001				2	2	2	2.463E-20	0.030	Artery_Coronary
ENSG00000124762.13	CDKN1A	-8.848	-1.959	8.890E-19	0.000				2	2	2	2.514E-35	0.018	Artery_Tibial
ENSG00000115808.11	STRN	8.436	0.277	3.278E-17	0.005				2	2	2	4.249E-17	0.134	Artery_Aorta
ENSG00000172650.13	AGAP5	-8.254	-0.076	1.533E-16	0.077				2	2	2	2.008E-15	0.222	Heart_Atrial_Appendag
ENSG00000124574.14	ABCC10	-8.054	-0.579	8.037E-16	0.001				1	1	1	8.037E-16	0.053	Heart_Left_Ventricle
ENSG00000172650.13	AGAP5	-8.018	-0.279	1.071E-15	0.007				1	1	1	1.071E-15	0.126	Artery_Tibial
ENSG00000196968.10	FUT11	7.980	0.118	1.461E-15	0.029				1	1	1	1.461E-15	0.258	Artery_Aorta
ENSG00000172650.13	AGAP5	-7.941	-0.186	2.008E-15	0.012				1	1	1	2.008E-15	0.163	Adipose_Subcutaneou
ENSG00000172650.13	AGAP5	-7.916	-0.151	2.450E-15	0.021				2	2	2	1.071E-15	0.142	Whole_Blood
ENSG00000203356.2	LINC01562	7.744	0.102	9.612E-15	0.024				1	1	1	9.612E-15	0.950	Artery_Tibial
ENSG00000196968.10	FUT11	7.735	0.153	1.035E-14	0.016				1	1	1	1.035E-14	0.189	Artery_Tibial
ENSG00000186510.11	CLCNKA	-7.706	-0.070	1.302E-14	0.088				1	1	1	1.302E-14	0.399	Artery_Coronary
ENSG00000186510.11	CLCNKA	-7.706	-0.060	1.302E-14	0.110				1	1	1	1.302E-14	0.467	Artery_Aorta

gene	gene_name	zscore	effect_size	pvalue	var_g	pred_	pred_	pred_	n_snps_used	n_snps_in_cov	n_snps_in_model	best_gwas_p	largest_weight	Tissue
ENSG00000146243.13	IRAK1BP1	7.654	0.037	1.948E-14	0.263				3	3	3	6.377E-14	0.402	Adipose_Subcutaneou
ENSG00000196968.10	FUT11	7.631	0.089	2.326E-14	0.047				1	1	1	2.326E-14	0.322	Heart_Left_Ventricle
ENSG00000196968.10	FUT11	7.631	0.095	2.326E-14	0.041				1	1	1	2.326E-14	0.300	Heart_Atrial_Appendag
ENSG00000272140.2	RP11-574K11.29	7.617	0.463	2.605E-14	0.002				1	1	1	2.605E-14	0.062	Artery_Coronary
ENSG00000272140.2	RP11-574K11.29	7.617	0.397	2.605E-14	0.002				1	2	2	2.605E-14	0.072	Heart_Left_Ventricle
ENSG00000272140.2	RP11-574K11.29	7.617	0.244	2.605E-14	0.006				1	2	2	2.605E-14	0.118	Adipose_Visceral_Om
ENSG00000272140.2	RP11-574K11.29	7.617	0.201	2.605E-14	0.008				1	2	2	2.605E-14	0.143	Kidney_Cortex
ENSG00000272140.2	RP11-574K11.29	7.617	0.283	2.605E-14	0.005				1	2	2	2.605E-14	0.101	Adipose_Subcutaneou
ENSG00000272140.2	RP11-574K11.29	7.617	0.296	2.605E-14	0.004				1	2	2	2.605E-14	0.097	Whole_Blood
ENSG00000172650.13	AGAP5	-7.563	-0.205	3.926E-14	0.012				1	1	1	3.926E-14	0.164	Heart_Left_Ventricle
ENSG00000172650.13	AGAP5	-7.563	-0.265	3.926E-14	0.007				1	1	1	3.926E-14	0.127	Adipose_Visceral_Om
ENSG00000172650.13	AGAP5	-7.563	-0.149	3.926E-14	0.022				1	1	1	3.926E-14	0.226	Liver
ENSG00000141452.9	C18orf8	-7.554	-0.092	4.227E-14	0.043				3	3	3	2.495E-13	0.166	Whole_Blood
ENSG00000134222.16	PSRC1	-7.541	-0.029	4.657E-14	0.412				1	1	1	4.657E-14	1.099	Liver
ENSG00000122861.15	PLAU	-7.530	-0.134	5.084E-14	0.028				2	2	2	8.906E-16	0.448	Heart_Atrial_Appendag
ENSG00000115808.11	STRN	-7.489	-0.140	6.944E-14	0.016				1	1	1	6.944E-14	0.184	Heart_Atrial_Appendag
ENSG00000141452.9	C18orf8	-7.488	-0.131	6.976E-14	0.021				2	2	2	2.495E-13	0.137	Heart_Left_Ventricle
ENSG00000141452.9	C18orf8	-7.478	-0.095	7.545E-14	0.041				2	2	2	2.495E-13	0.193	Artery_Tibial
ENSG00000141452.9	C18orf8	-7.454	-0.062	9.041E-14	0.097				3	3	3	2.495E-13	0.247	Heart_Atrial_Appendag
ENSG00000141736.13	ERBB2	7.450	0.525	9.354E-14	0.001				2	2	2	6.468E-14	0.052	Adipose_Subcutaneou
ENSG00000115808.11	STRN	-7.415	-0.202	1.213E-13	0.008				1	1	1	1.213E-13	0.128	Heart_Left_Ventricle
ENSG00000136379.11	ABHD17C	7.389	0.125	1.475E-13	0.024				2	2	2	1.504E-13	0.220	Heart_Atrial_Appendag
ENSG00000177791.11	MYOZ1	-7.377	-0.063	1.622E-13	0.062				2	2	2	1.647E-13	0.614	Heart_Left_Ventricle
ENSG00000176986.15	SEC24C	-7.375	-0.369	1.640E-13	0.003				1	1	1	1.640E-13	0.077	Adipose_Subcutaneou
ENSG00000141736.13	ERBB2	7.339	0.563	2.149E-13	0.001				2	2	2	6.468E-14	0.047	Adipose_Visceral_Om
ENSG00000141452.9	C18orf8	-7.334	-0.068	2.240E-13	0.078				3	3	3	2.495E-13	0.239	Adipose_Visceral_Om
ENSG00000204277.1	LINC01993	7.333	0.080	2.257E-13	0.058				3	3	3	2.273E-13	0.327	Kidney_Cortex
ENSG00000141452.9	C18orf8	-7.314	-0.093	2.595E-13	0.042				2	2	2	2.495E-13	0.177	Artery_Coronary
ENSG00000141458.12	NPC1	-7.307	-0.098	2.725E-13	0.036				2	2	2	2.509E-13	0.293	Artery_Aorta
ENSG00000203356.2	LINC01562	7.298	0.077	2.914E-13	0.014				2	2	2	3.651E-13	0.891	Artery_Aorta
ENSG00000141452.9	C18orf8	-7.279	-0.052	3.352E-13	0.134				3	3	3	2.495E-13	0.291	Adipose_Subcutaneou
ENSG00000203356.2	LINC01562	7.268	0.112	3.651E-13	0.011				1	1	1	3.651E-13	0.605	Adipose_Subcutaneou
ENSG00000123091.4	RNF11	-7.268	-5.180	3.651E-13	0.000				1	1	1	3.651E-13	0.013	Adipose_Subcutaneou

gene	gene_name	zscore	effect_size	pvalue	var_g	pred_	pred_	pred_	n_snps_used	n_snps_in_cov	n_snps_in_model	best_gwas_p	largest_weight	Tissue
ENSG00000141736.13	ERBB2	7.238	1.327	4.570E-13	0.000				2	2	2	6.468E-14	0.019	Whole_Blood
ENSG00000141452.9	C18orf8	-7.224	-0.147	5.059E-13	0.016				2	2	2	2.495E-13	0.184	Artery_Aorta
ENSG00000108306.11	FBXL20	-7.219	-0.189	5.241E-13	0.009				2	2	2	7.543E-13	0.161	Adipose_Subcutaneou
ENSG00000005007.12	UPF1	7.219	0.401	5.242E-13	0.002				2	2	2	5.316E-13	0.066	Artery_Tibial
ENSG00000161395.13	PGAP3	7.214	0.406	5.415E-13	0.002				1	2	2	5.415E-13	0.069	Whole_Blood
ENSG00000105974.11	CAV1	-7.186	-0.541	6.676E-13	0.001				3	3	3	1.347E-12	0.045	Heart_Atrial_Appendag
ENSG00000108306.11	FBXL20	-7.145	-0.203	8.981E-13	0.007				2	2	2	1.184E-12	0.149	Heart_Left_Ventricle
ENSG00000141741.11	MIEN1	-7.134	-2.604	9.728E-13	0.000				1	1	1	9.728E-13	0.010	Whole_Blood
ENSG00000108306.11	FBXL20	-7.117	-0.281	1.106E-12	0.004				2	2	2	1.184E-12	0.106	Adipose_Visceral_Om
ENSG00000108306.11	FBXL20	-7.108	-0.139	1.174E-12	0.016				2	2	2	1.184E-12	0.213	Artery_Aorta
ENSG00000141736.13	ERBB2	7.079	0.126	1.454E-12	0.020				3	3	3	6.468E-14	0.123	Heart_Left_Ventricle
ENSG00000108306.11	FBXL20	-7.071	-0.217	1.533E-12	0.007				2	2	2	1.184E-12	0.136	Heart_Atrial_Appendag
ENSG00000105662.15	CRTC1	7.048	0.182	1.817E-12	0.010				1	1	1	1.817E-12	0.139	Artery_Tibial
ENSG00000105662.15	CRTC1	7.048	0.231	1.817E-12	0.006				1	1	1	1.817E-12	0.109	Artery_Aorta
ENSG00000141736.13	ERBB2	7.033	0.233	2.019E-12	0.007				2	2	2	1.692E-12	0.113	Heart_Atrial_Appendag
ENSG00000146243.13	IRAK1BP1	7.024	0.056	2.151E-12	0.100				2	2	2	2.275E-12	0.448	Liver
ENSG00000108306.11	FBXL20	-7.016	-0.177	2.277E-12	0.013				2	2	2	3.366E-12	0.177	Artery_Coronary
ENSG00000125686.11	MED1	7.006	0.833	2.455E-12	0.000				2	2	2	3.777E-12	0.032	Heart_Left_Ventricle
ENSG00000125686.11	MED1	6.996	0.959	2.636E-12	0.000				2	2	2	3.777E-12	0.027	Heart_Atrial_Appendag
ENSG00000125686.11	MED1	6.985	1.009	2.858E-12	0.000				2	2	2	3.777E-12	0.026	Artery_Tibial
ENSG00000125686.11	MED1	6.966	0.580	3.254E-12	0.001				2	2	2	3.777E-12	0.042	Liver
ENSG00000146243.13	IRAK1BP1	6.966	0.060	3.272E-12	0.086				2	2	2	2.275E-12	0.391	Artery_Aorta
ENSG00000108306.11	FBXL20	-6.948	-0.163	3.697E-12	0.012				2	2	2	3.981E-12	0.178	Artery_Tibial
ENSG00000141458.12	NPC1	-6.935	-0.042	4.062E-12	0.194				2	2	2	2.509E-13	0.397	Heart_Atrial_Appendag
ENSG00000125686.11	MED1	6.927	0.754	4.284E-12	0.001				2	2	2	3.777E-12	0.033	Adipose_Visceral_Om
ENSG00000125686.11	MED1	6.925	0.611	4.366E-12	0.001				2	2	2	3.777E-12	0.041	Adipose_Subcutaneou
ENSG00000185519.8	FAM131C	6.915	1.571	4.678E-12	0.000				1	1	1	4.678E-12	0.024	Heart_Left_Ventricle
ENSG00000146243.13	IRAK1BP1	6.912	0.073	4.773E-12	0.058				3	3	3	2.275E-12	0.327	Adipose_Visceral_Om
ENSG00000099953.9	MMP11	6.912	0.085	4.789E-12	0.039				1	1	1	4.789E-12	0.341	Heart_Atrial_Appendag
ENSG00000085831.15	TTC39A	-6.912	-86.825	4.795E-12	0.000				1	1	1	4.795E-12	0.001	Heart_Atrial_Appendag
ENSG00000125686.11	MED1	6.901	0.610	5.157E-12	0.001				2	2	2	3.777E-12	0.039	Whole_Blood
ENSG00000161395.13	PGAP3	6.883	0.041	5.878E-12	0.176				2	2	2	5.415E-13	0.332	Heart_Left_Ventricle
ENSG00000185056.9	C5orf47	-6.866	-0.095	6.585E-12	0.037				1	1	1	6.585E-12	0.302	Heart_Atrial_Appendag
ENSG00000146247.13	PHIP	6.854	0.205	7.158E-12	0.007				1	1	1	7.158E-12	0.120	Heart_Left_Ventricle
ENSG00000119326.14	CTNNAL1	6.851	0.091	7.318E-12	0.038				3	3	3	6.852E-10	0.187	Heart_Atrial_Appendag
ENSG00000125686.11	MED1	6.842	1.236	7.802E-12	0.000				2	2	2	3.777E-12	0.020	Artery_Coronary
ENSG00000125686.11	MED1	6.842	0.566	7.824E-12	0.001				2	2	2	3.777E-12	0.039	Kidney_Cortex

gene	gene_name	zscore	effect_size	pvalue	var_g	pred_	pred_	pred_	n_snps_used	n_snps_in_cov	n_snps_in_model	best_gwas_p	largest_weight	Tissue
ENSG00000111785.19	RIC8B	6.841	0.259	7.839E-12	0.004				2	2	2	9.188E-12	0.097	Heart_Atrial_Appendag
ENSG00000077522.12	ACTN2	6.841	0.192	7.879E-12	0.007				2	2	2	1.303E-11	0.124	Heart_Atrial_Appendag
ENSG00000141458.12	NPC1	-6.838	-0.049	8.027E-12	0.140				3	3	3	2.509E-13	0.321	Artery_Coronary
ENSG00000146243.13	IRAK1BP1	6.821	0.058	9.047E-12	0.093				2	2	2	2.275E-12	0.362	Heart_Left_Ventricle
ENSG00000085831.15	TTC39A	-6.810	-2.741	9.759E-12	0.000				2	2	2	4.795E-12	0.026	Artery_Coronary
ENSG00000111785.19	RIC8B	6.807	0.294	9.946E-12	0.004				2	2	2	9.188E-12	0.083	Adipose_Visceral_Om
ENSG00000204387.12	C6orf48	6.796	0.137	1.075E-11	0.012				2	2	2	1.840E-13	0.219	Adipose_Visceral_Om
ENSG00000146247.13	PHIP	6.790	0.264	1.124E-11	0.004				1	1	1	1.124E-11	0.093	Heart_Atrial_Appendag
ENSG00000146247.13	PHIP	6.790	0.289	1.124E-11	0.004				1	1	1	1.124E-11	0.085	Adipose_Visceral_Om
ENSG00000146247.13	PHIP	6.790	0.249	1.124E-11	0.005				1	1	1	1.124E-11	0.099	Artery_Aorta
ENSG00000146247.13	PHIP	6.790	0.251	1.124E-11	0.004				1	1	1	1.124E-11	0.098	Adipose_Subcutaneou
ENSG00000146247.13	PHIP	6.790	0.341	1.124E-11	0.002				1	1	1	1.124E-11	0.072	Whole_Blood
ENSG00000145194.17	ECE2	6.776	0.064	1.236E-11	0.070				1	1	1	1.236E-11	0.376	Heart_Atrial_Appendag
ENSG00000122884.12	P4HA1	6.775	16.278	1.242E-11	0.000				1	1	1	1.242E-11	0.003	Adipose_Visceral_Om
ENSG00000122884.12	P4HA1	-6.775	-8.682	1.242E-11	0.000				1	1	1	1.242E-11	0.005	Heart_Left_Ventricle
ENSG00000122884.12	P4HA1	-6.775	-10.867	1.242E-11	0.000				1	1	1	1.242E-11	0.004	Artery_Tibial
ENSG00000122884.12	P4HA1	6.775	30.151	1.242E-11	0.000				1	1	1	1.242E-11	0.002	Liver
ENSG00000122884.12	P4HA1	-6.775	-9.756	1.242E-11	0.000				1	1	1	1.242E-11	0.005	Artery_Aorta
ENSG00000122884.12	P4HA1	-6.775	-12.546	1.242E-11	0.000				1	1	1	1.242E-11	0.004	Artery_Coronary
ENSG00000122884.12	P4HA1	-6.775	-3.821	1.242E-11	0.000				1	1	1	1.242E-11	0.012	Whole_Blood
ENSG00000122884.12	P4HA1	6.775	28.240	1.242E-11	0.000				1	1	1	1.242E-11	0.002	Adipose_Subcutaneou
ENSG00000122884.12	P4HA1	-6.775	-16.043	1.242E-11	0.000				1	1	1	1.242E-11	0.003	Heart_Atrial_Appendag
ENSG00000099953.9	MMP11	6.764	0.073	1.341E-11	0.049				2	2	2	4.789E-12	0.370	Heart_Left_Ventricle
ENSG00000141744.3	PNMT	6.714	0.130	1.894E-11	0.018				1	1	1	1.894E-11	0.200	Heart_Left_Ventricle
ENSG00000148926.9	ADM	-6.704	-1.987	2.029E-11	0.000				1	1	1	2.029E-11	0.019	Adipose_Visceral_Om
ENSG00000055483.19	USP36	-6.704	-0.076	2.033E-11	0.051				3	3	3	9.587E-11	0.313	Artery_Aorta
ENSG00000151135.9	TMEM263	6.687	0.670	2.279E-11	0.001				1	1	1	2.279E-11	0.037	Whole_Blood
ENSG00000151135.9	TMEM263	6.687	0.165	2.279E-11	0.011				1	1	1	2.279E-11	0.149	Artery_Coronary
ENSG00000146243.13	IRAK1BP1	6.683	0.061	2.346E-11	0.085				2	2	2	2.275E-12	0.382	Artery_Coronary
ENSG00000213719.8	CLIC1	-6.675	-343.128	2.469E-11	0.000				2	2	2	3.718E-11	0.000	Kidney_Cortex
ENSG00000141744.3	PNMT	6.673	0.128	2.513E-11	0.020				2	2	2	4.601E-11	0.194	Heart_Atrial_Appendag
ENSG00000146243.13	IRAK1BP1	6.638	0.066	3.180E-11	0.063				2	2	2	2.275E-12	0.350	Artery_Tibial
ENSG00000146215.13	CRIP3	-6.635	-0.165	3.250E-11	0.010				2	2	2	1.888E-07	0.147	Adipose_Subcutaneou
ENSG00000128591.15	FLNC	6.634	10.553	3.269E-11	0.000				1	1	1	3.269E-11	0.004	Artery_Coronary
ENSG00000128591.15	FLNC	6.634	0.793	3.269E-11	0.001				1	1	1	3.269E-11	0.057	Kidney_Cortex
ENSG00000128591.15	FLNC	-6.634	-1.635	3.269E-11	0.000				1	1	1	3.269E-11	0.028	Heart_Left_Ventricle
ENSG00000128591.15	FLNC	-6.634	-2.389	3.269E-11	0.000				1	1	1	3.269E-11	0.019	Whole_Blood

gene	gene_name	zscore	effect_size	pvalue	var_g	pred_	pred_	pred_	n_snps_used	n_snps_in_cov	n_snps_in_model	best_gwas_p	largest_weight	Tissue
ENSG00000146215.13	CRIP3	6.622	0.067	3.539E-11	0.066				2	2	2	3.298E-10	0.273	Liver
ENSG00000171634.16	BPTF	-6.622	-0.157	3.546E-11	0.011				2	2	2	7.330E-11	0.118	Heart_Atrial_Appendag
ENSG00000112137.17	PHACTR1	-6.599	-0.086	4.153E-11	0.037				1	1	1	4.153E-11	0.275	Artery_Coronary
ENSG00000112137.17	PHACTR1	-6.587	-0.076	4.485E-11	0.045				2	2	2	4.153E-11	0.312	Artery_Tibial
ENSG00000196141.13	SPATS2L	6.584	0.179	4.574E-11	0.009				2	2	2	5.227E-11	0.134	Adipose_Subcutaneou
ENSG00000141744.3	PNMT	6.583	0.148	4.601E-11	0.014				1	1	1	4.601E-11	0.170	Whole_Blood
ENSG00000119326.14	CTNNAL1	6.581	0.074	4.680E-11	0.058				3	3	3	6.852E-10	0.635	Liver
ENSG00000197696.9	NMB	-6.545	-0.073	5.962E-11	0.059				1	1	1	5.962E-11	0.375	Artery_Aorta
ENSG00000197696.9	NMB	-6.545	-0.111	5.962E-11	0.025				1	1	1	5.962E-11	0.246	Heart_Atrial_Appendag
ENSG00000197696.9	NMB	-6.545	-0.110	5.962E-11	0.025				1	1	1	5.962E-11	0.248	Heart_Left_Ventricle
ENSG00000197696.9	NMB	-6.545	-0.062	5.962E-11	0.076				1	1	1	5.962E-11	0.438	Liver
ENSG00000197696.9	NMB	-6.545	-0.103	5.962E-11	0.028				1	1	1	5.962E-11	0.266	Artery_Tibial
ENSG00000197696.9	NMB	-6.545	-0.118	5.962E-11	0.021				1	1	1	5.962E-11	0.231	Artery_Coronary
ENSG00000197696.9	NMB	-6.545	-0.173	5.962E-11	0.010				1	1	1	5.962E-11	0.158	Adipose_Visceral_Om
ENSG00000133805.15	AMPD3	-6.526	-15.370	6.770E-11	0.000				1	1	1	6.770E-11	0.002	Liver
ENSG00000134222.16	PSRC1	-6.521	-0.084	6.978E-11	0.034				3	3	3	1.287E-16	0.246	Heart_Left_Ventricle
ENSG00000146243.13	IRAK1BP1	6.519	0.061	7.086E-11	0.075				2	2	2	2.275E-12	0.367	Heart_Atrial_Appendag
ENSG00000112137.17	PHACTR1	-6.508	-0.085	7.592E-11	0.034				2	2	2	4.153E-11	0.272	Artery_Aorta
ENSG00000141744.3	PNMT	6.504	0.102	7.826E-11	0.028				2	2	2	4.601E-11	0.149	Adipose_Visceral_Om
ENSG00000161395.13	PGAP3	6.499	0.097	8.082E-11	0.029				2	2	2	5.415E-13	0.469	Artery_Coronary
ENSG00000171634.16	BPTF	-6.497	-0.214	8.184E-11	0.007				2	2	2	7.330E-11	0.076	Artery_Aorta
ENSG00000204498.10	NFKBIL1	6.491	0.220	8.552E-11	0.004				3	3	3	3.082E-07	0.224	Adipose_Subcutaneou
ENSG00000204438.10	GPANK1	-6.489	-0.210	8.650E-11	0.007				2	2	2	1.839E-07	0.095	Heart_Atrial_Appendag
ENSG00000108379.9	WNT3	6.481	0.062	9.136E-11	0.090				2	2	2	4.455E-09	0.392	Artery_Coronary
ENSG00000116809.11	ZBTB17	6.478	5.039	9.296E-11	0.000				1	1	1	9.296E-11	0.005	Artery_Aorta
ENSG00000116809.11	ZBTB17	-6.478	-191.316	9.296E-11	0.000				1	1	1	9.296E-11	0.000	Liver
ENSG00000116809.11	ZBTB17	6.478	6.664	9.296E-11	0.000				1	1	1	9.296E-11	0.004	Whole_Blood
ENSG00000116809.11	ZBTB17	6.478	24.001	9.296E-11	0.000				1	1	1	9.296E-11	0.001	Heart_Atrial_Appendag
ENSG00000146215.13	CRIP3	6.476	0.070	9.414E-11	0.060				2	2	2	3.298E-10	0.262	Heart_Left_Ventricle
ENSG00000055483.19	USP36	-6.473	-0.088	9.587E-11	0.033				1	1	1	9.587E-11	0.261	Artery_Coronary
ENSG00000151135.9	TMEM263	6.471	0.225	9.716E-11	0.005				2	2	2	2.279E-11	0.078	Heart_Left_Ventricle
ENSG00000196141.13	SPATS2L	-6.468	-0.124	9.952E-11	0.019				3	3	3	5.227E-11	0.277	Heart_Left_Ventricle
ENSG00000055483.19	USP36	6.463	0.378	1.027E-10	0.002				2	2	2	4.337E-11	0.050	Whole_Blood
ENSG00000147883.10	CDKN2B	-6.462	-2.393	1.030E-10	0.000				1	1	1	1.030E-10	0.010	Artery_Coronary
ENSG00000147883.10	CDKN2B	6.462	3.110	1.030E-10	0.000				1	1	1	1.030E-10	0.008	Artery_Tibial
ENSG00000147883.10	CDKN2B	-6.462	-2.228	1.030E-10	0.000				1	1	1	1.030E-10	0.011	Kidney_Cortex
ENSG00000182240.15	BACE2	-6.422	-0.049	1.345E-10	0.123				3	3	3	4.834E-10	0.386	Artery_Aorta

gene	gene_name	zscore	effect_size	pvalue	var_g	pred_	pred_	pred_	n_snps_used	n_snps_in_cov	n_snps_in_model	best_gwas_p	largest_weight	Tissue
ENSG00000111785.19	RIC8B	6.421	0.379	1.350E-10	0.002				2	2	2	1.377E-10	0.062	Heart_Left_Ventricle
ENSG00000177082.12	WDR73	-6.418	-0.075	1.380E-10	0.050				2	2	2	1.380E-10	0.357	Adipose_Visceral_Om
ENSG00000111785.19	RIC8B	6.417	0.330	1.385E-10	0.002				2	2	2	1.377E-10	0.072	Adipose_Subcutaneou
ENSG00000128591.15	FLNC	-6.409	-3.668	1.464E-10	0.000				1	1	1	1.464E-10	0.011	Artery_Aorta
ENSG00000128591.15	FLNC	6.409	0.340	1.464E-10	0.002				1	1	1	1.464E-10	0.114	Liver
ENSG00000146215.13	CRIP3	-6.402	-0.167	1.537E-10	0.010				2	2	2	3.298E-10	0.088	Artery_Tibial
ENSG00000177082.12	WDR73	-6.390	-0.055	1.664E-10	0.093				2	2	2	1.186E-10	0.484	Artery_Tibial
ENSG00000146215.13	CRIP3	6.385	0.234	1.709E-10	0.005				2	2	2	3.298E-10	0.062	Whole_Blood
ENSG00000169016.16	E2F6	6.380	0.129	1.775E-10	0.018				1	1	1	1.775E-10	0.195	Heart_Atrial_Appendag
ENSG00000055483.19	USP36	-6.375	-0.136	1.828E-10	0.015				1	1	1	1.828E-10	0.174	Heart_Atrial_Appendag
ENSG00000146215.13	CRIP3	6.358	0.089	2.041E-10	0.028				2	2	2	3.298E-10	0.165	Kidney_Cortex
ENSG00000177082.12	WDR73	-6.354	-0.109	2.103E-10	0.026				2	2	2	1.372E-10	0.252	Heart_Left_Ventricle
ENSG00000025039.14	RRAGD	6.347	0.222	2.195E-10	0.005				1	2	2	2.195E-10	0.100	Adipose_Subcutaneou
ENSG00000177082.12	WDR73	-6.347	-0.057	2.196E-10	0.094				2	2	2	1.380E-10	0.469	Artery_Aorta
ENSG00000141458.12	NPC1	-6.345	-0.041	2.223E-10	0.150				3	3	3	2.509E-13	0.258	Artery_Tibial
ENSG00000224477.5	RP1-81D8.3	-6.336	-0.055	2.358E-10	0.096				2	2	2	2.336E-11	0.406	Liver
ENSG00000112081.16	SRSF3	6.330	7.048	2.446E-10	0.000				2	2	2	6.034E-10	0.007	Heart_Left_Ventricle
ENSG00000119326.14	CTNNAL1	6.328	0.036	2.480E-10	0.233				3	4	4	6.852E-10	0.731	Artery_Tibial
ENSG00000119326.14	CTNNAL1	6.322	0.046	2.583E-10	0.130				4	4	4	1.532E-08	0.639	Artery_Aorta
ENSG00000182240.15	BACE2	-6.320	-0.027	2.614E-10	0.410				3	3	3	4.834E-10	0.885	Artery_Tibial
ENSG00000146147.14	MLIP	-6.320	-0.075	2.616E-10	0.046				1	1	1	2.616E-10	0.331	Adipose_Subcutaneou
ENSG00000055483.19	USP36	-6.320	-0.119	2.620E-10	0.019				2	2	2	1.007E-10	0.198	Heart_Left_Ventricle
ENSG00000177082.12	WDR73	-6.294	-0.165	3.102E-10	0.010				2	2	2	1.410E-10	0.159	Whole_Blood
ENSG00000146215.13	CRIP3	-6.284	-0.388	3.298E-10	0.002				1	1	1	3.298E-10	0.062	Adipose_Visceral_Om
ENSG00000146215.13	CRIP3	-6.284	-6.599	3.298E-10	0.000				1	1	1	3.298E-10	0.004	Artery_Coronary
ENSG00000173641.17	HSPB7	6.275	0.284	3.490E-10	0.003				2	2	2	1.302E-14	0.070	Heart_Left_Ventricle
ENSG00000134490.13	TMEM241	-6.272	-0.074	3.559E-10	0.051				2	2	2	6.278E-09	0.188	Heart_Atrial_Appendag
ENSG00000055483.19	USP36	-6.266	-0.080	3.699E-10	0.041				2	2	2	1.007E-10	0.280	Artery_Tibial
ENSG00000272391.5	POM121C	6.250	0.104	4.099E-10	0.045				2	3	3	8.098E-11	0.240	Artery_Aorta
ENSG00000182240.15	BACE2	-6.224	-0.049	4.834E-10	0.121				1	1	1	4.834E-10	0.483	Heart_Atrial_Appendag
ENSG00000182240.15	BACE2	-6.224	-0.043	4.834E-10	0.150				1	1	1	4.834E-10	0.556	Artery_Coronary
ENSG00000131771.13	PPP1R1B	6.212	0.134	5.223E-10	0.015				2	2	2	5.331E-10	0.186	Heart_Atrial_Appendag
ENSG00000214546.3	AC087491.2	-6.209	-216.570	5.331E-10	0.000				1	1	1	5.331E-10	0.000	Adipose_Subcutaneou
ENSG00000111785.19	RIC8B	6.206	0.416	5.436E-10	0.001				2	2	2	1.377E-10	0.074	Whole_Blood
ENSG00000161395.13	PGAP3	6.199	0.114	5.690E-10	0.028				1	2	2	5.690E-10	0.253	Adipose_Visceral_Om
ENSG00000108306.11	FBXL20	-6.197	-0.171	5.765E-10	0.007				1	1	1	5.765E-10	0.164	Kidney_Cortex
ENSG00000108669.16	CYTH1	6.189	0.100	6.047E-10	0.026				2	2	2	6.745E-13	0.157	Heart_Atrial_Appendag

gene	gene_name	zscore	effect_size	pvalue	var_g	pred_	pred_	pred_	n_snps_used	n_snps_in_cov	n_snps_in_model	best_gwas_p	largest_weight	Tissue
ENSG00000105971.14	CAV2	-6.182	-0.397	6.345E-10	0.002				2	2	2	1.920E-09	0.057	Heart_Atrial_Appendag
ENSG00000141736.13	ERBB2	6.180	0.366	6.417E-10	0.002				2	2	2	6.468E-14	0.133	Liver
ENSG00000272391.5	POM121C	6.172	0.085	6.763E-10	0.065				2	3	3	8.098E-11	0.343	Whole_Blood
ENSG00000118655.4	DCLRE1B	-6.170	-0.231	6.835E-10	0.005				1	1	1	6.835E-10	0.109	Artery_Coronary
ENSG00000119326.14	CTNNAL1	6.169	0.083	6.852E-10	0.033				1	1	1	6.852E-10	0.400	Kidney_Cortex
ENSG00000119326.14	CTNNAL1	6.169	0.074	6.852E-10	0.055				1	1	1	6.852E-10	0.452	Artery_Coronary
ENSG00000233276.3	GPX1	6.166	0.248	7.014E-10	0.004				2	2	2	4.012E-08	0.065	Whole_Blood
ENSG00000166348.18	USP54	6.146	0.131	7.927E-10	0.018				2	2	2	1.451E-08	0.414	Artery_Aorta
ENSG00000055483.19	USP36	-6.144	-0.169	8.031E-10	0.009				2	2	2	1.828E-10	0.131	Adipose_Subcutaneou
ENSG00000108669.16	CYTH1	6.132	0.099	8.670E-10	0.025				2	2	2	1.364E-12	0.160	Heart_Left_Ventricle
ENSG00000178053.17	MLF1	6.122	0.327	9.227E-10	0.002				1	1	1	9.227E-10	0.067	Kidney_Cortex
ENSG00000178053.17	MLF1	6.122	0.108	9.227E-10	0.020				1	1	1	9.227E-10	0.202	Artery_Coronary
ENSG00000204310.12	AGPAT1	6.112	0.046	9.811E-10	0.118				1	1	1	9.811E-10	0.487	Liver
ENSG00000204314.10	PRRT1	-6.110	-0.403	9.950E-10	0.002				3	3	3	2.511E-05	0.076	Artery_Tibial
ENSG00000108306.11	FBXL20	-6.107	-0.284	1.017E-09	0.003				2	2	2	5.765E-10	0.124	Liver
ENSG00000169016.16	E2F6	-6.106	-0.490	1.021E-09	0.001				1	1	1	1.021E-09	0.048	Artery_Coronary
ENSG00000169016.16	E2F6	-6.106	-0.334	1.021E-09	0.002				1	1	1	1.021E-09	0.071	Artery_Aorta
ENSG00000113580.14	NR3C1	-6.095	-0.584	1.096E-09	0.001				1	1	1	1.096E-09	0.050	Whole_Blood
ENSG00000136383.6	ALPK3	6.089	0.285	1.138E-09	0.003				2	2	2	1.343E-10	0.097	Artery_Tibial
ENSG00000118655.4	DCLRE1B	-6.087	-0.128	1.150E-09	0.013				2	2	2	5.109E-09	0.123	Artery_Tibial
ENSG00000151135.9	TMEM263	6.086	0.137	1.157E-09	0.014				2	2	2	3.417E-11	0.110	Adipose_Visceral_Om
ENSG00000146147.14	MLIP	-6.064	-0.124	1.332E-09	0.017				2	2	2	1.330E-09	0.184	Artery_Aorta
ENSG00000263001.5	GTF2I	6.062	0.103	1.341E-09	0.064				2	2	2	2.319E-09	0.428	Artery_Aorta
ENSG00000148297.15	MED22	6.046	1.334	1.482E-09	0.000				1	1	1	1.482E-09	0.027	Heart_Left_Ventricle
ENSG00000178053.17	MLF1	6.041	0.089	1.533E-09	0.027				2	2	2	9.227E-10	0.246	Artery_Aorta
ENSG00000106771.12	TMEM245	-6.039	-0.096	1.548E-09	0.028				2	2	2	6.613E-11	0.158	Liver
ENSG00000128596.16	CCDC136	-6.033	-0.112	1.608E-09	0.020				2	2	2	1.953E-09	0.172	Artery_Coronary
ENSG00000204356.13	NELFE	6.031	0.185	1.631E-09	0.006				3	3	3	1.741E-08	0.204	Heart_Atrial_Appendag
ENSG00000273559.4	CWC25	6.025	0.786	1.688E-09	0.000				1	1	1	1.688E-09	0.032	Kidney_Cortex
ENSG00000128596.16	CCDC136	-6.025	-0.157	1.691E-09	0.009				2	2	2	1.953E-09	0.237	Artery_Aorta
ENSG00000108379.9	WNT3	6.012	0.036	1.835E-09	0.205				2	2	2	2.686E-08	0.538	Adipose_Subcutaneou
ENSG00000149084.12	HSD17B12	-6.006	-0.055	1.907E-09	0.071				1	1	1	1.907E-09	0.418	Liver
ENSG00000070061.14	IKBKAP	-5.984	-0.142	2.184E-09	0.018				2	2	2	1.819E-08	0.299	Adipose_Subcutaneou
ENSG00000128596.16	CCDC136	-5.983	-0.141	2.195E-09	0.013				2	2	2	2.209E-09	0.228	Artery_Tibial
ENSG00000115486.11	GGCX	5.980	0.060	2.237E-09	0.055				1	1	1	2.237E-09	0.354	Heart_Left_Ventricle
ENSG00000115486.11	GGCX	5.980	0.055	2.237E-09	0.068				1	1	1	2.237E-09	0.390	Artery_Aorta
ENSG00000115486.11	GGCX	5.980	0.127	2.237E-09	0.012				1	1	1	2.237E-09	0.168	Liver

gene	gene_name	zscore	effect_size	pvalue	var_g	pred_	pred_	pred_	n_snps_used	n_snps_in_cov	n_snps_in_model	best_gwas_p	largest_weight	Tissue
ENSG00000148120.16	C9orf3	-5.975	-0.205	2.299E-09	0.005				1	1	1	2.299E-09	0.106	Heart_Atrial_Appendag
ENSG00000196275.13	GTF2IRD2	5.974	0.078	2.319E-09	0.111				1	2	2	2.319E-09	0.569	Liver
ENSG00000263001.5	GTF2I	5.974	0.120	2.319E-09	0.054				1	1	1	2.319E-09	0.371	Artery_Coronary
ENSG00000263001.5	GTF2I	5.974	0.103	2.319E-09	0.062				1	1	1	2.319E-09	0.433	Adipose_Subcutaneou
ENSG00000263001.5	GTF2I	5.974	0.116	2.319E-09	0.046				1	1	1	2.319E-09	0.383	Artery_Tibial
ENSG00000263001.5	GTF2I	5.974	0.145	2.319E-09	0.031				1	1	1	2.319E-09	0.309	Whole_Blood
ENSG00000263001.5	GTF2I	5.974	0.199	2.319E-09	0.016				1	1	1	2.319E-09	0.224	Heart_Left_Ventricle
ENSG00000263001.5	GTF2I	5.974	0.131	2.319E-09	0.040				1	1	1	2.319E-09	0.342	Liver
ENSG00000158517.13	NCF1	5.974	0.413	2.319E-09	0.004				1	2	2	2.319E-09	0.108	Whole_Blood
ENSG00000228672.3	PROB1	5.968	0.171	2.399E-09	0.007				1	1	1	2.399E-09	0.136	Heart_Atrial_Appendag
ENSG00000228672.3	PROB1	5.968	0.053	2.399E-09	0.075				1	1	1	2.399E-09	0.435	Artery_Tibial
ENSG00000228672.3	PROB1	5.968	0.079	2.399E-09	0.034				1	2	2	2.399E-09	0.292	Adipose_Subcutaneou
ENSG00000228672.3	PROB1	5.968	0.077	2.399E-09	0.036				1	1	1	2.399E-09	0.300	Artery_Coronary
ENSG00000228672.3	PROB1	5.968	0.069	2.399E-09	0.044				1	1	1	2.399E-09	0.338	Artery_Aorta
ENSG00000067208.14	EVI5	5.968	0.096	2.407E-09	0.028				2	2	2	4.078E-09	0.212	Artery_Tibial
ENSG00000146215.13	CRIP3	5.966	0.061	2.429E-09	0.106				2	2	2	3.546E-15	0.290	Heart_Atrial_Appendag
ENSG00000124762.13	CDKN1A	-5.958	-1.238	2.558E-09	0.000				2	2	2	1.632E-09	0.020	Adipose_Visceral_Om
ENSG00000173517.10	PEAK1	5.955	14.946	2.598E-09	0.000				1	1	1	2.598E-09	0.002	Artery_Coronary
ENSG00000173517.10	PEAK1	-5.955	-2.709	2.598E-09	0.000				1	1	1	2.598E-09	0.008	Adipose_Visceral_Om
ENSG00000228672.3	PROB1	5.955	0.165	2.607E-09	0.008				2	2	2	2.399E-09	0.139	Heart_Left_Ventricle
ENSG00000115486.11	GGCX	5.953	0.036	2.639E-09	0.173				2	2	2	7.545E-09	0.434	Whole_Blood
ENSG00000196141.13	SPATS2L	5.948	0.109	2.716E-09	0.019				2	2	2	5.227E-11	0.215	Artery_Tibial
ENSG00000161395.13	PGAP3	5.945	0.099	2.758E-09	0.032				2	2	2	5.690E-10	0.395	Artery_Aorta
ENSG00000161395.13	PGAP3	5.943	0.141	2.799E-09	0.011				2	3	3	5.415E-13	0.347	Artery_Tibial
ENSG00000169016.16	E2F6	5.927	0.097	3.085E-09	0.027				2	2	2	1.775E-10	0.217	Heart_Left_Ventricle
ENSG00000150938.9	CRIM1	-5.925	-0.098	3.125E-09	0.022				2	2	2	4.880E-09	0.183	Heart_Left_Ventricle
ENSG00000260025.1	RP11-490M8.1	-5.925	-0.047	3.132E-09	0.097				2	2	2	9.852E-09	0.404	Heart_Left_Ventricle
ENSG00000214160.9	ALG3	5.918	0.231	3.265E-09	0.004				2	2	2	3.653E-11	0.070	Heart_Left_Ventricle
ENSG00000196141.13	SPATS2L	5.899	0.392	3.661E-09	0.001				2	2	2	5.227E-11	0.132	Kidney_Cortex
ENSG00000067208.14	EVI5	5.889	0.111	3.877E-09	0.020				2	2	2	4.078E-09	0.197	Whole_Blood
ENSG00000119326.14	CTNNAL1	5.881	0.057	4.077E-09	0.076				2	2	2	6.852E-10	0.528	Adipose_Visceral_Om
ENSG00000067208.14	EVI5	5.881	0.169	4.078E-09	0.007				1	1	1	4.078E-09	0.208	Heart_Atrial_Appendag
ENSG00000067208.14	EVI5	5.881	0.246	4.078E-09	0.004				1	1	1	4.078E-09	0.143	Artery_Coronary
ENSG00000151617.15	EDNRA	5.879	0.131	4.139E-09	0.012				2	2	2	1.451E-08	0.172	Liver
ENSG00000154305.16	MIA3	-5.874	-0.211	4.246E-09	0.004				2	2	2	1.310E-08	0.106	Adipose_Visceral_Om
ENSG00000185614.4	FAM212A	5.870	0.320	4.345E-09	0.002				1	1	1	4.345E-09	0.067	Heart_Atrial_Appendag
ENSG00000185614.4	FAM212A	-5.870	-0.207	4.345E-09	0.005				1	1	1	4.345E-09	0.104	Artery_Tibial

gene	gene_name	zscore	effect_size	pvalue	var_g	pred_	pred_	pred_	n_snps_used	n_snps_in_cov	n_snps_in_model	best_gwas_p	largest_weight	Tissue
ENSG00000185614.4	FAM212A	-5.870	-0.263	4.345E-09	0.003				1	1	1	4.345E-09	0.082	Adipose_Visceral_Om
ENSG00000143106.12	PSMA5	-5.868	-0.429	4.403E-09	0.002				2	2	2	3.711E-09	0.057	Liver
ENSG00000171303.6	KCNK3	5.865	0.164	4.483E-09	0.008				1	1	1	4.483E-09	0.129	Adipose_Subcutaneou
ENSG00000158517.13	NCF1	5.864	0.222	4.515E-09	0.006				1	1	1	4.515E-09	0.112	Adipose_Visceral_Om
ENSG00000138286.14	FAM149B1	-5.848	-0.149	4.971E-09	0.007				1	1	1	4.971E-09	0.270	Whole_Blood
ENSG00000118655.4	DCLRE1B	-5.844	-0.157	5.109E-09	0.008				1	1	1	5.109E-09	0.171	Adipose_Subcutaneou
ENSG00000118655.4	DCLRE1B	-5.844	-0.175	5.109E-09	0.008				1	1	1	5.109E-09	0.154	Liver
ENSG00000118655.4	DCLRE1B	-5.844	-0.270	5.109E-09	0.003				1	1	1	5.109E-09	0.099	Whole_Blood
ENSG00000176095.11	IP6K1	5.843	0.335	5.119E-09	0.002				1	1	1	5.119E-09	0.064	Artery_Tibial
ENSG00000170873.18	MTSS1	5.836	0.065	5.362E-09	0.045				2	2	2	6.067E-10	0.322	Heart_Atrial_Appendag
ENSG00000204310.12	AGPAT1	-5.834	-0.363	5.407E-09	0.002				2	2	2	2.322E-08	0.064	Whole_Blood
ENSG00000154305.16	MIA3	-5.834	-0.131	5.410E-09	0.012				2	2	2	4.793E-10	0.134	Adipose_Subcutaneou
ENSG00000146147.14	MLIP	-5.829	-0.026	5.561E-09	0.334				2	2	2	5.569E-09	0.849	Liver
ENSG00000173641.17	HSPB7	5.828	0.061	5.619E-09	0.054				4	4	4	1.116E-20	0.248	Artery_Tibial
ENSG00000149084.12	HSD17B12	-5.819	-0.027	5.937E-09	0.291				2	2	2	2.204E-08	0.848	Artery_Tibial
ENSG00000039650.11	PNKP	-5.808	-0.204	6.318E-09	0.006				2	2	2	5.860E-09	0.107	Liver
ENSG00000187492.8	CDHR4	5.790	0.120	7.035E-09	0.016				1	1	1	7.035E-09	0.177	Artery_Aorta
ENSG00000196821.9	C6orf106	5.785	0.120	7.263E-09	0.015				2	2	2	3.659E-08	0.171	Heart_Left_Ventricle
ENSG00000170482.16	SLC23A1	5.780	0.079	7.468E-09	0.028				1	1	1	7.468E-09	0.265	Heart_Left_Ventricle
ENSG00000170482.16	SLC23A1	5.780	0.660	7.468E-09	0.000				1	1	1	7.468E-09	0.032	Artery_Coronary
ENSG00000170482.16	SLC23A1	5.780	0.227	7.468E-09	0.003				1	1	1	7.468E-09	0.092	Artery_Aorta
ENSG00000170482.16	SLC23A1	5.780	0.541	7.468E-09	0.001				1	1	1	7.468E-09	0.038	Heart_Atrial_Appendag
ENSG00000148660.20	CAMK2G	5.773	0.347	7.803E-09	0.002				1	1	1	7.803E-09	0.061	Adipose_Subcutaneou
ENSG00000149084.12	HSD17B12	-5.766	-0.037	8.139E-09	0.177				2	2	2	1.881E-08	0.693	Kidney_Cortex
ENSG00000136379.11	ABHD17C	5.757	0.117	8.580E-09	0.015				1	1	1	8.580E-09	0.181	Heart_Left_Ventricle
ENSG00000136379.11	ABHD17C	5.757	0.110	8.580E-09	0.017				1	1	1	8.580E-09	0.192	Artery_Aorta
ENSG00000112164.5	GLP1R	-5.755	-0.188	8.672E-09	0.007				1	1	1	8.672E-09	0.115	Adipose_Visceral_Om
ENSG00000112164.5	GLP1R	-5.755	-0.166	8.672E-09	0.009				1	1	1	8.672E-09	0.130	Adipose_Subcutaneou
ENSG00000272354.1	RP11-307L14.2	-5.749	-6.299	8.968E-09	0.000				2	2	2	1.424E-08	0.005	Artery_Coronary
ENSG00000156042.17	CFAP70	5.747	0.085	9.080E-09	0.018				1	1	1	9.080E-09	0.438	Whole_Blood
ENSG00000141744.3	PNMT	5.737	0.258	9.655E-09	0.003				2	2	2	3.614E-09	0.083	Adipose_Subcutaneou
ENSG00000149084.12	HSD17B12	-5.726	-0.041	1.026E-08	0.115				2	2	2	3.254E-08	0.556	Heart_Atrial_Appendag
ENSG00000177733.6	HNRNPA0	-5.723	-1.072	1.049E-08	0.000				1	1	1	1.049E-08	0.025	Kidney_Cortex
ENSG00000151617.15	EDNRA	5.718	0.211	1.078E-08	0.004				2	2	2	1.451E-08	0.096	Artery_Aorta
ENSG00000198373.12	WWP2	5.717	0.202	1.082E-08	0.006				1	1	1	1.082E-08	0.121	Artery_Tibial
ENSG00000149084.12	HSD17B12	-5.714	-0.029	1.106E-08	0.249				2	2	2	2.204E-08	0.772	Adipose_Visceral_Om
ENSG00000172992.11	DCAKD	-5.708	-0.044	1.140E-08	0.116				2	2	2	1.875E-07	0.354	Kidney_Cortex

gene	gene_name	zscore	effect_size	pvalue	var_g	pred_	pred_	pred_	n_snps_used	n_snps_in_cov	n_snps_in_model	best_gwas_p	largest_weight	Tissue
ENSG00000197696.9	NMB	-5.704	-0.147	1.170E-08	0.015				1	1	1	1.170E-08	0.196	Whole_Blood
ENSG00000197696.9	NMB	-5.704	-0.073	1.170E-08	0.079				1	1	1	1.170E-08	0.393	Kidney_Cortex
ENSG00000050820.16	BCAR1	5.701	0.089	1.190E-08	0.021				1	1	1	1.190E-08	0.216	Artery_Aorta
ENSG00000108433.16	GOSR2	5.691	0.259	1.261E-08	0.003				1	1	1	1.261E-08	0.081	Artery_Aorta
ENSG00000146247.13	PHIP	5.690	0.295	1.269E-08	0.002				1	1	1	1.269E-08	0.075	Artery_Tibial
ENSG00000164916.10	FOXK1	-5.687	-0.070	1.296E-08	0.037				1	1	1	1.296E-08	0.583	Heart_Atrial_Appendag
ENSG00000164068.15	RNF123	5.684	0.081	1.318E-08	0.035				1	2	2	1.318E-08	0.262	Artery_Aorta
ENSG00000164068.15	RNF123	5.684	0.090	1.318E-08	0.027				1	2	2	1.318E-08	0.234	Artery_Tibial
ENSG00000164068.15	RNF123	5.684	0.072	1.318E-08	0.043				1	2	2	1.318E-08	0.293	Adipose_Subcutaneou
ENSG00000164068.15	RNF123	5.684	0.343	1.318E-08	0.002				1	1	1	1.318E-08	0.062	Whole_Blood
ENSG00000149084.12	HSD17B12	-5.684	-0.030	1.318E-08	0.215				3	3	3	3.254E-08	0.736	Artery_Coronary
ENSG00000149084.12	HSD17B12	-5.678	-0.026	1.360E-08	0.301				3	3	3	2.204E-08	0.820	Adipose_Subcutaneou
ENSG00000163491.16	NEK10	-5.675	-0.114	1.388E-08	0.018				1	1	1	1.388E-08	0.193	Artery_Aorta
ENSG00000151135.9	TMEM263	5.669	0.056	1.437E-08	0.073				3	3	3	3.417E-11	0.151	Adipose_Subcutaneou
ENSG00000151617.15	EDNRA	5.667	0.278	1.451E-08	0.002				1	1	1	1.451E-08	0.099	Heart_Atrial_Appendag
ENSG00000166348.18	USP54	5.667	0.886	1.451E-08	0.000				1	1	1	1.451E-08	0.039	Kidney_Cortex
ENSG00000166348.18	USP54	-5.667	-0.387	1.451E-08	0.001				1	1	1	1.451E-08	0.089	Whole_Blood
ENSG00000166348.18	USP54	5.667	0.136	1.451E-08	0.006				1	1	1	1.451E-08	0.252	Heart_Atrial_Appendag
ENSG00000166348.18	USP54	5.667	0.578	1.451E-08	0.000				1	1	1	1.451E-08	0.059	Liver
ENSG00000151617.15	EDNRA	5.667	0.779	1.451E-08	0.000				1	1	1	1.451E-08	0.035	Adipose_Visceral_Om
ENSG00000166348.18	USP54	5.667	0.145	1.451E-08	0.006				1	1	1	1.451E-08	0.237	Artery_Coronary
ENSG00000127804.12	METTL16	-5.662	-0.146	1.494E-08	0.009				1	1	1	1.494E-08	0.172	Liver
ENSG00000110536.13	PTPMT1	5.662	0.294	1.498E-08	0.003				2	2	2	1.317E-07	0.146	Adipose_Visceral_Om
ENSG00000164068.15	RNF123	5.662	0.180	1.500E-08	0.006				2	2	2	1.318E-08	0.115	Heart_Left_Ventricle
ENSG00000153113.23	CAST	-5.657	-4.148	1.539E-08	0.000				1	1	1	1.539E-08	0.005	Heart_Left_Ventricle
ENSG00000124762.13	CDKN1A	-5.650	-20.267	1.601E-08	0.000				1	1	1	1.601E-08	0.001	Adipose_Subcutaneou
ENSG00000198862.13	LTN1	-5.647	-0.331	1.628E-08	0.002				2	2	2	3.419E-08	0.156	Adipose_Subcutaneou
ENSG00000138792.9	ENPEP	-5.639	-0.083	1.710E-08	0.033				4	4	4	1.111E-04	0.908	Adipose_Visceral_Om
ENSG00000204356.13	NELFE	5.636	1.106	1.741E-08	0.000				1	1	1	1.741E-08	0.044	Adipose_Visceral_Om
ENSG00000149084.12	HSD17B12	-5.627	-0.028	1.830E-08	0.268				2	2	2	4.430E-08	0.785	Artery_Aorta
ENSG00000164068.15	RNF123	5.615	0.094	1.961E-08	0.026				1	1	1	1.961E-08	0.221	Artery_Coronary
ENSG00000164068.15	RNF123	5.615	0.142	1.961E-08	0.011				1	1	1	1.961E-08	0.147	Heart_Atrial_Appendag
ENSG00000164068.15	RNF123	5.615	0.091	1.961E-08	0.026				1	2	2	1.961E-08	0.229	Adipose_Visceral_Om
ENSG00000164916.10	FOXK1	-5.611	-0.073	2.008E-08	0.042				2	2	2	1.296E-08	0.531	Heart_Left_Ventricle
ENSG00000008196.12	TFAP2B	5.611	35.689	2.012E-08	0.000				1	1	1	2.012E-08	0.001	Kidney_Cortex
ENSG00000008196.12	TFAP2B	5.611	24.165	2.012E-08	0.000				1	2	2	2.012E-08	0.001	Adipose_Subcutaneou
ENSG00000115486.11	GGCX	5.610	0.068	2.018E-08	0.038				2	2	2	2.237E-09	0.275	Adipose_Subcutaneou

gene	gene_name	zscore	effect_size	pvalue	var_g	pred_	pred_	pred_	n_snps_used	n_snps_in_cov	n_snps_in_model	best_gwas_p	largest_weight	Tissue
ENSG00000138675.16	FGF5	5.609	0.046	2.039E-08	0.088				2	2	2	1.461E-08	0.461	Kidney_Cortex
ENSG00000170464.9	DNAJC18	-5.603	-0.072	2.108E-08	0.034				1	1	1	2.108E-08	0.292	Artery_Tibial
ENSG00000170464.9	DNAJC18	-5.603	-0.049	2.108E-08	0.093				1	1	1	2.108E-08	0.431	Kidney_Cortex
ENSG00000170464.9	DNAJC18	-5.603	-0.031	2.108E-08	0.179				1	1	1	2.108E-08	0.677	Heart_Atrial_Appendag
ENSG00000108641.14	B9D1	5.600	0.231	2.144E-08	0.004				1	1	1	2.144E-08	0.115	Adipose_Visceral_Om
ENSG00000108641.14	B9D1	5.600	0.183	2.144E-08	0.007				1	1	1	2.144E-08	0.144	Adipose_Subcutaneou
ENSG00000108641.14	B9D1	5.600	2.185	2.144E-08	0.000				1	1	1	2.144E-08	0.012	Heart_Atrial_Appendag
ENSG00000108641.14	B9D1	5.600	0.121	2.144E-08	0.017				1	1	1	2.144E-08	0.219	Artery_Coronary
ENSG00000108641.14	B9D1	-5.600	-1.753	2.144E-08	0.000				1	1	1	2.144E-08	0.015	Liver
ENSG00000115486.11	GGCX	5.599	0.053	2.158E-08	0.070				3	3	3	2.237E-09	0.353	Heart_Atrial_Appendag
ENSG00000118849.9	RARRES1	5.589	0.049	2.282E-08	0.073				3	3	3	9.064E-08	0.269	Liver
ENSG00000204310.12	AGPAT1	5.586	0.175	2.322E-08	0.007				1	1	1	2.322E-08	0.114	Heart_Left_Ventricle
ENSG00000228727.8	SAPCD1	5.584	1.394	2.353E-08	0.000				1	1	1	2.353E-08	0.019	Artery_Tibial
ENSG00000178053.17	MLF1	5.583	0.091	2.358E-08	0.025				2	2	2	9.227E-10	0.193	Whole_Blood
ENSG00000166484.19	MAPK7	5.580	0.527	2.401E-08	0.001				2	2	2	2.144E-08	0.050	Artery_Aorta
ENSG00000263001.5	GTF2I	5.579	0.063	2.417E-08	0.142				2	2	2	2.319E-09	0.359	Adipose_Visceral_Om
ENSG00000170464.9	DNAJC18	-5.574	-0.036	2.483E-08	0.125				1	1	1	2.483E-08	0.574	Heart_Left_Ventricle
ENSG00000176095.11	IP6K1	5.574	0.249	2.483E-08	0.004				2	2	2	1.307E-09	0.124	Adipose_Visceral_Om
ENSG00000170464.9	DNAJC18	-5.573	-0.038	2.507E-08	0.110				2	2	2	2.108E-08	0.348	Adipose_Visceral_Om
ENSG00000055483.19	USP36	-5.570	-0.120	2.548E-08	0.025				2	2	2	1.394E-08	0.220	Liver
ENSG00000182240.15	BACE2	-5.569	-0.105	2.567E-08	0.021				3	3	3	4.834E-10	0.170	Adipose_Subcutaneou
ENSG00000185614.4	FAM212A	-5.568	-0.142	2.582E-08	0.011				2	2	2	4.345E-09	0.139	Artery_Coronary
ENSG00000263001.5	GTF2I	5.560	0.145	2.695E-08	0.026				2	2	2	2.319E-09	0.265	Heart_Atrial_Appendag
ENSG00000108433.16	GOSR2	5.545	0.157	2.947E-08	0.009				2	2	2	1.261E-08	0.194	Whole_Blood
ENSG00000166321.13	NUDT13	5.540	0.052	3.026E-08	0.044				2	2	2	7.759E-08	0.716	Heart_Left_Ventricle
ENSG00000115486.11	GGCX	5.536	0.081	3.093E-08	0.027				1	1	1	3.093E-08	0.250	Adipose_Visceral_Om
ENSG00000115561.15	CHMP3	-5.535	-0.135	3.108E-08	0.010				2	2	2	8.481E-08	0.116	Heart_Left_Ventricle
ENSG00000213551.4	DNAJC9	5.515	0.068	3.486E-08	0.025				2	2	2	5.675E-10	0.305	Heart_Left_Ventricle
ENSG00000130227.16	XPO7	-5.515	-0.279	3.495E-08	0.002				2	2	2	4.229E-08	0.068	Artery_Coronary
ENSG00000183527.11	PSMG1	-5.513	-0.023	3.522E-08	0.379				4	4	4	3.537E-08	0.459	Artery_Tibial
ENSG00000118849.9	RARRES1	5.513	0.055	3.529E-08	0.059				2	2	2	9.064E-08	0.213	Adipose_Subcutaneou
ENSG00000196821.9	C6orf106	5.507	0.121	3.659E-08	0.009				1	1	1	3.659E-08	0.186	Kidney_Cortex
ENSG00000196821.9	C6orf106	5.507	0.189	3.659E-08	0.005				1	2	2	3.659E-08	0.119	Artery_Coronary
ENSG00000121039.9	RDH10	-5.507	-0.629	3.660E-08	0.000				1	1	1	3.660E-08	0.040	Artery_Coronary
ENSG00000170464.9	DNAJC18	-5.506	-0.074	3.666E-08	0.031				2	2	2	2.108E-08	0.168	Adipose_Subcutaneou
ENSG00000110536.13	PTPMT1	5.505	0.397	3.689E-08	0.002				2	2	2	1.317E-07	0.123	Whole_Blood
ENSG00000182218.9	HHIPL1	-5.505	-0.121	3.698E-08	0.012				2	2	2	1.092E-05	0.213	Artery_Coronary

gene	gene_name	zscore	effect_size	pvalue	var_g	pred_	pred_	pred_	n_snps_used	n_snps_in_cov	n_snps_in_model	best_gwas_p	largest_weight	Tissue
ENSG00000176095.11	IP6K1	5.504	0.249	3.709E-08	0.004				2	2	2	2.089E-09	0.098	Adipose_Subcutaneou
ENSG00000166822.12	TMEM170A	-5.501	-0.194	3.775E-08	0.004				1	1	1	3.775E-08	0.099	Heart_Atrial_Appendag
ENSG00000198373.12	WWP2	5.497	0.107	3.855E-08	0.022				3	3	3	7.593E-10	0.437	Artery_Aorta
ENSG00000141458.12	NPC1	-5.497	-0.021	3.873E-08	0.412				3	3	3	4.090E-10	0.381	Heart_Left_Ventricle
ENSG00000115486.11	GGCX	5.495	0.034	3.915E-08	0.157				3	3	3	5.100E-09	0.344	Artery_Tibial
ENSG00000185909.14	KLHDC8B	5.494	0.521	3.920E-08	0.001				2	2	2	7.379E-08	0.046	Liver
ENSG00000031003.10	FAM13B	-5.492	-0.063	3.968E-08	0.057				3	3	3	1.391E-05	0.291	Heart_Atrial_Appendag
ENSG00000233276.3	GPX1	5.490	0.812	4.012E-08	0.000				1	1	1	4.012E-08	0.028	Adipose_Subcutaneou
ENSG00000233276.3	GPX1	5.490	0.272	4.012E-08	0.003				1	1	1	4.012E-08	0.084	Heart_Atrial_Appendag
ENSG00000233276.3	GPX1	5.490	0.324	4.012E-08	0.002				1	1	1	4.012E-08	0.071	Artery_Tibial
ENSG00000233276.3	GPX1	5.490	0.208	4.012E-08	0.005				1	1	1	4.012E-08	0.110	Artery_Aorta
ENSG00000233276.3	GPX1	5.490	0.507	4.012E-08	0.001				1	1	1	4.012E-08	0.045	Adipose_Visceral_Om
ENSG00000170464.9	DNAJC18	-5.490	-0.026	4.026E-08	0.223				2	2	2	2.108E-08	0.481	Liver
ENSG00000116793.15	PHTF1	-5.488	-0.122	4.054E-08	0.011				1	1	1	4.054E-08	0.185	Artery_Tibial
ENSG00000116793.15	PHTF1	-5.488	-0.154	4.054E-08	0.008				1	1	1	4.054E-08	0.146	Artery_Coronary
ENSG00000116793.15	PHTF1	-5.488	-0.133	4.054E-08	0.009				1	1	1	4.054E-08	0.170	Adipose_Visceral_Om
ENSG00000116793.15	PHTF1	-5.488	-0.173	4.054E-08	0.006				1	1	1	4.054E-08	0.130	Artery_Aorta
ENSG00000185909.14	KLHDC8B	5.486	0.398	4.103E-08	0.001				2	2	2	7.379E-08	0.056	Adipose_Visceral_Om
ENSG00000166348.18	USP54	5.481	0.099	4.224E-08	0.016				2	2	2	1.451E-08	0.217	Heart_Left_Ventricle
ENSG00000149084.12	HSD17B12	-5.477	-0.030	4.317E-08	0.220				2	2	2	3.254E-08	0.688	Whole_Blood
ENSG00000173421.16	CCDC36	-5.473	-0.019	4.434E-08	0.588				2	2	2	6.013E-08	0.697	Artery_Aorta
ENSG00000115561.15	CHMP3	-5.473	-0.106	4.435E-08	0.018				3	3	3	8.481E-08	0.146	Heart_Atrial_Appendag
ENSG00000170464.9	DNAJC18	-5.470	-0.033	4.511E-08	0.151				2	2	2	2.108E-08	0.358	Whole_Blood
ENSG00000141736.13	ERBB2	5.465	0.571	4.623E-08	0.001				2	2	2	6.468E-14	0.084	Artery_Coronary
ENSG00000103253.17	HAGHL	5.464	0.028	4.667E-08	0.218				3	3	3	2.424E-07	0.565	Heart_Atrial_Appendag
ENSG00000185909.14	KLHDC8B	5.463	0.210	4.683E-08	0.005				2	2	2	2.903E-08	0.114	Adipose_Subcutaneou
ENSG00000128596.16	CCDC136	5.459	0.115	4.783E-08	0.013				1	1	1	4.783E-08	0.235	Liver
ENSG00000070061.14	IKBKAP	-5.452	-0.137	4.987E-08	0.016				4	4	4	1.819E-08	0.252	Artery_Tibial
ENSG00000130227.16	XPO7	-5.449	-0.319	5.077E-08	0.002				2	2	2	4.229E-08	0.058	Liver
ENSG00000188266.13	HYKK	-5.448	-0.247	5.097E-08	0.003				1	1	1	5.097E-08	0.077	Whole_Blood
ENSG00000178053.17	MLF1	5.447	0.037	5.112E-08	0.142				3	3	3	9.227E-10	0.330	Heart_Left_Ventricle
ENSG00000166348.18	USP54	5.447	0.062	5.115E-08	0.040				2	2	2	1.451E-08	0.422	Artery_Tibial
ENSG00000204475.9	NCR3	5.447	0.097	5.136E-08	0.023				3	3	3	8.136E-07	0.428	Adipose_Subcutaneou
ENSG00000214546.3	AC087491.2	-5.442	-126.254	5.283E-08	0.000				1	1	1	5.283E-08	0.000	Adipose_Visceral_Om
ENSG00000172992.11	DCAKD	-5.436	-0.019	5.446E-08	0.563				4	4	4	1.875E-07	0.370	Artery_Aorta
ENSG00000130227.16	XPO7	-5.433	-0.298	5.540E-08	0.002				2	2	2	4.229E-08	0.062	Artery_Aorta
ENSG00000128482.15	RNF112	5.431	0.072	5.598E-08	0.042				2	2	2	4.175E-08	0.224	Artery_Aorta

gene	gene_name	zscore	effect_size	pvalue	var_g	pred_	pred_	pred_	n_snps_used	n_snps_in_cov	n_snps_in_model	best_gwas_p	largest_weight	Tissue
ENSG00000186063.12	AIDA	5.431	0.471	5.609E-08	0.001				2	2	2	2.230E-07	0.048	Kidney_Cortex
ENSG00000170464.9	DNAJC18	-5.423	-0.044	5.873E-08	0.088				2	2	2	2.108E-08	0.258	Artery_Coronary
ENSG00000091732.15	ZC3HC1	5.419	0.328	5.978E-08	0.002				2	2	2	4.535E-08	0.064	Heart_Atrial_Appendag
ENSG00000173421.16	CCDC36	-5.418	-0.047	6.013E-08	0.092				1	2	2	6.013E-08	0.439	Adipose_Visceral_Om
ENSG00000173421.16	CCDC36	-5.418	-0.063	6.013E-08	0.046				1	2	2	6.013E-08	0.332	Kidney_Cortex
ENSG00000166348.18	USP54	5.418	0.088	6.021E-08	0.021				2	2	2	1.451E-08	0.309	Adipose_Visceral_Om
ENSG00000166822.12	TMEM170A	-5.417	-0.183	6.058E-08	0.004				2	2	2	7.263E-08	0.099	Liver
ENSG00000131748.15	STARD3	-5.410	-10.048	6.296E-08	0.000				1	1	1	6.296E-08	0.002	Adipose_Visceral_Om
ENSG00000146243.13	IRAK1BP1	5.408	0.059	6.375E-08	0.052				2	2	2	1.411E-07	0.313	Kidney_Cortex
ENSG00000182240.15	BACE2	-5.405	-0.100	6.464E-08	0.027				2	2	2	9.101E-09	0.192	Heart_Left_Ventricle
ENSG00000166348.18	USP54	5.404	0.090	6.501E-08	0.018				2	2	2	1.451E-08	0.326	Adipose_Subcutaneou
ENSG00000070366.13	SMG6	5.402	0.682	6.573E-08	0.000				1	1	1	6.573E-08	0.045	Adipose_Subcutaneou
ENSG00000122008.15	POLK	-5.400	-0.177	6.647E-08	0.005				1	1	1	6.647E-08	0.123	Artery_Coronary
ENSG00000131389.16	SLC6A6	-5.400	-0.461	6.678E-08	0.001				1	1	1	6.678E-08	0.050	Heart_Atrial_Appendag
ENSG00000110536.13	PTPMT1	5.395	0.197	6.861E-08	0.006				2	2	2	1.317E-07	0.239	Artery_Aorta
ENSG00000185519.8	FAM131C	5.394	0.074	6.907E-08	0.036				2	2	2	1.197E-06	0.175	Adipose_Subcutaneou
ENSG00000035928.15	RFC1	-5.392	-0.136	6.969E-08	0.009				2	2	2	1.013E-06	0.082	Artery_Tibial
ENSG00000109163.6	GNRHR	-5.383	-0.233	7.329E-08	0.004				1	1	1	7.329E-08	0.086	Liver
ENSG00000110536.13	PTPMT1	5.382	0.290	7.367E-08	0.002				2	2	2	1.317E-07	0.167	Liver
ENSG00000070061.14	IKBKAP	-5.378	-0.097	7.529E-08	0.026				1	1	1	7.529E-08	0.313	Artery_Coronary
ENSG00000128482.15	RNF112	5.378	0.115	7.538E-08	0.016				2	2	2	4.175E-08	0.212	Adipose_Visceral_Om
ENSG00000158865.12	SLC5A11	-5.377	-0.018	7.586E-08	0.514				2	2	2	7.597E-08	1.385	Artery_Coronary
ENSG00000158865.12	SLC5A11	-5.376	-0.018	7.597E-08	0.483				1	1	1	7.597E-08	1.369	Adipose_Visceral_Om
ENSG00000166321.13	NUDT13	5.373	0.039	7.759E-08	0.103				1	1	1	7.759E-08	0.883	Kidney_Cortex
ENSG00000166321.13	NUDT13	5.373	0.025	7.759E-08	0.170				1	1	1	7.759E-08	1.380	Artery_Tibial
ENSG00000204310.12	AGPAT1	5.372	0.168	7.766E-08	0.007				2	2	2	2.322E-08	0.115	Heart_Atrial_Appendag
ENSG00000176986.15	SEC24C	-5.370	-0.318	7.895E-08	0.002				3	3	3	4.210E-21	0.037	Heart_Atrial_Appendag
ENSG00000035928.15	RFC1	-5.365	-0.162	8.112E-08	0.007				2	2	2	1.013E-06	0.068	Artery_Aorta
ENSG00000213551.4	DNAJC9	5.363	0.102	8.173E-08	0.015				2	2	2	5.675E-10	0.241	Kidney_Cortex
ENSG00000272791.1	RP11-464F9.22	5.361	0.486	8.267E-08	0.001				2	2	2	4.396E-08	0.053	Kidney_Cortex
ENSG00000145020.15	AMT	-5.357	-0.059	8.456E-08	0.057				1	1	1	8.456E-08	0.380	Whole_Blood
ENSG00000145020.15	AMT	-5.357	-0.061	8.456E-08	0.054				1	1	1	8.456E-08	0.369	Adipose_Visceral_Om
ENSG00000145020.15	AMT	-5.357	-0.060	8.456E-08	0.059				1	1	1	8.456E-08	0.371	Artery_Coronary
ENSG00000114316.12	USP4	5.356	0.211	8.504E-08	0.005				1	1	1	8.504E-08	0.106	Liver
ENSG00000114316.12	USP4	5.356	0.257	8.504E-08	0.003				1	1	1	8.504E-08	0.087	Artery_Tibial
ENSG00000114316.12	USP4	5.356	0.265	8.504E-08	0.003				1	1	1	8.504E-08	0.084	Artery_Coronary
ENSG00000114316.12	USP4	5.356	0.185	8.504E-08	0.006				1	1	1	8.504E-08	0.121	Heart_Left_Ventricle

gene	gene_name	zscore	effect_size	pvalue	var_g	pred_	pred_	pred_	n_snps_used	n_snps_in_cov	n_snps_in_model	best_gwas_p	largest_weight	Tissue
ENSG00000114316.12	USP4	5.356	0.624	8.504E-08	0.001				1	1	1	8.504E-08	0.036	Whole_Blood
ENSG00000114316.12	USP4	5.356	0.191	8.504E-08	0.006				1	1	1	8.504E-08	0.118	Heart_Atrial_Appendag
ENSG00000114316.12	USP4	5.356	0.161	8.504E-08	0.008				1	1	1	8.504E-08	0.139	Artery_Aorta
ENSG00000227078.1	LINC02094	5.354	0.048	8.602E-08	0.108				2	2	2	5.977E-07	0.340	Artery_Aorta
ENSG00000179673.4	RPRML	-5.354	-0.081	8.626E-08	0.027				2	2	2	8.796E-08	0.235	Artery_Aorta
ENSG00000008869.11	HEATR5B	5.352	0.362	8.684E-08	0.002				1	1	1	8.684E-08	0.129	Adipose_Visceral_Om
ENSG00000106460.18	TMEM106B	5.352	0.087	8.720E-08	0.024				1	1	1	8.720E-08	0.238	Whole_Blood
ENSG00000213676.10	ATF6B	5.350	0.079	8.781E-08	0.040				2	3	3	5.006E-06	0.306	Artery_Aorta
ENSG00000145020.15	AMT	-5.348	-0.488	8.911E-08	0.001				1	1	1	8.911E-08	0.046	Liver
ENSG00000035928.15	RFC1	-5.345	-0.299	9.047E-08	0.002				2	2	2	1.013E-06	0.037	Heart_Atrial_Appendag
ENSG00000274021.1	RP11-823E8.3	-5.344	-1.829	9.077E-08	0.000				2	2	2	2.111E-08	0.011	Liver
ENSG00000067208.14	EVI5	5.343	0.078	9.122E-08	0.032				2	2	2	4.078E-09	0.193	Adipose_Visceral_Om
ENSG00000164867.10	NOS3	-5.343	-0.205	9.122E-08	0.006				1	1	1	9.122E-08	0.193	Heart_Atrial_Appendag
ENSG00000164867.10	NOS3	-5.343	-0.242	9.122E-08	0.004				1	1	1	9.122E-08	0.163	Artery_Tibial
ENSG00000164867.10	NOS3	-5.343	-0.215	9.122E-08	0.005				1	1	1	9.122E-08	0.184	Artery_Aorta
ENSG00000164867.10	NOS3	-5.343	-0.271	9.122E-08	0.003				1	1	1	9.122E-08	0.146	Heart_Left_Ventricle
ENSG00000109065.11	NAT9	-5.339	-0.222	9.334E-08	0.005				3	3	3	5.245E-07	0.229	Artery_Aorta
ENSG00000148290.9	SURF1	-5.336	-0.028	9.488E-08	0.526				5	5	5	2.792E-15	0.509	Heart_Atrial_Appendag
ENSG00000130177.14	CDC16	5.336	0.055	9.492E-08	0.078				2	2	2	4.597E-07	0.418	Liver
ENSG00000108433.16	GOSR2	5.334	0.134	9.627E-08	0.010				3	3	3	1.039E-07	0.147	Heart_Left_Ventricle
ENSG00000186665.9	C17orf58	-5.332	-1.087	9.732E-08	0.000				1	1	1	9.732E-08	0.020	Adipose_Subcutaneou
ENSG00000065526.10	SPEN	-5.331	-0.765	9.751E-08	0.001				1	1	1	9.751E-08	0.037	Artery_Tibial
ENSG00000124562.9	SNRPC	-5.327	-0.117	9.969E-08	0.016				2	2	2	1.457E-11	0.126	Heart_Left_Ventricle
ENSG00000172992.11	DCAKD	-5.320	-0.045	1.035E-07	0.080				2	2	2	7.081E-06	0.332	Heart_Atrial_Appendag
ENSG00000227078.1	LINC02094	5.316	0.050	1.060E-07	0.102				2	2	2	5.977E-07	0.315	Artery_Tibial
ENSG00000130584.10	ZBTB46	-5.315	-0.093	1.065E-07	0.019				1	1	1	1.065E-07	0.210	Artery_Aorta
ENSG00000158865.12	SLC5A11	-5.315	-0.020	1.066E-07	0.440				2	2	2	1.070E-07	1.271	Whole_Blood
ENSG00000158865.12	SLC5A11	-5.314	-0.020	1.070E-07	0.409				1	1	1	1.070E-07	1.232	Heart_Atrial_Appendag
ENSG00000105662.15	CRTC1	5.314	0.409	1.075E-07	0.001				1	1	1	1.075E-07	0.053	Adipose_Visceral_Om
ENSG00000154229.11	PRKCA	-5.313	-0.033	1.079E-07	0.174				2	2	2	8.908E-09	0.466	Heart_Left_Ventricle
ENSG00000196821.9	C6orf106	5.311	0.129	1.088E-07	0.012				1	1	1	1.088E-07	0.235	Heart_Atrial_Appendag
ENSG00000050426.15	LETMD1	-5.310	-0.063	1.098E-07	0.048				1	1	1	1.098E-07	0.306	Heart_Left_Ventricle
ENSG00000204385.10	SLC44A4	-5.300	-820.511	1.158E-07	0.000				1	1	1	1.158E-07	0.000	Kidney_Cortex
ENSG00000035928.15	RFC1	-5.298	-0.195	1.173E-07	0.004				2	2	2	1.013E-06	0.063	Adipose_Visceral_Om
ENSG00000108433.16	GOSR2	5.292	0.145	1.208E-07	0.010				2	2	2	1.026E-07	0.143	Adipose_Visceral_Om
ENSG00000171634.16	BPTF	-5.292	-0.323	1.209E-07	0.002				1	1	1	1.209E-07	0.075	Kidney_Cortex
ENSG00000171634.16	BPTF	-5.292	-0.380	1.209E-07	0.002				1	1	1	1.209E-07	0.064	Adipose_Subcutaneou

gene	gene_name	zscore	effect_size	pvalue	var_g	pred_	pred_	pred_	n_snps_used	n_snps_in_cov	n_snps_in_model	best_gwas_p	largest_weight	Tissue
ENSG00000171634.16	BPTF	-5.292	-0.356	1.209E-07	0.002				1	1	1	1.209E-07	0.068	Liver
ENSG00000171634.16	BPTF	-5.292	-0.334	1.209E-07	0.002				1	1	1	1.209E-07	0.073	Artery_Tibial
ENSG00000130177.14	CDC16	5.285	0.033	1.254E-07	0.159				2	2	2	2.325E-07	0.479	Heart_Left_Ventricle
ENSG00000225399.4	RP11-3B7.1	-5.283	-0.040	1.271E-07	0.118				2	2	2	1.344E-07	0.510	Artery_Coronary
ENSG00000196666.4	FAM180B	-5.281	-0.260	1.284E-07	0.004				1	1	1	1.284E-07	0.184	Adipose_Subcutaneou
ENSG00000196666.4	FAM180B	-5.281	-0.333	1.284E-07	0.002				1	1	1	1.284E-07	0.143	Artery_Aorta
ENSG00000196666.4	FAM180B	-5.281	-0.355	1.284E-07	0.002				1	1	1	1.284E-07	0.134	Artery_Tibial
ENSG00000181652.19	ATG9B	-5.280	-0.176	1.290E-07	0.008				2	2	2	9.122E-08	0.220	Artery_Aorta
ENSG00000131748.15	STARD3	-5.278	-0.234	1.304E-07	0.002				2	2	2	6.296E-08	0.238	Artery_Coronary
ENSG00000198563.13	DDX39B	-5.278	-0.186	1.309E-07	0.006				1	1	1	1.309E-07	0.118	Artery_Tibial
ENSG00000106460.18	TMEM106B	5.277	0.085	1.312E-07	0.034				2	2	2	2.358E-08	0.173	Kidney_Cortex
ENSG00000149187.17	CELF1	5.276	0.727	1.317E-07	0.000				1	1	1	1.317E-07	0.066	Heart_Atrial_Appendag
ENSG00000110536.13	PTPMT1	5.276	0.200	1.317E-07	0.006				1	1	1	1.317E-07	0.240	Kidney_Cortex
ENSG00000110536.13	PTPMT1	5.276	0.271	1.317E-07	0.004				1	1	1	1.317E-07	0.177	Artery_Coronary
ENSG00000149187.17	CELF1	5.276	0.886	1.317E-07	0.000				1	1	1	1.317E-07	0.054	Heart_Left_Ventricle
ENSG00000110536.13	PTPMT1	5.276	0.260	1.317E-07	0.004				1	1	1	1.317E-07	0.184	Artery_Tibial
ENSG00000149187.17	CELF1	5.276	0.999	1.317E-07	0.000				1	1	1	1.317E-07	0.048	Artery_Coronary
ENSG00000225399.4	RP11-3B7.1	-5.273	-0.056	1.344E-07	0.063				1	1	1	1.344E-07	0.362	Adipose_Visceral_Om
ENSG00000225399.4	RP11-3B7.1	-5.273	-0.033	1.344E-07	0.172				1	1	1	1.344E-07	0.606	Artery_Aorta
ENSG00000035928.15	RFC1	-5.268	-0.116	1.379E-07	0.013				2	2	2	1.013E-06	0.097	Artery_Coronary
ENSG00000123268.8	ATF1	5.267	0.037	1.386E-07	0.135				2	2	2	7.671E-07	0.386	Artery_Aorta
ENSG00000204472.12	AIF1	5.263	0.246	1.419E-07	0.003				2	2	2	3.035E-07	0.069	Adipose_Visceral_Om
ENSG00000145029.13	NICN1	-5.261	-0.091	1.431E-07	0.025				1	1	1	1.431E-07	0.242	Artery_Coronary
ENSG00000145029.13	NICN1	-5.261	-0.745	1.431E-07	0.000				1	1	1	1.431E-07	0.030	Whole_Blood
ENSG00000145029.13	NICN1	-5.261	-0.101	1.431E-07	0.019				1	1	1	1.431E-07	0.217	Kidney_Cortex
ENSG00000081154.11	PCNP	5.255	0.499	1.477E-07	0.001				1	1	1	1.477E-07	0.039	Heart_Atrial_Appendag
ENSG00000081154.11	PCNP	5.255	0.795	1.477E-07	0.000				1	1	1	1.477E-07	0.025	Kidney_Cortex
ENSG00000225399.4	RP11-3B7.1	-5.249	-0.048	1.527E-07	0.081				2	2	2	1.344E-07	0.421	Heart_Left_Ventricle
ENSG00000070061.14	IKBKAP	-5.248	-0.100	1.537E-07	0.028				3	3	3	1.819E-08	0.336	Adipose_Visceral_Om
ENSG00000110536.13	PTPMT1	5.242	0.196	1.589E-07	0.006				2	2	2	1.317E-07	0.243	Heart_Atrial_Appendag
ENSG00000225399.4	RP11-3B7.1	-5.241	-0.038	1.600E-07	0.131				2	2	2	1.344E-07	0.528	Artery_Tibial
ENSG00000173402.11	DAG1	-5.239	-0.588	1.618E-07	0.001				1	1	1	1.618E-07	0.037	Artery_Tibial
ENSG00000173402.11	DAG1	-5.239	-0.568	1.618E-07	0.001				1	1	1	1.618E-07	0.039	Heart_Left_Ventricle
ENSG00000228672.3	PROB1	5.235	0.073	1.649E-07	0.027				1	1	1	1.649E-07	0.244	Whole_Blood
ENSG00000170476.15	MZB1	5.235	0.661	1.649E-07	0.000				1	1	1	1.649E-07	0.027	Heart_Left_Ventricle
ENSG00000130595.17	TNNT3	-5.234	-0.078	1.657E-07	0.028				2	2	2	1.697E-07	0.251	Heart_Left_Ventricle
ENSG00000158865.12	SLC5A11	-5.234	-0.018	1.663E-07	0.503				2	2	2	7.597E-08	1.341	Adipose_Subcutaneou

gene	gene_name	zscore	effect_size	pvalue	var_g	pred_	pred_	pred_	n_snps_used	n_snps_in_cov	n_snps_in_model	best_gwas_p	largest_weight	Tissue
ENSG00000178053.17	MLF1	5.232	0.055	1.673E-07	0.056				2	2	2	9.227E-10	0.273	Heart_Atrial_Appendag
ENSG00000123268.8	ATF1	5.230	0.091	1.693E-07	0.023				2	2	2	7.671E-07	0.154	Heart_Left_Ventricle
ENSG00000173421.16	CCDC36	-5.224	-0.031	1.746E-07	0.193				2	2	2	1.840E-07	0.651	Artery_Coronary
ENSG00000130177.14	CDC16	5.223	0.038	1.764E-07	0.119				1	1	1	1.764E-07	0.569	Artery_Coronary
ENSG00000010310.8	GIPR	-5.221	-0.530	1.779E-07	0.001				1	1	1	1.779E-07	0.054	Artery_Tibial
ENSG00000122008.15	POLK	-5.219	-0.163	1.801E-07	0.006				2	2	2	6.647E-08	0.125	Heart_Left_Ventricle
ENSG00000173641.17	HSPB7	5.217	0.101	1.816E-07	0.018				3	3	3	1.302E-14	0.122	Heart_Atrial_Appendag
ENSG00000166822.12	TMEM170A	-5.217	-0.115	1.817E-07	0.013				3	3	3	7.263E-08	0.103	Adipose_Subcutaneou
ENSG00000173421.16	CCDC36	-5.215	-0.036	1.840E-07	0.155				1	2	2	1.840E-07	0.560	Heart_Atrial_Appendag
ENSG00000173421.16	CCDC36	-5.215	-0.046	1.840E-07	0.089				1	2	2	1.840E-07	0.436	Adipose_Subcutaneou
ENSG00000173421.16	CCDC36	-5.215	-0.041	1.840E-07	0.111				1	2	2	1.840E-07	0.490	Heart_Left_Ventricle
ENSG00000115561.15	CHMP3	-5.215	-0.055	1.842E-07	0.066				2	2	2	2.387E-07	0.339	Adipose_Subcutaneou
ENSG00000110536.13	PTPMT1	5.214	0.257	1.843E-07	0.004				2	2	2	1.317E-07	0.185	Heart_Left_Ventricle
ENSG00000183072.9	NKX2-5	5.210	0.265	1.890E-07	0.002				1	1	1	1.890E-07	0.075	Heart_Atrial_Appendag
ENSG00000136448.11	NMT1	5.205	0.066	1.941E-07	0.043				2	2	2	3.773E-06	0.223	Liver
ENSG00000188779.10	SKOR1	5.202	0.064	1.969E-07	0.033				1	1	1	1.969E-07	0.327	Heart_Left_Ventricle
ENSG00000108379.9	WNT3	5.198	0.027	2.011E-07	0.260				3	3	3	2.686E-08	0.538	Adipose_Visceral_Om
ENSG00000170482.16	SLC23A1	5.197	0.199	2.022E-07	0.003				1	1	1	2.022E-07	0.088	Whole_Blood
ENSG00000070061.14	IKBKAP	-5.197	-0.094	2.022E-07	0.022				2	2	2	7.529E-08	0.290	Heart_Atrial_Appendag
ENSG00000204348.9	DXO	5.194	0.099	2.054E-07	0.017				2	2	2	3.615E-06	0.218	Liver
ENSG00000243649.8	CFB	5.192	0.206	2.083E-07	0.004				1	1	1	2.083E-07	0.147	Heart_Atrial_Appendag
ENSG00000172992.11	DCAKD	-5.190	-0.017	2.105E-07	0.592				3	3	3	7.081E-06	0.518	Artery_Tibial
ENSG00000145022.4	TCTA	-5.188	-0.194	2.120E-07	0.005				1	1	1	2.120E-07	0.111	Artery_Tibial
ENSG00000157259.6	GATAD1	5.186	0.104	2.147E-07	0.024				3	3	3	3.464E-05	0.450	Heart_Atrial_Appendag
ENSG00000177352.9	CCDC71	5.184	0.174	2.172E-07	0.005				1	1	1	2.172E-07	0.113	Kidney_Cortex
ENSG00000177352.9	CCDC71	5.184	0.335	2.172E-07	0.002				1	1	1	2.172E-07	0.059	Adipose_Visceral_Om
ENSG00000177352.9	CCDC71	5.184	0.208	2.172E-07	0.004				1	1	1	2.172E-07	0.094	Whole_Blood
ENSG00000154305.16	MIA3	-5.181	-1.430	2.202E-07	0.000				1	1	1	2.202E-07	0.025	Heart_Left_Ventricle
ENSG00000154305.16	MIA3	5.181	0.457	2.202E-07	0.001				1	1	1	2.202E-07	0.078	Whole_Blood
ENSG00000139722.6	VPS37B	5.181	0.151	2.204E-07	0.005				1	1	1	2.204E-07	0.176	Heart_Left_Ventricle
ENSG00000139722.6	VPS37B	5.181	0.059	2.204E-07	0.031				1	1	1	2.204E-07	0.447	Artery_Coronary
ENSG00000105329.9	TGFB1	5.181	0.183	2.204E-07	0.006				3	3	3	4.634E-07	0.142	Artery_Coronary
ENSG00000186063.12	AIDA	-5.179	-0.542	2.230E-07	0.001				1	1	1	2.230E-07	0.045	Whole_Blood
ENSG00000176095.11	IP6K1	5.174	0.272	2.291E-07	0.003				2	2	2	3.574E-09	0.082	Artery_Aorta
ENSG00000168890.13	TMEM150A	-5.171	-0.181	2.324E-07	0.005				1	1	1	2.324E-07	0.111	Heart_Left_Ventricle
ENSG00000168883.19	USP39	-5.171	-0.162	2.324E-07	0.007				1	1	1	2.324E-07	0.124	Adipose_Subcutaneou
ENSG00000130177.14	CDC16	5.171	0.028	2.325E-07	0.237				1	1	1	2.325E-07	0.769	Whole_Blood

gene	gene_name	zscore	effect_size	pvalue	var_g	pred_	pred_	pred_	n_snps_used	n_snps_in_cov	n_snps_in_model	best_gwas_p	largest_weight	Tissue
ENSG00000185909.14	KLHDC8B	5.169	0.111	2.354E-07	0.013				2	2	2	7.379E-08	0.172	Kidney_Cortex
ENSG00000225399.4	RP11-3B7.1	-5.166	-0.062	2.388E-07	0.047				2	2	2	1.344E-07	0.316	Adipose_Subcutaneou
ENSG00000108641.14	B9D1	5.165	0.045	2.407E-07	0.114				2	2	2	2.144E-08	0.319	Artery_Aorta
ENSG00000085831.15	TTC39A	-5.164	-1.542	2.415E-07	0.000				2	2	2	4.795E-12	0.027	Artery_Tibial
ENSG00000178053.17	MLF1	5.163	0.060	2.430E-07	0.049				3	3	3	9.227E-10	0.239	Artery_Tibial
ENSG00000073605.18	GSDMB	5.163	0.042	2.431E-07	0.098				3	3	3	9.829E-08	0.339	Whole_Blood
ENSG00000168899.4	VAMP5	-5.160	-0.166	2.463E-07	0.007				1	1	1	2.463E-07	0.122	Adipose_Visceral_Om
ENSG00000104946.12	TBC1D17	-5.157	-0.235	2.507E-07	0.002				2	2	2	5.860E-09	0.078	Whole_Blood
ENSG00000170469.10	SPATA24	-5.156	-0.070	2.518E-07	0.028				1	1	1	2.518E-07	0.253	Adipose_Subcutaneou
ENSG00000170469.10	SPATA24	-5.156	-0.112	2.518E-07	0.013				1	1	1	2.518E-07	0.157	Kidney_Cortex
ENSG00000122484.8	RPAP2	5.155	0.143	2.532E-07	0.012				2	2	2	1.008E-06	0.128	Heart_Atrial_Appendag
ENSG00000081154.11	PCNP	5.147	0.523	2.647E-07	0.001				1	1	1	2.647E-07	0.044	Whole_Blood
ENSG00000138075.11	ABCG5	-5.145	-1.467	2.675E-07	0.000				1	1	1	2.675E-07	0.026	Kidney_Cortex
ENSG00000143921.6	ABCG8	-5.145	-6.077	2.675E-07	0.000				1	1	1	2.675E-07	0.006	Liver
ENSG00000138075.11	ABCG5	-5.145	-1.123	2.675E-07	0.000				1	1	1	2.675E-07	0.034	Adipose_Visceral_Om
ENSG00000162407.8	PLPP3	5.145	0.131	2.681E-07	0.009				2	2	2	2.342E-07	0.183	Heart_Left_Ventricle
ENSG00000170185.9	USP38	-5.144	-0.333	2.684E-07	0.001				1	2	2	2.684E-07	0.057	Adipose_Visceral_Om
ENSG00000237541.3	HLA-DQA2	5.143	0.041	2.702E-07	0.191				1	3	3	2.702E-07	0.672	Liver
ENSG00000164062.12	APEH	-5.142	-0.227	2.723E-07	0.007				1	1	1	2.723E-07	0.125	Adipose_Subcutaneou
ENSG00000078403.16	MLLT10	-5.136	-2.155	2.804E-07	0.000				1	1	1	2.804E-07	0.012	Heart_Atrial_Appendag
ENSG00000204682.5	CASC10	5.136	0.144	2.807E-07	0.010				2	2	2	9.691E-08	0.149	Artery_Tibial
ENSG00000204387.12	C6orf48	5.135	0.111	2.816E-07	0.011				1	1	1	2.816E-07	0.226	Artery_Tibial
ENSG00000072110.13	ACTN1	5.133	0.332	2.850E-07	0.002				2	2	2	3.230E-07	0.062	Adipose_Subcutaneou
ENSG00000115561.15	CHMP3	-5.131	-0.059	2.877E-07	0.057				3	3	3	5.291E-08	0.371	Artery_Aorta
ENSG00000136448.11	NMT1	5.129	0.037	2.916E-07	0.130				3	3	3	3.773E-06	0.250	Artery_Aorta
ENSG00000164078.12	MST1R	-5.128	-0.085	2.935E-07	0.025				2	2	2	2.716E-07	0.231	Whole_Blood
ENSG00000172992.11	DCAKD	-5.127	-0.034	2.948E-07	0.164				3	3	3	4.558E-06	0.414	Adipose_Subcutaneou
ENSG00000172992.11	DCAKD	-5.121	-0.038	3.035E-07	0.139				2	3	3	7.081E-06	0.373	Artery_Coronary
ENSG00000168899.4	VAMP5	-5.120	-0.135	3.048E-07	0.010				2	2	2	2.324E-07	0.127	Adipose_Subcutaneou
ENSG00000225399.4	RP11-3B7.1	-5.119	-0.042	3.070E-07	0.108				3	3	3	1.344E-07	0.449	Heart_Atrial_Appendag
ENSG00000261701.6	HPR	-5.119	-0.098	3.072E-07	0.021				2	2	2	3.998E-07	0.222	Liver
ENSG00000256269.7	HMBS	5.119	0.057	3.074E-07	0.053				3	3	3	1.942E-06	0.284	Heart_Left_Ventricle
ENSG00000181885.18	CLDN7	-5.116	-0.079	3.115E-07	0.035				3	4	4	3.165E-05	0.401	Whole_Blood
ENSG00000167202.11	TBC1D2B	-5.116	-0.112	3.127E-07	0.015				3	3	3	3.026E-07	0.140	Heart_Left_Ventricle
ENSG00000103253.17	HAGHL	5.115	0.044	3.130E-07	0.071				1	1	1	3.130E-07	0.449	Adipose_Subcutaneou
ENSG00000168899.4	VAMP5	-5.115	-0.125	3.144E-07	0.011				2	2	2	2.463E-07	0.137	Heart_Left_Ventricle
ENSG00000198563.13	DDX39B	-5.110	-0.093	3.213E-07	0.023				2	3	3	1.309E-07	0.178	Adipose_Subcutaneou

gene	gene_name	zscore	effect_size	pvalue	var_g	pred_	pred_	pred_	n_snps_used	n_snps_in_cov	n_snps_in_model	best_gwas_p	largest_weight	Tissue
ENSG00000136383.6	ALPK3	5.108	0.111	3.262E-07	0.014				2	2	2	1.035E-07	0.148	Heart_Atrial_Appendag
ENSG00000204498.10	NFKBIL1	5.107	0.302	3.278E-07	0.001				1	1	1	3.278E-07	0.143	Adipose_Visceral_Om
ENSG00000204498.10	NFKBIL1	5.107	0.301	3.278E-07	0.001				1	2	2	3.278E-07	0.144	Whole_Blood
ENSG00000119979.16	FAM45A	-5.106	-0.358	3.282E-07	0.001				1	1	1	3.282E-07	0.050	Liver
ENSG00000005379.15	TSPOAP1	5.105	0.084	3.306E-07	0.028				3	3	3	5.024E-07	0.180	Adipose_Subcutaneou
ENSG00000182180.13	MRPS16	-5.105	-0.409	3.311E-07	0.001				2	2	2	1.356E-09	0.073	Kidney_Cortex
ENSG00000146215.13	CRIP3	-5.103	-0.570	3.336E-07	0.000				1	1	1	3.336E-07	0.038	Artery_Aorta
ENSG00000204463.12	BAG6	5.103	0.211	3.347E-07	0.004				2	2	2	3.035E-07	0.090	Whole_Blood
ENSG00000164867.10	NOS3	-5.102	-0.209	3.358E-07	0.005				2	2	2	9.122E-08	0.179	Adipose_Visceral_Om
ENSG00000272087.1	RP11-379F4.7	5.100	0.083	3.395E-07	0.022				2	2	2	4.292E-07	0.215	Artery_Aorta
ENSG00000112245.10	PTP4A1	-5.100	-0.722	3.400E-07	0.000				1	1	1	3.400E-07	0.024	Artery_Aorta
ENSG00000196141.13	SPATS2L	-5.096	-0.048	3.475E-07	0.081				4	4	4	4.574E-07	0.331	Heart_Atrial_Appendag
ENSG00000172992.11	DCAKD	-5.095	-0.038	3.481E-07	0.117				3	3	3	7.081E-06	0.241	Adipose_Visceral_Om
ENSG00000118655.4	DCLRE1B	-5.095	-0.133	3.487E-07	0.009				2	2	2	5.109E-09	0.132	Artery_Aorta
ENSG00000167112.9	TRUB2	-5.093	-0.117	3.533E-07	0.012				2	2	2	9.567E-08	0.194	Artery_Tibial
ENSG00000162676.11	GFI1	-5.088	-5.423	3.613E-07	0.000				1	1	1	3.613E-07	0.004	Artery_Aorta
ENSG00000162676.11	GFI1	5.088	3.716	3.613E-07	0.000				1	1	1	3.613E-07	0.007	Whole_Blood
ENSG00000162676.11	GFI1	-5.088	-4.708	3.613E-07	0.000				1	1	1	3.613E-07	0.005	Heart_Atrial_Appendag
ENSG00000162676.11	GFI1	-5.088	-6.088	3.613E-07	0.000				1	1	1	3.613E-07	0.004	Artery_Coronary
ENSG00000186665.9	C17orf58	-5.088	-0.554	3.616E-07	0.001				1	1	1	3.616E-07	0.040	Artery_Aorta
ENSG00000173175.14	ADCY5	5.084	0.212	3.694E-07	0.005				1	1	1	3.694E-07	0.106	Adipose_Subcutaneou
ENSG00000183527.11	PSMG1	-5.083	-0.071	3.713E-07	0.034				2	2	2	1.116E-06	0.258	Artery_Coronary
ENSG00000148120.16	C9orf3	-5.082	-0.066	3.736E-07	0.038				2	2	2	4.387E-14	0.404	Whole_Blood
ENSG00000052841.14	TTC17	-5.080	-0.048	3.773E-07	0.060				1	1	1	3.773E-07	0.390	Kidney_Cortex
ENSG00000052841.14	TTC17	5.080	11.138	3.773E-07	0.000				1	1	1	3.773E-07	0.002	Heart_Atrial_Appendag
ENSG00000197757.7	HOXC6	5.079	0.167	3.796E-07	0.006				1	1	1	3.796E-07	0.106	Artery_Aorta

Supplementary Table 1F: All-cause heart failure in a European population														
gene	gene_name	zscore	effect_size	pvalue	var_g	pred_	pred_	pred_	n_snps_used	n_snps_in_cov	n_snps_in_model	best_gwas_p	largest_weight	Tissue
ENSG00000124762.13	CDKN1A	13.776	2.365	3.558E-43	0.000				1	1	1	3.558E-43	0.028	Heart_Left_Ventricle
ENSG00000147883.10	CDKN2B	12.164	0.725	4.835E-34	0.002				2	2	2	1.160E-34	0.061	Artery_Aorta
ENSG00000177791.11	MYOZ1	-10.269	-0.038	9.747E-25	0.509				2	2	2	7.290E-23	1.044	Heart_Atrial_Appendag
ENSG00000196968.10	FUT11	9.806	0.203	1.066E-22	0.021				2	2	2	4.577E-21	0.160	Whole_Blood
ENSG00000166317.11	SYNPO2L	9.660	0.623	4.456E-22	0.002				1	1	1	4.456E-22	0.083	Heart_Atrial_Appendag
ENSG00000196968.10	FUT11	9.627	0.089	6.163E-22	0.085				2	2	2	3.991E-20	0.326	Adipose_Visceral_Om
ENSG00000166317.11	SYNPO2L	-9.613	-0.309	7.033E-22	0.007				2	2	2	4.456E-22	0.166	Artery_Tibial
ENSG00000196968.10	FUT11	9.596	0.067	8.302E-22	0.144				2	2	2	6.381E-20	0.410	Adipose_Subcutaneou
ENSG00000166317.11	SYNPO2L	9.495	1.067	2.204E-21	0.000				2	2	2	2.742E-21	0.048	Heart_Left_Ventricle
ENSG00000176986.15	SEC24C	-9.495	-0.816	2.206E-21	0.001				2	2	2	1.433E-20	0.034	Artery_Aorta
ENSG00000172650.13	AGAP5	-9.304	-0.466	1.347E-20	0.003				1	1	1	1.347E-20	0.114	Artery_Aorta
ENSG00000176986.15	SEC24C	9.298	2.566	1.433E-20	0.000				1	1	1	1.433E-20	0.021	Kidney_Cortex
ENSG00000196968.10	FUT11	9.138	0.137	6.381E-20	0.028				1	1	1	6.381E-20	0.380	Artery_Coronary
ENSG00000196968.10	FUT11	9.138	0.206	6.381E-20	0.009				1	1	1	6.381E-20	0.254	Kidney_Cortex
ENSG00000176986.15	SEC24C	-9.109	-5.171	8.291E-20	0.000				1	1	1	8.291E-20	0.010	Whole_Blood
ENSG00000176986.15	SEC24C	-9.109	-1.056	8.291E-20	0.001				1	1	1	8.291E-20	0.051	Artery_Tibial
ENSG00000176986.15	SEC24C	-9.051	-0.995	1.412E-19	0.001				2	2	2	8.291E-20	0.030	Artery_Coronary
ENSG00000176986.15	SEC24C	-8.955	-1.087	3.396E-19	0.000				2	2	2	8.291E-20	0.027	Heart_Left_Ventricle
ENSG00000115808.11	STRN	8.457	0.306	2.753E-17	0.005				2	2	2	4.058E-17	0.134	Artery_Aorta
ENSG00000124762.13	CDKN1A	-8.193	-2.067	2.538E-16	0.000				2	2	2	1.532E-28	0.018	Artery_Tibial
ENSG00000172650.13	AGAP5	-8.008	-0.079	1.168E-15	0.077				2	2	2	7.320E-15	0.222	Heart_Atrial_Appendag
ENSG00000141452.9	C18orf8	-8.005	-0.105	1.197E-15	0.043				3	3	3	7.239E-14	0.166	Whole_Blood
ENSG00000186510.11	CLCNKA	-7.909	-0.154	2.589E-15	0.019				1	1	1	2.589E-15	0.216	Kidney_Cortex
ENSG00000141452.9	C18orf8	-7.897	-0.070	2.848E-15	0.097				3	3	3	7.239E-14	0.247	Heart_Atrial_Appendag
ENSG00000172650.13	AGAP5	-7.863	-0.300	3.743E-15	0.007				1	1	1	3.743E-15	0.127	Adipose_Visceral_Om
ENSG00000172650.13	AGAP5	-7.863	-0.231	3.743E-15	0.012				1	1	1	3.743E-15	0.164	Heart_Left_Ventricle
ENSG00000172650.13	AGAP5	-7.863	-0.169	3.743E-15	0.022				1	1	1	3.743E-15	0.226	Liver
ENSG00000203356.2	LINC01562	7.859	0.106	3.886E-15	0.024				1	1	1	3.886E-15	0.950	Artery_Tibial
ENSG00000146243.13	IRAK1BP1	7.838	0.042	4.569E-15	0.263				3	3	3	9.940E-15	0.402	Adipose_Subcutaneou
ENSG00000141452.9	C18orf8	-7.786	-0.144	6.910E-15	0.021				2	2	2	7.239E-14	0.137	Heart_Left_Ventricle
ENSG00000172650.13	AGAP5	-7.779	-0.198	7.320E-15	0.012				1	1	1	7.320E-15	0.163	Adipose_Subcutaneou

gene	gene_name	zscore	effect_size	pvalue	var_g	pred_	pred	pred_	n_snps_used	n_snps_in_cov	n_snps_in_model	best_gwas_p	largest_weight	Tissue
ENSG00000141452.9	C18orf8	-7.768	-0.104	7.976E-15	0.041				2	2	2	7.239E-14	0.193	Artery_Tibial
ENSG00000172650.13	AGAP5	-7.743	-0.161	9.721E-15	0.021				2	2	2	1.413E-14	0.142	Whole_Blood
ENSG00000203356.2	LINC01562	7.737	0.109	1.020E-14	0.014				2	2	2	1.286E-14	0.891	Artery_Aorta
ENSG00000196968.10	FUT11	7.713	0.123	1.226E-14	0.029				1	1	1	1.226E-14	0.258	Artery_Aorta
ENSG00000203356.2	LINC01562	7.707	0.160	1.286E-14	0.011				1	1	1	1.286E-14	0.605	Adipose_Subcutaneou
ENSG00000123091.4	RNF11	-7.707	-7.359	1.286E-14	0.000				1	1	1	1.286E-14	0.013	Adipose_Subcutaneou
ENSG00000172650.13	AGAP5	-7.695	-0.293	1.413E-14	0.007				1	1	1	1.413E-14	0.126	Artery_Tibial
ENSG00000134222.16	PSRC1	-7.674	-0.033	1.663E-14	0.412				1	1	1	1.663E-14	1.099	Liver
ENSG00000141452.9	C18orf8	-7.623	-0.075	2.477E-14	0.078				3	3	3	7.239E-14	0.239	Adipose_Visceral_Om
ENSG00000141452.9	C18orf8	-7.611	-0.103	2.722E-14	0.042				2	2	2	7.239E-14	0.177	Artery_Coronary
ENSG00000141452.9	C18orf8	-7.559	-0.057	4.076E-14	0.134				3	3	3	7.239E-14	0.291	Adipose_Subcutaneou
ENSG00000213719.8	CLIC1	-7.539	-443.240	4.723E-14	0.000				2	2	2	7.185E-14	0.000	Kidney_Cortex
ENSG00000141458.12	NPC1	-7.493	-0.105	6.746E-14	0.036				2	2	2	4.406E-14	0.293	Artery_Aorta
ENSG00000196968.10	FUT11	7.486	0.161	7.086E-14	0.016				1	1	1	7.086E-14	0.189	Artery_Tibial
ENSG00000272140.2	RP11-574K11.29	7.464	0.490	8.405E-14	0.002				1	1	1	8.405E-14	0.062	Artery_Coronary
ENSG00000272140.2	RP11-574K11.29	7.464	0.258	8.405E-14	0.006				1	2	2	8.405E-14	0.118	Adipose_Visceral_Om
ENSG00000272140.2	RP11-574K11.29	7.464	0.213	8.405E-14	0.008				1	2	2	8.405E-14	0.143	Kidney_Cortex
ENSG00000272140.2	RP11-574K11.29	7.464	0.420	8.405E-14	0.002				1	2	2	8.405E-14	0.072	Heart_Left_Ventricle
ENSG00000272140.2	RP11-574K11.29	7.464	0.314	8.405E-14	0.004				1	2	2	8.405E-14	0.097	Whole_Blood
ENSG00000272140.2	RP11-574K11.29	7.464	0.300	8.405E-14	0.005				1	2	2	8.405E-14	0.101	Adipose_Subcutaneou
ENSG00000204385.10	SLC44A4	-7.447	-1330.065	9.513E-14	0.000				1	1	1	9.513E-14	0.000	Kidney_Cortex
ENSG00000196968.10	FUT11	7.417	0.093	1.197E-13	0.047				1	1	1	1.197E-13	0.322	Heart_Left_Ventricle
ENSG00000196968.10	FUT11	7.417	0.100	1.197E-13	0.041				1	1	1	1.197E-13	0.300	Heart_Atrial_Appendag
ENSG00000176986.15	SEC24C	-7.398	-0.405	1.378E-13	0.003				1	1	1	1.378E-13	0.077	Adipose_Subcutaneou
ENSG00000141452.9	C18orf8	-7.365	-0.156	1.773E-13	0.016				2	2	2	7.239E-14	0.184	Artery_Aorta
ENSG00000141458.12	NPC1	-7.311	-0.046	2.650E-13	0.194				2	2	2	4.406E-14	0.397	Heart_Atrial_Appendag
ENSG00000115808.11	STRN	-7.310	-0.147	2.663E-13	0.016				1	1	1	2.663E-13	0.184	Heart_Atrial_Appendag
ENSG00000005007.12	UPF1	7.258	0.436	3.936E-13	0.002				2	2	2	4.049E-13	0.066	Artery_Tibial
ENSG00000122884.12	P4HA1	-7.252	-12.347	4.105E-13	0.000				1	1	1	4.105E-13	0.005	Artery_Aorta
ENSG00000122884.12	P4HA1	-7.252	-15.877	4.105E-13	0.000				1	1	1	4.105E-13	0.004	Artery_Coronary
ENSG00000122884.12	P4HA1	-7.252	-10.988	4.105E-13	0.000				1	1	1	4.105E-13	0.005	Heart_Left_Ventricle
ENSG00000122884.12	P4HA1	-7.252	-4.836	4.105E-13	0.000				1	1	1	4.105E-13	0.012	Whole_Blood
ENSG00000122884.12	P4HA1	7.252	38.157	4.105E-13	0.000				1	1	1	4.105E-13	0.002	Liver

gene	gene_name	zscore	effect_size	pvalue	var_g	pred	pred	pred	n_snps_used	n_snps_in_cov	n_snps_in_model	best_gwas_p	largest_weight	Tissue
ENSG00000122884.12	P4HA1	7.252	20.600	4.105E-13	0.000				1	1	1	4.105E-13	0.003	Adipose_Visceral_Om
ENSG00000122884.12	P4HA1	-7.252	-13.753	4.105E-13	0.000				1	1	1	4.105E-13	0.004	Artery_Tibial
ENSG00000122884.12	P4HA1	7.252	35.738	4.105E-13	0.000				1	1	1	4.105E-13	0.002	Adipose_Subcutaneou
ENSG00000122884.12	P4HA1	-7.252	-20.302	4.105E-13	0.000				1	1	1	4.105E-13	0.003	Heart_Atrial_Appendag
ENSG00000177791.11	MYOZ1	-7.231	-0.077	4.794E-13	0.062				2	2	2	4.868E-13	0.614	Heart_Left_Ventricle
ENSG00000141458.12	NPC1	-7.191	-0.054	6.438E-13	0.140				3	3	3	4.406E-14	0.321	Artery_Coronary
ENSG00000085831.15	TTC39A	-7.161	-3.221	8.011E-13	0.000				2	2	2	8.419E-13	0.026	Artery_Coronary
ENSG00000085831.15	TTC39A	-7.154	-98.536	8.419E-13	0.000				1	1	1	8.419E-13	0.001	Heart_Atrial_Appendag
ENSG00000146243.13	IRAK1BP1	7.139	0.063	9.417E-13	0.100				2	2	2	9.802E-13	0.448	Liver
ENSG00000105662.15	CRTC1	7.137	0.198	9.542E-13	0.010				1	1	1	9.542E-13	0.139	Artery_Tibial
ENSG00000105662.15	CRTC1	7.137	0.253	9.542E-13	0.006				1	1	1	9.542E-13	0.109	Artery_Aorta
ENSG00000115808.11	STRN	-7.124	-0.209	1.052E-12	0.008				1	1	1	1.052E-12	0.128	Heart_Left_Ventricle
ENSG00000146247.13	PHIP	7.104	0.287	1.209E-12	0.004				1	1	1	1.209E-12	0.098	Adipose_Subcutaneou
ENSG00000146247.13	PHIP	7.104	0.285	1.209E-12	0.005				1	1	1	1.209E-12	0.099	Artery_Aorta
ENSG00000146247.13	PHIP	7.104	0.331	1.209E-12	0.004				1	1	1	1.209E-12	0.085	Adipose_Visceral_Om
ENSG00000146247.13	PHIP	7.104	0.391	1.209E-12	0.002				1	1	1	1.209E-12	0.072	Whole_Blood
ENSG00000146247.13	PHIP	7.104	0.303	1.209E-12	0.004				1	1	1	1.209E-12	0.093	Heart_Atrial_Appendag
ENSG00000146247.13	PHIP	7.060	0.232	1.662E-12	0.007				1	1	1	1.662E-12	0.120	Heart_Left_Ventricle
ENSG00000146243.13	IRAK1BP1	7.060	0.067	1.665E-12	0.086				2	2	2	9.802E-13	0.391	Artery_Aorta
ENSG00000204387.12	C6orf48	6.991	0.156	2.729E-12	0.012				2	2	2	4.770E-14	0.219	Adipose_Visceral_Om
ENSG00000166348.18	USP54	6.990	0.234	2.754E-12	0.006				1	1	1	2.754E-12	0.237	Artery_Coronary
ENSG00000166348.18	USP54	6.990	0.221	2.754E-12	0.006				1	1	1	2.754E-12	0.252	Heart_Atrial_Appendag
ENSG00000166348.18	USP54	-6.990	-0.627	2.754E-12	0.001				1	1	1	2.754E-12	0.089	Whole_Blood
ENSG00000166348.18	USP54	6.990	1.434	2.754E-12	0.000				1	1	1	2.754E-12	0.039	Kidney_Cortex
ENSG00000166348.18	USP54	6.990	0.936	2.754E-12	0.000				1	1	1	2.754E-12	0.059	Liver
ENSG00000146243.13	IRAK1BP1	6.989	0.081	2.759E-12	0.058				3	3	3	9.802E-13	0.327	Adipose_Visceral_Om
ENSG00000204277.1	LINC01993	6.955	0.084	3.536E-12	0.058				3	3	3	3.492E-12	0.327	Kidney_Cortex
ENSG00000122861.15	PLAU	-6.951	-0.131	3.620E-12	0.028				2	2	2	9.854E-14	0.448	Heart_Atrial_Appendag
ENSG00000182240.15	BACE2	-6.943	-0.058	3.850E-12	0.123				3	3	3	2.892E-11	0.386	Artery_Aorta
ENSG00000055483.19	USP36	6.938	0.438	3.982E-12	0.002				2	2	2	7.030E-13	0.050	Whole_Blood
ENSG00000141458.12	NPC1	-6.937	-0.048	4.015E-12	0.150				3	3	3	4.406E-14	0.258	Artery_Tibial
ENSG00000146243.13	IRAK1BP1	6.893	0.065	5.454E-12	0.093				2	2	2	9.802E-13	0.362	Heart_Left_Ventricle
ENSG00000186510.11	CLCNKA	-6.854	-0.058	7.161E-12	0.110				1	1	1	7.161E-12	0.467	Artery_Aorta
ENSG00000186510.11	CLCNKA	-6.854	-0.068	7.161E-12	0.088				1	1	1	7.161E-12	0.399	Artery_Coronary
ENSG00000166348.18	USP54	6.791	0.157	1.112E-11	0.018				2	2	2	2.754E-12	0.414	Artery_Aorta
ENSG00000146243.13	IRAK1BP1	6.744	0.068	1.545E-11	0.085				2	2	2	9.802E-13	0.382	Artery_Coronary
ENSG00000124574.14	ABCC10	-6.711	-0.537	1.937E-11	0.001				1	1	1	1.937E-11	0.053	Heart_Left_Ventricle

gene	gene_name	zscore	effect_size	pvalue	var_g	pred_	pred	pred_	n_snps_used	n_snps_in_cov	n_snps_in_model	best_gwas_p	largest_weight	Tissue
ENSG00000108669.16	CYTH1	6.701	0.118	2.068E-11	0.026				2	2	2	5.427E-14	0.157	Heart_Atrial_Appendag
ENSG00000146243.13	IRAK1BP1	6.688	0.073	2.262E-11	0.063				2	2	2	9.802E-13	0.350	Artery_Tibial
ENSG00000055483.19	USP36	-6.653	-0.084	2.881E-11	0.051				3	3	3	2.236E-10	0.313	Artery_Aorta
ENSG00000182240.15	BACE2	-6.652	-0.058	2.892E-11	0.121				1	1	1	2.892E-11	0.483	Heart_Atrial_Appendag
ENSG00000182240.15	BACE2	-6.652	-0.050	2.892E-11	0.150				1	1	1	2.892E-11	0.556	Artery_Coronary
ENSG00000134490.13	TMEM241	-6.645	-0.085	3.025E-11	0.051				2	2	2	6.000E-12	0.188	Heart_Atrial_Appendag
ENSG00000213551.4	DNAJC9	6.644	0.167	3.055E-11	0.015				2	2	2	8.768E-11	0.241	Kidney_Cortex
ENSG00000228727.8	SAPCD1	6.637	1.821	3.212E-11	0.000				1	1	1	3.212E-11	0.019	Artery_Tibial
ENSG00000182240.15	BACE2	-6.621	-0.031	3.565E-11	0.410				3	3	3	2.892E-11	0.885	Artery_Tibial
ENSG00000204438.10	GPANK1	-6.615	-0.231	3.705E-11	0.007				2	2	2	2.351E-07	0.095	Heart_Atrial_Appendag
ENSG00000055483.19	USP36	-6.596	-0.152	4.214E-11	0.015				1	1	1	4.214E-11	0.174	Heart_Atrial_Appendag
ENSG00000213551.4	DNAJC9	6.596	0.106	4.227E-11	0.025				2	2	2	8.768E-11	0.305	Heart_Left_Ventricle
ENSG00000055483.19	USP36	-6.590	-0.091	4.399E-11	0.041				2	2	2	2.295E-11	0.280	Artery_Tibial
ENSG00000166348.18	USP54	6.583	0.143	4.596E-11	0.016				2	2	2	2.754E-12	0.217	Heart_Left_Ventricle
ENSG00000204387.12	C6orf48	6.561	0.154	5.331E-11	0.011				1	1	1	5.331E-11	0.226	Artery_Tibial
ENSG00000146243.13	IRAK1BP1	6.561	0.067	5.350E-11	0.075				2	2	2	9.802E-13	0.367	Heart_Atrial_Appendag
ENSG00000108379.9	WNT3	6.546	0.066	5.928E-11	0.090				2	2	2	9.188E-09	0.392	Artery_Coronary
ENSG00000055483.19	USP36	-6.539	-0.133	6.181E-11	0.019				2	2	2	2.295E-11	0.198	Heart_Left_Ventricle
ENSG00000145194.17	ECE2	6.533	0.068	6.442E-11	0.070				1	1	1	6.442E-11	0.376	Heart_Atrial_Appendag
ENSG00000166348.18	USP54	6.511	0.088	7.481E-11	0.040				2	2	2	2.754E-12	0.422	Artery_Tibial
ENSG00000204356.13	NELFE	6.502	0.222	7.945E-11	0.006				3	3	3	6.531E-09	0.204	Heart_Atrial_Appendag
ENSG00000272791.1	RP11-464F9.22	6.492	0.691	8.473E-11	0.001				2	2	2	3.653E-12	0.053	Kidney_Cortex
ENSG00000138286.14	FAM149B1	-6.487	-0.202	8.745E-11	0.007				1	1	1	8.745E-11	0.270	Whole_Blood
ENSG00000204387.12	C6orf48	6.474	0.339	9.534E-11	0.002				2	2	2	5.331E-11	0.104	Heart_Atrial_Appendag
ENSG00000166348.18	USP54	6.465	0.125	1.012E-10	0.021				2	2	2	2.754E-12	0.309	Adipose_Visceral_Om
ENSG00000166348.18	USP54	6.434	0.126	1.240E-10	0.018				2	2	2	2.754E-12	0.326	Adipose_Subcutaneou
ENSG00000171634.16	BPTF	-6.425	-0.174	1.322E-10	0.011				2	2	2	4.641E-10	0.118	Heart_Atrial_Appendag
ENSG00000185056.9	C5orf47	-6.410	-0.093	1.452E-10	0.037				1	1	1	1.452E-10	0.302	Heart_Atrial_Appendag
ENSG00000116809.11	ZBTB17	6.409	7.228	1.463E-10	0.000				1	1	1	1.463E-10	0.004	Whole_Blood
ENSG00000116809.11	ZBTB17	-6.409	-207.530	1.463E-10	0.000				1	1	1	1.463E-10	0.000	Liver
ENSG00000116809.11	ZBTB17	6.409	5.466	1.463E-10	0.000				1	1	1	1.463E-10	0.005	Artery_Aorta
ENSG00000116809.11	ZBTB17	6.409	26.035	1.463E-10	0.000				1	1	1	1.463E-10	0.001	Heart_Atrial_Appendag
ENSG00000148926.9	ADM	-6.394	-1.976	1.615E-10	0.000				1	1	1	1.615E-10	0.019	Adipose_Visceral_Om
ENSG00000166321.13	NUDT13	6.372	0.022	1.863E-10	0.557				2	2	2	7.379E-11	1.256	Artery_Coronary
ENSG00000108669.16	CYTH1	6.358	0.115	2.049E-10	0.025				2	2	2	1.695E-12	0.160	Heart_Left_Ventricle
ENSG00000138286.14	FAM149B1	-6.354	-0.057	2.097E-10	0.077				2	2	2	1.967E-10	0.541	Adipose_Subcutaneou
ENSG00000166321.13	NUDT13	6.352	0.075	2.130E-10	0.044				2	2	2	4.003E-10	0.716	Heart_Left_Ventricle

gene	gene_name	zscore	effect_size	pvalue	var_g	pred_	pred	pred_	n_snps_used	n_snps_in_cov	n_snps_in_model	best_gwas_p	largest_weight	Tissue
ENSG00000055483.19	USP36	-6.344	-0.096	2.236E-10	0.033				1	1	1	2.236E-10	0.261	Artery_Coronary
ENSG00000136379.11	ABHD17C	6.335	0.113	2.376E-10	0.024				2	2	2	2.399E-10	0.220	Heart_Atrial_Appendag
ENSG00000111785.19	RIC8B	6.327	0.266	2.507E-10	0.004				2	2	2	2.833E-10	0.097	Heart_Atrial_Appendag
ENSG00000147883.10	CDKN2B	-6.326	-2.282	2.518E-10	0.000				1	1	1	2.518E-10	0.011	Kidney_Cortex
ENSG00000147883.10	CDKN2B	6.326	3.186	2.518E-10	0.000				1	1	1	2.518E-10	0.008	Artery_Tibial
ENSG00000147883.10	CDKN2B	-6.326	-2.452	2.518E-10	0.000				1	1	1	2.518E-10	0.010	Artery_Coronary
ENSG00000055483.19	USP36	-6.317	-0.187	2.668E-10	0.009				2	2	2	4.214E-11	0.131	Adipose_Subcutaneou
ENSG00000156042.17	CFAP70	6.317	0.119	2.672E-10	0.018				1	1	1	2.672E-10	0.438	Whole_Blood
ENSG00000119326.14	CTNNAL1	6.316	0.093	2.684E-10	0.038				3	3	3	1.880E-09	0.187	Heart_Atrial_Appendag
ENSG00000171634.16	BPTF	-6.313	-0.236	2.728E-10	0.007				2	2	2	4.641E-10	0.076	Artery_Aorta
ENSG00000156042.17	CFAP70	6.292	0.070	3.137E-10	0.049				2	2	2	2.368E-10	0.403	Heart_Atrial_Appendag
ENSG00000213551.4	DNAJC9	6.291	0.278	3.146E-10	0.003				1	1	1	3.146E-10	0.175	Artery_Tibial
ENSG00000213551.4	DNAJC9	6.291	0.273	3.146E-10	0.003				1	1	1	3.146E-10	0.178	Adipose_Subcutaneou
ENSG00000213551.4	DNAJC9	6.291	0.233	3.146E-10	0.005				1	1	1	3.146E-10	0.209	Artery_Aorta
ENSG00000213551.4	DNAJC9	6.291	0.374	3.146E-10	0.002				1	1	1	3.146E-10	0.130	Adipose_Visceral_Om
ENSG00000213551.4	DNAJC9	6.291	0.672	3.146E-10	0.001				1	1	1	3.146E-10	0.072	Whole_Blood
ENSG00000213551.4	DNAJC9	6.291	0.234	3.146E-10	0.005				1	1	1	3.146E-10	0.208	Artery_Coronary
ENSG00000111785.19	RIC8B	6.289	0.302	3.187E-10	0.004				2	2	2	2.833E-10	0.083	Adipose_Visceral_Om
ENSG00000133805.15	AMPD3	-6.289	-15.087	3.199E-10	0.000				1	1	1	3.199E-10	0.002	Liver
ENSG00000112137.17	PHACTR1	-6.269	-0.087	3.639E-10	0.037				1	1	1	3.639E-10	0.275	Artery_Coronary
ENSG00000138279.15	ANXA7	6.266	2.206	3.700E-10	0.000				1	1	1	3.700E-10	0.023	Adipose_Subcutaneou
ENSG00000138279.15	ANXA7	6.266	3.399	3.700E-10	0.000				1	1	1	3.700E-10	0.015	Heart_Left_Ventricle
ENSG00000138279.15	ANXA7	6.266	21.635	3.700E-10	0.000				1	1	1	3.700E-10	0.002	Artery_Aorta
ENSG00000138279.15	ANXA7	6.266	1.991	3.700E-10	0.000				1	1	1	3.700E-10	0.026	Heart_Atrial_Appendag
ENSG00000138279.15	ANXA7	6.266	4.793	3.700E-10	0.000				1	1	1	3.700E-10	0.011	Artery_Coronary
ENSG00000138279.15	ANXA7	6.266	3.985	3.700E-10	0.000				1	1	1	3.700E-10	0.013	Adipose_Visceral_Om
ENSG00000138279.15	ANXA7	-6.266	-40.918	3.700E-10	0.000				1	1	1	3.700E-10	0.001	Whole_Blood
ENSG00000138279.15	ANXA7	-6.266	-2.419	3.700E-10	0.000				1	1	1	3.700E-10	0.021	Liver
ENSG00000138279.15	ANXA7	-6.266	-1.873	3.700E-10	0.000				1	1	1	3.700E-10	0.028	Kidney_Cortex
ENSG00000138279.15	ANXA7	6.266	2.570	3.700E-10	0.000				1	1	1	3.700E-10	0.020	Artery_Tibial
ENSG00000112137.17	PHACTR1	-6.262	-0.077	3.807E-10	0.045				2	2	2	3.639E-10	0.312	Artery_Tibial
ENSG00000166321.13	NUDT13	6.254	0.038	4.003E-10	0.170				1	1	1	4.003E-10	1.380	Artery_Tibial
ENSG00000166321.13	NUDT13	6.254	0.059	4.003E-10	0.103				1	1	1	4.003E-10	0.883	Kidney_Cortex
ENSG00000213551.4	DNAJC9	6.250	0.796	4.106E-10	0.000				2	2	2	8.768E-11	0.063	Liver
ENSG00000141458.12	NPC1	-6.238	-0.026	4.424E-10	0.412				3	3	3	3.293E-12	0.381	Heart_Left_Ventricle
ENSG00000166321.13	NUDT13	6.230	0.040	4.672E-10	0.152				2	2	2	4.003E-10	0.709	Heart_Atrial_Appendag
ENSG00000108379.9	WNT3	6.224	0.040	4.835E-10	0.205				2	2	2	7.497E-09	0.538	Adipose_Subcutaneou

gene	gene_name	zscore	effect_size	pvalue	var_g	pred	pred	pred	n_snps_used	n_snps_in_cov	n_snps_in_model	best_gwas_p	largest_weight	Tissue
ENSG00000146247.13	PHIP	6.223	0.354	4.886E-10	0.002				1	1	1	4.886E-10	0.075	Artery_Tibial
ENSG00000115486.11	GGCX	6.201	0.146	5.622E-10	0.012				1	1	1	5.622E-10	0.168	Liver
ENSG00000115486.11	GGCX	6.201	0.063	5.622E-10	0.068				1	1	1	5.622E-10	0.390	Artery_Aorta
ENSG00000115486.11	GGCX	6.201	0.069	5.622E-10	0.055				1	1	1	5.622E-10	0.354	Heart_Left_Ventricle
ENSG00000112137.17	PHACTR1	-6.187	-0.085	6.124E-10	0.034				2	2	2	3.639E-10	0.272	Artery_Aorta
ENSG00000204387.12	C6orf48	6.165	0.114	7.041E-10	0.019				2	2	2	3.152E-10	0.258	Adipose_Subcutaneou
ENSG00000115486.11	GGCX	6.131	0.040	8.744E-10	0.173				2	2	2	2.215E-09	0.434	Whole_Blood
ENSG00000141736.13	ERBB2	6.126	0.120	9.023E-10	0.020				3	3	3	8.314E-10	0.123	Heart_Left_Ventricle
ENSG00000166321.13	NUDT13	6.124	0.023	9.153E-10	0.587				2	2	2	8.071E-10	1.175	Artery_Aorta
ENSG00000113580.14	NR3C1	-6.109	-0.633	1.003E-09	0.001				1	1	1	1.003E-09	0.050	Whole_Blood
ENSG00000272391.5	POM121C	6.109	0.104	1.006E-09	0.045				2	3	3	2.305E-10	0.240	Artery_Aorta
ENSG00000141736.13	ERBB2	6.105	0.476	1.028E-09	0.001				2	2	2	8.314E-10	0.052	Adipose_Subcutaneou
ENSG00000118655.4	DCLRE1B	-6.078	-0.139	1.215E-09	0.013				2	2	2	5.759E-09	0.123	Artery_Tibial
ENSG00000156042.17	CFAP70	6.075	0.107	1.239E-09	0.024				1	1	1	1.239E-09	0.452	Artery_Aorta
ENSG00000156042.17	CFAP70	6.075	0.139	1.239E-09	0.013				1	1	1	1.239E-09	0.350	Heart_Left_Ventricle
ENSG00000156042.17	CFAP70	6.075	0.110	1.239E-09	0.022				1	1	1	1.239E-09	0.442	Adipose_Visceral_Om
ENSG00000156042.17	CFAP70	6.075	0.099	1.239E-09	0.025				1	1	1	1.239E-09	0.490	Artery_Tibial
ENSG00000105974.11	CAV1	-6.066	-0.503	1.315E-09	0.001				3	3	3	1.391E-09	0.045	Heart_Atrial_Appendag
ENSG00000156042.17	CFAP70	6.063	0.109	1.339E-09	0.021				2	2	2	1.239E-09	0.445	Artery_Coronary
ENSG00000156042.17	CFAP70	6.058	0.103	1.375E-09	0.018				2	2	2	1.239E-09	0.469	Liver
ENSG00000224477.5	RP1-81D8.3	-6.058	-0.058	1.382E-09	0.096				2	2	2	3.720E-11	0.406	Liver
ENSG00000272391.5	POM121C	6.041	0.086	1.533E-09	0.065				2	3	3	2.305E-10	0.343	Whole_Blood
ENSG00000111785.19	RIC8B	6.036	0.343	1.576E-09	0.002				2	2	2	1.569E-09	0.072	Adipose_Subcutaneou
ENSG00000111785.19	RIC8B	6.035	0.393	1.591E-09	0.002				2	2	2	1.569E-09	0.062	Heart_Left_Ventricle
ENSG00000148660.20	CAMK2G	6.030	0.395	1.639E-09	0.002				1	1	1	1.639E-09	0.061	Adipose_Subcutaneou
ENSG00000141736.13	ERBB2	6.018	0.511	1.769E-09	0.001				2	2	2	8.314E-10	0.047	Adipose_Visceral_Om
ENSG00000272630.1	RP11-344N10.5	-6.003	-0.151	1.938E-09	0.020				1	1	1	1.938E-09	0.353	Kidney_Cortex
ENSG00000025039.14	RRAGD	5.993	0.229	2.064E-09	0.005				1	2	2	2.064E-09	0.100	Adipose_Subcutaneou
ENSG00000151135.9	TMEM263	5.984	0.665	2.181E-09	0.001				1	1	1	2.181E-09	0.037	Whole_Blood
ENSG00000151135.9	TMEM263	5.984	0.164	2.181E-09	0.011				1	1	1	2.181E-09	0.149	Artery_Coronary
ENSG00000166321.13	NUDT13	5.979	0.023	2.243E-09	0.598				2	2	2	2.442E-09	1.335	Adipose_Subcutaneou
ENSG00000039650.11	PNKP	-5.973	-0.231	2.329E-09	0.006				2	2	2	2.163E-09	0.107	Liver
ENSG00000141744.3	PNMT	5.973	0.128	2.332E-09	0.020				2	2	2	3.566E-09	0.194	Heart_Atrial_Appendag
ENSG00000166321.13	NUDT13	5.965	0.055	2.442E-09	0.079				1	1	1	2.442E-09	0.866	Whole_Blood
ENSG00000166321.13	NUDT13	5.965	0.049	2.442E-09	0.106				1	2	2	2.442E-09	0.983	Adipose_Visceral_Om
ENSG00000204421.2	LY6G6C	-5.952	-0.224	2.655E-09	0.004				1	1	1	2.655E-09	0.118	Artery_Aorta
ENSG00000204421.2	LY6G6C	-5.952	-0.278	2.655E-09	0.003				1	1	1	2.655E-09	0.095	Kidney_Cortex

gene	gene_name	zscore	effect_size	pvalue	var_g	pred	pred	pred	n_snps_used	n_snps_in_cov	n_snps_in_model	best_gwas_p	largest_weight	Tissue
ENSG00000204421.2	LY6G6C	5.952	1.842	2.655E-09	0.000				1	1	1	2.655E-09	0.014	Whole_Blood
ENSG00000204421.2	LY6G6C	-5.952	-0.403	2.655E-09	0.001				1	1	1	2.655E-09	0.066	Adipose_Subcutaneou
ENSG00000204421.2	LY6G6C	-5.952	-0.280	2.655E-09	0.003				1	1	1	2.655E-09	0.095	Artery_Coronary
ENSG00000141736.13	ERBB2	5.939	1.207	2.866E-09	0.000				2	2	2	8.314E-10	0.019	Whole_Blood
ENSG00000010310.8	GIPR	-5.937	-0.706	2.910E-09	0.001				1	1	1	2.910E-09	0.054	Artery_Tibial
ENSG00000161395.13	PGAP3	5.936	0.371	2.923E-09	0.002				1	2	2	2.923E-09	0.069	Whole_Blood
ENSG00000138286.14	FAM149B1	-5.926	-0.266	3.110E-09	0.005				1	1	1	3.110E-09	0.179	Kidney_Cortex
ENSG00000138286.14	FAM149B1	-5.926	-0.246	3.110E-09	0.004				1	1	1	3.110E-09	0.194	Artery_Aorta
ENSG00000141736.13	ERBB2	5.909	0.217	3.451E-09	0.007				2	2	2	3.062E-09	0.113	Heart_Atrial_Appendag
ENSG00000141744.3	PNMT	5.903	0.149	3.566E-09	0.014				1	1	1	3.566E-09	0.170	Whole_Blood
ENSG00000141744.3	PNMT	5.902	0.102	3.584E-09	0.028				2	2	2	3.566E-09	0.149	Adipose_Visceral_Om
ENSG00000151135.9	TMEM263	5.892	0.225	3.807E-09	0.005				2	2	2	2.181E-09	0.078	Heart_Left_Ventricle
ENSG00000204421.2	LY6G6C	-5.891	-1.172	3.837E-09	0.000				2	2	2	2.655E-09	0.021	Adipose_Visceral_Om
ENSG00000196141.13	SPATS2L	5.885	0.173	3.992E-09	0.009				2	2	2	4.732E-09	0.134	Adipose_Subcutaneou
ENSG00000149084.12	HSD17B12	-5.882	-0.060	4.065E-09	0.071				1	1	1	4.065E-09	0.418	Liver
ENSG00000067208.14	EVI5	5.878	0.119	4.156E-09	0.020				2	2	2	4.268E-08	0.197	Whole_Blood
ENSG00000188266.13	HYKK	-5.876	-0.300	4.203E-09	0.003				1	1	1	4.203E-09	0.077	Whole_Blood
ENSG00000115486.11	GGCX	5.873	0.095	4.274E-09	0.027				1	1	1	4.274E-09	0.250	Adipose_Visceral_Om
ENSG00000177733.6	HNRNPA0	-5.873	-1.173	4.291E-09	0.000				1	1	1	4.291E-09	0.025	Kidney_Cortex
ENSG00000141741.11	MIEN1	-5.872	-2.375	4.309E-09	0.000				1	1	1	4.309E-09	0.010	Whole_Blood
ENSG00000148297.15	MED22	5.872	1.352	4.318E-09	0.000				1	1	1	4.318E-09	0.027	Heart_Left_Ventricle
ENSG00000263001.5	GTF2I	5.870	0.102	4.347E-09	0.064				2	2	2	1.311E-08	0.428	Artery_Aorta
ENSG00000125686.11	MED1	5.869	0.550	4.386E-09	0.001				2	2	2	1.500E-08	0.042	Liver
ENSG00000196821.9	C6orf106	5.850	0.129	4.915E-09	0.015				2	2	2	8.441E-09	0.171	Heart_Left_Ventricle
ENSG00000196141.13	SPATS2L	-5.831	-0.120	5.515E-09	0.019				3	3	3	4.732E-09	0.277	Heart_Left_Ventricle
ENSG00000115486.11	GGCX	5.829	0.060	5.575E-09	0.070				3	3	3	5.622E-10	0.353	Heart_Atrial_Appendag
ENSG00000125686.11	MED1	5.825	0.780	5.713E-09	0.000				2	2	2	1.500E-08	0.032	Heart_Left_Ventricle
ENSG00000118655.4	DCLRE1B	-5.824	-0.172	5.759E-09	0.008				1	1	1	5.759E-09	0.171	Adipose_Subcutaneou
ENSG00000118655.4	DCLRE1B	-5.824	-0.192	5.759E-09	0.008				1	1	1	5.759E-09	0.154	Liver
ENSG00000118655.4	DCLRE1B	-5.824	-0.297	5.759E-09	0.003				1	1	1	5.759E-09	0.099	Whole_Blood
ENSG00000125686.11	MED1	5.817	0.578	5.996E-09	0.001				2	2	2	1.500E-08	0.039	Whole_Blood
ENSG00000125686.11	MED1	5.816	0.897	6.015E-09	0.000				2	2	2	1.500E-08	0.027	Heart_Atrial_Appendag
ENSG00000111785.19	RIC8B	5.815	0.432	6.072E-09	0.001				2	2	2	1.569E-09	0.074	Whole_Blood
ENSG00000204498.10	NFKBIL1	5.808	0.212	6.315E-09	0.004				3	3	3	9.922E-07	0.224	Adipose_Subcutaneou
ENSG00000125686.11	MED1	5.804	0.539	6.468E-09	0.001				2	2	2	1.500E-08	0.039	Kidney_Cortex
ENSG00000204356.13	NELFE	5.803	1.154	6.531E-09	0.000				1	1	1	6.531E-09	0.044	Adipose_Visceral_Om
ENSG00000125686.11	MED1	5.799	0.944	6.657E-09	0.000				2	2	2	1.500E-08	0.026	Artery_Tibial

gene	gene_name	zscore	effect_size	pvalue	var_g	pred	pred	pred	n_snps_used	n_snps_in_cov	n_snps_in_model	best_gwas_p	largest_weight	Tissue
ENSG00000204314.10	PRRT1	-5.799	-0.418	6.666E-09	0.002				3	3	3	1.240E-05	0.076	Artery_Tibial
ENSG00000149084.12	HSD17B12	-5.795	-0.034	6.832E-09	0.215				3	3	3	2.735E-08	0.736	Artery_Coronary
ENSG00000125686.11	MED1	5.793	0.710	6.902E-09	0.001				2	2	2	1.500E-08	0.033	Adipose_Visceral_Om
ENSG00000115486.11	GGCX	5.790	0.039	7.038E-09	0.157				3	3	3	8.116E-10	0.344	Artery_Tibial
ENSG00000125686.11	MED1	5.787	0.575	7.171E-09	0.001				2	2	2	1.500E-08	0.041	Adipose_Subcutaneou
ENSG00000149084.12	HSD17B12	-5.772	-0.030	7.853E-09	0.291				2	2	2	3.128E-08	0.848	Artery_Tibial
ENSG00000149084.12	HSD17B12	-5.771	-0.045	7.900E-09	0.115				2	2	2	2.735E-08	0.556	Heart_Atrial_Appendag
ENSG00000085831.15	TTC39A	-5.770	-2.019	7.913E-09	0.000				2	2	2	8.419E-13	0.027	Artery_Tibial
ENSG00000115486.11	GGCX	5.769	0.077	7.971E-09	0.038				2	2	2	5.622E-10	0.275	Adipose_Subcutaneou
ENSG00000119326.14	CTNNAL1	5.767	0.070	8.067E-09	0.058				3	3	3	1.894E-07	0.635	Liver
ENSG00000146147.14	MLIP	-5.766	-0.075	8.097E-09	0.046				1	1	1	8.097E-09	0.331	Adipose_Subcutaneou
ENSG00000146215.13	CRIP3	5.760	0.064	8.422E-09	0.066				2	2	2	7.262E-08	0.273	Liver
ENSG00000204310.12	AGPAT1	5.760	0.048	8.430E-09	0.118				1	1	1	8.430E-09	0.487	Liver
ENSG00000196821.9	C6orf106	5.759	0.142	8.441E-09	0.009				1	1	1	8.441E-09	0.186	Kidney_Cortex
ENSG00000196821.9	C6orf106	5.759	0.222	8.441E-09	0.005				1	2	2	8.441E-09	0.119	Artery_Coronary
ENSG00000112164.5	GLP1R	-5.759	-0.175	8.444E-09	0.009				1	1	1	8.444E-09	0.130	Adipose_Subcutaneou
ENSG00000112164.5	GLP1R	-5.759	-0.198	8.444E-09	0.007				1	1	1	8.444E-09	0.115	Adipose_Visceral_Om
ENSG00000139722.6	VPS37B	5.757	0.077	8.537E-09	0.031				1	1	1	8.537E-09	0.447	Artery_Coronary
ENSG00000139722.6	VPS37B	5.757	0.196	8.537E-09	0.005				1	1	1	8.537E-09	0.176	Heart_Left_Ventricle
ENSG00000031003.10	FAM13B	-5.754	-0.071	8.691E-09	0.057				3	3	3	3.958E-06	0.291	Heart_Atrial_Appendag
ENSG00000119326.14	CTNNAL1	5.753	0.046	8.790E-09	0.130				4	4	4	7.568E-07	0.639	Artery_Aorta
ENSG00000055483.19	USP36	-5.745	-0.139	9.204E-09	0.025				2	2	2	4.933E-09	0.220	Liver
ENSG00000161395.13	PGAP3	5.744	0.039	9.246E-09	0.176				2	2	2	2.923E-09	0.332	Heart_Left_Ventricle
ENSG00000108306.11	FBXL20	-5.737	-0.166	9.622E-09	0.013				2	2	2	1.191E-08	0.177	Artery_Coronary
ENSG00000125686.11	MED1	5.725	1.163	1.037E-08	0.000				2	2	2	1.500E-08	0.020	Artery_Coronary
ENSG00000185519.8	FAM131C	5.707	1.423	1.148E-08	0.000				1	1	1	1.148E-08	0.024	Heart_Left_Ventricle
ENSG00000149084.12	HSD17B12	-5.701	-0.040	1.193E-08	0.177				2	2	2	2.847E-08	0.693	Kidney_Cortex
ENSG00000149084.12	HSD17B12	-5.700	-0.030	1.195E-08	0.301				3	3	3	3.128E-08	0.820	Adipose_Subcutaneou
ENSG00000263001.5	GTF2I	5.685	0.101	1.311E-08	0.062				1	1	1	1.311E-08	0.433	Adipose_Subcutaneou
ENSG00000263001.5	GTF2I	5.685	0.142	1.311E-08	0.031				1	1	1	1.311E-08	0.309	Whole_Blood
ENSG00000263001.5	GTF2I	5.685	0.114	1.311E-08	0.046				1	1	1	1.311E-08	0.383	Artery_Tibial
ENSG00000263001.5	GTF2I	5.685	0.195	1.311E-08	0.016				1	1	1	1.311E-08	0.224	Heart_Left_Ventricle
ENSG00000196275.13	GTF2IRD2	5.685	0.077	1.311E-08	0.111				1	2	2	1.311E-08	0.569	Liver
ENSG00000263001.5	GTF2I	5.685	0.128	1.311E-08	0.040				1	1	1	1.311E-08	0.342	Liver
ENSG00000158517.13	NCF1	5.685	0.404	1.311E-08	0.004				1	2	2	1.311E-08	0.108	Whole_Blood
ENSG00000263001.5	GTF2I	5.685	0.118	1.311E-08	0.054				1	1	1	1.311E-08	0.371	Artery_Coronary
ENSG00000118655.4	DCLRE1B	-5.682	-0.232	1.333E-08	0.005				1	1	1	1.333E-08	0.109	Artery_Coronary

gene	gene_name	zscore	effect_size	pvalue	var_g	pred_	pred_	pred_	n_snps_used	n_snps_in_cov	n_snps_in_model	best_gwas_p	largest_weight	Tissue
ENSG00000108306.11	FBXL20	-5.675	-0.166	1.390E-08	0.009				2	2	2	1.614E-08	0.161	Adipose_Subcutaneou
ENSG00000176986.15	SEC24C	-5.669	-0.376	1.436E-08	0.002				3	3	3	1.433E-20	0.037	Heart_Atrial_Appendag
ENSG00000233276.3	GPX1	5.667	0.247	1.455E-08	0.004				2	2	2	1.834E-06	0.065	Whole_Blood
ENSG00000149084.12	HSD17B12	-5.666	-0.031	1.465E-08	0.268				2	2	2	3.815E-08	0.785	Artery_Aorta
ENSG00000169016.16	E2F6	5.663	0.124	1.490E-08	0.018				1	1	1	1.490E-08	0.195	Heart_Atrial_Appendag
ENSG00000149084.12	HSD17B12	-5.660	-0.032	1.515E-08	0.249				2	2	2	3.128E-08	0.772	Adipose_Visceral_Om
ENSG00000166822.12	TMEM170A	-5.659	-0.138	1.518E-08	0.013				3	3	3	5.946E-08	0.103	Adipose_Subcutaneou
ENSG00000213551.4	DNAJC9	5.643	0.153	1.668E-08	0.011				1	1	1	1.668E-08	0.336	Heart_Atrial_Appendag
ENSG00000180828.2	BHLHE22	5.640	0.319	1.702E-08	0.003				1	1	1	1.702E-08	0.077	Artery_Aorta
ENSG00000154305.16	MIA3	-5.629	-0.223	1.818E-08	0.004				2	2	2	4.408E-08	0.106	Adipose_Visceral_Om
ENSG00000106771.12	TMEM245	-5.627	-0.097	1.830E-08	0.028				2	2	2	6.803E-09	0.158	Liver
ENSG00000146215.13	CRIP3	5.626	0.067	1.849E-08	0.060				2	2	2	7.262E-08	0.262	Heart_Left_Ventricle
ENSG00000204310.12	AGPAT1	-5.625	-0.379	1.860E-08	0.002				2	2	2	2.395E-07	0.064	Whole_Blood
ENSG00000108306.11	FBXL20	-5.622	-0.123	1.884E-08	0.016				2	2	2	1.888E-08	0.213	Artery_Aorta
ENSG00000196126.11	HLA-DRB1	-5.619	-0.096	1.918E-08	0.094				2	3	3	1.993E-09	0.425	Adipose_Subcutaneou
ENSG00000197696.9	NMB	-5.618	-0.056	1.928E-08	0.076				1	1	1	1.928E-08	0.438	Liver
ENSG00000197696.9	NMB	-5.618	-0.092	1.928E-08	0.028				1	1	1	1.928E-08	0.266	Artery_Tibial
ENSG00000197696.9	NMB	-5.618	-0.065	1.928E-08	0.059				1	1	1	1.928E-08	0.375	Artery_Aorta
ENSG00000197696.9	NMB	-5.618	-0.099	1.928E-08	0.025				1	1	1	1.928E-08	0.246	Heart_Atrial_Appendag
ENSG00000197696.9	NMB	-5.618	-0.106	1.928E-08	0.021				1	1	1	1.928E-08	0.231	Artery_Coronary
ENSG00000197696.9	NMB	-5.618	-0.155	1.928E-08	0.010				1	1	1	1.928E-08	0.158	Adipose_Visceral_Om
ENSG00000197696.9	NMB	-5.618	-0.099	1.928E-08	0.025				1	1	1	1.928E-08	0.248	Heart_Left_Ventricle
ENSG00000182180.13	MRPS16	-5.605	-0.253	2.081E-08	0.003				2	2	2	1.821E-09	0.166	Whole_Blood
ENSG00000108306.11	FBXL20	-5.603	-0.248	2.103E-08	0.004				2	2	2	1.888E-08	0.106	Adipose_Visceral_Om
ENSG00000108306.11	FBXL20	-5.603	-0.178	2.105E-08	0.007				2	2	2	1.888E-08	0.149	Heart_Left_Ventricle
ENSG00000146215.13	CRIP3	-5.601	-0.162	2.133E-08	0.010				2	2	2	7.262E-08	0.088	Artery_Tibial
ENSG00000272791.1	RP11-464F9.22	-5.599	-0.710	2.153E-08	0.001				1	1	1	2.153E-08	0.082	Heart_Left_Ventricle
ENSG00000272791.1	RP11-464F9.22	-5.599	-0.997	2.153E-08	0.000				1	1	1	2.153E-08	0.059	Artery_Tibial
ENSG00000272791.1	RP11-464F9.22	5.599	0.553	2.153E-08	0.001				1	1	1	2.153E-08	0.106	Whole_Blood
ENSG00000166822.12	TMEM170A	-5.598	-0.209	2.172E-08	0.004				2	2	2	5.946E-08	0.099	Liver
ENSG00000172992.11	DCAKD	-5.593	-0.046	2.238E-08	0.116				2	2	2	3.603E-07	0.354	Kidney_Cortex
ENSG00000204421.2	LY6G6C	-5.592	-0.172	2.239E-08	0.007				2	2	2	2.655E-09	0.208	Artery_Tibial
ENSG00000198862.13	LTN1	-5.592	-0.337	2.241E-08	0.002				2	2	2	5.417E-08	0.156	Adipose_Subcutaneou
ENSG00000146215.13	CRIP3	5.588	0.227	2.291E-08	0.005				2	2	2	7.262E-08	0.062	Whole_Blood
ENSG00000177051.5	FBXO46	-5.587	-0.385	2.308E-08	0.002				1	1	1	2.308E-08	0.074	Whole_Blood
ENSG00000168899.4	VAMP5	-5.587	-0.199	2.314E-08	0.007				1	1	1	2.314E-08	0.122	Adipose_Visceral_Om
ENSG00000204356.13	NELFE	5.580	0.098	2.402E-08	0.025				2	2	2	1.793E-08	0.386	Adipose_Subcutaneou

gene	gene_name	zscore	effect_size	pvalue	var_g	pred_	pred_	pred_	n_snps_used	n_snps_in_cov	n_snps_in_model	best_gwas_p	largest_weight	Tissue
ENSG00000149084.12	HSD17B12	-5.580	-0.034	2.403E-08	0.220				2	2	2	2.735E-08	0.688	Whole_Blood
ENSG00000182180.13	MRPS16	-5.571	-0.549	2.533E-08	0.001				2	2	2	5.984E-11	0.073	Kidney_Cortex
ENSG00000146215.13	CRIP3	-5.564	-0.156	2.642E-08	0.010				2	2	2	4.022E-06	0.147	Adipose_Subcutaneou
ENSG00000109501.13	WFS1	5.560	0.110	2.703E-08	0.018				1	2	2	2.703E-08	0.200	Heart_Atrial_Appendag
ENSG00000182180.13	MRPS16	-5.559	-0.224	2.709E-08	0.004				3	3	3	5.984E-11	0.113	Heart_Atrial_Appendag
ENSG00000108306.11	FBXL20	-5.557	-0.191	2.742E-08	0.007				2	2	2	1.888E-08	0.136	Heart_Atrial_Appendag
ENSG00000272791.1	RP11-464F9.22	5.555	2.686	2.773E-08	0.000				1	1	1	2.773E-08	0.022	Liver
ENSG00000272791.1	RP11-464F9.22	-5.555	-2.286	2.773E-08	0.000				1	1	1	2.773E-08	0.025	Artery_Aorta
ENSG00000272791.1	RP11-464F9.22	-5.555	-0.680	2.773E-08	0.001				1	1	1	2.773E-08	0.085	Heart_Atrial_Appendag
ENSG00000272791.1	RP11-464F9.22	-5.555	-2.747	2.773E-08	0.000				1	1	1	2.773E-08	0.021	Adipose_Subcutaneou
ENSG00000146215.13	CRIP3	5.553	0.086	2.812E-08	0.028				2	2	2	7.262E-08	0.165	Kidney_Cortex
ENSG00000141744.3	PNMT	5.552	0.120	2.826E-08	0.018				1	1	1	2.826E-08	0.200	Heart_Left_Ventricle
ENSG00000205213.13	LGR4	-5.535	-0.184	3.106E-08	0.008				3	3	3	5.043E-07	0.105	Artery_Tibial
ENSG00000273559.4	CWC25	5.531	0.818	3.180E-08	0.000				1	1	1	3.180E-08	0.032	Kidney_Cortex
ENSG00000122882.10	ECD	5.529	2.204	3.216E-08	0.000				1	1	1	3.216E-08	0.026	Whole_Blood
ENSG00000138286.14	FAM149B1	-5.529	-0.365	3.216E-08	0.002				1	1	1	3.216E-08	0.156	Heart_Left_Ventricle
ENSG00000122882.10	ECD	5.529	0.255	3.216E-08	0.004				1	1	1	3.216E-08	0.223	Adipose_Subcutaneou
ENSG00000138286.14	FAM149B1	-5.529	-0.312	3.216E-08	0.003				1	1	1	3.216E-08	0.183	Heart_Atrial_Appendag
ENSG00000122882.10	ECD	5.529	0.189	3.216E-08	0.008				1	1	1	3.216E-08	0.301	Heart_Atrial_Appendag
ENSG00000138286.14	FAM149B1	-5.529	-0.177	3.216E-08	0.010				1	1	1	3.216E-08	0.322	Adipose_Visceral_Om
ENSG00000138286.14	FAM149B1	-5.529	-0.312	3.216E-08	0.003				1	1	1	3.216E-08	0.182	Artery_Coronary
ENSG00000122882.10	ECD	5.529	0.225	3.216E-08	0.007				1	1	1	3.216E-08	0.253	Artery_Coronary
ENSG00000122882.10	ECD	5.529	1.096	3.216E-08	0.000				1	1	1	3.216E-08	0.052	Liver
ENSG00000138286.14	FAM149B1	-5.529	-0.120	3.216E-08	0.015				1	1	1	3.216E-08	0.475	Liver
ENSG00000151135.9	TMEM263	5.528	0.139	3.241E-08	0.014				2	2	2	3.090E-09	0.110	Adipose_Visceral_Om
ENSG00000108306.11	FBXL20	-5.524	-0.145	3.308E-08	0.012				2	2	2	3.420E-08	0.178	Artery_Tibial
ENSG00000168899.4	VAMP5	-5.517	-0.152	3.448E-08	0.011				2	2	2	2.314E-08	0.137	Heart_Left_Ventricle
ENSG00000077522.12	ACTN2	5.511	0.168	3.565E-08	0.007				2	2	2	9.178E-08	0.124	Heart_Atrial_Appendag
ENSG00000151617.15	EDNRA	5.501	0.142	3.775E-08	0.012				2	2	2	5.735E-07	0.172	Liver
ENSG00000146243.13	IRAK1BP1	5.498	0.066	3.835E-08	0.052				2	2	2	7.284E-08	0.313	Kidney_Cortex
ENSG00000050426.15	LETMD1	-5.495	-0.070	3.906E-08	0.048				1	1	1	3.906E-08	0.306	Heart_Left_Ventricle
ENSG00000148290.9	SURF1	-5.491	-0.030	3.986E-08	0.526				5	5	5	6.219E-15	0.509	Heart_Atrial_Appendag
ENSG00000141458.12	NPC1	-5.491	-0.039	4.000E-08	0.157				2	2	2	2.128E-09	0.512	Adipose_Subcutaneou
ENSG00000091732.15	ZC3HC1	5.490	0.353	4.015E-08	0.002				2	2	2	2.792E-08	0.064	Heart_Atrial_Appendag
ENSG00000204472.12	AIF1	5.482	0.283	4.200E-08	0.003				2	2	2	5.555E-08	0.069	Adipose_Visceral_Om
ENSG00000067208.14	EVI5	5.479	0.246	4.268E-08	0.004				1	1	1	4.268E-08	0.143	Artery_Coronary
ENSG00000067208.14	EVI5	5.479	0.169	4.268E-08	0.007				1	1	1	4.268E-08	0.208	Heart_Atrial_Appendag

gene	gene_name	zscore	effect_size	pvalue	var_g	pred_	pred_	pred_	n_snps_used	n_snps_in_cov	n_snps_in_model	best_gwas_p	largest_weight	Tissue
ENSG00000204435.13	CSNK2B	-5.475	-0.605	4.370E-08	0.001				2	2	2	1.060E-07	0.047	Artery_Coronary
ENSG00000187492.8	CDHR4	5.468	0.125	4.544E-08	0.016				1	1	1	4.544E-08	0.177	Artery_Aorta
ENSG00000168890.13	TMEM150A	-5.467	-0.215	4.581E-08	0.005				1	1	1	4.581E-08	0.111	Heart_Left_Ventricle
ENSG00000168883.19	USP39	-5.467	-0.192	4.581E-08	0.007				1	1	1	4.581E-08	0.124	Adipose_Subcutaneou
ENSG00000109501.13	WFS1	5.460	0.090	4.749E-08	0.027				2	2	2	3.931E-08	0.237	Adipose_Visceral_Om
ENSG00000153113.23	CAST	-5.460	-4.207	4.751E-08	0.000				1	1	1	4.751E-08	0.005	Heart_Left_Ventricle
ENSG00000185614.4	FAM212A	5.458	0.330	4.822E-08	0.002				1	1	1	4.822E-08	0.067	Heart_Atrial_Appendag
ENSG00000185614.4	FAM212A	-5.458	-0.213	4.822E-08	0.005				1	1	1	4.822E-08	0.104	Artery_Tibial
ENSG00000185614.4	FAM212A	-5.458	-0.271	4.822E-08	0.003				1	1	1	4.822E-08	0.082	Adipose_Visceral_Om
ENSG00000272354.1	RP11-307L14.2	-5.454	-6.521	4.917E-08	0.000				2	2	2	9.817E-08	0.005	Artery_Coronary
ENSG00000179673.4	RPRML	-5.454	-0.091	4.934E-08	0.027				2	2	2	5.535E-08	0.235	Artery_Aorta
ENSG00000157259.6	GATAD1	5.453	0.112	4.955E-08	0.024				3	3	3	3.201E-06	0.450	Heart_Atrial_Appendag
ENSG00000108433.16	GOSR2	5.453	0.151	4.963E-08	0.010				3	3	3	1.675E-07	0.147	Heart_Left_Ventricle
ENSG00000110536.13	PTPMT1	5.452	0.402	4.988E-08	0.002				2	2	2	1.536E-07	0.123	Whole_Blood
ENSG00000141458.12	NPC1	-5.449	-0.047	5.071E-08	0.106				2	2	2	2.819E-09	0.415	Adipose_Visceral_Om
ENSG00000182240.15	BACE2	-5.437	-0.113	5.412E-08	0.021				3	3	3	2.892E-11	0.170	Adipose_Subcutaneou
ENSG00000177082.12	WDR73	-5.434	-0.049	5.507E-08	0.093				2	2	2	4.218E-08	0.484	Artery_Tibial
ENSG00000146147.14	MLIP	-5.432	-0.027	5.561E-08	0.334				2	2	2	5.563E-08	0.849	Liver
ENSG00000254093.8	PINX1	-5.431	-0.434	5.588E-08	0.001				2	2	2	8.722E-07	0.071	Adipose_Subcutaneou
ENSG00000148120.16	C9orf3	-5.428	-0.204	5.703E-08	0.005				1	1	1	5.703E-08	0.106	Heart_Atrial_Appendag
ENSG00000177082.12	WDR73	-5.426	-0.066	5.761E-08	0.050				2	2	2	5.739E-08	0.357	Adipose_Visceral_Om
ENSG00000168899.4	VAMP5	-5.425	-0.164	5.807E-08	0.009				2	2	2	2.314E-08	0.119	Artery_Tibial
ENSG00000154305.16	MIA3	-5.424	-0.138	5.828E-08	0.012				2	2	2	1.460E-08	0.134	Adipose_Subcutaneou
ENSG00000108379.9	WNT3	5.422	0.030	5.881E-08	0.260				3	3	3	7.497E-09	0.538	Adipose_Visceral_Om
ENSG00000169016.16	E2F6	-5.418	-0.469	6.016E-08	0.001				1	1	1	6.016E-08	0.048	Artery_Coronary
ENSG00000169016.16	E2F6	-5.418	-0.319	6.016E-08	0.002				1	1	1	6.016E-08	0.071	Artery_Aorta
ENSG00000204463.12	BAG6	5.417	0.247	6.072E-08	0.004				2	2	2	5.555E-08	0.090	Whole_Blood
ENSG00000204356.13	NELFE	5.415	0.148	6.139E-08	0.010				2	2	2	6.531E-09	0.255	Artery_Coronary
ENSG00000138792.9	ENPEP	-5.414	-0.086	6.148E-08	0.033				4	4	4	8.841E-05	0.908	Adipose_Visceral_Om
ENSG00000067208.14	EVI5	5.412	0.085	6.244E-08	0.032				2	2	2	4.268E-08	0.193	Adipose_Visceral_Om
ENSG00000204438.10	GPANK1	5.411	0.278	6.273E-08	0.002				2	2	2	6.605E-08	0.101	Kidney_Cortex
ENSG00000168899.4	VAMP5	-5.410	-0.162	6.296E-08	0.010				2	2	2	4.581E-08	0.127	Adipose_Subcutaneou
ENSG00000204387.12	C6orf48	5.410	0.149	6.308E-08	0.007				3	3	3	4.947E-11	0.176	Whole_Blood
ENSG00000204356.13	NELFE	5.408	0.129	6.365E-08	0.014				2	2	2	6.531E-09	0.294	Artery_Tibial
ENSG00000272630.1	RP11-344N10.5	-5.408	-0.065	6.385E-08	0.070				2	2	2	7.916E-10	0.787	Adipose_Subcutaneou
ENSG00000197757.7	HOXC6	5.401	0.197	6.609E-08	0.006				1	1	1	6.609E-08	0.106	Artery_Aorta
ENSG00000204308.7	RNF5	-5.396	-0.234	6.830E-08	0.006				2	2	2	5.328E-08	0.139	Adipose_Subcutaneou

gene	gene_name	zscore	effect_size	pvalue	var_g	pred_	pred_	pred_	n_snps_used	n_snps_in_cov	n_snps_in_model	best_gwas_p	largest_weight	Tissue
ENSG00000105971.14	CAV2	-5.391	-0.381	7.015E-08	0.002				2	2	2	7.486E-08	0.057	Heart_Atrial_Appendag
ENSG00000101782.14	RIOK3	-5.388	-0.374	7.142E-08	0.001				2	2	2	2.894E-07	0.056	Artery_Tibial
ENSG00000119326.14	CTNNAL1	5.386	0.033	7.188E-08	0.233				3	4	4	1.894E-07	0.731	Artery_Tibial
ENSG00000151617.15	EDNRA	5.385	0.229	7.257E-08	0.004				2	2	2	5.735E-07	0.096	Artery_Aorta
ENSG00000146215.13	CRIP3	-5.385	-6.163	7.262E-08	0.000				1	1	1	7.262E-08	0.004	Artery_Coronary
ENSG00000146215.13	CRIP3	-5.385	-0.363	7.262E-08	0.002				1	1	1	7.262E-08	0.062	Adipose_Visceral_Om
ENSG00000166822.12	TMEM170A	-5.381	-0.207	7.400E-08	0.004				1	1	1	7.400E-08	0.099	Heart_Atrial_Appendag
ENSG00000161395.13	PGAP3	5.379	0.090	7.492E-08	0.029				2	2	2	2.923E-09	0.469	Artery_Coronary
ENSG00000177082.12	WDR73	-5.371	-0.098	7.823E-08	0.026				2	2	2	5.601E-08	0.252	Heart_Left_Ventricle
ENSG00000164068.15	RNF123	5.367	0.099	8.018E-08	0.026				1	1	1	8.018E-08	0.221	Artery_Coronary
ENSG00000164068.15	RNF123	5.367	0.150	8.018E-08	0.011				1	1	1	8.018E-08	0.147	Heart_Atrial_Appendag
ENSG00000164068.15	RNF123	5.367	0.096	8.018E-08	0.026				1	2	2	8.018E-08	0.229	Adipose_Visceral_Om
ENSG00000177082.12	WDR73	-5.362	-0.050	8.249E-08	0.094				2	2	2	5.739E-08	0.469	Artery_Aorta
ENSG00000176095.11	IP6K1	5.354	0.339	8.623E-08	0.002				1	1	1	8.623E-08	0.064	Artery_Tibial
ENSG00000067208.14	EVI5	5.351	0.090	8.739E-08	0.028				2	2	2	4.268E-08	0.212	Artery_Tibial
ENSG00000149295.13	DRD2	5.351	0.084	8.753E-08	0.032				1	1	1	8.753E-08	0.249	Adipose_Subcutaneou
ENSG00000073605.18	GSDMB	5.348	0.046	8.869E-08	0.098				3	3	3	1.041E-08	0.339	Whole_Blood
ENSG00000185909.14	KLHDC8B	5.344	0.421	9.079E-08	0.001				2	2	2	1.411E-07	0.056	Adipose_Visceral_Om
ENSG00000110536.13	PTPMT1	5.343	0.293	9.129E-08	0.002				2	2	2	1.536E-07	0.167	Liver
ENSG00000139722.6	VPS37B	5.341	0.105	9.235E-08	0.016				2	2	2	8.537E-09	0.280	Adipose_Subcutaneou
ENSG00000104946.12	TBC1D17	-5.339	-0.275	9.363E-08	0.002				2	2	2	2.163E-09	0.078	Whole_Blood
ENSG00000272630.1	RP11-344N10.5	-5.335	-0.101	9.576E-08	0.031				2	2	2	1.938E-09	0.543	Adipose_Visceral_Om
ENSG00000181885.18	CLDN7	-5.333	-0.084	9.668E-08	0.035				3	4	4	4.232E-05	0.401	Whole_Blood
ENSG00000039650.11	PNKP	-5.331	-0.056	9.777E-08	0.085				4	4	4	6.243E-10	0.612	Whole_Blood
ENSG00000237541.3	HLA-DQA2	5.330	0.050	9.805E-08	0.191				1	3	3	9.805E-08	0.672	Liver
ENSG00000128482.15	RNF112	5.322	0.125	1.024E-07	0.016				2	2	2	2.249E-07	0.212	Adipose_Visceral_Om
ENSG00000232629.8	HLA-DQB2	5.321	0.016	1.033E-07	2.472				3	3	3	2.931E-06	1.111	Adipose_Subcutaneou
ENSG00000177082.12	WDR73	-5.316	-0.145	1.058E-07	0.010				2	2	2	5.742E-08	0.159	Whole_Blood
ENSG00000204435.13	CSNK2B	-5.316	-0.372	1.060E-07	0.001				1	1	1	1.060E-07	0.074	Liver
ENSG00000263001.5	GTF2I	5.310	0.142	1.095E-07	0.026				2	2	2	1.311E-08	0.265	Heart_Atrial_Appendag
ENSG00000204308.7	RNF5	-5.308	-0.150	1.110E-07	0.012				2	2	2	5.328E-08	0.207	Adipose_Visceral_Om
ENSG00000168899.4	VAMP5	-5.305	-0.105	1.127E-07	0.021				3	3	3	2.304E-07	0.152	Artery_Aorta
ENSG00000196141.13	SPATS2L	5.305	0.381	1.128E-07	0.001				2	2	2	4.732E-09	0.132	Kidney_Cortex
ENSG00000182240.15	BACE2	5.304	0.115	1.135E-07	0.016				1	1	1	1.135E-07	0.201	Kidney_Cortex
ENSG00000108433.16	GOSR2	5.301	0.043	1.154E-07	0.117				2	2	2	1.675E-07	0.276	Liver
ENSG00000165861.13	ZFYVE1	5.298	0.249	1.168E-07	0.004				2	2	2	4.716E-08	0.113	Whole_Blood
ENSG00000108641.14	B9D1	5.292	0.122	1.212E-07	0.017				1	1	1	1.212E-07	0.219	Artery_Coronary

gene	gene_name	zscore	effect_size	pvalue	var_g	pred_	pred_	pred_	n_snps_used	n_snps_in_cov	n_snps_in_model	best_gwas_p	largest_weight	Tissue
ENSG00000108641.14	B9D1	-5.292	-1.773	1.212E-07	0.000				1	1	1	1.212E-07	0.015	Liver
ENSG00000108641.14	B9D1	5.292	0.233	1.212E-07	0.004				1	1	1	1.212E-07	0.115	Adipose_Visceral_Om
ENSG00000108641.14	B9D1	5.292	2.210	1.212E-07	0.000				1	1	1	1.212E-07	0.012	Heart_Atrial_Appendag
ENSG00000108641.14	B9D1	5.292	0.185	1.212E-07	0.007				1	1	1	1.212E-07	0.144	Adipose_Subcutaneou
ENSG00000128591.15	FLNC	5.290	0.326	1.220E-07	0.002				1	1	1	1.220E-07	0.114	Liver
ENSG00000128591.15	FLNC	-5.290	-3.517	1.220E-07	0.000				1	1	1	1.220E-07	0.011	Artery_Aorta
ENSG00000105662.15	CRTC1	5.286	0.431	1.252E-07	0.001				1	1	1	1.252E-07	0.053	Adipose_Visceral_Om
ENSG00000204463.12	BAG6	5.280	0.351	1.290E-07	0.001				1	1	1	1.290E-07	0.079	Kidney_Cortex
ENSG00000172992.11	DCAKD	-5.276	-0.020	1.317E-07	0.563				4	4	4	3.603E-07	0.370	Artery_Aorta
ENSG00000263001.5	GTF2I	5.274	0.061	1.338E-07	0.142				2	2	2	1.311E-08	0.359	Adipose_Visceral_Om
ENSG00000134222.16	PSRC1	-5.268	-0.071	1.377E-07	0.034				3	3	3	3.411E-14	0.246	Heart_Left_Ventricle
ENSG00000128482.15	RNF112	5.268	0.075	1.378E-07	0.042				2	2	2	2.249E-07	0.224	Artery_Aorta
ENSG00000185909.14	KLHDC8B	5.267	0.543	1.383E-07	0.001				2	2	2	1.411E-07	0.046	Liver
ENSG00000143106.12	PSMA5	-5.265	-0.432	1.406E-07	0.002				2	2	2	1.127E-08	0.057	Liver
ENSG00000166484.19	MAPK7	5.258	0.529	1.458E-07	0.001				2	2	2	1.212E-07	0.050	Artery_Aorta
ENSG00000141744.3	PNMT	5.255	0.260	1.482E-07	0.003				2	2	2	6.459E-08	0.083	Adipose_Subcutaneou
ENSG00000164068.15	RNF123	5.254	0.092	1.488E-07	0.027				1	2	2	1.488E-07	0.234	Artery_Tibial
ENSG00000164068.15	RNF123	5.254	0.074	1.488E-07	0.043				1	2	2	1.488E-07	0.293	Adipose_Subcutaneou
ENSG00000164068.15	RNF123	5.254	0.083	1.488E-07	0.035				1	2	2	1.488E-07	0.262	Artery_Aorta
ENSG00000164068.15	RNF123	5.254	0.351	1.488E-07	0.002				1	1	1	1.488E-07	0.062	Whole_Blood
ENSG00000115561.15	CHMP3	-5.254	-0.115	1.489E-07	0.018				3	3	3	4.466E-07	0.146	Heart_Atrial_Appendag
ENSG00000065526.10	SPEN	-5.253	-0.544	1.496E-07	0.001				1	1	1	1.496E-07	0.039	Artery_Aorta
ENSG00000196666.4	FAM180B	-5.253	-0.262	1.496E-07	0.004				1	1	1	1.496E-07	0.184	Adipose_Subcutaneou
ENSG00000065526.10	SPEN	-5.253	-0.662	1.496E-07	0.000				1	1	1	1.496E-07	0.032	Adipose_Visceral_Om
ENSG00000196666.4	FAM180B	-5.253	-0.358	1.496E-07	0.002				1	1	1	1.496E-07	0.134	Artery_Tibial
ENSG00000196666.4	FAM180B	-5.253	-0.336	1.496E-07	0.002				1	1	1	1.496E-07	0.143	Artery_Aorta
ENSG00000134490.13	TMEM241	5.252	0.081	1.502E-07	0.030				3	3	3	1.575E-07	0.209	Whole_Blood
ENSG00000110536.13	PTPMT1	5.248	0.263	1.536E-07	0.004				1	1	1	1.536E-07	0.184	Artery_Tibial
ENSG00000149187.17	CELF1	5.248	0.895	1.536E-07	0.000				1	1	1	1.536E-07	0.054	Heart_Left_Ventricle
ENSG00000149187.17	CELF1	5.248	0.735	1.536E-07	0.000				1	1	1	1.536E-07	0.066	Heart_Atrial_Appendag
ENSG00000110536.13	PTPMT1	5.248	0.202	1.536E-07	0.006				1	1	1	1.536E-07	0.240	Kidney_Cortex
ENSG00000110536.13	PTPMT1	5.248	0.273	1.536E-07	0.004				1	1	1	1.536E-07	0.177	Artery_Coronary
ENSG00000149187.17	CELF1	5.248	1.009	1.536E-07	0.000				1	1	1	1.536E-07	0.048	Artery_Coronary
ENSG00000196821.9	C6orf106	5.247	0.130	1.544E-07	0.012				1	1	1	1.544E-07	0.235	Heart_Atrial_Appendag
ENSG00000041357.15	PSMA4	5.247	0.185	1.549E-07	0.006				3	3	3	1.280E-07	0.161	Whole_Blood
ENSG00000065526.10	SPEN	-5.246	-0.800	1.558E-07	0.001				1	1	1	1.558E-07	0.037	Artery_Tibial
ENSG00000171634.16	BPTF	-5.240	-0.418	1.605E-07	0.002				1	1	1	1.605E-07	0.064	Adipose_Subcutaneou

gene	gene_name	zscore	effect_size	pvalue	var_g	pred_	pred	pred_	n_snps_used	n_snps_in_cov	n_snps_in_model	best_gwas_p	largest_weight	Tissue
ENSG00000171634.16	BPTF	-5.240	-0.355	1.605E-07	0.002				1	1	1	1.605E-07	0.075	Kidney_Cortex
ENSG00000171634.16	BPTF	-5.240	-0.367	1.605E-07	0.002				1	1	1	1.605E-07	0.073	Artery_Tibial
ENSG00000171634.16	BPTF	-5.240	-0.392	1.605E-07	0.002				1	1	1	1.605E-07	0.068	Liver
ENSG00000164068.15	RNF123	5.240	0.185	1.608E-07	0.006				2	2	2	1.488E-07	0.115	Heart_Left_Ventricle
ENSG00000110911.14	SLC11A2	5.235	0.599	1.654E-07	0.001				2	2	2	5.248E-08	0.035	Artery_Tibial
ENSG00000115486.11	GGCX	5.232	0.055	1.681E-07	0.062				2	2	2	4.274E-09	0.346	Kidney_Cortex
ENSG00000119979.16	FAM45A	-5.230	-0.406	1.697E-07	0.001				1	1	1	1.697E-07	0.050	Liver
ENSG00000151135.9	TMEM263	5.229	0.058	1.701E-07	0.073				3	3	3	3.090E-09	0.151	Adipose_Subcutaneou
ENSG00000169016.16	E2F6	5.225	0.093	1.737E-07	0.027				2	2	2	1.490E-08	0.217	Heart_Left_Ventricle
ENSG00000115561.15	CHMP3	-5.222	-0.141	1.766E-07	0.010				2	2	2	4.466E-07	0.116	Heart_Left_Ventricle
ENSG00000198265.11	HELZ	5.219	0.213	1.800E-07	0.004				2	2	2	3.144E-05	0.075	Whole_Blood
ENSG00000110536.13	PTPMT1	5.217	0.198	1.818E-07	0.006				2	2	2	1.536E-07	0.243	Heart_Atrial_Appendag
ENSG00000157259.6	GATAD1	5.216	0.076	1.824E-07	0.043				3	3	3	3.201E-06	0.422	Adipose_Subcutaneou
ENSG00000119326.14	CTNNAL1	5.209	0.068	1.894E-07	0.055				1	1	1	1.894E-07	0.452	Artery_Coronary
ENSG00000119326.14	CTNNAL1	5.209	0.077	1.894E-07	0.033				1	1	1	1.894E-07	0.400	Kidney_Cortex
ENSG00000186063.12	AIDA	-5.208	-0.628	1.911E-07	0.001				1	1	1	1.911E-07	0.045	Whole_Blood
ENSG00000050820.16	BCAR1	5.193	0.089	2.067E-07	0.021				1	1	1	2.067E-07	0.216	Artery_Aorta
ENSG00000070061.14	IKBKAP	-5.192	-0.135	2.075E-07	0.018				2	2	2	1.280E-06	0.299	Adipose_Subcutaneou
ENSG00000110536.13	PTPMT1	5.192	0.259	2.079E-07	0.004				2	2	2	1.536E-07	0.185	Heart_Left_Ventricle
ENSG00000160710.15	ADAR	5.188	0.162	2.129E-07	0.008				1	1	1	2.129E-07	0.126	Artery_Coronary
ENSG00000232388.4	LINC00493	5.187	0.175	2.135E-07	0.005				2	2	2	3.008E-05	0.194	Kidney_Cortex
ENSG00000274021.1	RP11-823E8.3	-5.184	-1.988	2.169E-07	0.000				2	2	2	1.886E-07	0.011	Liver
ENSG00000160710.15	ADAR	5.182	0.078	2.198E-07	0.036				2	2	2	2.129E-07	0.263	Liver
ENSG00000116793.15	PHTF1	-5.181	-0.124	2.211E-07	0.011				1	1	1	2.211E-07	0.185	Artery_Tibial
ENSG00000116793.15	PHTF1	-5.181	-0.157	2.211E-07	0.008				1	1	1	2.211E-07	0.146	Artery_Coronary
ENSG00000116793.15	PHTF1	-5.181	-0.135	2.211E-07	0.009				1	1	1	2.211E-07	0.170	Adipose_Visceral_Om
ENSG00000116793.15	PHTF1	-5.181	-0.176	2.211E-07	0.006				1	1	1	2.211E-07	0.130	Artery_Aorta
ENSG00000261701.6	HPR	-5.177	-0.109	2.259E-07	0.021				2	2	2	2.288E-07	0.222	Liver
ENSG00000257017.8	HP	-5.174	-0.152	2.288E-07	0.009				1	1	1	2.288E-07	0.163	Kidney_Cortex
ENSG00000173641.17	HSPB7	5.174	0.256	2.290E-07	0.003				2	2	2	7.161E-12	0.070	Heart_Left_Ventricle
ENSG00000168890.13	TMEM150A	-5.173	-0.221	2.304E-07	0.005				1	1	1	2.304E-07	0.103	Liver
ENSG00000168890.13	TMEM150A	-5.173	-0.302	2.304E-07	0.003				1	1	1	2.304E-07	0.075	Artery_Aorta
ENSG00000168883.19	USP39	-5.173	-0.181	2.304E-07	0.006				1	1	1	2.304E-07	0.126	Artery_Coronary
ENSG00000168883.19	USP39	-5.173	-0.157	2.304E-07	0.009				1	1	1	2.304E-07	0.144	Heart_Left_Ventricle
ENSG00000186532.11	SMYD4	-5.173	-0.232	2.308E-07	0.004				1	1	1	2.308E-07	0.089	Whole_Blood
ENSG00000243649.8	CFB	5.172	0.222	2.313E-07	0.004				1	1	1	2.313E-07	0.147	Heart_Atrial_Appendag
ENSG00000182218.9	HHIPL1	-5.172	-0.127	2.322E-07	0.012				2	2	2	4.768E-05	0.213	Artery_Coronary

gene	gene_name	zscore	effect_size	pvalue	var_g	pred_	pred	pred_	n_snps_used	n_snps_in_cov	n_snps_in_model	best_gwas_p	largest_weight	Tissue
ENSG00000167110.17	GOLGA2	-5.169	-0.712	2.351E-07	0.000				1	1	1	2.351E-07	0.039	Artery_Tibial
ENSG00000186063.12	AIDA	5.166	0.505	2.389E-07	0.001				2	2	2	1.911E-07	0.048	Kidney_Cortex
ENSG00000106460.18	TMEM106B	5.166	0.095	2.389E-07	0.034				2	2	2	2.079E-08	0.173	Kidney_Cortex
ENSG00000204310.12	AGPAT1	5.166	0.177	2.395E-07	0.007				1	1	1	2.395E-07	0.114	Heart_Left_Ventricle
ENSG00000106460.18	TMEM106B	5.161	0.093	2.456E-07	0.024				1	1	1	2.456E-07	0.238	Whole_Blood
ENSG00000050426.15	LETMD1	-5.161	-0.315	2.458E-07	0.002				1	1	1	2.458E-07	0.065	Kidney_Cortex
ENSG00000050426.15	LETMD1	-5.161	-0.157	2.458E-07	0.007				1	1	1	2.458E-07	0.130	Liver
ENSG00000050426.15	LETMD1	-5.161	-0.283	2.458E-07	0.002				1	1	1	2.458E-07	0.072	Artery_Tibial
ENSG00000163491.16	NEK10	-5.160	-0.110	2.467E-07	0.018				1	1	1	2.467E-07	0.193	Artery_Aorta
ENSG00000124562.9	SNRPC	-5.159	-0.128	2.481E-07	0.016				2	2	2	3.830E-11	0.126	Heart_Left_Ventricle
ENSG00000185614.4	FAM212A	-5.149	-0.146	2.623E-07	0.011				2	2	2	4.822E-08	0.139	Artery_Coronary
ENSG00000196735.11	HLA-DQA1	-5.148	-0.049	2.634E-07	0.155				2	2	2	1.632E-06	0.463	Adipose_Subcutaneou
ENSG00000227078.1	LINC02094	5.148	0.050	2.639E-07	0.108				2	2	2	4.448E-06	0.340	Artery_Aorta
ENSG00000267302.5	RP11-178C3.2	-5.141	-0.483	2.735E-07	0.001				2	2	2	1.485E-06	0.083	Kidney_Cortex
ENSG00000159199.13	ATP5G1	5.140	0.353	2.744E-07	0.002				2	2	2	4.455E-07	0.069	Adipose_Visceral_Om
ENSG00000172260.14	NEGR1	-5.137	-0.605	2.788E-07	0.001				2	2	2	4.367E-07	0.026	Heart_Left_Ventricle
ENSG00000127804.12	METTL16	-5.132	-0.145	2.873E-07	0.009				1	1	1	2.873E-07	0.172	Liver
ENSG00000183527.11	PSMG1	-5.127	-0.024	2.944E-07	0.379				4	4	4	1.487E-07	0.459	Artery_Tibial
ENSG00000128596.16	CCDC136	-5.126	-0.155	2.955E-07	0.009				2	2	2	3.205E-07	0.237	Artery_Aorta
ENSG00000198373.12	WWP2	5.124	0.104	2.987E-07	0.022				3	3	3	1.794E-08	0.437	Artery_Aorta
ENSG00000112245.10	PTP4A1	-5.123	-0.784	3.010E-07	0.000				1	1	1	3.010E-07	0.024	Artery_Aorta
ENSG00000227078.1	LINC02094	5.116	0.052	3.113E-07	0.102				2	2	2	4.448E-06	0.315	Artery_Tibial
ENSG00000204305.13	AGER	5.116	0.054	3.125E-07	0.154				5	5	5	3.862E-08	0.964	Adipose_Subcutaneou
ENSG00000118655.4	DCLRE1B	-5.108	-0.146	3.254E-07	0.009				2	2	2	5.759E-09	0.132	Artery_Aorta
ENSG00000173421.16	CCDC36	-5.107	-0.033	3.279E-07	0.193				2	2	2	3.785E-07	0.651	Artery_Coronary
ENSG00000109917.10	ZPR1	-5.099	-0.344	3.422E-07	0.002				1	1	1	3.422E-07	0.119	Whole_Blood
ENSG00000204396.10	VWA7	5.098	0.116	3.434E-07	0.020				4	4	4	2.442E-06	0.380	Heart_Left_Ventricle
ENSG00000159199.13	ATP5G1	5.096	0.075	3.462E-07	0.040				2	2	2	3.139E-07	0.301	Heart_Left_Ventricle
ENSG00000130595.17	TNNT3	-5.095	-0.095	3.479E-07	0.022				2	2	2	2.631E-07	0.214	Whole_Blood
ENSG00000136448.11	NMT1	5.095	0.041	3.494E-07	0.130				3	3	3	3.582E-06	0.250	Artery_Aorta
ENSG00000244731.7	C4A	-5.094	-0.049	3.499E-07	0.125				2	2	2	5.131E-06	0.797	Liver
ENSG00000141736.13	ERBB2	5.090	0.335	3.585E-07	0.002				2	2	2	8.314E-10	0.133	Liver
ENSG00000146147.14	MLIP	-5.087	-0.116	3.643E-07	0.017				2	2	2	3.663E-07	0.184	Artery_Aorta
ENSG00000119326.14	CTNNAL1	5.085	0.053	3.684E-07	0.076				2	2	2	1.894E-07	0.528	Adipose_Visceral_Om
ENSG00000185909.14	KLHDC8B	5.084	0.118	3.688E-07	0.013				2	2	2	1.411E-07	0.172	Kidney_Cortex
ENSG00000131771.13	PPP1R1B	5.083	0.123	3.717E-07	0.015				2	2	2	3.719E-07	0.186	Heart_Atrial_Appendag
ENSG00000109917.10	ZPR1	-5.083	-0.344	3.718E-07	0.002				2	2	2	3.422E-07	0.120	Adipose_Visceral_Om

gene	gene_name	zscore	effect_size	pvalue	var_g	pred_	pred	pred_	n_snps_used	n_snps_in_cov	n_snps_in_model	best_gwas_p	largest_weight	Tissue
ENSG00000214546.3	AC087491.2	-5.083	-199.244	3.719E-07	0.000				1	1	1	3.719E-07	0.000	Adipose_Subcutaneou
ENSG00000172992.11	DCAKD	-5.080	-0.036	3.771E-07	0.164				3	3	3	6.250E-06	0.414	Adipose_Subcutaneou
ENSG00000173421.16	CCDC36	-5.079	-0.043	3.785E-07	0.111				1	2	2	3.785E-07	0.490	Heart_Left_Ventricle
ENSG00000173421.16	CCDC36	-5.079	-0.048	3.785E-07	0.089				1	2	2	3.785E-07	0.436	Adipose_Subcutaneou
ENSG00000173421.16	CCDC36	-5.079	-0.037	3.785E-07	0.155				1	2	2	3.785E-07	0.560	Heart_Atrial_Appendag
ENSG00000105329.9	TGFB1	5.079	0.185	3.801E-07	0.006				3	3	3	6.300E-07	0.142	Artery_Coronary
