# Supplementary material for: Connecting intermediate phenotypes to disease using multi-omics in heart failure
Source: Pac Symp Biocomput. Author manuscript; Available in PMC 2025 Feb 13. (PMC11822568; doi:10.1142/9789819807024_0036)
Supplement: Supplemental Table 4 (A-F) [file NIHMS2038838-supplement-Supplemental_Table_4__A-F_.pdf]

Supplementary Table 4A: Left venticular ejectrion fraction (LVEF)

MCODE App Results

Date: Jul 15, 2024, 3:10:14 PM

Parameters:
  Network Scoring:
    Include Loops: true Degree Cutoff: 3
  Cluster Finding:
    Node Score Cutoff: 0.2 Haircut: true Fluff: false K-Core: 2 Max. Depth from Seed: 100

Cluster	Score (Density	Nodes	Edges	Node IDs
1	1.5	3	3	HSPB7, FLNC, ALPK3

Metascape Enrichment

**Cluster**	**GroupID**	**Category**	**Term**	**Description**	**LogP**	**Log(q-value)**	**Genes**	**Symbols**	**InTerm_InList**
1	No enriched terms								

Supplementary Table 4B: Left venticular mass (LVM)

MCODE App Results

No clusters Identified

Metascape Enrichment

Cluster	GroupID	Category	Term	Description	LogP	Log(q-value)	Genes	Symbols	InTerm_InList
1	No enriched terms								

Supplementary Table 4C: Left venticular-end systolic volume (LVESV)

MCODE App Results

Date: Jul 29, 2024, 2:48:33 AM

Parameters:

Network Scoring:

Include Loops: false Degree Cutoff: 3

Cluster Finding:

Node Score Cutoff: 0.2 Haircut: true Fluff: false K-Core: 2 Max. Depth from Seed: 100

Cluster	Score (Density	Nodes	Edges	Node IDs
1	3	3	3	ALPK3, FLNC, HSPB7

Cluster	GroupID	Category	Term	Description	LogP	Log(q-value)	Genes	Symbols	InTerm_InList
1	No enriched terms								

Supplementary Table 4D: Left venticular-end diastolic volume (LVEDV)

MCODE App Results

No clusters Identified

Metascape Enrichment

Cluster	GroupID	Category	Term	Description	LogP	Log(q-value)	Genes	Symbols	InTerm_InList
1	No enriched terms								

Supplementary Table 4E: All-cause heart failure in a multi-ancestry population

MCODE App Results

Date: Jul 15, 2024, 9:58:17 PM

Parameters:

Network Scoring:

Include Loops: false Degree Cutoff: 3

Cluster Finding:

Node Score Cutoff: 0.2 Haircut: true Fluff: false K-Core: 2 Max. Depth from Seed: 100

Cluster	Score (Density*#N	Nodes	Edges	Node IDs
1	4.889	10	22	NCF1, CAV1, ACTN1, PRKCA, CD36, FLNC, ACTN2, SYNPO2L, NOS3, MYOZ1
2	4.286	8	15	MIA3, ABCG8, HHIPL1, ABCG5, PSRC1, ZC3HC1, PHACTR1, PCSK9
3	3.75	17	30	SRSF3, STARD3, SNRPC, LGR5, CDKN1A, GSDMB, AIF1, HNRNPA0, UPF1, CDKN2B, FGF5, PLAU, DDX39B, TGFB1, MIEN1, PPP1R1B, PGAP3
4	3	3	3	DNAJC9, PSMG1, MRPS16
5	3	3	3	IP6K1, APEH, RNF123

Metascape Enrichment

Cluster	GroupID	Category	Term	Description	LogP	Log(q-value)	Genes	Symbols	InTerm_InList
1	1_Summary	GO Biological Processes	GO:0045214	sarcomere organization	-9.092	-4.871	88,2318,58529,79933,87,857,948	ACTN2,FLNC,MYOZ1,SYNPO2L,ACTN1,CAV1,CD36	7/-
1	1_Member	GO Biological Processes	GO:0045214	sarcomere organization	-9.092	-4.871	88,2318,58529,79933	ACTN2,FLNC,MYOZ1,SYNPO2L	4/44
1	1_Member	GO Biological Processes	GO:0055001	muscle cell development	-8.825	-4.871	87,88,2318,58529,79933	ACTN1,ACTN2,FLNC,MYOZ1,SYNPO2L	5/175
1	1_Member	GO Biological Processes	GO:0061061	muscle structure development	-8.307	-4.659	87,88,857,2318,58529,79933	ACTN1,ACTN2,CAV1,FLNC,MYOZ1,SYNPO2L	6/520
1	1_Member	GO Biological Processes	GO:0030239	myofibril assembly	-8.265	-4.659	88,2318,58529,79933	ACTN2,FLNC,MYOZ1,SYNPO2L	4/70
1	1_Member	GO Biological Processes	GO:0055002	striated muscle cell development	-8.215	-4.659	88,2318,58529,79933	ACTN2,FLNC,MYOZ1,SYNPO2L	4/72
1	1_Member	GO Biological Processes	GO:0097435	supramolecular fiber organization	-7.971	-4.494	87,88,948,2318,58529,79933	ACTN1,ACTN2,CD36,FLNC,MYOZ1,SYNPO2L	6/592
1	1_Member	GO Biological Processes	GO:0042692	muscle cell differentiation	-7.732	-4.380	87,88,2318,58529,79933	ACTN1,ACTN2,FLNC,MYOZ1,SYNPO2L	5/289
1	1_Member	GO Biological Processes	GO:0010927	cellular component assembly involved in morphogenesis	-7.273	-4.083	88,2318,58529,79933	ACTN2,FLNC,MYOZ1,SYNPO2L	4/123
1	1_Member	GO Biological Processes	GO:0031032	actomyosin structure organization	-7.258	-4.083	88,2318,58529,79933	ACTN2,FLNC,MYOZ1,SYNPO2L	4/124
1	1_Member	GO Biological Processes	GO:0030036	actin cytoskeleton organization	-6.367	-3.367	87,88,2318,58529,79933	ACTN1,ACTN2,FLNC,MYOZ1,SYNPO2L	5/544
1	1_Member	GO Biological Processes	GO:0051146	striated muscle cell differentiation	-6.205	-3.252	88,2318,58529,79933	ACTN2,FLNC,MYOZ1,SYNPO2L	4/227
1	1_Member	GO Biological Processes	GO:0030029	actin filament-based process	-6.108	-3.175	87,88,2318,58529,79933	ACTN1,ACTN2,FLNC,MYOZ1,SYNPO2L	5/614
1	1_Member	GO Biological Processes	GO:0140694	non-membrane-bounded organelle assembly	-5.498	-2.674	88,2318,58529,79933	ACTN2,FLNC,MYOZ1,SYNPO2L	4/342
1	1_Member	Reactome Gene Sets	R-HSA-1500931	Cell-Cell communication	-4.830	-2.112	87,88,2318	ACTN1,ACTN2,FLNC	3/153

1	1_Member	GO Biological Processes	GO:0032989	cellular anatomical entity morphogenesis	-4.522	-1.846	88,2318,58529,79933	ACTN2,FLNC,MYOZ1,SYNPO2L	4/605
1	1_Member	KEGG Pathway	hsa04820	Cytoskeleton in muscle cells	-4.308	-1.686	88,2318,58529	ACTN2,FLNC,MYOZ1	3/229
1	2_Summary	Reactome Gene Sets	R-HSA-109582	Hemostasis	-7.843	-4.434	87,88,857,948,4846,5578	ACTN1,ACTN2,CAV1,CD36,NOS3,PRKCA	6/-
1	2_Member	Reactome Gene Sets	R-HSA-109582	Hemostasis	-7.843	-4.434	87,88,857,948,4846,5578	ACTN1,ACTN2,CAV1,CD36,NOS3,PRKCA	6/622
1	2_Member	Reactome Gene Sets	R-HSA-76005	Response to elevated platelet cytosolic Ca2+	-7.123	-3.982	87,88,948,5578	ACTN1,ACTN2,CD36,PRKCA	4/134
1	2_Member	Reactome Gene Sets	R-HSA-76002	Platelet activation, signaling and aggregation	-5.951	-3.038	87,88,948,5578	ACTN1,ACTN2,CD36,PRKCA	4/263
1	2_Member	Reactome Gene Sets	R-HSA-114608	Platelet degranulation	-5.052	-2.275	87,88,948	ACTN1,ACTN2,CD36	3/129
1	2_Member	GO Biological Processes	GO:0034329	cell junction assembly	-4.035	-1.479	87,88,5578	ACTN1,ACTN2,PRKCA	3/283
1	2_Member	GO Biological Processes	GO:0034330	cell junction organization	-3.227	-0.835	87,88,5578	ACTN1,ACTN2,PRKCA	3/533
1	3_Summary	Reactome Gene Sets	R-HSA-4420097	VEGFA-VEGFR2 Pathway	-7.653	-4.353	857,4846,5578,653361,948,58529,79933	CAV1,NOS3,PRKCA,NCF1,CD36,MYOZ1,SYNPO2L	7/-
1	3_Member	Reactome Gene Sets	R-HSA-4420097	VEGFA-VEGFR2 Pathway	-7.653	-4.353	857,4846,5578,653361	CAV1,NOS3,PRKCA,NCF1	4/99
1	3_Member	Reactome Gene Sets	R-HSA-194138	Signaling by VEGF	-7.501	-4.246	857,4846,5578,653361	CAV1,NOS3,PRKCA,NCF1	4/108
1	3_Member	GO Biological Processes	GO:0061041	regulation of wound healing	-7.071	-3.963	857,948,4846,58529	CAV1,CD36,NOS3,MYOZ1	4/138
1	3_Member	GO Biological Processes	GO:1903034	regulation of response to wounding	-6.618	-3.567	857,948,4846,58529	CAV1,CD36,NOS3,MYOZ1	4/179
1	3_Member	KEGG Pathway	hsa05415	Diabetic cardiomyopathy	-6.399	-3.375	948,4846,5578,653361	CD36,NOS3,PRKCA,NCF1	4/203
1	3_Member	KEGG Pathway	hsa05417	Lipid and atherosclerosis	-6.300	-3.323	948,4846,5578,653361	CD36,NOS3,PRKCA,NCF1	4/215
1	3_Member	GO Biological Processes	GO:0030193	regulation of blood coagulation	-5.872	-2.993	857,948,4846	CAV1,CD36,NOS3	3/69
1	3_Member	GO Biological Processes	GO:0062197	cellular response to chemical stress	-5.867	-2.993	857,948,4846,653361	CAV1,CD36,NOS3,NCF1	4/276
1	3_Member	GO Biological Processes	GO:1900046	regulation of hemostasis	-5.834	-2.977	857,948,4846	CAV1,CD36,NOS3	3/71
1	3_Member	GO Biological Processes	GO:0050818	regulation of coagulation	-5.780	-2.940	857,948,4846	CAV1,CD36,NOS3	3/74
1	3_Member	KEGG Pathway	hsa05418	Fluid shear stress and atherosclerosis	-4.955	-2.219	857,4846,653361	CAV1,NOS3,NCF1	3/139
1	3_Member	GO Biological Processes	GO:0044089	positive regulation of cellular component biogenesis	-4.823	-2.112	857,948,5578,79933	CAV1,CD36,PRKCA,SYNPO2L	4/507
1	3_Member	GO Biological Processes	GO:0002831	regulation of response to biotic stimulus	-4.766	-2.068	857,948,5578,653361	CAV1,CD36,PRKCA,NCF1	4/524
1	3_Member	Reactome Gene Sets	R-HSA-9006934	Signaling by Receptor Tyrosine Kinases	-4.734	-2.047	857,4846,5578,653361	CAV1,NOS3,PRKCA,NCF1	4/534
1	3_Member	GO Biological Processes	GO:0043408	regulation of MAPK cascade	-4.351	-1.706	857,948,5578,653361	CAV1,CD36,PRKCA,NCF1	4/669
1	3_Member	GO Biological Processes	GO:0034599	cellular response to oxidative stress	-4.348	-1.706	948,4846,653361	CD36,NOS3,NCF1	3/222
1	3_Member	GO Biological Processes	GO:0050730	regulation of peptidyl-tyrosine phosphorylation	-4.331	-1.699	857,948,653361	CAV1,CD36,NCF1	3/225
1	3_Member	GO Biological Processes	GO:0010562	positive regulation of phosphorus metabolic process	-4.269	-1.668	857,948,4846,653361	CAV1,CD36,NOS3,NCF1	4/702
1	3_Member	GO Biological Processes	GO:0045937	positive regulation of phosphate metabolic process	-4.269	-1.668	857,948,4846,653361	CAV1,CD36,NOS3,NCF1	4/702
1	3_Member	GO Biological Processes	GO:0090257	regulation of muscle system process	-4.221	-1.647	857,4846,5578	CAV1,NOS3,PRKCA	3/245
1	3_Member	GO Biological Processes	GO:0003018	vascular process in circulatory system	-4.072	-1.507	857,948,4846	CAV1,CD36,NOS3	3/275
1	3_Member	GO Biological Processes	GO:0002833	positive regulation of response to biotic stimulus	-3.930	-1.383	857,948,5578	CAV1,CD36,PRKCA	3/307
1	3_Member	GO Biological Processes	GO:0001525	angiogenesis	-3.807	-1.276	857,4846,5578	CAV1,NOS3,PRKCA	3/338
1	3_Member	GO Biological Processes	GO:0050878	regulation of body fluid levels	-3.751	-1.237	857,948,4846	CAV1,CD36,NOS3	3/353
1	3_Member	GO Biological Processes	GO:0006979	response to oxidative stress	-3.705	-1.198	948,4846,653361	CD36,NOS3,NCF1	3/366

1	3_Member	GO Biological Processes	GO:0048514	blood vessel morphogenesis	-3.496	-1.012	857,4846,5578	CAV1,NOS3,PRKCA	3/431
1	3_Member	GO Biological Processes	GO:0045088	regulation of innate immune response	-3.470	-0.993	857,948,653361	CAV1,CD36,NCF1	3/440
1	3_Member	GO Biological Processes	GO:0043410	positive regulation of MAPK cascade	-3.373	-0.937	948,5578,653361	CD36,PRKCA,NCF1	3/475
1	3_Member	GO Biological Processes	GO:0045785	positive regulation of cell adhesion	-3.346	-0.918	857,948,5578	CAV1,CD36,PRKCA	3/485
1	3_Member	GO Biological Processes	GO:0003013	circulatory system process	-3.288	-0.865	857,948,4846	CAV1,CD36,NOS3	3/508
1	3_Member	GO Biological Processes	GO:0001568	blood vessel development	-3.241	-0.841	857,4846,5578	CAV1,NOS3,PRKCA	3/527
1	3_Member	GO Biological Processes	GO:0010035	response to inorganic substance	-3.241	-0.841	857,4846,653361	CAV1,NOS3,NCF1	3/527
1	3_Member	GO Biological Processes	GO:0071396	cellular response to lipid	-3.220	-0.834	948,4846,653361	CD36,NOS3,NCF1	3/536
1	3_Member	GO Biological Processes	GO:0001944	vasculature development	-3.194	-0.814	857,4846,5578	CAV1,NOS3,PRKCA	3/547
1	3_Member	GO Biological Processes	GO:0032103	positive regulation of response to external stimulus	-3.162	-0.805	857,948,5578	CAV1,CD36,PRKCA	3/561
1	3_Member	GO Biological Processes	GO:0044057	regulation of system process	-3.162	-0.805	857,4846,5578	CAV1,NOS3,PRKCA	3/561
1	3_Member	GO Biological Processes	GO:0001934	positive regulation of protein phosphorylation	-3.124	-0.779	857,948,653361	CAV1,CD36,NCF1	3/578
1	3_Member	GO Biological Processes	GO:0030335	positive regulation of cell migration	-3.120	-0.779	857,4846,5578	CAV1,NOS3,PRKCA	3/580
1	3_Member	GO Biological Processes	GO:2000147	positive regulation of cell motility	-3.063	-0.727	857,4846,5578	CAV1,NOS3,PRKCA	3/607
1	3_Member	GO Biological Processes	GO:0040017	positive regulation of locomotion	-3.034	-0.705	857,4846,5578	CAV1,NOS3,PRKCA	3/621
1	3_Member	GO Biological Processes	GO:0042327	positive regulation of phosphorylation	-3.030	-0.705	857,948,653361	CAV1,CD36,NCF1	3/623
1	3_Member	GO Biological Processes	GO:0050865	regulation of cell activation	-3.016	-0.699	857,4846,5578	CAV1,NOS3,PRKCA	3/630
1	3_Member	GO Biological Processes	GO:0035239	tube morphogenesis	-2.916	-0.609	857,4846,5578	CAV1,NOS3,PRKCA	3/682
1	3_Member	GO Biological Processes	GO:0019725	cellular homeostasis	-2.915	-0.609	857,4846,653361	CAV1,NOS3,NCF1	3/683
1	3_Member	GO Biological Processes	GO:0009617	response to bacterium	-2.866	-0.565	857,948,4846	CAV1,CD36,NOS3	3/710
1	3_Member	GO Biological Processes	GO:0061024	membrane organization	-2.781	-0.485	857,948,5578	CAV1,CD36,PRKCA	3/760
1	3_Member	GO Biological Processes	GO:0031401	positive regulation of protein modification process	-2.775	-0.483	857,948,653361	CAV1,CD36,NCF1	3/764
1	4_Summary	GO Biological Processes	GO:0043267	negative regulation of potassium ion transport	-6.698	-3.620	88,857,4846,948,58529,79933,653361	ACTN2,CAV1,NOS3,CD36,MYOZ1,SYNPO2L,NCF1	7/-
1	4_Member	GO Biological Processes	GO:0043267	negative regulation of potassium ion transport	-6.698	-3.620	88,857,4846	ACTN2,CAV1,NOS3	3/37
1	4_Member	GO Biological Processes	GO:0043266	regulation of potassium ion transport	-5.359	-2.551	88,857,4846	ACTN2,CAV1,NOS3	3/102
1	4_Member	GO Biological Processes	GO:0043271	negative regulation of monoatomic ion transport	-5.013	-2.249	88,857,4846	ACTN2,CAV1,NOS3	3/133
1	4_Member	GO Biological Processes	GO:0051051	negative regulation of transport	-4.981	-2.232	88,857,948,4846	ACTN2,CAV1,CD36,NOS3	4/462
1	4_Member	GO Biological Processes	GO:1901653	cellular response to peptide	-3.905	-1.367	88,857,948	ACTN2,CAV1,CD36	3/313
1	4_Member	GO Biological Processes	GO:0060537	muscle tissue development	-3.773	-1.251	88,857,58529	ACTN2,CAV1,MYOZ1	3/347
1	4_Member	GO Biological Processes	GO:0032970	regulation of actin filament-based process	-3.640	-1.141	88,857,79933	ACTN2,CAV1,SYNPO2L	3/385
1	4_Member	GO Biological Processes	GO:0010959	regulation of metal ion transport	-3.611	-1.119	88,857,4846	ACTN2,CAV1,NOS3	3/394
1	4_Member	GO Biological Processes	GO:0042391	regulation of membrane potential	-3.453	-0.983	88,857,948	ACTN2,CAV1,CD36	3/446
1	4_Member	GO Biological Processes	GO:1901652	response to peptide	-3.389	-0.938	88,857,948	ACTN2,CAV1,CD36	3/469
1	4_Member	GO Biological Processes	GO:0043269	regulation of monoatomic ion transport	-3.386	-0.938	88,857,4846	ACTN2,CAV1,NOS3	3/470
1	4_Member	GO Biological Processes	GO:0072657	protein localization to membrane	-3.373	-0.937	88,857,653361	ACTN2,CAV1,NCF1	3/475

1	4_Member	GO Biological Processes	GO:0090066	regulation of anatomical structure size	-3.268	-0.852	88,857,4846	ACTN2,CAV1,NOS3	3/516
1	4_Member	GO Biological Processes	GO:0060627	regulation of vesicle-mediated transport	-3.239	-0.841	88,857,948	ACTN2,CAV1,CD36	3/528
1	4_Member	GO Biological Processes	GO:0071417	cellular response to organonitrogen compound	-3.173	-0.805	88,857,948	ACTN2,CAV1,CD36	3/556
1	4_Member	GO Biological Processes	GO:0051668	localization within membrane	-3.167	-0.805	88,857,653361	ACTN2,CAV1,NCF1	3/559
1	4_Member	GO Biological Processes	GO:1901699	cellular response to nitrogen compound	-3.014	-0.699	88,857,948	ACTN2,CAV1,CD36	3/631
1	4_Member	GO Biological Processes	GO:0009725	response to hormone	-2.741	-0.454	88,857,4846	ACTN2,CAV1,NOS3	3/785
1	5_Summary	KEGG Pathway	hsa04670	Leukocyte transendothelial migration	-5.202	-2.410	87,5578,653361,857	ACTN1,PRKCA,NCF1,CAV1	4/-
1	5_Member	KEGG Pathway	hsa04670	Leukocyte transendothelial migration	-5.202	-2.410	87,5578,653361	ACTN1,PRKCA,NCF1	3/115
1	5_Member	KEGG Pathway	hsa04510	Focal adhesion	-4.470	-1.806	87,857,5578	ACTN1,CAV1,PRKCA	3/202
1	5_Member	Reactome Gene Sets	R-HSA-194315	Signaling by Rho GTPases	-4.260	-1.668	87,857,5578,653361	ACTN1,CAV1,PRKCA,NCF1	4/706
1	5_Member	Reactome Gene Sets	R-HSA-9716542	Signaling by Rho GTPases, Miro GTPases and RHOBTB3	-4.222	-1.647	87,857,5578,653361	ACTN1,CAV1,PRKCA,NCF1	4/722
1	5_Member	Reactome Gene Sets	R-HSA-9012999	RHO GTPase cycle	-3.444	-0.982	87,857,653361	ACTN1,CAV1,NCF1	3/449
1	6_Summary	GO Biological Processes	GO:0007507	heart development	-3.142	-0.790	88,4846,79933	ACTN2,NOS3,SYNPO2L	3/-
1	6_Member	GO Biological Processes	GO:0007507	heart development	-3.142	-0.790	88,4846,79933	ACTN2,NOS3,SYNPO2L	3/570
2	1_Summary	GO Biological Processes	GO:0032372	negative regulation of sterol transport	-7.223	-3.270	64240,64241,255738	ABCG5,ABCG8,PCSK9	3/-
2	1_Member	GO Biological Processes	GO:0032372	negative regulation of sterol transport	-7.223	-3.270	64240,64241,255738	ABCG5,ABCG8,PCSK9	3/32
2	1_Member	GO Biological Processes	GO:0032375	negative regulation of cholesterol transport	-7.223	-3.270	64240,64241,255738	ABCG5,ABCG8,PCSK9	3/32
2	1_Member	GO Biological Processes	GO:0032369	negative regulation of lipid transport	-6.654	-2.948	64240,64241,255738	ABCG5,ABCG8,PCSK9	3/49
2	1_Member	KEGG Pathway	hsa04979	Cholesterol metabolism	-6.601	-2.948	64240,64241,255738	ABCG5,ABCG8,PCSK9	3/51
2	1_Member	GO Biological Processes	GO:1905953	negative regulation of lipid localization	-6.240	-2.684	64240,64241,255738	ABCG5,ABCG8,PCSK9	3/67
2	1_Member	GO Biological Processes	GO:0032371	regulation of sterol transport	-5.958	-2.549	64240,64241,255738	ABCG5,ABCG8,PCSK9	3/83
2	1_Member	GO Biological Processes	GO:0032374	regulation of cholesterol transport	-5.958	-2.549	64240,64241,255738	ABCG5,ABCG8,PCSK9	3/83
2	1_Member	GO Biological Processes	GO:0042632	cholesterol homeostasis	-5.701	-2.387	64240,64241,255738	ABCG5,ABCG8,PCSK9	3/101
2	1_Member	GO Biological Processes	GO:0055092	sterol homeostasis	-5.688	-2.387	64240,64241,255738	ABCG5,ABCG8,PCSK9	3/102
2	1_Member	GO Biological Processes	GO:0032368	regulation of lipid transport	-5.133	-1.878	64240,64241,255738	ABCG5,ABCG8,PCSK9	3/156
2	1_Member	GO Biological Processes	GO:0055088	lipid homeostasis	-4.976	-1.762	64240,64241,255738	ABCG5,ABCG8,PCSK9	3/176
2	1_Member	GO Biological Processes	GO:1905952	regulation of lipid localization	-4.904	-1.728	64240,64241,255738	ABCG5,ABCG8,PCSK9	3/186
2	1_Member	GO Biological Processes	GO:0051051	negative regulation of transport	-3.729	-0.588	64240,64241,255738	ABCG5,ABCG8,PCSK9	3/462
2	1_Member	GO Biological Processes	GO:0031667	response to nutrient levels	-3.683	-0.574	64240,64241,255738	ABCG5,ABCG8,PCSK9	3/479
2	1_Member	GO Biological Processes	GO:0009991	response to extracellular stimulus	-3.608	-0.529	64240,64241,255738	ABCG5,ABCG8,PCSK9	3/508
2	1_Member	Reactome Gene Sets	R-HSA-382551	Transport of small molecules	-3.135	-0.084	64240,64241,255738	ABCG5,ABCG8,PCSK9	3/737
2	2_Summary	GO Biological Processes	GO:0061024	membrane organization	-3.096	-0.071	64240,64241,375056	ABCG5,ABCG8,MIA3	3/-
2	2_Member	GO Biological Processes	GO:0061024	membrane organization	-3.096	-0.071	64240,64241,375056	ABCG5,ABCG8,MIA3	3/760
3	1_Summary	GO Biological Processes	GO:0006913	nucleocytoplasmic transport	-6.704	-2.741	1026,5976,6428,7040,7919,6631,10949	CDKN1A,UPF1,SRSF3,TGFB1,DDX39B,SNRPC,HNRNPA(	7/-
3	1_Member	GO Biological Processes	GO:0006913	nucleocytoplasmic transport	-6.704	-2.741	1026,5976,6428,7040,7919	CDKN1A,UPF1,SRSF3,TGFB1,DDX39B	5/247

3	1_Member	GO Biological Processes	GO:0051169	nuclear transport	-6.695	-2.741	1026,5976,6428,7040,7919	CDKN1A,UPF1,SRSF3,TGFB1,DDX39B	5/248
3	1_Member	GO Biological Processes	GO:0051168	nuclear export	-6.092	-2.314	5976,6428,7040,7919	UPF1,SRSF3,TGFB1,DDX39B	4/133
3	1_Member	GO Biological Processes	GO:0006406	mRNA export from nucleus	-5.182	-1.626	5976,6428,7919	UPF1,SRSF3,DDX39B	3/66
3	1_Member	GO Biological Processes	GO:0006405	RNA export from nucleus	-4.882	-1.405	5976,6428,7919	UPF1,SRSF3,DDX39B	3/83
3	1_Member	GO Biological Processes	GO:0016071	mRNA metabolic process	-4.666	-1.256	5976,6428,6631,7919,10949	UPF1,SRSF3,SNRPC,DDX39B,HNRNPA0	5/645
3	1_Member	GO Biological Processes	GO:0051028	mRNA transport	-4.339	-1.075	5976,6428,7919	UPF1,SRSF3,DDX39B	3/126
3	1_Member	GO Biological Processes	GO:0050657	nucleic acid transport	-4.088	-1.004	5976,6428,7919	UPF1,SRSF3,DDX39B	3/153
3	1_Member	GO Biological Processes	GO:0050658	RNA transport	-4.088	-1.004	5976,6428,7919	UPF1,SRSF3,DDX39B	3/153
3	1_Member	GO Biological Processes	GO:0051236	establishment of RNA localization	-4.072	-1.004	5976,6428,7919	UPF1,SRSF3,DDX39B	3/155
3	1_Member	GO Biological Processes	GO:0006403	RNA localization	-3.915	-0.916	5976,6428,7919	UPF1,SRSF3,DDX39B	3/175
3	1_Member	GO Biological Processes	GO:0006397	mRNA processing	-3.872	-0.896	6428,6631,7919,10949	SRSF3,SNRPC,DDX39B,HNRNPA0	4/488
3	1_Member	Reactome Gene Sets	R-HSA-72163	mRNA Splicing - Major Pathway	-3.700	-0.807	6428,6631,7919	SRSF3,SNRPC,DDX39B	3/207
3	1_Member	Reactome Gene Sets	R-HSA-72172	mRNA Splicing	-3.652	-0.803	6428,6631,7919	SRSF3,SNRPC,DDX39B	3/215
3	1_Member	KEGG Pathway	hsa03040	Spliceosome	-3.640	-0.803	6428,6631,7919	SRSF3,SNRPC,DDX39B	3/217
3	1_Member	GO Biological Processes	GO:0015931	nucleobase-containing compound transport	-3.623	-0.803	5976,6428,7919	UPF1,SRSF3,DDX39B	3/220
3	1_Member	GO Biological Processes	GO:0000377	RNA splicing, via transesterification reactions with bulged ad	-3.339	-0.603	6428,6631,7919	SRSF3,SNRPC,DDX39B	3/275
3	1_Member	GO Biological Processes	GO:0000398	mRNA splicing, via spliceosome	-3.339	-0.603	6428,6631,7919	SRSF3,SNRPC,DDX39B	3/275
3	1_Member	GO Biological Processes	GO:0000375	RNA splicing, via transesterification reactions	-3.321	-0.597	6428,6631,7919	SRSF3,SNRPC,DDX39B	3/279
3	1_Member	Reactome Gene Sets	R-HSA-72203	Processing of Capped Intron-Containing Pre-mRNA	-3.294	-0.583	6428,6631,7919	SRSF3,SNRPC,DDX39B	3/285
3	1_Member	Reactome Gene Sets	R-HSA-8953854	Metabolism of RNA	-3.216	-0.518	5976,6428,6631,7919	UPF1,SRSF3,SNRPC,DDX39B	4/726
3	1_Member	GO Biological Processes	GO:0008380	RNA splicing	-2.837	-0.255	6428,6631,7919	SRSF3,SNRPC,DDX39B	3/410
3	2_Summary	KEGG Pathway	hsa05226	Gastric cancer	-5.895	-2.242	1026,1030,2250,7040,84152,199,5328,5976	CDKN1A,CDKN2B,FGF5,TGFB1,PPP1R1B,AIF1,PLAU,UPF	8/-
3	2_Member	KEGG Pathway	hsa05226	Gastric cancer	-5.895	-2.242	1026,1030,2250,7040	CDKN1A,CDKN2B,FGF5,TGFB1	4/149
3	2_Member	GO Biological Processes	GO:0045936	negative regulation of phosphate metabolic process	-4.301	-1.075	1026,1030,7040,84152	CDKN1A,CDKN2B,TGFB1,PPP1R1B	4/378
3	2_Member	GO Biological Processes	GO:0010563	negative regulation of phosphorus metabolic process	-4.297	-1.075	1026,1030,7040,84152	CDKN1A,CDKN2B,TGFB1,PPP1R1B	4/379
3	2_Member	KEGG Pathway	hsa04068	FoxO signaling pathway	-4.289	-1.075	1026,1030,7040	CDKN1A,CDKN2B,TGFB1	3/131
3	2_Member	KEGG Pathway	hsa04218	Cellular senescence	-4.063	-1.004	1026,1030,7040	CDKN1A,CDKN2B,TGFB1	3/156
3	2_Member	KEGG Pathway	hsa04110	Cell cycle	-4.055	-1.004	1026,1030,7040	CDKN1A,CDKN2B,TGFB1	3/157
3	2_Member	GO Biological Processes	GO:0048660	regulation of smooth muscle cell proliferation	-3.923	-0.916	199,1026,7040	AIF1,CDKN1A,TGFB1	3/174
3	2_Member	GO Biological Processes	GO:2000045	regulation of G1/S transition of mitotic cell cycle	-3.844	-0.890	199,1026,1030	AIF1,CDKN1A,CDKN2B	3/185
3	2_Member	KEGG Pathway	hsa05200	Pathways in cancer	-3.732	-0.813	1026,1030,2250,7040	CDKN1A,CDKN2B,FGF5,TGFB1	4/531
3	2_Member	KEGG Pathway	hsa05205	Proteoglycans in cancer	-3.725	-0.813	1026,5328,7040	CDKN1A,PLAU,TGFB1	3/203
3	2_Member	GO Biological Processes	GO:1902806	regulation of cell cycle G1/S phase transition	-3.676	-0.803	199,1026,1030	AIF1,CDKN1A,CDKN2B	3/211
3	2_Member	KEGG Pathway	hsa05166	Human T-cell leukemia virus 1 infection	-3.611	-0.803	1026,1030,7040	CDKN1A,CDKN2B,TGFB1	3/222
3	2_Member	GO Biological Processes	GO:0042326	negative regulation of phosphorylation	-3.163	-0.480	1026,1030,7040	CDKN1A,CDKN2B,TGFB1	3/316

3	2_Member	GO Biological Processes	GO:0042060	wound healing	-3.155	-0.480	1026,5328,7040	CDKN1A,PLAU,TGFB1	3/318
3	2_Member	GO Biological Processes	GO:0051251	positive regulation of lymphocyte activation	-3.117	-0.453	199,1026,7040	AIF1,CDKN1A,TGFB1	3/328
3	2_Member	GO Biological Processes	GO:0008285	negative regulation of cell population proliferation	-3.077	-0.424	199,1026,1030,7040	AIF1,CDKN1A,CDKN2B,TGFB1	4/791
3	2_Member	GO Biological Processes	GO:0070482	response to oxygen levels	-3.064	-0.422	1026,5328,7040	CDKN1A,PLAU,TGFB1	3/342
3	2_Member	GO Biological Processes	GO:1901990	regulation of mitotic cell cycle phase transition	-3.014	-0.382	199,1026,1030	AIF1,CDKN1A,CDKN2B	3/356
3	2_Member	GO Biological Processes	GO:0002696	positive regulation of leukocyte activation	-2.993	-0.371	199,1026,7040	AIF1,CDKN1A,TGFB1	3/362
3	2_Member	GO Biological Processes	GO:0045786	negative regulation of cell cycle	-2.932	-0.330	1026,1030,7040	CDKN1A,CDKN2B,TGFB1	3/380
3	2_Member	GO Biological Processes	GO:0050867	positive regulation of cell activation	-2.932	-0.330	199,1026,7040	AIF1,CDKN1A,TGFB1	3/380
3	2_Member	GO Biological Processes	GO:0009611	response to wounding	-2.790	-0.225	1026,5328,7040	CDKN1A,PLAU,TGFB1	3/426
3	2_Member	GO Biological Processes	GO:0031400	negative regulation of protein modification process	-2.790	-0.225	1026,7040,84152	CDKN1A,TGFB1,PPP1R1B	3/426
3	2_Member	GO Biological Processes	GO:1901987	regulation of cell cycle phase transition	-2.708	-0.161	199,1026,1030	AIF1,CDKN1A,CDKN2B	3/455
3	2_Member	Reactome Gene Sets	R-HSA-5663202	Diseases of signal transduction by growth factor receptors	-2.697	-0.158	1026,2250,7040	CDKN1A,FGF5,TGFB1	3/459
3	2_Member	GO Biological Processes	GO:0071363	cellular response to growth factor stimulus	-2.622	-0.091	1030,2250,7040	CDKN2B,FGF5,TGFB1	3/488
3	2_Member	GO Biological Processes	GO:0051249	regulation of lymphocyte activation	-2.577	-0.055	199,1026,7040	AIF1,CDKN1A,TGFB1	3/506
3	2_Member	GO Biological Processes	GO:0070848	response to growth factor	-2.544	-0.033	1030,2250,7040	CDKN2B,FGF5,TGFB1	3/520
3	2_Member	GO Biological Processes	GO:0007346	regulation of mitotic cell cycle	-2.539	-0.033	199,1026,1030	AIF1,CDKN1A,CDKN2B	3/522
3	2_Member	GO Biological Processes	GO:0051052	regulation of DNA metabolic process	-2.532	-0.033	1026,5976,7040	CDKN1A,UPF1,TGFB1	3/525
3	2_Member	GO Biological Processes	GO:0002694	regulation of leukocyte activation	-2.425	0.000	199,1026,7040	AIF1,CDKN1A,TGFB1	3/573
3	2_Member	GO Biological Processes	GO:0001934	positive regulation of protein phosphorylation	-2.414	0.000	1026,2250,7040	CDKN1A,FGF5,TGFB1	3/578
3	2_Member	GO Biological Processes	GO:0042327	positive regulation of phosphorylation	-2.323	0.000	1026,2250,7040	CDKN1A,FGF5,TGFB1	3/623
3	2_Member	GO Biological Processes	GO:0050865	regulation of cell activation	-2.310	0.000	199,1026,7040	AIF1,CDKN1A,TGFB1	3/630
3	2_Member	GO Biological Processes	GO:0010562	positive regulation of phosphorus metabolic process	-2.179	0.000	1026,2250,7040	CDKN1A,FGF5,TGFB1	3/702
3	2_Member	GO Biological Processes	GO:0045937	positive regulation of phosphate metabolic process	-2.179	0.000	1026,2250,7040	CDKN1A,FGF5,TGFB1	3/702
3	2_Member	GO Biological Processes	GO:0010564	regulation of cell cycle process	-2.107	0.000	199,1026,1030	AIF1,CDKN1A,CDKN2B	3/746
3	2_Member	GO Biological Processes	GO:0031401	positive regulation of protein modification process	-2.078	0.000	1026,2250,7040	CDKN1A,FGF5,TGFB1	3/764
3	3_Summary	GO Biological Processes	GO:0030335	positive regulation of cell migration	-3.585	-0.793	199,5328,7040,84299,10949	AIF1,PLAU,TGFB1,MIEN1,HNRNPA0	5/-
3	3_Member	GO Biological Processes	GO:0030335	positive regulation of cell migration	-3.585	-0.793	199,5328,7040,84299	AIF1,PLAU,TGFB1,MIEN1	4/580
3	3_Member	GO Biological Processes	GO:2000147	positive regulation of cell motility	-3.510	-0.732	199,5328,7040,84299	AIF1,PLAU,TGFB1,MIEN1	4/607
3	3_Member	GO Biological Processes	GO:0040017	positive regulation of locomotion	-3.473	-0.709	199,5328,7040,84299	AIF1,PLAU,TGFB1,MIEN1	4/621
3	3_Member	GO Biological Processes	GO:0032102	negative regulation of response to external stimulus	-2.714	-0.161	199,5328,7040	AIF1,PLAU,TGFB1	3/453
3	3_Member	GO Biological Processes	GO:0032103	positive regulation of response to external stimulus	-2.451	0.000	199,5328,7040	AIF1,PLAU,TGFB1	3/561
3	3_Member	GO Biological Processes	GO:0006954	inflammatory response	-2.446	0.000	199,7040,10949	AIF1,TGFB1,HNRNPA0	3/563
3	4_Summary	GO Biological Processes	GO:1903311	regulation of mRNA metabolic process	-2.856	-0.264	5976,6428,10949,55876,199	UPF1,SRSF3,HNRNPA0,GSDMB,AIF1	5/-
3	4_Member	GO Biological Processes	GO:1903311	regulation of mRNA metabolic process	-2.856	-0.264	5976,6428,10949	UPF1,SRSF3,HNRNPA0	3/404
3	4_Member	GO Biological Processes	GO:0009617	response to bacterium	-2.166	0.000	5976,10949,55876	UPF1,HNRNPA0,GSDMB	3/710

3	4_Member	GO Biological Processes	GO:0071345	cellular response to cytokine stimulus	-2.120	0.000	199,5976,6428	AIF1,UPF1,SRSF3	3/738
4	No enriched terms								
5	No enriched terms								

Supplementary Table 4F: All-cause heart failure in a European population

MCODE App Results

Date: Jul 15, 2024, 11:35:36 PM

Parameters:

Network Scoring:

Include Loops: false Degree Cutoff: 3

Cluster Finding:

Node Score Cutoff: 0.2 Haircut: true Fluff: false K-Core: 2 Max. Depth from Seed: 100

Cluster	Score (Density*	Nodes	Edges	Node IDs
1	4.889	10	22	ITIH4, APOH, HLA-DQA1, HP, SEC24C, HLA-DQB2, C4A, HLA-DRB1, HPR, HLA-DQA2
2	4	9	16	PSRC1, MYOZ1, FLNC, SYNPO2L, ACTN2, ZC3HC1, PHACTR1, MIA3, HHIPL1
3	3	3	3	TGFB1, NCF1, AIF1

Metascape Enrichment

Cluster	GroupID	Category	Term	Description	LogP	Log(q-value)	Genes	Symbols	InTerm_InList
1	1_Summary	GO Biological Processes	GO:0002399	MHC class II protein complex assembly	-10.963	-7.009	3117,3118,3120,3123,9632,720	HLA-DQA1,HLA-DQA2,HLA-DQB2,HLA-DRB1,SEC24C,C4A	6/-
1	1_Member	GO Biological Processes	GO:0002399	MHC class II protein complex assembly	-10.963	-7.009	3117,3118,3120,3123	HLA-DQA1,HLA-DQA2,HLA-DQB2,HLA-DRB1	4/16
1	1_Member	GO Biological Processes	GO:0002503	peptide antigen assembly with MHC class II protein complex	-10.963	-7.009	3117,3118,3120,3123	HLA-DQA1,HLA-DQA2,HLA-DQB2,HLA-DRB1	4/16
1	1_Member	Reactome Gene Sets	R-HSA-202430	Translocation of ZAP-70 to Immunological synapse	-10.635	-6.891	3117,3118,3120,3123	HLA-DQA1,HLA-DQA2,HLA-DQB2,HLA-DRB1	4/19
1	1_Member	GO Biological Processes	GO:0002396	MHC protein complex assembly	-10.447	-6.891	3117,3118,3120,3123	HLA-DQA1,HLA-DQA2,HLA-DQB2,HLA-DRB1	4/21
1	1_Member	GO Biological Processes	GO:0002501	peptide antigen assembly with MHC protein complex	-10.447	-6.891	3117,3118,3120,3123	HLA-DQA1,HLA-DQA2,HLA-DQB2,HLA-DRB1	4/21
1	1_Member	Reactome Gene Sets	R-HSA-202427	Phosphorylation of CD3 and TCR zeta chains	-10.359	-6.883	3117,3118,3120,3123	HLA-DQA1,HLA-DQA2,HLA-DQB2,HLA-DRB1	4/22
1	1_Member	Reactome Gene Sets	R-HSA-389948	PD-1 signaling	-10.277	-6.867	3117,3118,3120,3123	HLA-DQA1,HLA-DQA2,HLA-DQB2,HLA-DRB1	4/23
1	1_Member	GO Biological Processes	GO:0019886	antigen processing and presentation of exogenous peptide antigen via MHC class II	-9.726	-6.382	3117,3118,3120,3123	HLA-DQA1,HLA-DQA2,HLA-DQB2,HLA-DRB1	4/31
1	1_Member	GO Biological Processes	GO:0002495	antigen processing and presentation of peptide antigen via MHC class II	-9.613	-6.382	3117,3118,3120,3123	HLA-DQA1,HLA-DQA2,HLA-DQB2,HLA-DRB1	4/33
1	1_Member	Reactome Gene Sets	R-HSA-2132295	MHC class II antigen presentation	-9.598	-6.382	3117,3118,3120,3123,9632	HLA-DQA1,HLA-DQA2,HLA-DQB2,HLA-DRB1,SEC24C	5/123
1	1_Member	GO Biological Processes	GO:0002504	antigen processing and presentation of peptide or polysaccharide antigen via MHC class II	-9.558	-6.382	3117,3118,3120,3123	HLA-DQA1,HLA-DQA2,HLA-DQB2,HLA-DRB1	4/34
1	1_Member	Reactome Gene Sets	R-HSA-202433	Generation of second messenger molecules	-9.558	-6.382	3117,3118,3120,3123	HLA-DQA1,HLA-DQA2,HLA-DQB2,HLA-DRB1	4/34
1	1_Member	GO Biological Processes	GO:0002478	antigen processing and presentation of exogenous peptide antigen	-9.220	-6.079	3117,3118,3120,3123	HLA-DQA1,HLA-DQA2,HLA-DQB2,HLA-DRB1	4/41
1	1_Member	GO Biological Processes	GO:0019884	antigen processing and presentation of exogenous antigen	-8.863	-5.755	3117,3118,3120,3123	HLA-DQA1,HLA-DQA2,HLA-DQB2,HLA-DRB1	4/50
1	1_Member	Reactome Gene Sets	R-HSA-388841	Costimulation by the CD28 family	-8.290	-5.214	3117,3118,3120,3123	HLA-DQA1,HLA-DQA2,HLA-DQB2,HLA-DRB1	4/69
1	1_Member	GO Biological Processes	GO:0048002	antigen processing and presentation of peptide antigen	-8.265	-5.214	3117,3118,3120,3123	HLA-DQA1,HLA-DQA2,HLA-DQB2,HLA-DRB1	4/70

1	1_Member	Reactome Gene Sets	R-HSA-877300	Interferon gamma signaling	-7.707	-4.708	3117,3118,3120,3123	HLA-DQA1,HLA-DQA2,HLA-DQB2,HLA-DRB1	4/96
1	1_Member	KEGG Pathway	hsa05150	Staphylococcus aureus infection	-7.707	-4.708	720,3117,3118,3123	C4A,HLA-DQA1,HLA-DQA2,HLA-DRB1	4/96
1	1_Member	Reactome Gene Sets	R-HSA-202424	Downstream TCR signaling	-7.671	-4.695	3117,3118,3120,3123	HLA-DQA1,HLA-DQA2,HLA-DQB2,HLA-DRB1	4/98
1	1_Member	GO Biological Processes	GO:0019882	antigen processing and presentation	-7.567	-4.613	3117,3118,3120,3123	HLA-DQA1,HLA-DQA2,HLA-DQB2,HLA-DRB1	4/104
1	1_Member	Reactome Gene Sets	R-HSA-202403	TCR signaling	-7.316	-4.383	3117,3118,3120,3123	HLA-DQA1,HLA-DQA2,HLA-DQB2,HLA-DRB1	4/120
1	1_Member	KEGG Pathway	hsa05322	Systemic lupus erythematosus	-7.084	-4.172	720,3117,3118,3123	C4A,HLA-DQA1,HLA-DQA2,HLA-DRB1	4/137
1	1_Member	KEGG Pathway	hsa05310	Asthma	-6.936	-4.043	3117,3118,3123	HLA-DQA1,HLA-DQA2,HLA-DRB1	3/31
1	1_Member	KEGG Pathway	hsa05330	Allograft rejection	-6.663	-3.788	3117,3118,3123	HLA-DQA1,HLA-DQA2,HLA-DRB1	3/38
1	1_Member	KEGG Pathway	hsa05332	Graft-versus-host disease	-6.529	-3.672	3117,3118,3123	HLA-DQA1,HLA-DQA2,HLA-DRB1	3/42
1	1_Member	KEGG Pathway	hsa04940	Type I diabetes mellitus	-6.498	-3.658	3117,3118,3123	HLA-DQA1,HLA-DQA2,HLA-DRB1	3/43
1	1_Member	KEGG Pathway	hsa04672	Intestinal immune network for IgA production	-6.324	-3.501	3117,3118,3123	HLA-DQA1,HLA-DQA2,HLA-DRB1	3/49
1	1_Member	KEGG Pathway	hsa05320	Autoimmune thyroid disease	-6.220	-3.413	3117,3118,3123	HLA-DQA1,HLA-DQA2,HLA-DRB1	3/53
1	1_Member	GO Biological Processes	GO:0050870	positive regulation of T cell activation	-6.018	-3.225	3117,3118,3120,3123	HLA-DQA1,HLA-DQA2,HLA-DQB2,HLA-DRB1	4/253
1	1_Member	GO Biological Processes	GO:0002250	adaptive immune response	-5.976	-3.209	720,3117,3118,3120,3123	C4A,HLA-DQA1,HLA-DQA2,HLA-DQB2,HLA-DRB1	5/653
1	1_Member	GO Biological Processes	GO:0050778	positive regulation of immune response	-5.973	-3.209	720,3117,3118,3120,3123	C4A,HLA-DQA1,HLA-DQA2,HLA-DQB2,HLA-DRB1	5/654
1	1_Member	KEGG Pathway	hsa05321	Inflammatory bowel disease	-5.951	-3.201	3117,3118,3123	HLA-DQA1,HLA-DQA2,HLA-DRB1	3/65
1	1_Member	Reactome Gene Sets	R-HSA-913531	Interferon Signaling	-5.912	-3.175	3117,3118,3120,3123	HLA-DQA1,HLA-DQA2,HLA-DQB2,HLA-DRB1	4/269
1	1_Member	KEGG Pathway	hsa05416	Viral myocarditis	-5.872	-3.150	3117,3118,3123	HLA-DQA1,HLA-DQA2,HLA-DRB1	3/69
1	1_Member	GO Biological Processes	GO:1903039	positive regulation of leukocyte cell-cell adhesion	-5.861	-3.150	3117,3118,3120,3123	HLA-DQA1,HLA-DQA2,HLA-DQB2,HLA-DRB1	4/277
1	1_Member	KEGG Pathway	hsa05140	Leishmaniasis	-5.728	-3.029	3117,3118,3123	HLA-DQA1,HLA-DQA2,HLA-DRB1	3/77
1	1_Member	KEGG Pathway	hsa04612	Antigen processing and presentation	-5.711	-3.024	3117,3118,3123	HLA-DQA1,HLA-DQA2,HLA-DRB1	3/78
1	1_Member	Reactome Gene Sets	R-HSA-1280218	Adaptive Immune System	-5.630	-2.954	3117,3118,3120,3123,9632	HLA-DQA1,HLA-DQA2,HLA-DQB2,HLA-DRB1,SEC24C	5/768
1	1_Member	GO Biological Processes	GO:0022409	positive regulation of cell-cell adhesion	-5.585	-2.922	3117,3118,3120,3123	HLA-DQA1,HLA-DQA2,HLA-DQB2,HLA-DRB1	4/325
1	1_Member	GO Biological Processes	GO:0051251	positive regulation of lymphocyte activation	-5.570	-2.917	3117,3118,3120,3123	HLA-DQA1,HLA-DQA2,HLA-DQB2,HLA-DRB1	4/328
1	1_Member	KEGG Pathway	hsa04658	Th1 and Th2 cell differentiation	-5.494	-2.852	3117,3118,3123	HLA-DQA1,HLA-DQA2,HLA-DRB1	3/92
1	1_Member	KEGG Pathway	hsa05323	Rheumatoid arthritis	-5.480	-2.848	3117,3118,3123	HLA-DQA1,HLA-DQA2,HLA-DRB1	3/93
1	1_Member	GO Biological Processes	GO:0002696	positive regulation of leukocyte activation	-5.400	-2.787	3117,3118,3120,3123	HLA-DQA1,HLA-DQA2,HLA-DQB2,HLA-DRB1	4/362
1	1_Member	KEGG Pathway	hsa04640	Hematopoietic cell lineage	-5.398	-2.787	3117,3118,3123	HLA-DQA1,HLA-DQA2,HLA-DRB1	3/99
1	1_Member	GO Biological Processes	GO:0050867	positive regulation of cell activation	-5.316	-2.725	3117,3118,3120,3123	HLA-DQA1,HLA-DQA2,HLA-DQB2,HLA-DRB1	4/380
1	1_Member	GO Biological Processes	GO:0050863	regulation of T cell activation	-5.307	-2.725	3117,3118,3120,3123	HLA-DQA1,HLA-DQA2,HLA-DQB2,HLA-DRB1	4/382
1	1_Member	GO Biological Processes	GO:1903037	regulation of leukocyte cell-cell adhesion	-5.307	-2.725	3117,3118,3120,3123	HLA-DQA1,HLA-DQA2,HLA-DQB2,HLA-DRB1	4/382
1	1_Member	KEGG Pathway	hsa04659	Th17 cell differentiation	-5.284	-2.711	3117,3118,3123	HLA-DQA1,HLA-DQA2,HLA-DRB1	3/108
1	1_Member	KEGG Pathway	hsa05145	Toxoplasmosis	-5.248	-2.684	3117,3118,3123	HLA-DQA1,HLA-DQA2,HLA-DRB1	3/111
1	1_Member	GO Biological Processes	GO:0045785	positive regulation of cell adhesion	-4.898	-2.342	3117,3118,3120,3123	HLA-DQA1,HLA-DQA2,HLA-DQB2,HLA-DRB1	4/485
1	1_Member	GO Biological Processes	GO:0022407	regulation of cell-cell adhesion	-4.860	-2.313	3117,3118,3120,3123	HLA-DQA1,HLA-DQA2,HLA-DQB2,HLA-DRB1	4/496

1	1_Member	KEGG Pathway	hsa04145	Phagosome	-4.839	-2.300	3117,3118,3123	HLA-DQA1,HLA-DQA2,HLA-DRB1	3/152
1	1_Member	GO Biological Processes	GO:0051249	regulation of lymphocyte activation	-4.826	-2.295	3117,3118,3120,3123	HLA-DQA1,HLA-DQA2,HLA-DQB2,HLA-DRB1	4/506
1	1_Member	KEGG Pathway	hsa04514	Cell adhesion molecules	-4.797	-2.274	3117,3118,3123	HLA-DQA1,HLA-DQA2,HLA-DRB1	3/157
1	1_Member	KEGG Pathway	hsa05164	Influenza A	-4.686	-2.171	3117,3118,3123	HLA-DQA1,HLA-DQA2,HLA-DRB1	3/171
1	1_Member	KEGG Pathway	hsa05152	Tuberculosis	-4.619	-2.123	3117,3118,3123	HLA-DQA1,HLA-DQA2,HLA-DRB1	3/180
1	1_Member	GO Biological Processes	GO:0002694	regulation of leukocyte activation	-4.614	-2.123	3117,3118,3120,3123	HLA-DQA1,HLA-DQA2,HLA-DQB2,HLA-DRB1	4/573
1	1_Member	KEGG Pathway	hsa05169	Epstein-Barr virus infection	-4.470	-1.986	3117,3118,3123	HLA-DQA1,HLA-DQA2,HLA-DRB1	3/202
1	1_Member	GO Biological Processes	GO:0050865	regulation of cell activation	-4.453	-1.976	3117,3118,3120,3123	HLA-DQA1,HLA-DQA2,HLA-DQB2,HLA-DRB1	4/630
1	1_Member	KEGG Pathway	hsa05166	Human T-cell leukemia virus 1 infection	-4.348	-1.878	3117,3118,3123	HLA-DQA1,HLA-DQA2,HLA-DRB1	3/222
1	1_Member	Reactome Gene Sets	R-HSA-1280215	Cytokine Signaling in Immune system	-4.079	-1.616	3117,3118,3120,3123	HLA-DQA1,HLA-DQA2,HLA-DQB2,HLA-DRB1	4/786
1	1_Member	KEGG Pathway	hsa05168	Herpes simplex virus 1 infection	-3.278	-0.822	3117,3118,3123	HLA-DQA1,HLA-DQA2,HLA-DRB1	3/512
1	2_Summary	GO Biological Processes	GO:0006954	inflammatory response	-4.644	-2.137	720,3123,3240,3700	C4A,HLA-DRB1,HP,ITIH4	4/-
1	2_Member	GO Biological Processes	GO:0006954	inflammatory response	-4.644	-2.137	720,3123,3240,3700	C4A,HLA-DRB1,HP,ITIH4	4/563
1	3_Summary	Reactome Gene Sets	R-HSA-5653656	Vesicle-mediated transport	-2.933	-0.484	3240,3250,9632	HP,HPR,SEC24C	3/-
1	3_Member	Reactome Gene Sets	R-HSA-5653656	Vesicle-mediated transport	-2.933	-0.484	3240,3250,9632	HP,HPR,SEC24C	3/673
2	1_Summary	GO Biological Processes	GO:0031032	actomyosin structure organization	-9.880	-5.625	88,2318,58529,79933,221692	ACTN2,FLNC,MYOZ1,SYNPO2L,PHACTR1	5/-
2	1_Member	GO Biological Processes	GO:0031032	actomyosin structure organization	-9.880	-5.625	88,2318,58529,79933,221692	ACTN2,FLNC,MYOZ1,SYNPO2L,PHACTR1	5/124
2	1_Member	GO Biological Processes	GO:0045214	sarcomere organization	-9.314	-5.360	88,2318,58529,79933	ACTN2,FLNC,MYOZ1,SYNPO2L	4/44
2	1_Member	GO Biological Processes	GO:0030239	myofibril assembly	-8.486	-4.783	88,2318,58529,79933	ACTN2,FLNC,MYOZ1,SYNPO2L	4/70
2	1_Member	GO Biological Processes	GO:0055002	striated muscle cell development	-8.436	-4.783	88,2318,58529,79933	ACTN2,FLNC,MYOZ1,SYNPO2L	4/72
2	1_Member	GO Biological Processes	GO:0010927	cellular component assembly involved in morphogenesis	-7.493	-3.937	88,2318,58529,79933	ACTN2,FLNC,MYOZ1,SYNPO2L	4/123
2	1_Member	GO Biological Processes	GO:0055001	muscle cell development	-6.877	-3.400	88,2318,58529,79933	ACTN2,FLNC,MYOZ1,SYNPO2L	4/175
2	1_Member	GO Biological Processes	GO:0030036	actin cytoskeleton organization	-6.662	-3.252	88,2318,58529,79933,221692	ACTN2,FLNC,MYOZ1,SYNPO2L,PHACTR1	5/544
2	1_Member	GO Biological Processes	GO:0097435	supramolecular fiber organization	-6.480	-3.188	88,2318,58529,79933,221692	ACTN2,FLNC,MYOZ1,SYNPO2L,PHACTR1	5/592
2	1_Member	GO Biological Processes	GO:0032989	cellular anatomical entity morphogenesis	-6.433	-3.188	88,2318,58529,79933,221692	ACTN2,FLNC,MYOZ1,SYNPO2L,PHACTR1	5/605
2	1_Member	GO Biological Processes	GO:0051146	striated muscle cell differentiation	-6.425	-3.188	88,2318,58529,79933	ACTN2,FLNC,MYOZ1,SYNPO2L	4/227
2	1_Member	GO Biological Processes	GO:0030029	actin filament-based process	-6.401	-3.188	88,2318,58529,79933,221692	ACTN2,FLNC,MYOZ1,SYNPO2L,PHACTR1	5/614
2	1_Member	GO Biological Processes	GO:0042692	muscle cell differentiation	-6.006	-2.831	88,2318,58529,79933	ACTN2,FLNC,MYOZ1,SYNPO2L	4/289
2	1_Member	GO Biological Processes	GO:0140694	non-membrane-bounded organelle assembly	-5.716	-2.575	88,2318,58529,79933	ACTN2,FLNC,MYOZ1,SYNPO2L	4/342
2	1_Member	GO Biological Processes	GO:0061061	muscle structure development	-4.995	-1.887	88,2318,58529,79933	ACTN2,FLNC,MYOZ1,SYNPO2L	4/520
2	1_Member	KEGG Pathway	hsa04820	Cytoskeleton in muscle cells	-4.460	-1.436	88,2318,58529	ACTN2,FLNC,MYOZ1	3/229
2	2_Summary	GO Biological Processes	GO:1902905	positive regulation of supramolecular fiber organization	-4.809	-1.730	88,79933,84722	ACTN2,SYNPO2L,PSRC1	3/-
2	2_Member	GO Biological Processes	GO:1902905	positive regulation of supramolecular fiber organization	-4.809	-1.730	88,79933,84722	ACTN2,SYNPO2L,PSRC1	3/175
2	2_Member	GO Biological Processes	GO:0051495	positive regulation of cytoskeleton organization	-4.744	-1.693	88,79933,84722	ACTN2,SYNPO2L,PSRC1	3/184
2	2_Member	GO Biological Processes	GO:1902903	regulation of supramolecular fiber organization	-3.768	-0.768	88,79933,84722	ACTN2,SYNPO2L,PSRC1	3/392

2	2_Member	GO Biological Processes	GO:0010638	positive regulation of organelle organization	-3.447	-0.471	88,79933,84722	ACTN2,SYNPO2L,PSRC1	3/504
2	2_Member	GO Biological Processes	GO:0051493	regulation of cytoskeleton organization	-3.400	-0.446	88,79933,84722	ACTN2,SYNPO2L,PSRC1	3/523
3	No enriched terms								
